# GABA_B_R activation alleviates *Streptococcus equi* subsp. *zooepidemicus*-induced acute lung injury by suppressing inflammation and oxidative stress in pulmonary macrophages via IRAK3

**DOI:** 10.1128/spectrum.01040-26

**Published:** 2026-10-06

**Authors:** Ziteng Deng, Huilin Liu, Yijiang Yang, Wenshu Yi, Deding Wu, Xiaoqing Cheng, Anqi Lu, Yiran Chen, Yajuan Li, Yaqiong Ye, Jiedan Liao, Yunfei Huang, Qiang Fu

**Affiliations:** 1 School of Animal Science and Technology, Foshan University 47868, Foshan, Guangdong, China; 2 Foshan University Veterinary Teaching Hospital, Foshan University 47868, Foshan, Guangdong, China; Ross University School of Veterinary Medicine, Basseterre, Saint Kitts and Nevis

**Keywords:** *Streptococcus equi *subsp. *zooepidemicus*, acute lung injury, macrophage, GABA_B_R, IRAK3

## Abstract

*Streptococcus equi* subsp. *zooepidemicus* (SEZ) infection poses a significant threat to animals and humans, causing acute lung injury driven by excessive inflammation and tissue destruction. The gamma-aminobutyric acid type B receptor (GABA_B_R) has been implicated in modulating inflammatory responses, but its specific role in SEZ-induced acute lung injury remains unexplored. This study aimed to investigate the protective effects of pharmacological GABA_B_R activation against SEZ-triggered pulmonary damage. Employing a mouse model of SEZ infection and MH-S alveolar macrophages, we demonstrated that pretreatment with baclofen downregulated a spectrum of pro-inflammatory mediators (CXCL9, CXCL10, GM-CSF, IL-1β, IL-6, IL-17A, and MCP-1) and counteracted oxidative stress by enhancing the NRF2/HO-1 antioxidant pathway and its downstream effectors (GPX4 and SOD) in both lung tissues and macrophages. Mechanistically, baclofen exerted its effects by elevating the expression of the negative regulator interleukin-1 receptor-associated kinase 3 (IRAK3), which in turn suppressed the NLRP3/caspase-1/gasdermin D (GSDMD) inflammatory axis. Due to the suppression of inflammation and oxidative stress, the pulmonary epithelial barrier was restored in infected mice, as evidenced by the upregulation of tight junction proteins (Claudin-1, Claudin-2, Occludin, and ZO-1) in the lung. These effects collectively markedly attenuated lung histopathological injury, reduced inflammatory cell infiltration, and restored goblet cell morphology and mucin secretion. Conversely, IRAK3 deficiency exacerbated SEZ-induced lung injury, barrier disruption, oxidative stress, and NLRP3/caspase-1/GSDMD pathway activation. Collectively, our findings establish that GABA_B_R activation protects against SEZ-induced acute lung injury by enhancing barrier integrity, reprogramming macrophage antioxidant responses, and inhibiting inflammatory signaling through an IRAK3-dependent mechanism, identifying GABA_B_R as a promising therapeutic target for bacterial pneumonia.

**IMPORTANCE:**

*Streptococcus equi* subsp. *zooepidemicus* (SEZ) is a major zoonotic pathogen responsible for severe animal outbreaks and occasional life-threatening human infections, creating an urgent need for effective interventions. Gamma-aminobutyric acid type B receptor (GABA_B_R) represents a promising immunomodulatory target, as it is known to help limit inflammation during bacterial infections. In this study, we demonstrate that activating GABA_B_R pharmacologically protects against SEZ-induced acute lung injury by increasing interleukin-1 receptor-associated kinase 3 (IRAK3) expression in macrophages. This elevation of IRAK3 serves a dual function, suppressing the NLRP3/caspase-1/gasdermin D pathway while also strengthening antioxidant defenses and epithelial barrier integrity. Further support for this mechanism comes from the observation that IRAK3 deletion leads to exacerbated tissue injury. Taken together, these findings reveal a previously unrecognized GABA_B_R-IRAK3 regulatory axis that plays a central role in SEZ pathogenesis, highlighting a promising host-directed target for therapeutic development.

## INTRODUCTION

*Streptococcus equi* subsp. *zooepidemicus* (SEZ) is a significant zoonotic pathogen with a broad animal host range (1, 2). Human infections, although less frequent, are often serious and are primarily linked to the consumption of contaminated raw dairy products or direct contact with infected animals, resulting in conditions such as septicemia and respiratory infections (3, 4). Major unresolved issues persist, including the absence of a universally effective commercial vaccine and the need for a deeper understanding of the molecular determinants that govern its pathogenesis and host adaptation. Current research is actively addressing these gaps. Recent studies are employing advanced genomic techniques like multilocus sequence typing and whole-genome sequencing to track the epidemiology and evolution of emerging virulent clones (5, 6). A critical research priority is to elucidate the precise molecular pathogenesis of SEZ infection, particularly its mechanisms of host cell adhesion, invasion, and the induction of inflammatory and oxidative cascades that lead to tissue damage. Therefore, a deeper investigation into SEZ-specific pathogenesis remains essential for effectively mitigating its transmission and disease severity.

Recent research further clarifies the complex dual role of macrophages in bacterial infections, emphasizing the critical balance they maintain between effective host defense and potential immunopathology (7). Although indispensable for bacterial clearance through phagocytosis, macrophage activation is a double-edged sword, as it can lead to detrimental outcomes, including cytokine storms and impaired tissue repair (8, 9). These pathological effects are largely driven by excessive oxidative stress and dysregulated inflammasome signaling (9). A central mechanism herein is the NLRP3 inflammasome pathway (10–12). Its assembly triggers the activation of caspase-1, which then cleaves gasdermin D (GSDMD) (10, 12). This cleavage induces a form of inflammatory cell death called pyroptosis and promotes the maturation and release of potent pro-inflammatory cytokines such as IL-1β and IL-18. Oxidative stress functions in a vicious cycle within this pathway, acting as both a key trigger and a damaging consequence (13, 14). Importantly, the antioxidant NRF2/HO-1 pathway serves as a vital cellular counterbalance. Activation of the transcription factor NRF2 upregulates antioxidant enzymes like HO-1. This response helps mitigate oxidative damage and has been shown to simultaneously suppress excessive activation of the NLRP3 inflammasome and subsequent pyroptosis (15). Collectively, these findings highlight the need for precise regulation of macrophage responses and the therapeutic promise of targeting the inflammatory pathway and NRF2/HO-1 axis. However, the specific functions and regulatory mechanisms of macrophages in certain infection contexts, such as SEZ-induced acute lung injury, remain an important area for future elucidation.

Emerging research underscores the significant immunomodulatory role of the gamma-aminobutyric acid B receptor (GABA_B_R) beyond the central nervous system, particularly its influence on macrophages during inflammatory and infectious processes (16, 17). GABA_B_R activation confers recognized anti-inflammatory and antibacterial effects through specific molecular mechanisms (18, 19). Mechanistic studies indicate that its activation in immune cells dampens inflammatory responses by suppressing key pro-inflammatory pathways, including the TLR4, MyD88, and NLRP3 inflammasome axis, which is central to cytokine production such as IL-1β (20–22). Concurrently, GABA_B_R signaling enhances cellular antioxidant defenses by upregulating the NRF2/HO-1 axis, thereby mitigating the oxidative stress that contributes to tissue damage and impairs bacterial clearance. Notably, the integrity of epithelial and endothelial barriers, governed by tight junction proteins, such as claudin-1 and ZO-1, is often compromised during severe inflammation, and the potential regulation of these barrier components by GABA_B_R-modulated macrophage activity presents an intriguing avenue for research, linking innate immunity with tissue homeostasis. Recent investigations into neuro-immune crosstalk further reveal that brain GABAergic neurons can modulate peripheral macrophage activity, as exemplified by an identified brain-to-lung neural circuit where their activation promotes norepinephrine release targeting ADRB2-positive interstitial macrophages to exacerbate pulmonary inflammation (23). Despite these advances clarifying systemic and neural influences, the precise cell-intrinsic signaling mechanisms of GABA_B_R within macrophages during specific bacterial infections remain an important area for future investigation.

The interleukin-1 receptor-associated kinase 3 (IRAK3) is established as a critical negative feedback regulator in macrophages that maintains immune homeostasis by suppressing signaling through Toll-like receptor (TLR) and interleukin-1 receptor pathways to prevent excessive inflammation, primarily via inhibiting IRAK1 phosphorylation and downstream inflammasome activation (24–26). However, recent research underscores a more complex and context-dependent role for IRAK3, extending beyond mere suppression. For instance, in specific bacterial infections such as those caused by *Haemophilus influenzae*, IRAK3 can paradoxically function as a pro-inflammatory amplifier by enhancing the TLR4-JNK/NF-κB axis, thereby increasing the production of cytokines like IL-6 and TNF-α (27, 28). This dual functionality positions IRAK3 as a pivotal molecular switch that finely balances effective bacterial clearance against harmful inflammatory pathology. A direct mechanistic link demonstrating that GABA_B_R signaling modulates IRAK3 expression or activity has not been established, representing a key gap in current understanding despite parallel advances in both pathways.

We hypothesize that the GABA_B_R mitigates SEZ-induced acute lung injury in mice via modulation of the IRAK3 signaling pathway, specifically by regulating macrophage-driven inflammation and antioxidant responses. Our findings show that pharmacological GABA_B_R activation significantly attenuates SEZ-triggered pneumonia. Mechanistically, GABA_B_R activation upregulates IRAK3 expression, especially within macrophages, to simultaneously suppress the pro-inflammatory NLRP3/caspase-1/GSDMD pathway and enhance host antioxidant defenses, alongside promoting epithelial tight junction protein expression. In contrast, genetic ablation of IRAK3 exacerbates inflammatory pathology, impairs antioxidant capacity, and diminishes tight junction protein expression. Collectively, our results provide a novel theoretical framework for understanding SEZ immunopathogenesis and propose the GABA_B_R-IRAK3 axis as a promising target for innovative host-directed therapeutic strategies.

## RESULTS

### GABA_B_R activation alleviates SEZ-induced acute lung injury in mice by suppressing inflammatory factor expression

**Fig 1:**
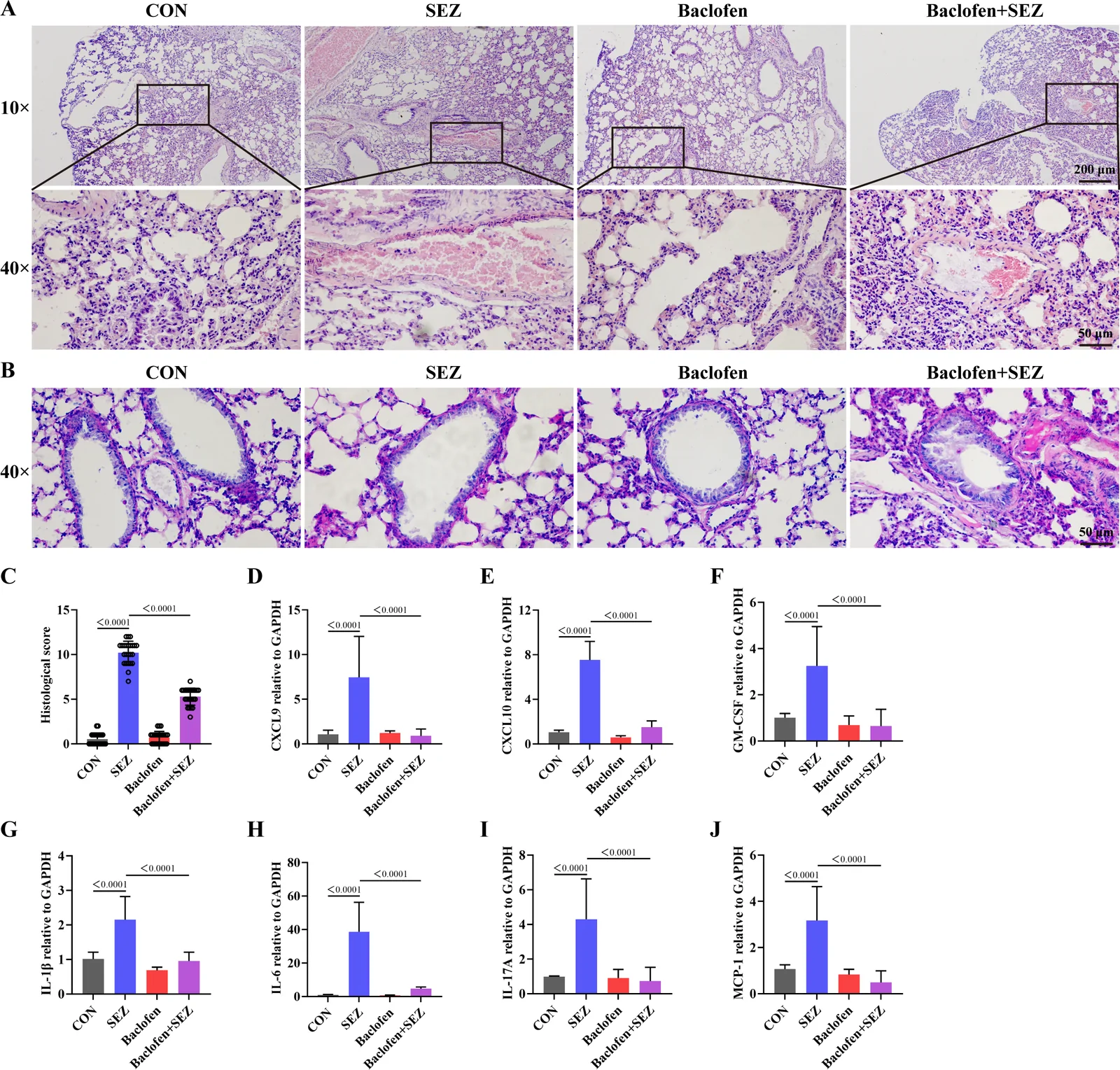
GABA_B_R activation attenuates pulmonary injury by modulating inflammatory factor expression in SEZ-induced mice. Mice received an intraperitoneal injection of 200 µL SEZ inoculum (1 × 10^8^ CFU/mL), and lung tissues were harvested 24 h later. (**A**) Representative hematoxylin and eosin (H&E)-stained images of lung tissues from different treatment groups. Magnifications: 10× (scale bar, 200 μm) and 40× (scale bar, 50 μm). (**B**) AB-PAS staining of lung sections from the different treatment groups (magnification, 40×; scale bar, 50 μm). (**C**) Histological scores based on H&E staining. (**D–J**) The mRNA expression levels of inflammatory factors in lung tissue were analyzed by quantitative real-time PCR. GAPDH was used for normalization, and the mean fold changes relative to the CON group were calculated using the 2^−ΔΔCT^ method. Data are presented as the mean ± SD (*n* ≥ 3 per group).

Building on previous evidence highlighting the anti-inflammatory role of GABA_B_R activation during bacterial infection (22), we first examined its potential to alleviate SEZ-induced pulmonary inflammation. Mice were then infected via intraperitoneal injection of 200 µL SEZ bacterial suspension (1 × 10^8^ CFU/mL). Baclofen was intraperitoneally administered starting 7 days prior to and again following SEZ infection. At 24 h post-infection, the animals were euthanized by isoflurane overdose, and lung tissues along with alveolar macrophages were harvested. Histopathological analysis of lung tissues from SEZ-infected mice showed substantial structural damage, including alveolar disruption, marked thickening of alveolar septa, extensive inflammatory cell infiltration, and evident hemorrhage, together indicating severe inflammatory injury (Fig. 1A). In contrast, pretreatment with the GABA_B_R agonist Baclofen (baclofen + SEZ group) significantly attenuated these pathological changes, leading to better-preserved tissue architecture, a notable reduction in inflammatory cell influx, and diminished hemorrhage (Fig. 1A). These findings demonstrate that pharmacological activation of GABA_B_R effectively suppresses SEZ-triggered lung inflammation. Quantitative histological scoring further supported these observations, revealing a significant increase in inflammatory scores in the SEZ group that was markedly reversed by baclofen treatment, confirming its efficacy in mitigating SEZ-induced lung pathology (Fig. 1C). Assessment of bronchial mucus secretion via Alcian blue periodic acid Schiff (AB-PAS) staining indicated impaired goblet cell function after SEZ challenge, characterized by decreased goblet cell numbers, disorganized cellular arrangement, and reduced positively stained mucin areas (Fig. 1B). Baclofen treatment effectively restored goblet cell morphology and mucin secretion capacity (Fig. 1B). At the molecular level, SEZ infection robustly upregulated a panel of inflammation-associated mediators, such as the chemokines CXCL9 and CXCL10, the cytokines GM-CSF, IL-1β, IL-6, and IL-17A, as well as monocyte chemoattractant protein-1 (MCP-1), all of which are key players in immune cell recruitment and inflammatory cascade amplification (Fig. 1D through J). Importantly, baclofen treatment significantly suppressed the expression of these inflammatory factors, indicating that the observed anti-inflammatory effect is mediated through activation of the GABA_B_R pathway.

### GABA_B_R activation strengthens pulmonary tight junctions and antioxidant defenses in SEZ-infected mice

**Fig 2:**
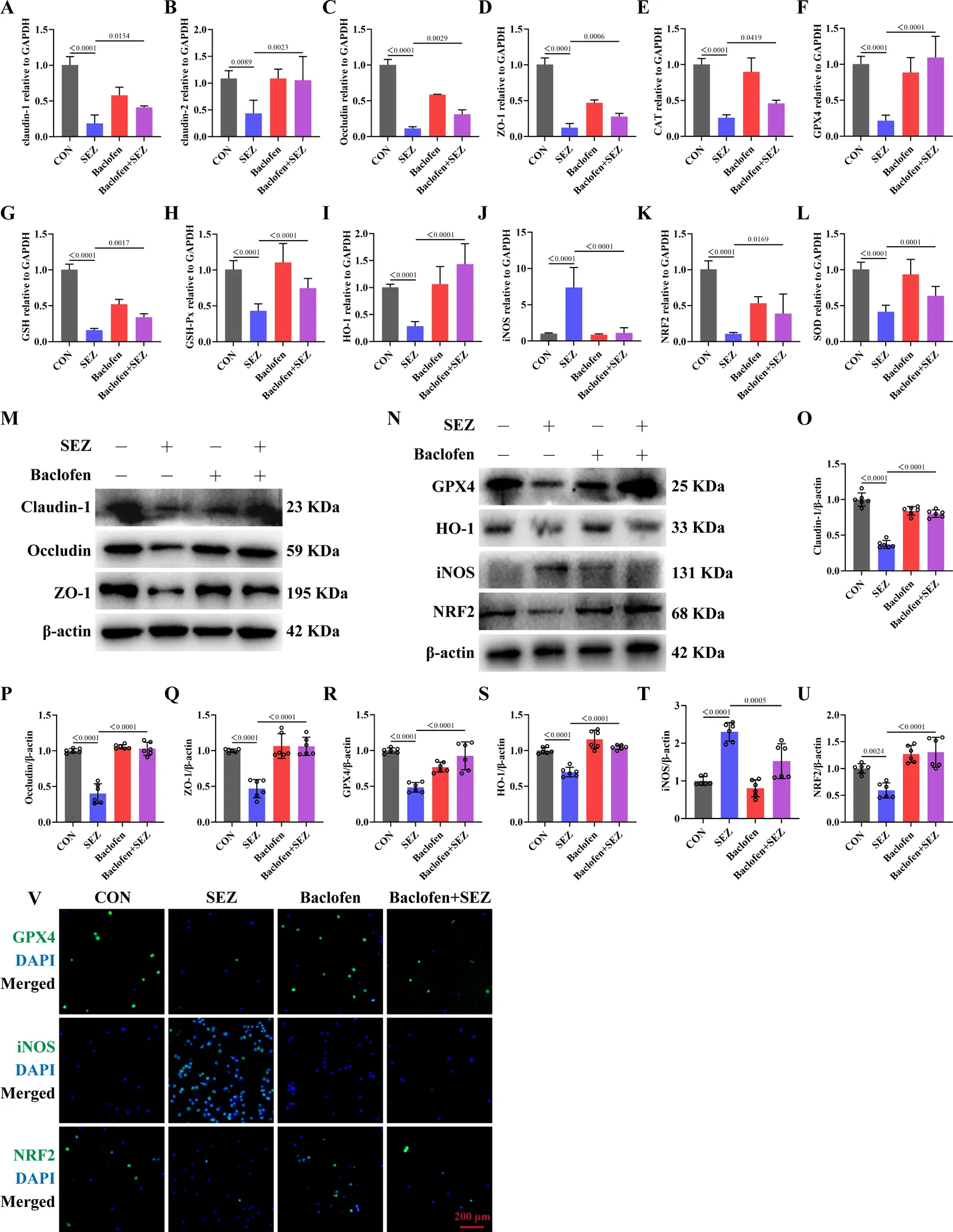
GABA_B_R activation enhances pulmonary tight junction integrity and antioxidant capacity in SEZ-induced mice. Mice received an intraperitoneal injection of 200 µL SEZ inoculum (1 × 10^8^ CFU/mL), and lung tissues along with alveolar macrophages were harvested 24 h later. (**A–D**) qRT-PCR analysis of mRNA levels for tight junction-associated molecules. (**E–L**) mRNA expression of oxidative stress-related molecules was quantified by qRT-PCR. (**M and O–Q**) Western blotting analysis of protein expression levels for the tight junction molecules Claudin-1, Occludin, and ZO-1. (**N and R–U**) Western blotting analysis was performed to determine the protein levels of the oxidative stress molecules GPX4, HO-1, iNOS, and NRF2. (**V**) Localization of oxidative stress proteins GPX4, iNOS, and NRF2 (green) in alveolar macrophages, as shown by representative immunofluorescence images. Nuclei are stained with DAPI (blue). Scale bar, 200 μm. In qRT-PCR analysis, gene expression levels were normalized to GAPDH, and relative fold changes were calculated via the 2^−ΔΔCT^ method. For Western blotting, protein expression was normalized to β-actin. Data are mean ± SD (*n* ≥ 3 per group).

Tight junctions between pulmonary epithelial cells are essential for maintaining the integrity of the lung barrier, as they orchestrate paracellular permeability and thereby ensure structural and functional homeostasis of lung tissue (29, 30). In this study, we evaluated the expression of key tight junction proteins, including Claudin-1, Claudin-2, Occludin, and ZO-1. Quantitative real-time PCR (qRT-PCR) analysis revealed that SEZ infection significantly downregulated the mRNA levels of these molecules, indicating that the pulmonary epithelial barrier was compromised (Fig. 2A through D). Pretreatment with baclofen markedly restored their expression, suggesting a barrier-protective role of the agonist (Fig. 2A through D). These findings were further corroborated at the protein level by Western blotting, which showed a pronounced reduction in Claudin-1, Occludin, and ZO-1 in SEZ-infected mice, an effect that was effectively reversed by baclofen treatment (Fig. 2M and O through Q). These results indicate that activation of GABA_B_R by baclofen alleviates SEZ-induced pulmonary epithelial barrier dysfunction by upregulating the expression of tight junction proteins.

Antioxidants are molecules that counteract harmful free radicals by scavenging and neutralizing them, thereby protecting cells from oxidative damage (31). Following SEZ infection, the expression of inducible nitric oxide synthase (iNOS), a marker of oxidative stress, was markedly upregulated (Fig. 2J). Concurrently, the expression of several key antioxidant factors was downregulated, including catalase (CAT), glutathione peroxidase 4 (GPX4), reduced glutathione (GSH), glutathione peroxidase (GSH-Px), heme oxygenase-1 (HO-1), nuclear factor erythroid 2-related factor 2 (NRF2), and superoxide dismutase (SOD), indicating impaired endogenous antioxidant defense (Fig. 2E through L). Baclofen treatment effectively reversed this imbalance, not only suppressing iNOS overexpression but also restoring the expression of the aforementioned antioxidant factors, suggesting activation of endogenous antioxidant pathways to alleviate oxidative stress (Fig. 2E through L). Consistent with transcriptional changes, Western blotting analysis showed that SEZ infection reduced protein levels of GPX4, HO-1, and NRF2 while elevating iNOS; these alterations were counteracted by baclofen, which upregulated GPX4, HO-1, and NRF2 and downregulated iNOS (Fig. 2N and R through U). These findings demonstrate that GABA_B_R activation mitigates SEZ-induced pulmonary oxidative damage by engaging the NRF2/HO-1-centered antioxidant pathway.

Macrophages play a central role in immune defense, serving not only to phagocytose and clear pathogens but also to regulate inflammation and cleanup post-infectious debris (9, 10, 25). To investigate the impact of SEZ infection and GABA_B_R activation on the redox state of macrophages, we isolated both peritoneal and alveolar macrophages and examined key antioxidant and oxidative-stress proteins by immunofluorescence. In peritoneal macrophages, SEZ infection markedly reduced the fluorescence intensity of GPX4, NRF2, and HO-1 while strongly inducing the expression of iNOS (Fig. S1A). Baclofen pretreatment effectively reversed these alterations, restoring the signals of GPX4, NRF2, and HO-1 and suppressing iNOS overexpression (Fig. S1A). An identical trend was observed in alveolar macrophages, where SEZ attenuated GPX4 and NRF2 signals and enhanced iNOS expression, whereas baclofen restored GPX4 and NRF2 levels and inhibited iNOS (Fig. 2V). Collectively, these imaging data demonstrate that SEZ infection disrupts the macrophage antioxidant defense system and exacerbates oxidative stress, whereas baclofen, by activating GABA_B_R, reprograms macrophage redox balance, thereby exerting significant antioxidative and anti-inflammatory protection in both macrophage populations.

### GABA_B_R activation directly enhances antioxidant capacity in SEZ-infected MH-S cells

**Fig 3:**
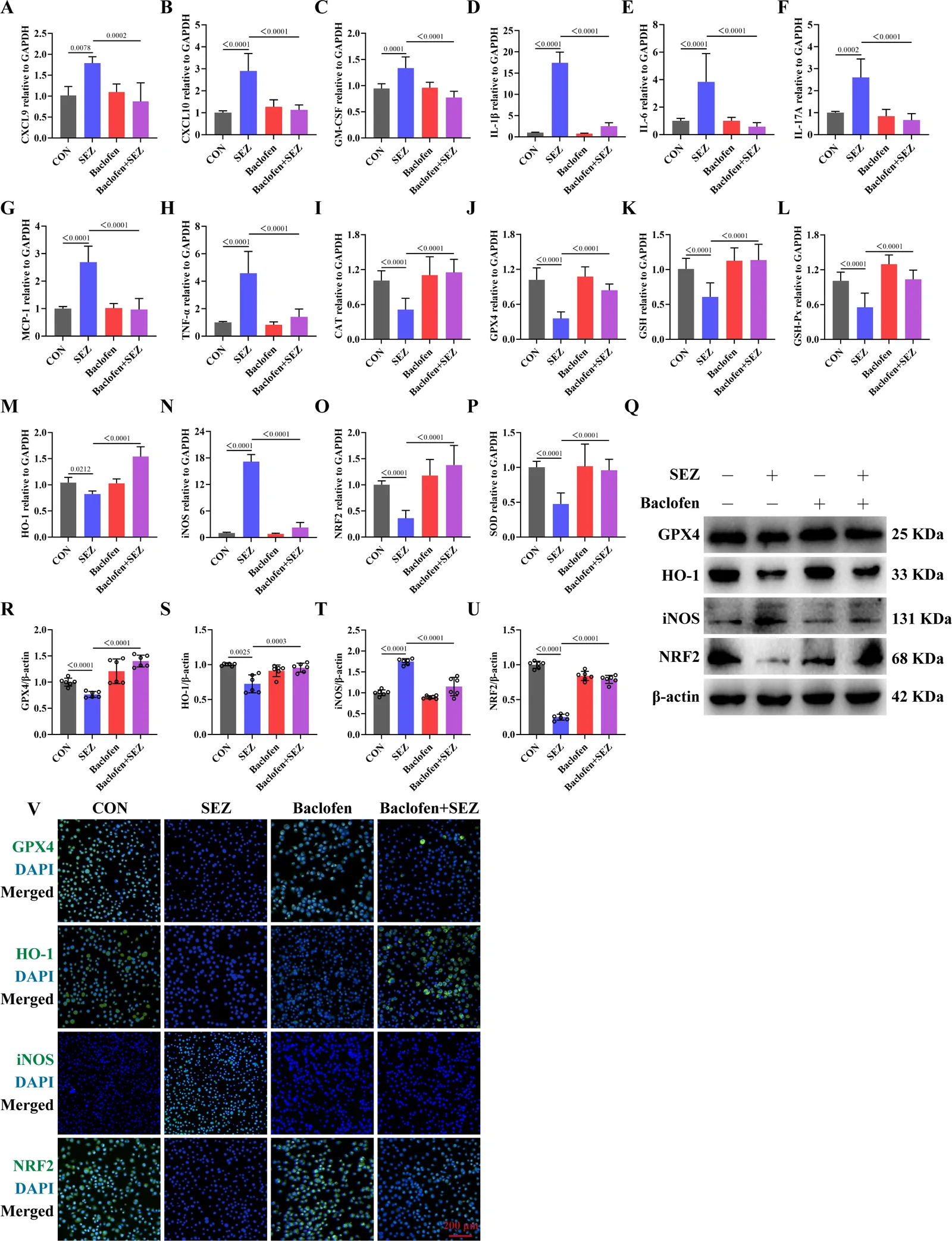
GABA_B_R activation inhibits inflammation and enhances antioxidant function in SEZ-induced MH-S cells. MH-S cells were exposed to SEZ at a multiplicity of infection (MOI) of 10 for 6 h. (**A–H**) qRT-PCR was performed to measure inflammatory factor mRNA in MH-S cells. (**I–P**) Quantification of oxidative stress-related gene expression in MH-S cells by qRT-PCR. (**Q–U**) Proteins associated with oxidative stress, including GPX4, HO-1, iNOS, and NRF2, were analyzed in MH-S cells by Western blotting. (**V**) Immunofluorescence was used to visualize oxidative stress-related molecules, including GPX4, HO-1, iNOS, and NRF2, in MH-S cells. For qRT-PCR, GAPDH was used for normalization, and relative fold changes were calculated using the 2^−ΔΔCT^ method. For Western blotting, β-actin served as the loading control. Data are presented as the mean ± SD (*n* ≥ 3 per group).

To further elucidate the mechanisms by which GABA_B_R activation modulates macrophage responses during SEZ infection, we conducted an in-depth investigation using the MH-S alveolar macrophage cell line. Cells were pretreated with 100 μM baclofen for 2 h and then infected with SEZ at a multiplicity of infection (MOI) of 10 for 6 h. qRT-PCR analysis revealed that SEZ infection significantly upregulated the mRNA expression of several pro-inflammatory mediators, including CXCL9, CXCL10, GM-CSF, IL-1β, IL-6, IL-17A, MCP-1, and TNF-α (Fig. 3A through H). In the baclofen + SEZ co-treatment group, these increases were markedly attenuated, with expression levels returning close to baseline, indicating a clear anti-inflammatory effect of GABA_B_R activation (Fig. 3A through H).

In the context of redox regulation, SEZ infection robustly suppressed the expression of key antioxidant genes, such as CAT, GPX4, GSH-Px, HO-1, NRF2, and SOD, accompanied by a significant reduction in GSH content, while concurrently upregulating iNOS (Fig. 3I through P). Baclofen treatment effectively reversed this pattern by significantly inhibiting iNOS expression and restoring the transcriptional levels of the aforementioned antioxidant factors (Fig. 3I through P). Western blotting further validated these changes at the protein level. SEZ infection reduced the abundance of GPX4, HO-1, and NRF2 and elevated iNOS, whereas co-treatment with baclofen restored GPX4, HO-1, and NRF2 to control levels and suppressed iNOS expression (Fig. 3Q through U). Immunofluorescence staining, consistent with these findings, showed diminished green fluorescence signals for GPX4, HO-1, and NRF2 alongside intense cytoplasmic iNOS staining in SEZ-infected cells (Fig. 3V). In contrast, baclofen + SEZ co-treatment markedly enhanced the fluorescence intensity of GPX4, HO-1, and NRF2 and nearly abolished the iNOS signal (Fig. 3V). Collectively, these results demonstrate that GABA_B_R activation enables MH-S macrophages to maintain a low-inflammatory, high-antioxidant phenotype upon SEZ challenge. This is achieved by concurrently suppressing inflammatory responses and enhancing antioxidant capacity, thereby providing direct cellular-level evidence for the lung-protective role of GABA_B_R signaling *in vivo*.

### GABA_B_R activation protects against lung injury by enhancing IRAK3 and inhibiting the NLRP3/caspase-1/GSDMD pathway

**Fig 4:**
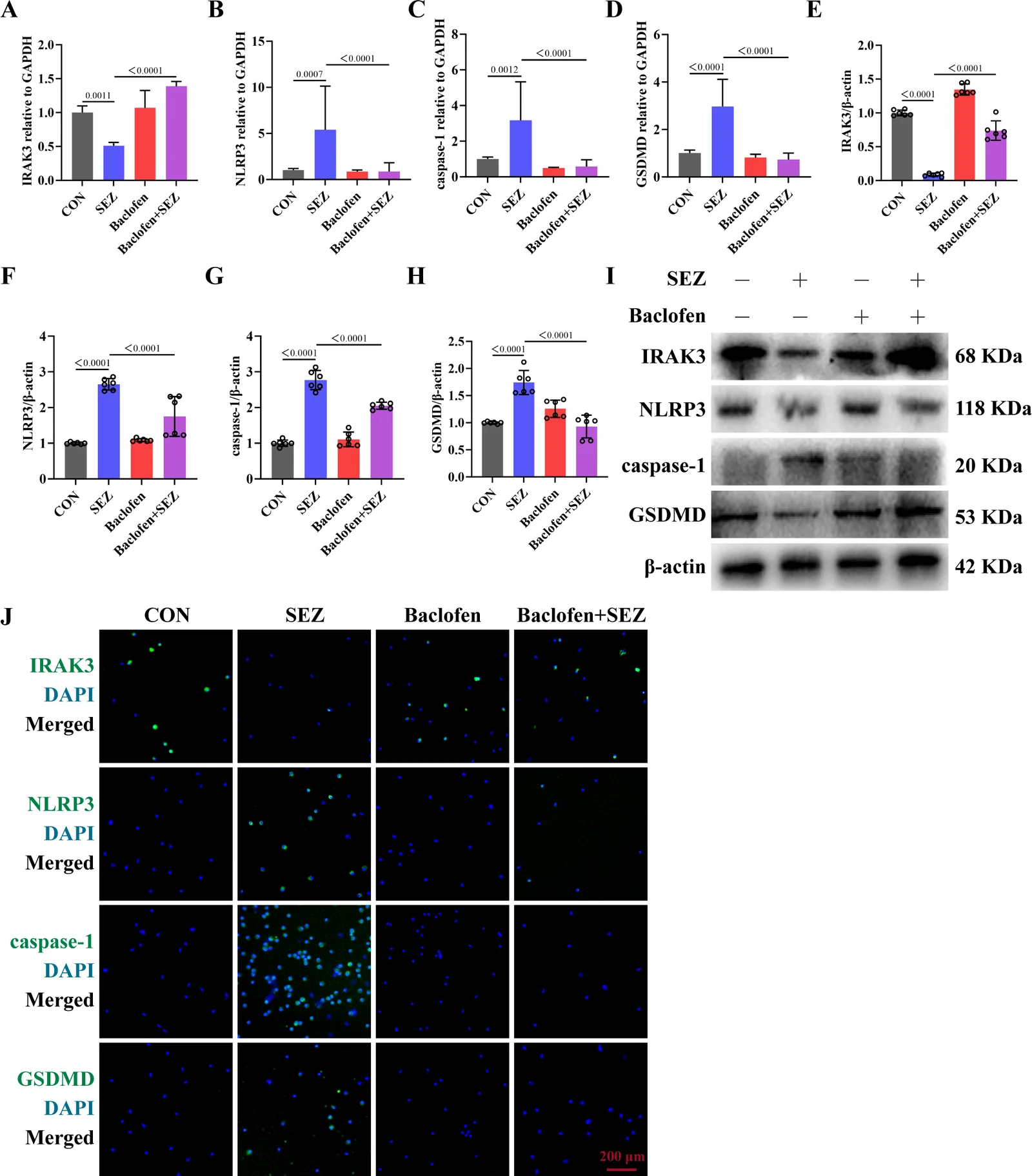
GABA_B_R activation suppresses the SEZ-activated inflammatory signaling pathway in the lungs of mice. An intraperitoneal injection of 200 µL SEZ inoculum (1 × 10^8^ CFU/mL) was administered to mice, and lung tissues along with alveolar macrophages were collected at 24 h post-injection. (**A–D**) Determination of mRNA levels of inflammatory signaling pathway-related genes in mouse lungs via qRT-PCR. (**E–I**) Western blotting was performed to detect the inflammation-related proteins IRAK3, NLRP3, caspase-1, and GSDMD in mouse lung tissue. (**J**) Localization of inflammatory proteins IRAK3, NLRP3, caspase-1, and GSDMD (green) in alveolar macrophages was visualized by immunofluorescence, with nuclei stained by DAPI (blue). Scale bar, 200 μm. For qRT-PCR, GAPDH was used for normalization, and relative fold changes were calculated using the 2^−ΔΔCT^ method. For Western blotting, β-actin served as the loading control. Data are presented as the mean ± SD (*n* ≥ 3 per group).

IRAK3 is a key negative regulator of inflammation within the interleukin-1 receptor-associated kinase family, functioning through a feedback mechanism to restrain excessive inflammatory signaling (27). Meanwhile, the NLRP3/caspase-1/GSDMD pathway serves as a canonical cascade that drives pyroptosis and promotes inflammatory cytokine release, thereby playing a central role in modulating inflammatory responses. In this study, SEZ challenge markedly downregulated IRAK3 mRNA while substantially upregulating the transcription of NLRP3, caspase-1, and GSDMD (Fig. 4A through D). Pretreatment with the GABA_B_R agonist baclofen restored IRAK3 expression to near-control levels and effectively suppressed the upregulation of NLRP3, caspase-1, and GSDMD (Fig. 4A through D). Western blotting analysis further confirmed these changes at the protein level, showing that SEZ infection reduced IRAK3 band intensity and enhanced expression of NLRP3, caspase-1, and GSDMD, whereas co-treatment with baclofen + SEZ reversed these alterations by promoting IRAK3 re-accumulation and significantly decreasing NLRP3, caspase-1, and GSDMD levels (Fig. 4E through I). Together, these findings suggest that GABA_B_R activation alleviates SEZ-induced pulmonary inflammatory injury by upregulating the negative regulator IRAK3, thereby inhibiting the NLRP3/caspase-1/GSDMD-mediated inflammatory axis.

To further delineate the regulatory mechanisms in macrophages, immunofluorescence was carried out on isolated peritoneal and alveolar macrophages. In control peritoneal macrophages, IRAK3 exhibited bright green fluorescence that colocalized with DAPI-labeled nuclei (Fig. S2A). SEZ infection strongly diminished this signal, whereas baclofen co-treatment largely restored IRAK3 fluorescence (Fig. S2A). In contrast, NLRP3, caspase-1, and GSDMD formed distinct punctate green aggregates in the cytoplasm after SEZ infection, and baclofen treatment significantly reduced both the abundance and intensity of these aggregates (Fig. S2A). These patterns were consistently observed in alveolar macrophages, where SEZ infection similarly suppressed IRAK3 fluorescence and enhanced signals for NLRP3, caspase-1, and GSDMD, all of which were markedly attenuated by baclofen (Fig. 4J). Collectively, these findings demonstrate that baclofen mitigates SEZ-driven pulmonary inflammation by reinstating the expression of the negative regulator IRAK3, thereby restraining the NLRP3/caspase-1/GSDMD pathway.

### GABA_B_R activation suppresses inflammatory signaling in SEZ-infected MH-S cells

**Fig 5:**
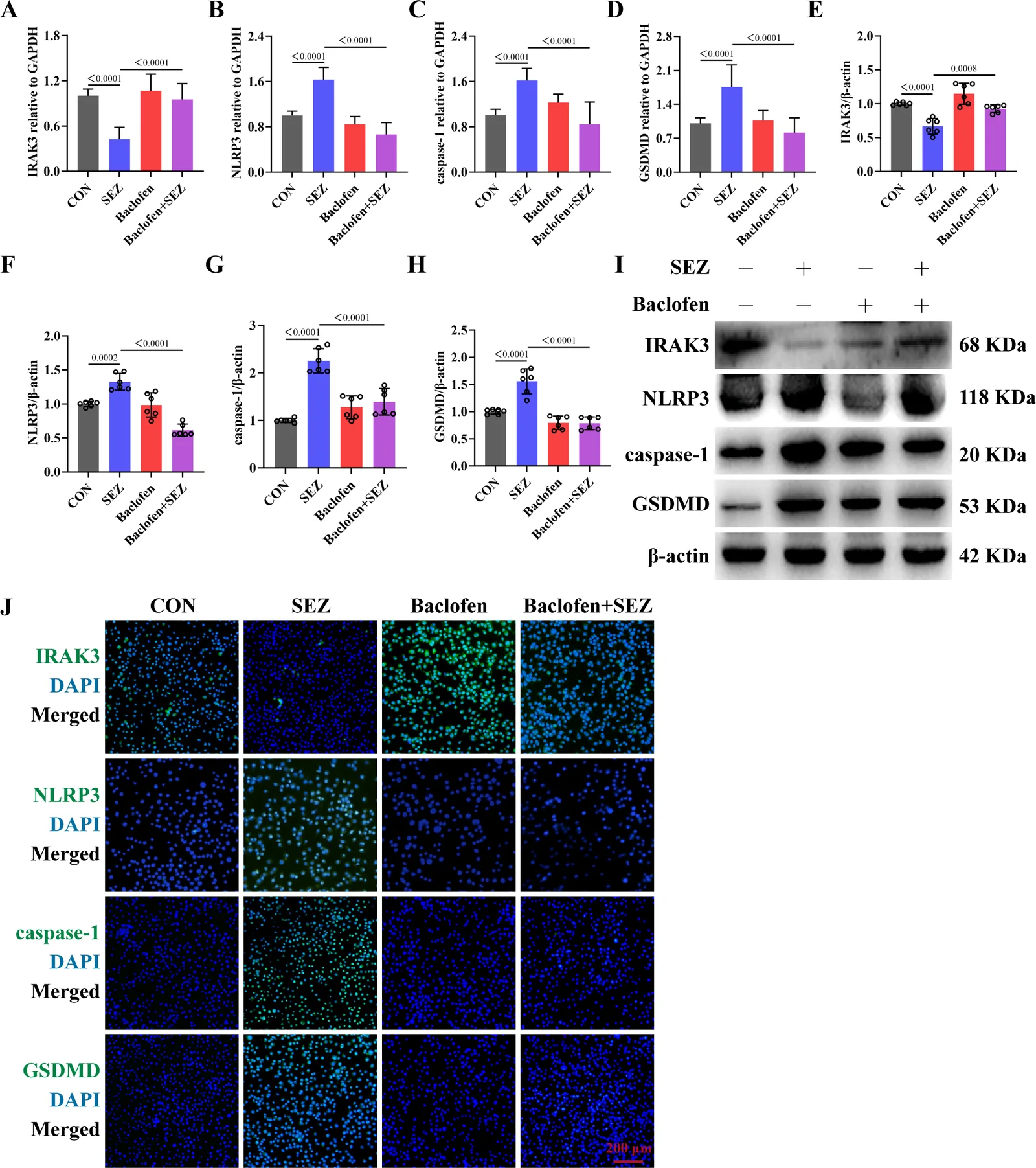
GABA_B_R activation blocks inflammatory signaling in MH-S cells following SEZ induction. MH-S cells were exposed to SEZ at a multiplicity of infection of 10 for 6 h. (**A–D**) mRNA levels of inflammatory signaling pathways in MH-S cells were quantified by qRT-PCR. (**E–I**) Western blotting analysis of inflammation-related proteins IRAK3, NLRP3, caspase-1, and GSDMD in MH-S cells. (**J**) Immunofluorescence visualization of inflammation-associated molecules IRAK3, NLRP3, caspase-1, and GSDMD in MH-S cells. For qRT-PCR, GAPDH was used for normalization, and relative fold changes were calculated using the 2^−ΔΔCT^ method. For Western blotting, β-actin served as the loading control. Data are presented as the mean ± SD (*n* ≥ 3 per group).

To further examine how GABA_B_R activation modulates inflammatory signaling, we conducted a set of experiments using the MH-S cell model. qRT-PCR analysis indicated that SEZ infection significantly downregulated the mRNA of the negative regulator IRAK3 and concurrently upregulated the transcription of NLRP3, caspase-1, and GSDMD (Fig. 5A through D). Baclofen pretreatment reversed these changes, restoring IRAK3 expression close to control levels and effectively suppressing the expression of NLRP3, caspase-1, and GSDMD (Fig. 5A through D). Western blotting confirmed this pattern at the protein level. IRAK3 band intensity was markedly weakened in the SEZ-infected group, while the expression of NLRP3, caspase-1, and GSDMD was notably increased (Fig. 5E through I). In contrast, co-treatment with baclofen and SEZ restored IRAK3 protein levels and significantly reduced the expression of NLRP3, caspase-1, and GSDMD, suggesting effective suppression of the inflammatory signaling cascade (Fig. 5E through I). Consistent results were obtained by immunofluorescence. After SEZ infection, IRAK3 showed sparse cytoplasmic fluorescence, which was replenished upon baclofen treatment (Fig. 5J). Meanwhile, fluorescence signals for NLRP3, caspase-1, and GSDMD were strongly elevated in the SEZ group and synchronously lowered in the baclofen + SEZ group (Fig. 5J). Together, these data demonstrate that baclofen reinstates the negative-feedback role of IRAK3, thereby inhibiting aberrant activation of the NLRP3/caspase-1/GSDMD pathway and effectively restraining macrophage-mediated inflammation, providing direct molecular and cellular evidence for how GABA_B_R activation alleviates SEZ-induced acute lung injury.

### IRAK3 knockout exacerbates SEZ-induced pulmonary inflammation and injury

**Fig 6:**
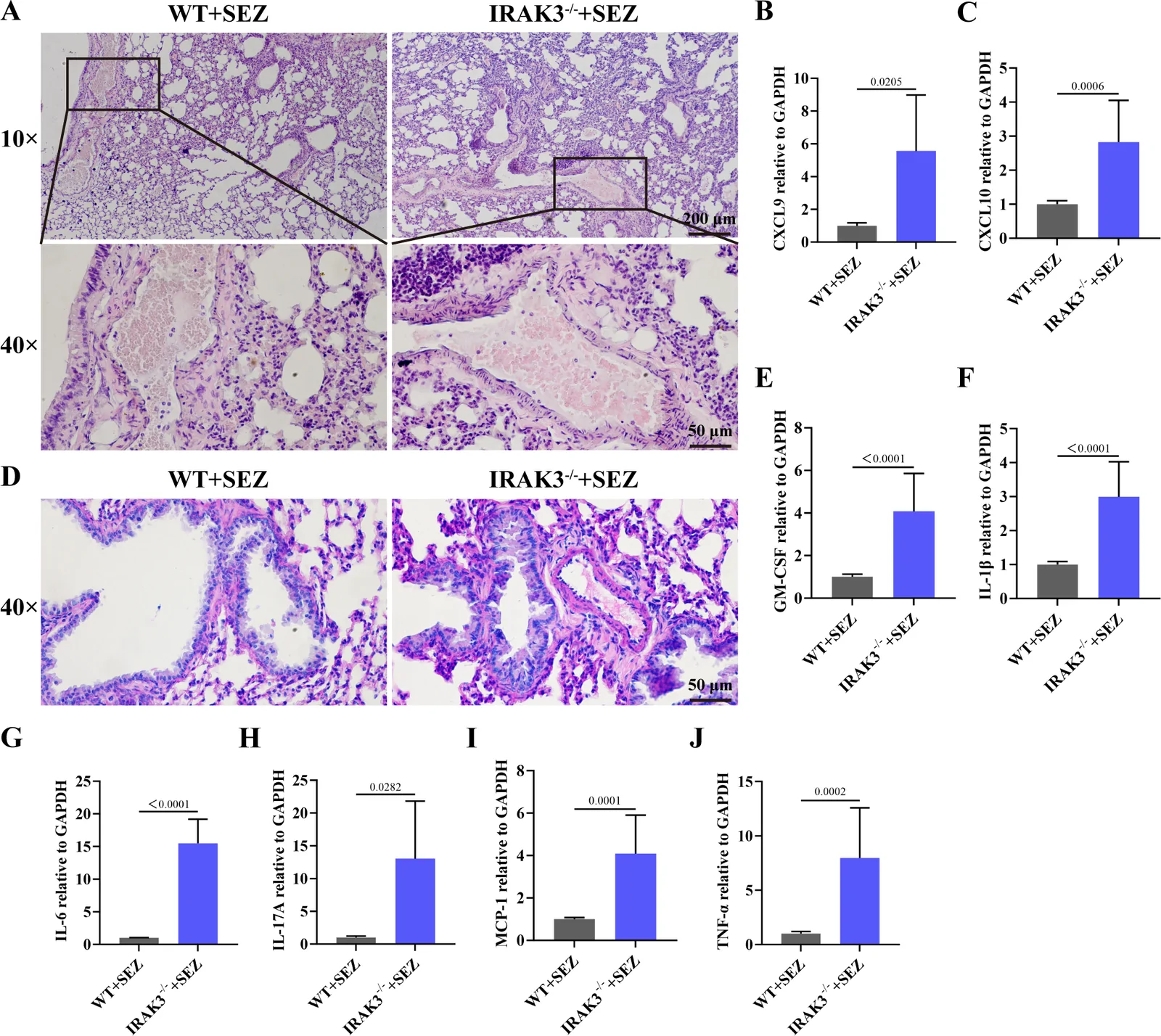
IRAK3 knockout exacerbates SEZ-induced lung injury by promoting the expression of inflammatory factors. An intraperitoneal injection of 200 µL SEZ inoculum (1 × 10^8^ CFU/mL) was administered to IRAK3^−/−^ mice, and lung tissues were collected at 24 h post-injection. (**A**) H&E staining of lung tissues from different treatment groups, showing histopathology at 10× and 40× magnifications (scale bars, 200 and 50 μm, respectively). (**B**) Quantification of histological injury scores from H&E images. (**D**) AB-PAS staining of lung sections (40×; scale bar, 50 μm) across treatment groups. (**C and E–J**) qRT-PCR quantification of inflammatory cytokine mRNA expression in lung tissue. GAPDH was used for normalization, and the mean fold changes relative to the CON group were calculated using the 2^−ΔΔCT^ method. Data are presented as the mean ± SD (*n* ≥ 3 per group).

Given the pivotal role of IRAK3 in the protective effect of baclofen against SEZ-induced pneumonia, we further examined the resulting phenotype in IRAK3^−/−^ mice. Hematoxylin and eosin (H&E)-stained lung sections (10× and 40× magnification) showed that wild-type (WT) mice infected with SEZ developed mild-to-moderate alveolar wall thickening accompanied by substantial inflammatory cell infiltration (Fig. 6A). In contrast, IRAK3^−/−^ mice exposed to SEZ exhibited aggravated pulmonary hemorrhage, extensive consolidation, and prominent neutrophil infiltration, indicating markedly worsened barrier disruption (Fig. 6A). Histopathological scoring confirmed significantly higher injury scores in the knockout group compared with WT controls (Fig. 6B). AB-PAS staining revealed disorganized goblet cell arrangement and reduced mucus secretion in WT + SEZ mice, whereas IRAK3^−/−^  +  SEZ mice displayed a sharp decline in goblet cell numbers, further disorganization, and a significant reduction in blue-purple positive areas, suggesting more severe impairment of mucociliary barrier function (Fig. 6D). qRT-PCR analysis further demonstrated that, in the absence of IRAK3, SEZ infection induced a more pronounced upregulation of inflammatory mediators such as CXCL9, CXCL10, GM-CSF, IL-1β, IL-6, IL-17A, MCP-1, and TNF-α compared with WT mice, reflecting an amplified cytokine response (Fig. 6C and E through K). Taken together, these results indicate that IRAK3 deficiency disrupts the negative feedback regulation of inflammation, exacerbates pulmonary structural damage and goblet cell dysfunction, and globally elevates the expression of pro-inflammatory mediators, thereby significantly aggravating SEZ-induced acute lung injury.

### Loss of IRAK3 impairs tight junction integrity and antioxidant responses

**Fig 7:**
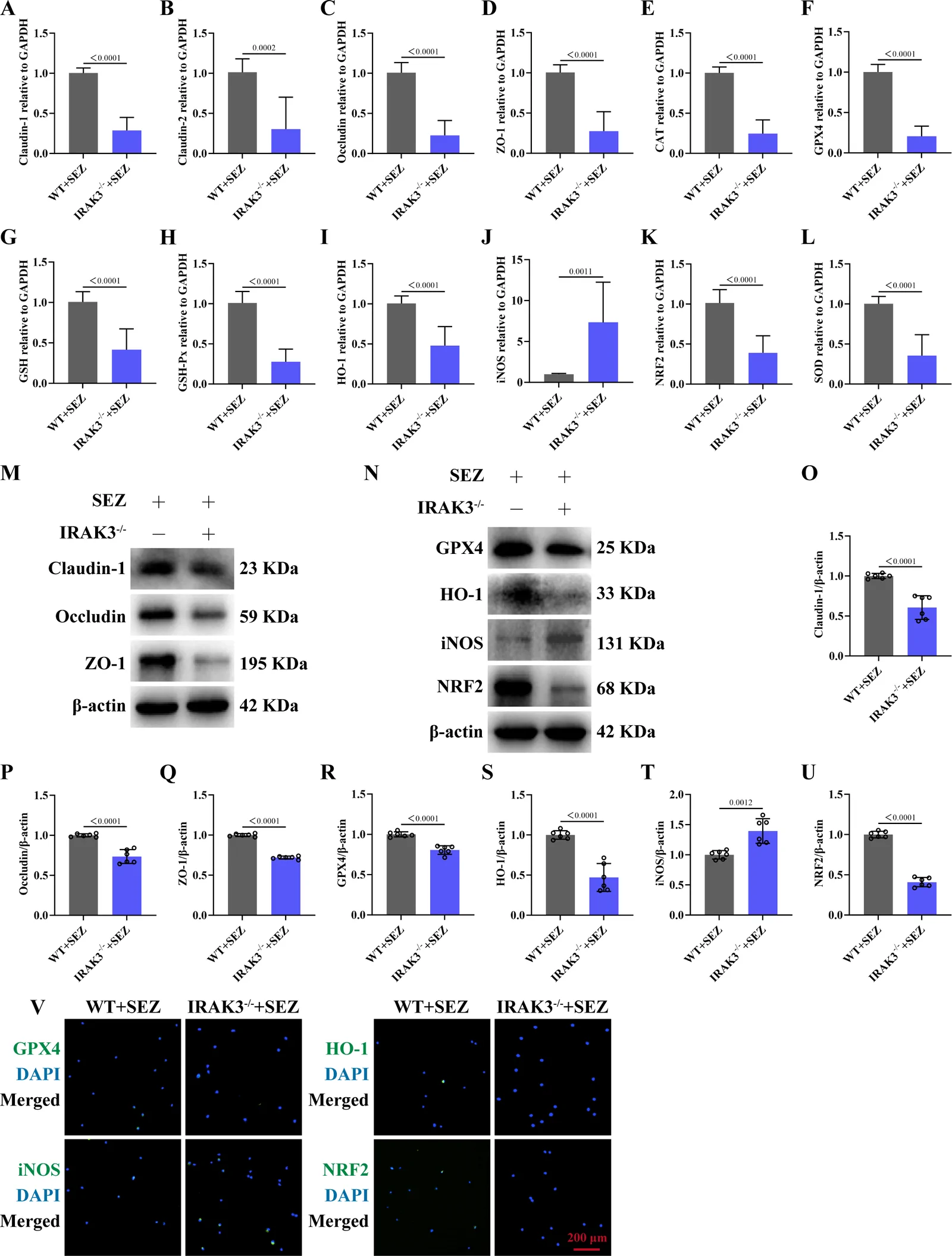
IRAK3 knockout impairs pulmonary tight junction integrity and antioxidant capacity in SEZ-infected mice. IRAK3^−/−^ mice received an intraperitoneal injection of 200 µL SEZ inoculum (1 × 10^8^ CFU/mL), and lung tissues along with alveolar macrophages were harvested 24 h later. (**A–D**) mRNA levels of tight junction-associated molecules were measured via qRT-PCR. (**E–L**) Oxidative stress-related gene expression was quantified using qRT-PCR. (**M and O–Q**) The protein expression of tight junction markers Claudin-1, Occludin, and ZO-1 was examined by Western blotting. (**N and R–U**) Western blotting analysis determined the expression levels of oxidative stress-related proteins GPX4, HO-1, iNOS, and NRF2. (**V**) Immunofluorescence detection of oxidative stress proteins GPX4, HO-1, iNOS, and NRF2 (green) in alveolar macrophages, respectively. Nuclei were counterstained with DAPI (blue). Scale bar, 200 μm. In qRT-PCR analysis, gene expression levels were normalized to GAPDH, and relative fold changes were calculated via the 2^−ΔΔCT^ method. For Western blotting, protein expression was normalized to β-actin. Data are mean ± SD (*n* ≥ 3 per group).

We next examined the effects of IRAK3 knockout on pulmonary tight junction integrity and oxidative stress in mice. qRT-PCR analysis showed that mRNA levels of the tight-junction proteins, Claudin-1, Claudin-2, Occludin, and ZO-1, were significantly reduced in WT + SEZ mice compared with controls, and this downregulation was further exacerbated in IRAK3^−/−^ + SEZ mice, where all four transcripts were markedly lower (Fig. 7A through D). Western blotting corroborated these findings at the protein level, with Claudin-1, Occludin, and ZO-1 exhibiting the weakest band intensity in the IRAK3^−/−^ + SEZ group relative to the WT + SEZ group, indicating that the expression of these barrier-related genes depends on IRAK3 (Fig. 7M and O through Q).

Moreover, a similar pattern was observed in the antioxidant system. In WT + SEZ mice, the mRNA expression of antioxidant genes, such as CAT, GPX4, GSH, GSH-Px, HO-1, SOD, and NRF2, was suppressed, while iNOS expression was elevated (Fig. 7E through L). IRAK3 deficiency led to a further decline in these antioxidant transcripts and an additional rise in iNOS. Consistent with the transcriptional changes, Western blotting revealed corresponding decreases in the protein levels of GPX4, HO-1, and NRF2, together with a significant increase in iNOS (Fig. 7N and R through U). Immunofluorescence analysis provided visual confirmation at the cellular level. In both peritoneal (Fig. S3A) and alveolar (Fig. 7V) macrophages, the WT + SEZ group displayed weakened green fluorescence for GPX4, HO-1, and NRF2, alongside an enhanced green signal for iNOS. Upon IRAK3 knockout, fluorescence signals for GPX4, HO-1, and NRF2 became nearly undetectable, while iNOS green fluorescence became diffusely distributed throughout the cytoplasm. These data indicated that IRAK3 acts as a critical molecular node through which GABA_B_R activation maintains tight junction integrity and activates the antioxidant program; loss of IRAK3 disrupts the dual protective mechanism of barrier function and antioxidant defense in macrophages, thereby aggravating SEZ-induced lung injury.

### IRAK3 deficiency leads to hyperactivation of the NLRP3/caspase-1/GSDMD pathway

**Fig 8:**
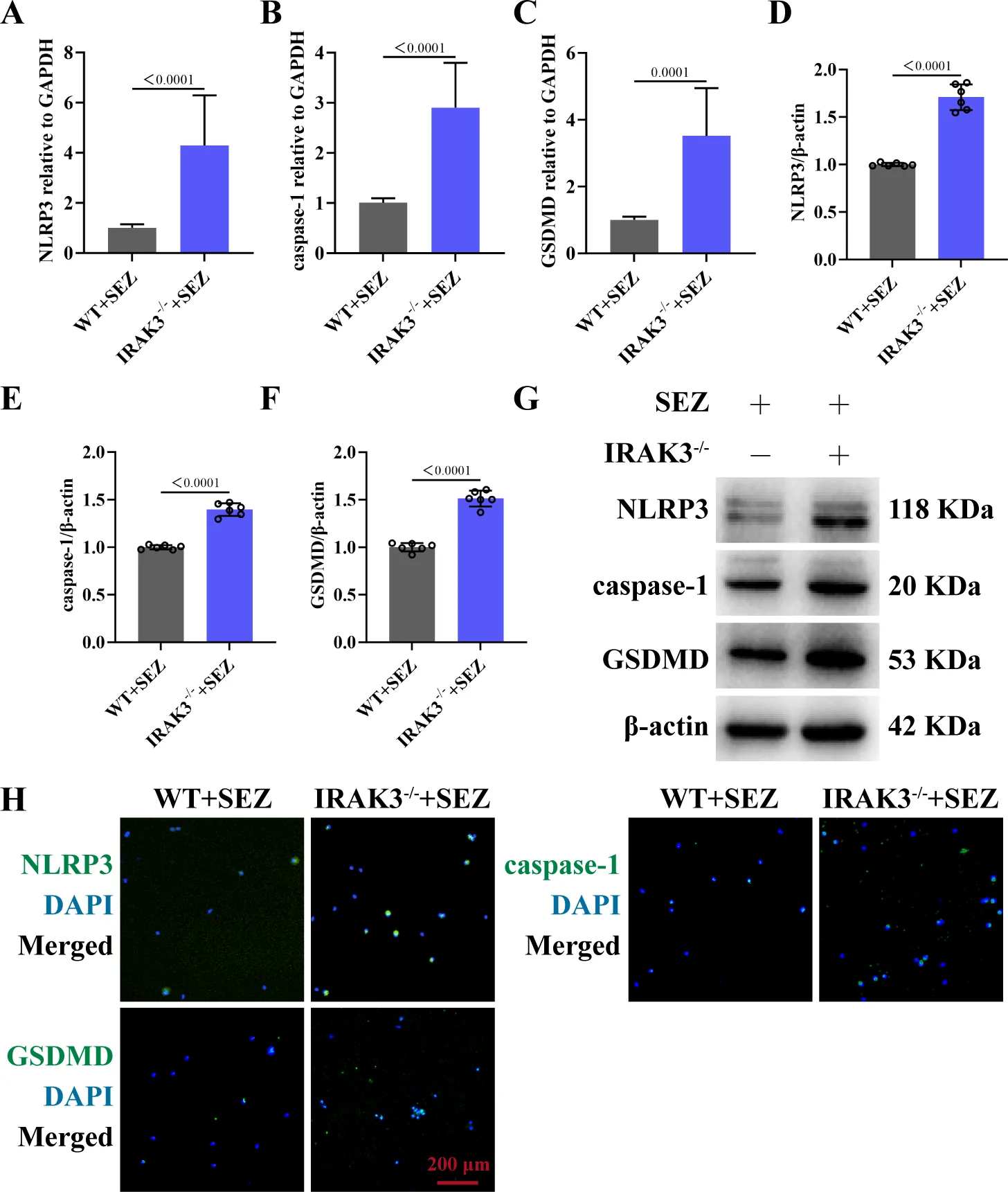
IRAK3 knockout potentiates inflammatory signaling in the lungs of SEZ-infected mice. An intraperitoneal injection of 200 µL SEZ inoculum (1 × 10^8^ CFU/mL) was administered to IRAK3^−/−^ mice, and lung tissues along with alveolar macrophages were collected at 24 h post-injection. (**A–C**) qRT-PCR analysis of mRNA levels for inflammatory signaling pathway-related genes in mouse lungs. (**D–G**) Expression of the inflammation-related proteins NLRP3, caspase-1, and GSDMD in mouse lung tissue was examined by Western blotting. (**H**) In alveolar macrophages, the localization of inflammatory proteins NLRP3, caspase-1, and GSDMD (green) was assessed using immunofluorescence. Nuclei were stained with DAPI (blue). Scale bar, 200 μm. For qRT-PCR, GAPDH was used for normalization, and relative fold changes were calculated using the 2^−ΔΔCT^ method. For Western blotting, β-actin served as the loading control. Data are presented as the mean ± SD (*n* ≥ 3 per group).

To further examine the impact of IRAK3 knockout on the NLRP3/caspase-1/GSDMD signaling pathway, we performed qRT-PCR, Western blotting, and immunofluorescence analyses. qRT-PCR results showed that the transcriptional levels of NLRP3, caspase-1, and GSDMD were already significantly elevated in WT + SEZ mice, and this upregulation became even more pronounced in IRAK3^−/−^ + SEZ mice, indicating a further derepression of inflammatory gene expression upon loss of IRAK3 (Fig. 8A through C). Western blotting corroborated this trend at the protein level, revealing that the expression of NLRP3, caspase-1, and GSDMD was further increased in IRAK3^−/−^ + SEZ mice compared with the WT + SEZ group, suggesting that activation of this inflammatory cascade is exacerbated due to the absence of IRAK3-mediated negative feedback (Fig. 8D through G). Immunofluorescence observations were consistent with these findings. In both peritoneal (Fig. S4A) and alveolar (Fig. 8H) macrophages, distinct green fluorescent puncta corresponding to NLRP3, caspase-1, and GSDMD were readily visible in the WT + SEZ group, and these signals were further intensified in the IRAK3^−/−^ + SEZ group. Together, these results demonstrate that IRAK3 deficiency leads to dysregulated inflammatory signaling in macrophages, providing direct molecular evidence that GABA_B_R activation alleviates SEZ-induced acute lung injury by restoring IRAK3 expression.

## DISCUSSION

SEZ is a formidable zoonotic pathogen, causing severe outbreaks in animals and sporadic, life-threatening infections in humans, in which acute lung injury is a prominent manifestation. The urgent need for effective therapeutic strategies is thereby underscored. Acute lung injury is characterized principally by a dysregulated inflammatory response, and GABA_B_R is posited as a promising immunomodulatory target, given its demonstrated ability to attenuate inflammation during bacterial infection. This study provides direct evidence that pharmacological GABA_B_R activation protects against SEZ-induced pneumonia by upregulating IRAK3 in macrophages. Elevated IRAK3 dually inhibits the NLRP3/caspase-1/GSDMD pathway and enhances antioxidant and epithelial barrier defenses, a mechanism corroborated by the exacerbated injury observed upon IRAK3 deletion. Collectively, these results delineate a previously unrecognized GABA_B_R-IRAK3 regulatory axis central to SEZ pathogenesis, revealing a promising host-directed target for therapeutic development.

The primary focus of this study is not the local alveolar infectious process *per se*, but rather the acute lung injury secondarily induced by the systemic inflammatory response following SEZ infection. To this end, an SEZ infection model was established in our laboratory via intraperitoneal injection, and viable bacteria were successfully re-isolated from the lungs of challenged mice. This finding validates that following intraperitoneal administration, SEZ disseminates hematogenously to the lung, where it upregulates the NLRP3/caspase-1/GSDMD axis, thereby triggering acute lung injury (32). Based on this evidence, the intraperitoneal route of SEZ administration was deliberately selected for the induction of acute lung injury in the present study. Demonstrating that pharmacological activation of the GABA_B_R with baclofen significantly attenuates SEZ-induced acute lung injury, the present study provides evidence, including preserved lung architecture, reduced inflammatory cell infiltration, restored goblet cell function, and the suppression of a broad panel of pro-inflammatory cytokines and chemokines such as IL-1β, IL-6, IL-17A, GM-CSF, CXCL9, CXCL10, and MCP-1. These findings robustly align with and extend the growing body of evidence supporting the potent immunomodulatory role of peripheral GABA_B_R signaling (20). Observed downregulation of key cytokines, including IL-1β and IL-6, is consistent with documented anti-inflammatory effects of baclofen in diverse models such as lipopolysaccharide (LPS)-induced enteritis and collagen-induced arthritis (20, 21). Mechanistically, the broad suppression of inflammatory mediators, particularly IL-1β, strongly implies an intervention in the innate immune signaling cascade upstream of cytokine production. Given that the TLR4/NLRP3 axis is a central hub for sensing pyogenic bacteria like SEZ (32), it is highly plausible that a similar mechanism, namely the suppression of the NLRP3 inflammasome and subsequent IL-1β maturation, underlies the protective effects observed in this pulmonary model. The work also resonates with the latest frontiers in neuro-immunology, particularly regarding the context-dependent outcomes of GABAergic signaling (33, 34). Direct support comes from previous studies, which demonstrated that baclofen alleviates intestinal inflammation by inhibiting the TLR4/NF-κB/NLRP3 inflammasome pathway (20–22). These observations indicate that GABA_B_R activation exerts its anti-inflammatory effects principally via the TLR4/NF-κB/NLRP3 signaling cascade. Nevertheless, the current work was confined to the IRAK3/NLRP3 inflammatory axis, leaving the upstream NF-κB pathway unexplored. Accordingly, further studies are planned to clarify the functional contribution of NF-κB signaling to the protective effects elicited by GABA_B_R activation in the context of SEZ-induced acute lung injury. While a direct protective effect of peripheral GABA_B_R activation on lung immune cells is demonstrated here, previous studies have revealed a contrasting, pro-inflammatory pathway where central GABAergic neurons in the brain can be activated during severe pneumonia to amplify lung inflammation via a sympathetic neural circuit targeting ADRB2^+^ interstitial macrophages (23). This contrast does not invalidate the present findings but rather highlights the exquisite complexity and compartmentalization of neuro-immune communication, suggesting that the final inflammatory outcome may depend on the specific site, cellular target, and receptor subtype involved in GABAergic signaling. Employing systemic baclofen administration, the study likely highlights the net beneficial effect of directly targeting GABA_B_R on lung-resident and infiltrating immune cells, thereby outweighing or bypassing potential pro-inflammatory neural pathways.

Findings from this study indicate that activation of GABA_B_R with baclofen provides comprehensive protection against SEZ-induced acute lung injury, achieved through a multi-tiered mechanism. Specifically, baclofen preserves the pulmonary epithelial barrier, activates systemic and cellular antioxidant defenses, and functionally reprograms macrophages. A key observation is the restoration of critical tight junction protein expression, such as Claudin-1, Occludin, and ZO-1, by baclofen. This outcome underscores a novel function for GABA_B_R signaling in upholding lung architectural integrity. Essential roles of these proteins, especially Claudin-1 and Occludin, in forming the paracellular seal are widely accepted, and their disruption constitutes a recognized hallmark of barrier failure across various lung pathologies (35–37). Although direct evidence connecting GABA_B_R to pulmonary tight junction regulation was previously scarce, strong support for its mechanistic plausibility now comes from prior research. That work showed baclofen, acting via GABA_B_R, alleviates LPS-induced intestinal inflammation by inhibiting the TLR4/NF-κB/NLRP3 pathway while simultaneously enhancing the NRF2/HO-1 antioxidant axis (20, 21). An identical molecular signature emerges, namely suppression of a central pro-inflammatory cascade paired with potentiation of the master antioxidant regulator. Such a parallel offers a compelling and credible mechanistic blueprint applicable to current findings in the lung. Downregulation of TLR4, NF-κB, and NLRP3 signaling constitutes a plausible upstream event that could account for reduced inflammatory damage to the tight junction complex observed in the SEZ model. Furthermore, potent activation of the NRF2/HO-1 pathway and consequent upregulation of antioxidant enzymes, including SOD, GSH, GPX4, and CAT, represent a fundamental, defense-oriented response. Precise alignment exists with the established role of NRF2 as a critical coordinator of cellular antioxidant defenses. Restoration of this pathway by baclofen directly counteracts the SEZ-induced oxidative burst, characterized by iNOS overexpression, thereby mitigating a principal driver of tissue damage. Extending this paradigm, data show that GABA_B_R-mediated antioxidant reprogramming is executed precisely within key immune sentinels. Following intraperitoneal injection, SEZ proliferates extensively in the peritoneal cavity, breaches the peritoneal barrier, enters the bloodstream, and disseminates to the lungs (32), resulting in acute lung injury. In the present study, peritoneal macrophages were initially isolated and examined, confirming successful SEZ infection and the associated inflammatory response in the peritoneal cavity. The predominant confinement of peritoneal macrophages to the local peritoneal environment limits their utility in demonstrating SEZ-induced acute lung injury; alveolar macrophages were subsequently harvested for further verification. These findings suggest that the antioxidant activity is predominantly localized to peritoneal and alveolar macrophages, and not merely systemically.

Results presented in the current investigation indicate that activation of GABA_B_R with baclofen protects against SEZ-induced acute lung injury. Protection is achieved through a mechanism involving upregulation of the negative regulator IRAK3 to suppress signaling along the NLRP3/caspase-1/and GSDMD pathway, representing a significant advancement in mechanistic understanding. Such a conclusion is firmly grounded in and extends contemporary literature, which validates both the therapeutic target and the identified regulatory axis (38–40). Crucially, work progresses beyond phenotypic confirmation to delineate a precise and novel intracellular signaling cascade centered on IRAK3. Critical phenotypic observations from the current work reveal that IRAK3 knockout mice exhibited markedly exacerbated SEZ-induced lung injury. Data presented show that loss of IRAK3 leads to catastrophic failure of all protective mechanisms. This failure includes severe barrier disruption, complete collapse of the NRF2-centered antioxidant program, and hyperactivation of the NLRP3/caspase-1/GSDMD inflammatory pathway. Such conclusions are powerfully supported by recent and compelling research that not only corroborates the mechanistic links but also provides a nuanced framework for understanding the complex, context-dependent role of IRAK3 in pulmonary immunity (24, 26, 27, 38). Exacerbated injury in IRAK3 knockout mice confirms that without this regulatory brake, a protective inflammatory response becomes a primary driver of lethal immunopathology. Although previous studies have suggested that GABA_B_R can modulate inflammatory responses through IRAK3, the present study did not perform agonist intervention experiments in IRAK3 knockout mice. Therefore, whether GABA_B_R exerts anti-inflammatory and antioxidant effects in SEZ-induced secondary lung injury in an IRAK3-dependent manner remains to be further elucidated. To address this issue, future studies are planned to combine GABA_B_R agonist treatment in the IRAK3-deficient background, thereby directly verifying the functional relevance of this signaling pathway. In addition, although bacterial burden was not quantified in this study, the dissociation between anti-inflammatory effects and barrier function protection in the absence of antibiotic treatment suggests that GABA_B_R activation primarily exerts protective effects through modulation of host immune responses rather than through direct antibacterial activity. Nevertheless, the potential contribution of bacterial clearance to the amelioration of acute lung injury cannot be completely excluded. Subsequent experiments will include quantitative assessment of bacterial load and *in vitro* bactericidal assays to further determine whether the protective effects of baclofen are indeed independent of its possible antibacterial actions. Data presented here robustly confirm a foundational concept within a distinct, bacterially driven model of acute lung injury, reaffirming the broad relevance of GABA_B_R as a host-directed immunomodulatory target in lung tissue.

### Conclusions

Findings from this work establish that GABA_B_R activation with baclofen confers robust protection against SEZ-induced pneumonia. Protection is mediated via upregulation of IRAK3, a central negative regulator. Acting through this node, baclofen coordinately suppresses the NLRP3/caspase-1/GSDMD inflammatory cascade, potentiates the NRF2/HO-1 antioxidant pathway, and reinstates pulmonary barrier integrity through tight junction proteins. Genetic ablation of IRAK3 conclusively abolishes this protection and aggravates disease pathology. Collectively, this work defines the GABA_B_R-IRAK3 axis as a master regulatory hub that integrates multiple host defense programs, thereby revealing a novel host-directed therapeutic strategy for severe bacterial pneumonia.

## MATERIALS AND METHODS

### Animals

C57BL/6 wild-type male mice aged 6–8 weeks were acquired from Guangdong Yaokang Biotechnology Co., Ltd. (Guangdong, China). IRAK3 knockout mice on a C57BL/6 background were sourced from The Jackson Laboratory (007016; PA, USA). Animals were housed under specific pathogen-free conditions, with temperature maintained at 20°C ± 3°C, humidity at 60% ± 5%, and *ad libitum* access to food and water. Every effort was made to alleviate discomfort and to employ the minimum number of animals required.

### Bacterial strains and cell line

The SEZ strain C55138 was sourced from the China Institute of Veterinary Drug Control (Beijing, China). Bacterial cultures were maintained at 37°C using tryptose soya agar (Oxoid, Basingstoke, UK) and tryptone soybean broth (Oxoid) enriched with 5% newborn bovine serum (HyClone, Logan, UT, USA). These cultures were then processed by centrifugation at 3,500 × *g* for 10 min, followed by PBS washing and serial dilution in preparation for downstream experiments (41, 42).

The murine alveolar macrophage cell line MH-S (CRL-2019; ATCC, Manassas, USA) was maintained in RPMI-1640 (SH30809.01; HyClone) medium supplemented with 10% fetal bovine serum (SH30406.05; HyClone), 0.05 mM β-mercaptoethanol, and 1% penicillin–streptomycin solution (SV30010; HyClone) and incubated at 37°C in a 5% CO_2_ atmosphere.

### Mouse infection

Mice were infected via intraperitoneal injection of 200 µL SEZ bacterial suspension (1 × 10^8^ CFU/mL) (41, 42). Twenty-four hours post-infection, the mice were euthanized by isoflurane overdose. To activate GABA_B_R, the agonist baclofen (10 mg/kg of body weight; HY-B0007, MCE, NJ, USA) was administered intraperitoneally, beginning 7 days prior to SEZ infection and again following infection (43). Upon harvest, lung tissues were divided: one portion was fixed in 4% paraformaldehyde (PFA), and the remainder was stored at −80°C for subsequent analysis.

### MH-S infection assay

MH-S cells were infected with SEZ using an established protocol (42). Upon reaching approximately 80% confluence, cells were seeded into 48- or 6-well plates and then exposed to SEZ at an MOI of 10 for 6 h. To activate GABA_B_R, cells were pretreated with 100 μM baclofen for 2 h prior to infection (43). Following the infection period, cells were either collected or fixed with ice-cold paraformaldehyde for subsequent analysis.

### Isolation of primary macrophages from the peritoneal cavity and alveoli

Peritoneal macrophages and alveolar macrophages were isolated from mice using established lavage protocols (32, 44). Peritoneal macrophages were isolated from SEZ-infected mice by intraperitoneal lavage. Briefly, after euthanasia, 5 mL of sterile PBS was injected into the peritoneal cavity, followed by gentle abdominal massage for 3 min, and subsequent aspiration of the lavage fluid. For alveolar macrophages, bronchoalveolar lavage was performed by cannulating the trachea and washing the lungs three to five times with ice-cold sterile PBS (1 mL per wash). Cells from both sources were collected by centrifugation at 200 × *g* for 10 min, processed at 4°C, resuspended in DMEM supplemented with 10% fetal bovine serum and 1% penicillin–streptomycin, and seeded into 48-well plates. After incubation for 2 h at 37°C in a humidified 5% CO_2_ atmosphere, nonadherent cells were removed by washing three times with PBS, and fresh medium was added. The adherent cells were defined as peritoneal macrophages or alveolar macrophages, respectively.

### Hematoxylin and eosin

Lung tissues were processed for histopathological evaluation using a standard protocol (22, 32). Briefly, samples were fixed in 4% PFA for 48 h, dehydrated through an ethanol gradient, cleared in xylene, and embedded in paraffin. Sections of 8 μm thickness were then cut, mounted on slides, and stained with H&E. Tissue morphology was assessed under a Nikon Ni-U light microscope (Tokyo, Japan) at 10× and 40× magnifications.

For histopathological scoring, lung sections from each group of mice were examined under 40× magnification; each dimension was scored from 0 (normal) to 4 (severe) according to the criteria in Table 1.

**TABLE 1:** Scoring dimensions and criteria

Dimension	Description	Score range
Inflammatory cell infiltration	Evaluates the type, quantity, and distribution of inflammatory cells in the lung interstitium and alveolar spaces.	0–4 points
Nature of alveolar exudate	Assesses the type (serous, fibrinous, purulent, etc.) and extent of exudate within alveolar spaces.	0–4 points
Alveolar structural damage and consolidation	Evaluates the degree of alveolar wall thickening, collapse, fusion, consolidation, and widening of the interstitium.	0–4 points
Neutrophil aggregation and abscess formation	Assesses the accumulation of neutrophils in alveolar spaces, bronchioles, and lung tissue and the presence of abscesses or necrosis.	0–4 points

### Alcian blue periodic acid Schiff

Sections of 8 μm thickness were deparaffinized, rehydrated, and stained with Alcian blue solution (pH 2.5) for acidic mucins. After rinsing, the sections were oxidized in 1% periodic acid and subsequently treated with Schiff’s reagent to visualize neutral mucins and glycoproteins. Nuclei were lightly counterstained with hematoxylin. Finally, sections were dehydrated, cleared in xylene, and mounted with a neutral resin. Stained sections were examined under a Nikon Ni-U light microscope (Tokyo, Japan) at 40× magnification.

### RNA isolation and quantitative real-time PCR

Total RNA was extracted from mouse lung tissues and MH-S cells using the RNA simple Total RNA Kit (TIANGEN, Beijing, China). The isolated RNA was then reverse transcribed into cDNA with the HiScript II 1st Strand cDNA Synthesis Kit (Vazyme, Nanjing, China). Quantitative real-time PCR was performed using cDNA, gene-specific primers, and the UltraSYBR Mixture (CW0957M; CWBIO, Taizhou, China) according to the manufacturer’s instructions. The thermal cycling protocol consisted of an initial denaturation at 95°C for 10 min, followed by 40 cycles of 94°C for 30 s, 60°C for 30 s, and 72°C for 30 s. Gene expression levels were normalized to GAPDH and quantified using the 2^−ΔΔCT^ method. All primer sequences are listed in Table S1 (20–22).

### Western blotting

Protein extracts from lung tissues and MH-S cells were obtained using Mammalian Protein Extraction Reagent (Solarbio, Beijing, China), and concentrations were quantified via a BCA assay. After separation by 10% SDS-PAGE, proteins were transferred to PVDF membranes. The membranes were incubated with primary antibodies (Table S2), followed by an HRP-conjugated secondary antibody. Immunoreactive bands were visualized using a Bio-Rad ChemiDoc MP imaging system. Band intensities for target proteins were normalized to β-actin for quantitative analysis.

### Immunofluorescence assays

After the respective treatments, cells, including peritoneal macrophages, alveolar macrophages, and MH-S, were permeabilized using 1% Triton X-100 (1 h, 37°C). Blocking was performed with 5% goat serum (1 h, 37°C) prior to overnight incubation at 4°C with the appropriate primary antibodies (Table S3). The samples were subsequently exposed to an Alexa Fluor 488-labeled anti-rabbit IgG secondary antibody (1:100; A11034, Invitrogen, CA, USA) for 24 h at 4°C. Nuclear staining was achieved with DAPI (0100-20; SouthernBiotech, AL, USA; 5 min, RT). Negative controls received PBS in place of the primary antibody. All fluorescence images were obtained with an inverted microscope (T00423; ZEISS, Baden-Württemberg, Germany).

### Statistical analysis

Where appropriate, two-group comparisons were made with two-tailed unpaired Student’s *t* tests, whereas multi-group comparisons employed one-way analysis of variance coupled with Dunnett’s *post hoc* test. The assumptions of normality and equal variance were verified for all data sets before analysis. Values are shown as mean ± standard deviation. A *P* value below 0.05 was considered statistically significant, with *P* < 0.01 and *P* < 0.001 indicating greater significance. All analyses were carried out using GraphPad Prism 9 (GraphPad Software, San Diego, CA, USA).

## Data Availability

All data generated or analyzed during this study are included in this published article. Further details are available from the corresponding author on reasonable request.

## SUPPLEMENTAL MATERIAL

Fig. S1 to S4, Tables S1 to S3, and Additional experimental details. spectrum.01040-26-s0001.docx Experimental materials and fluorescence images of peritoneal macrophages.

Fig. S2, S4, S7, S9, and S10 spectrum.01040-26-s0002.pdf WB-raw data.

ASM does not own the copyrights to Supplemental Material that may be linked to, or accessed through, an article. The authors have granted ASM a non-exclusive, world-wide license to publish the Supplemental Material files. Please contact the corresponding author directly for reuse.
