# Supplementary material for: GABABR activation alleviates Streptococcus equi subsp. zooepidemicus-induced acute lung injury by suppressing inflammation and oxidative stress in pulmonary macrophages via IRAK3
Source: Microbiol Spectr. 2026 Oct 6;14(11):e01040-26. doi: 10.1128/spectrum.01040-26 (PMC13729176; doi:10.1128/spectrum.01040-26)

Figure 2 M Claudin-1(repeated 1)

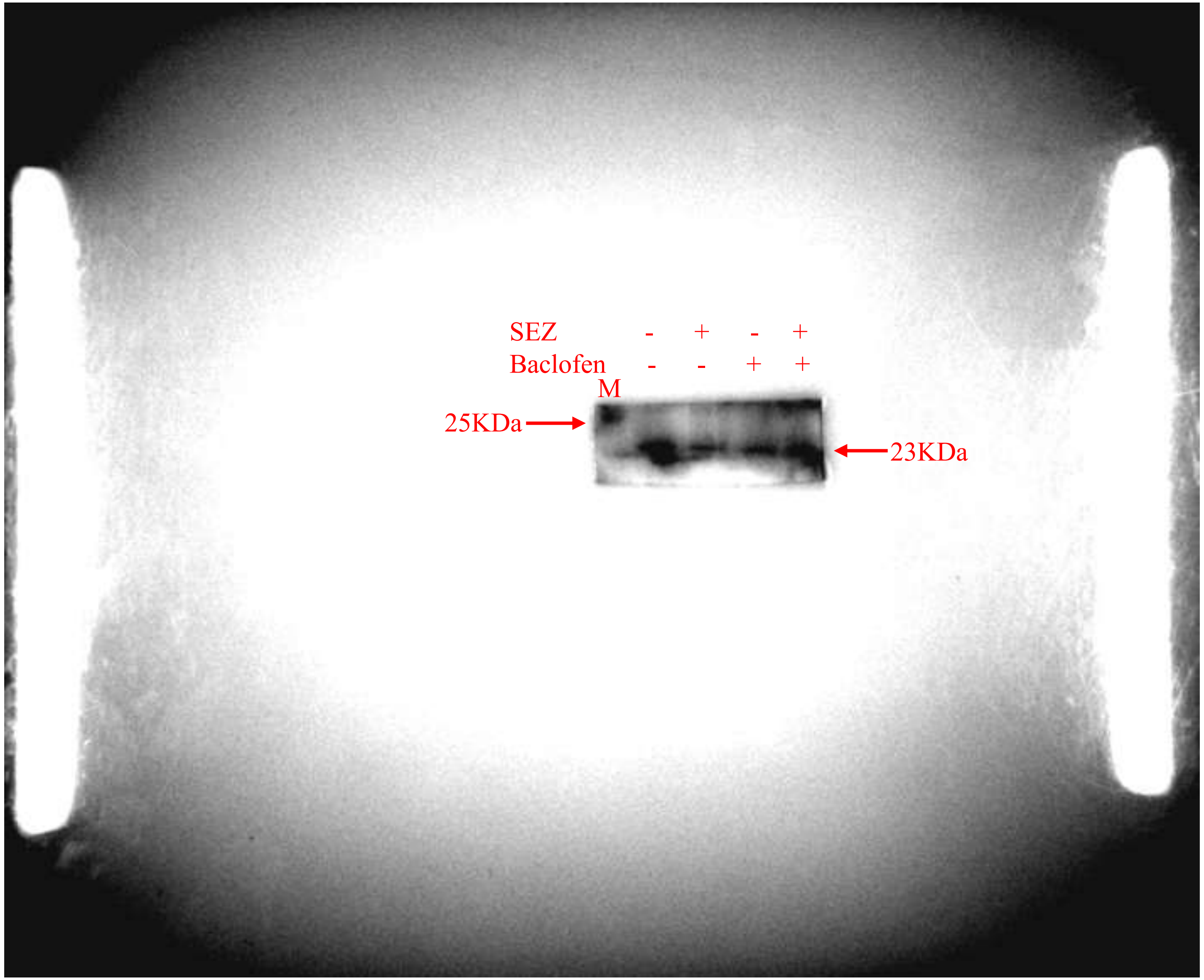

Figure 2 M Claudin-1(repeated 2)

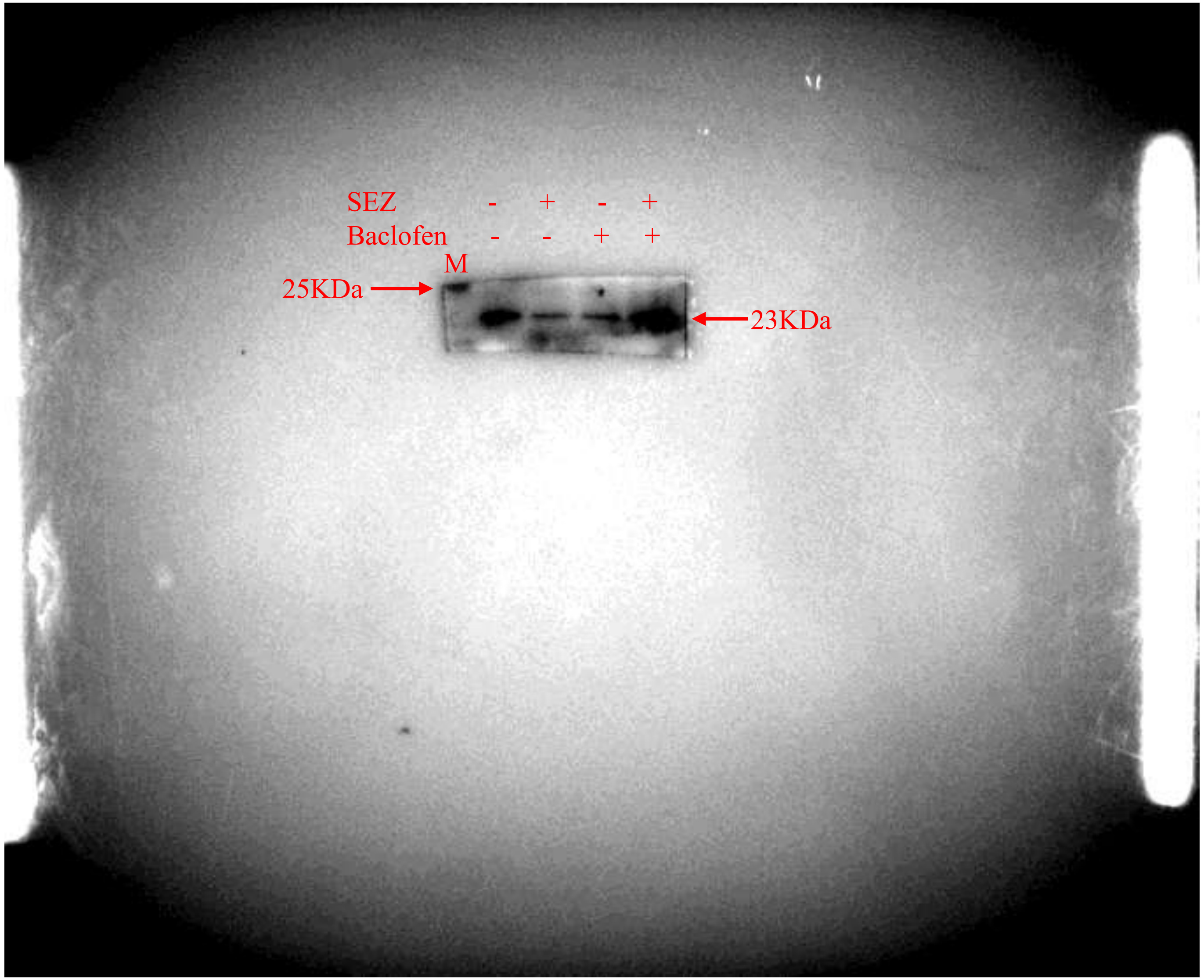

Figure 2 M Claudin-1(repeated 3)

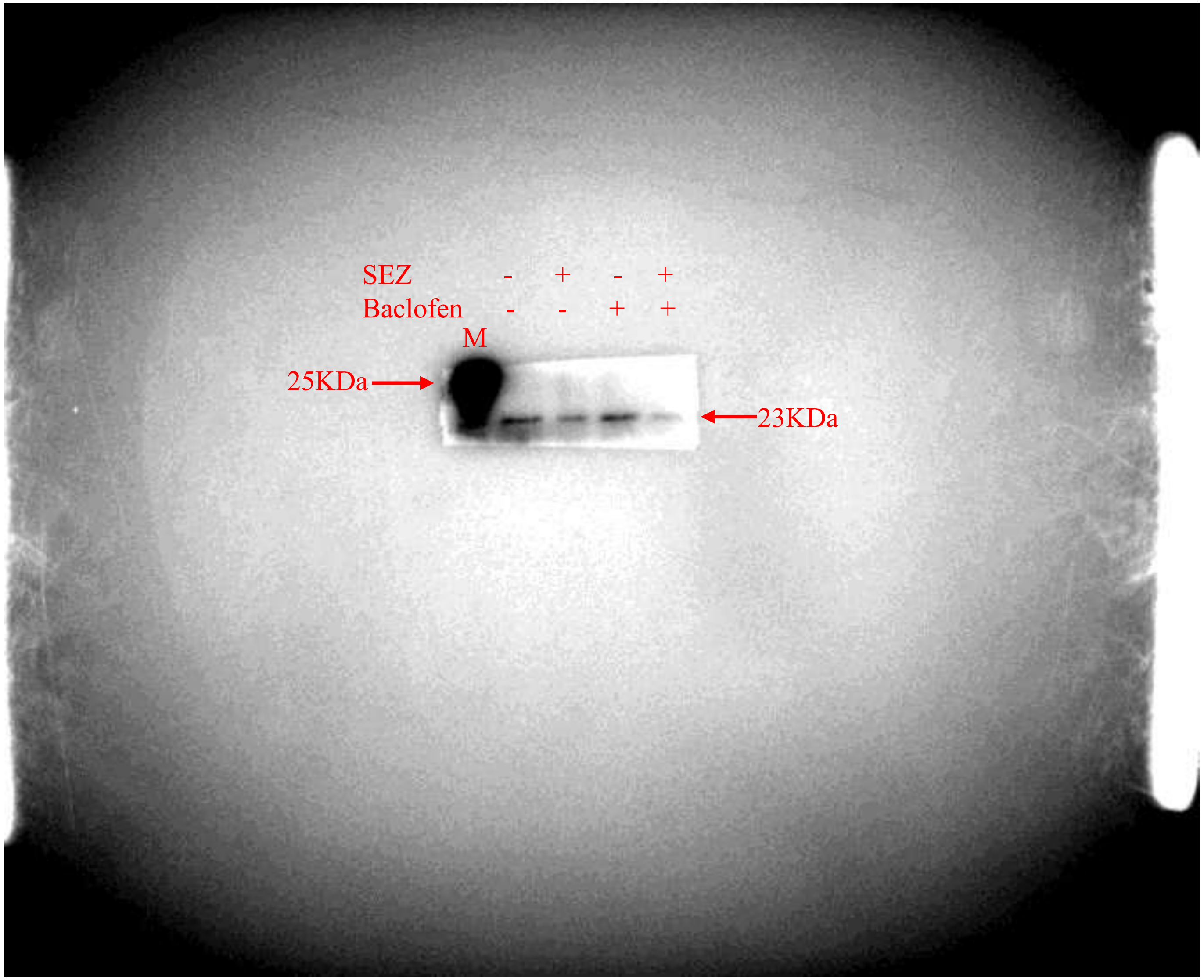

Figure 2 M Occludin (repeated 1)

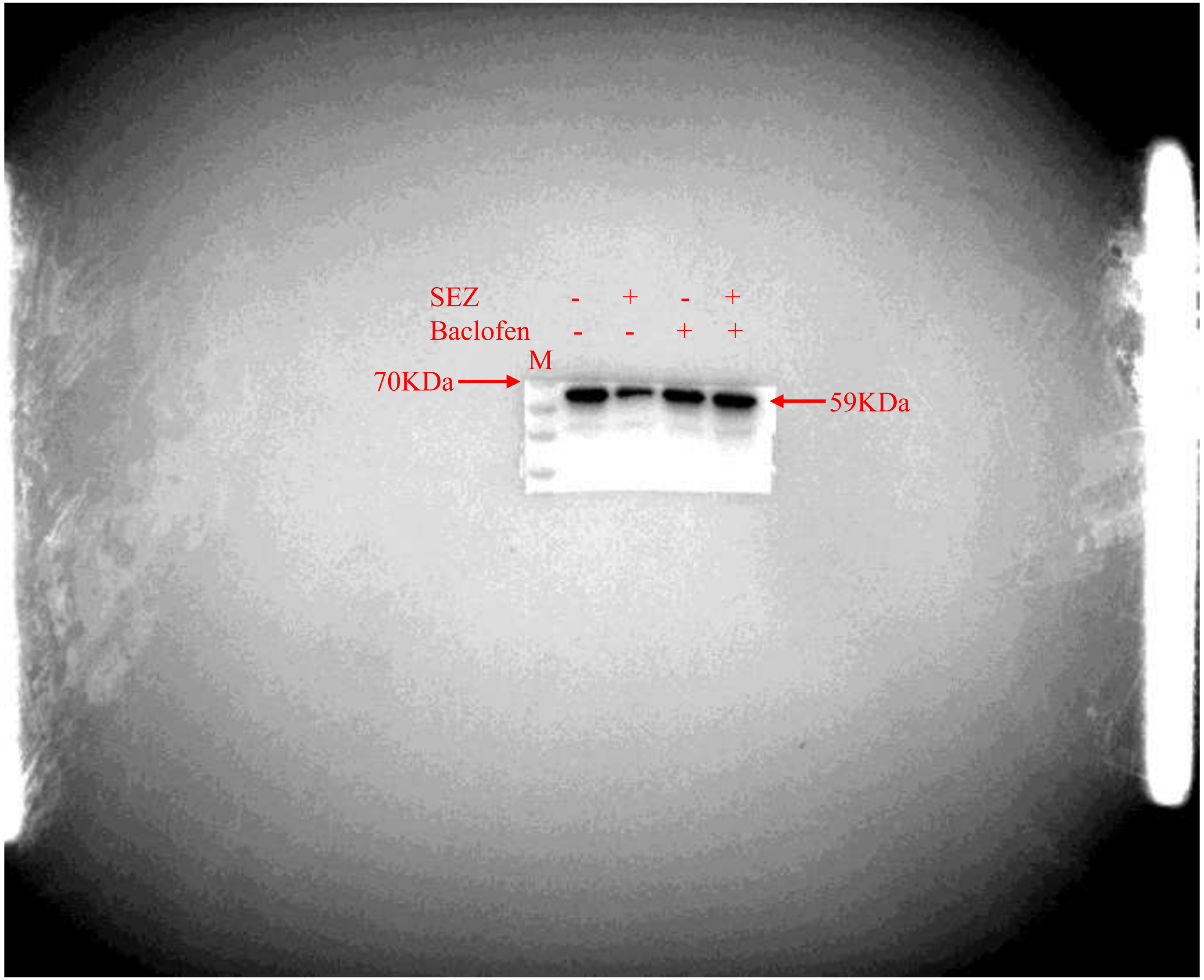

Figure 2 M Occludin (repeated 2)

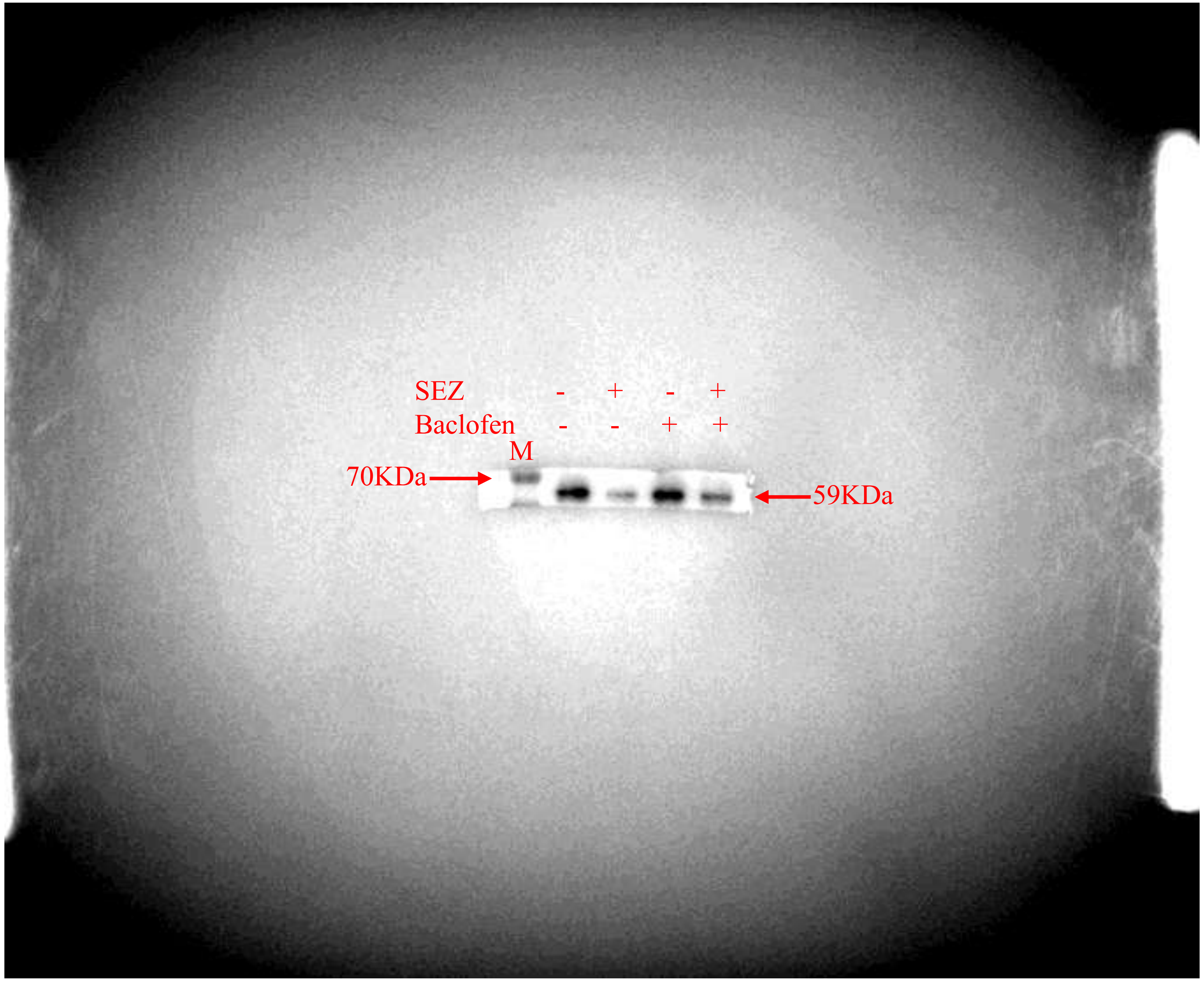

Figure 2 M Occludin (repeated 3)

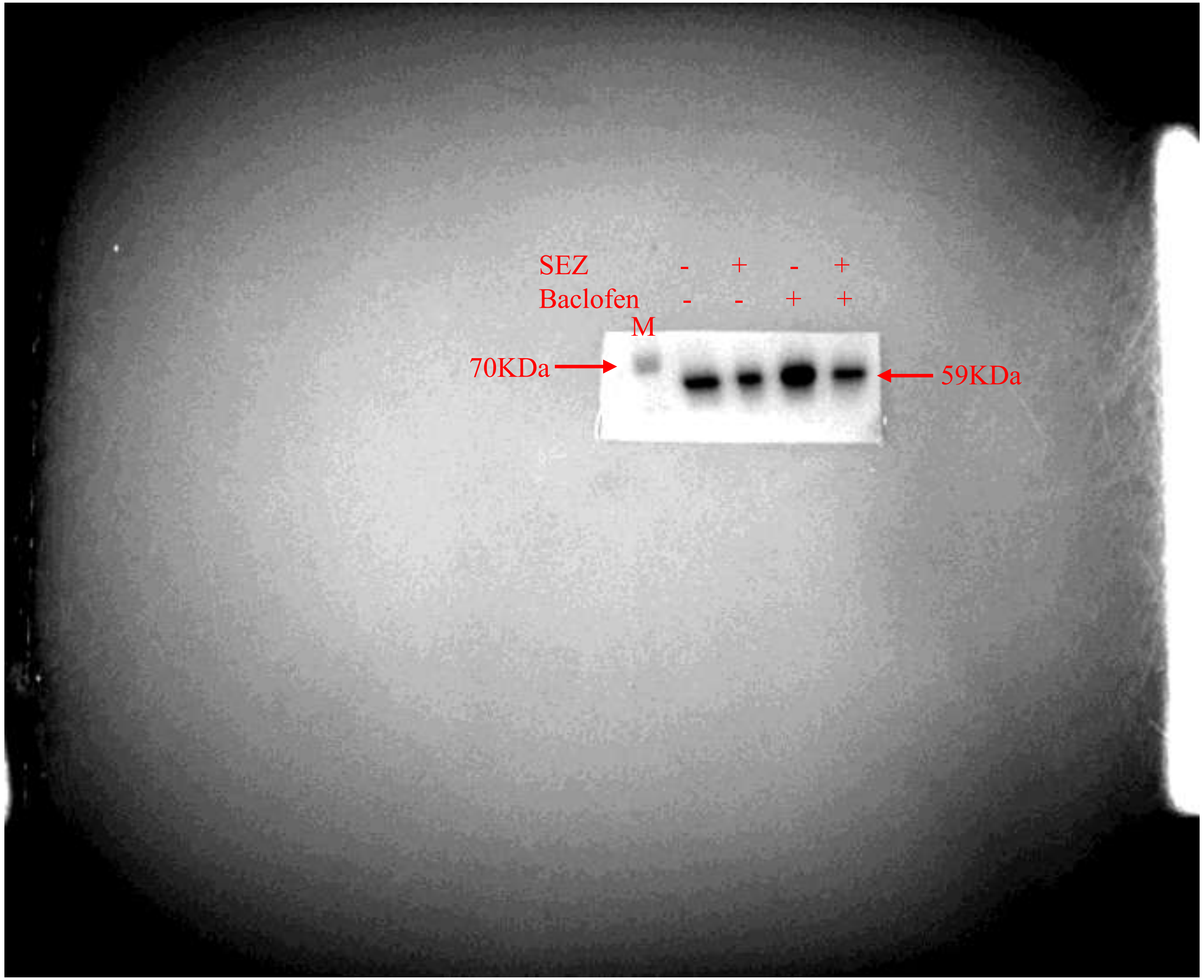

Figure 2 M ZO-1 (repeated 1)

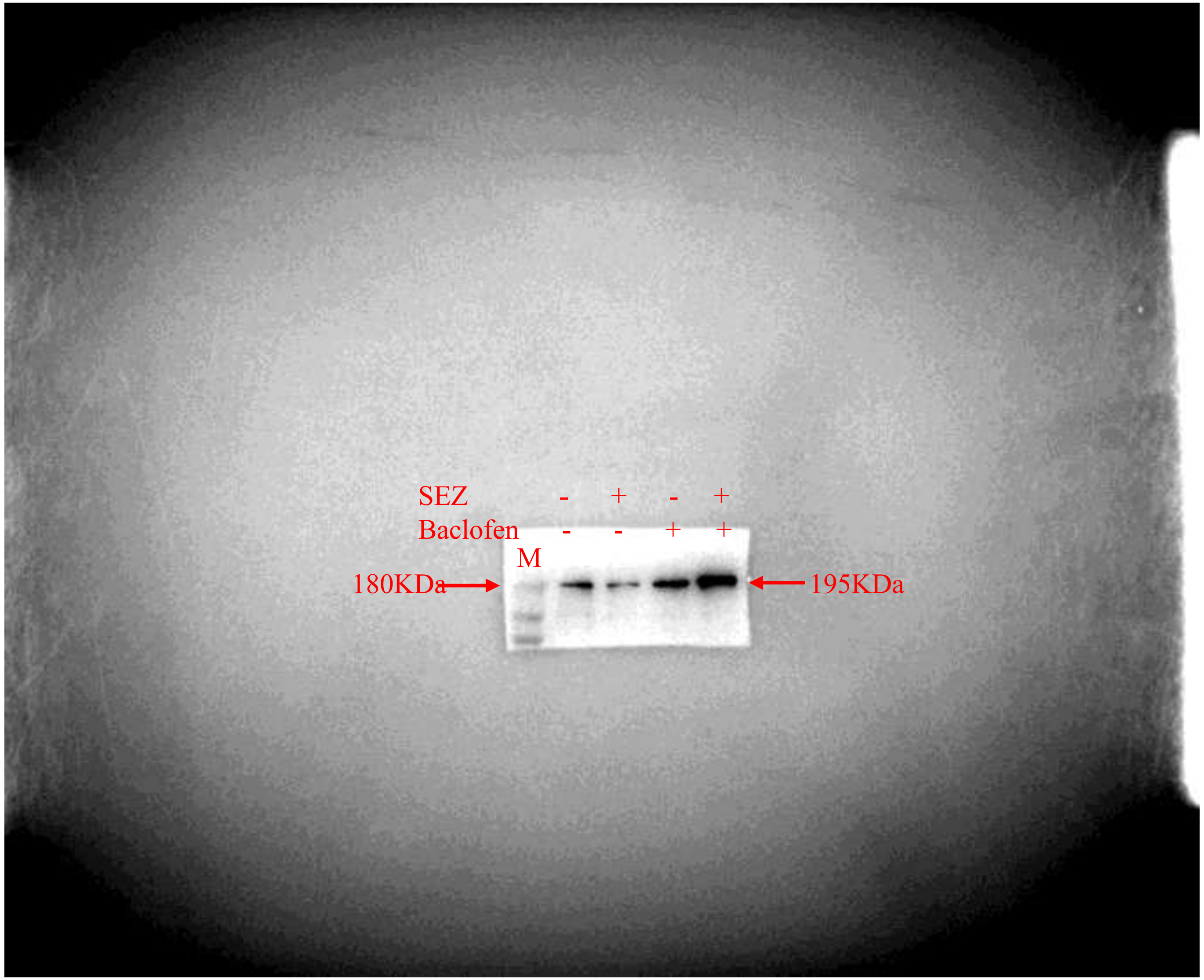

Figure 2 M ZO-1 (repeated 2)

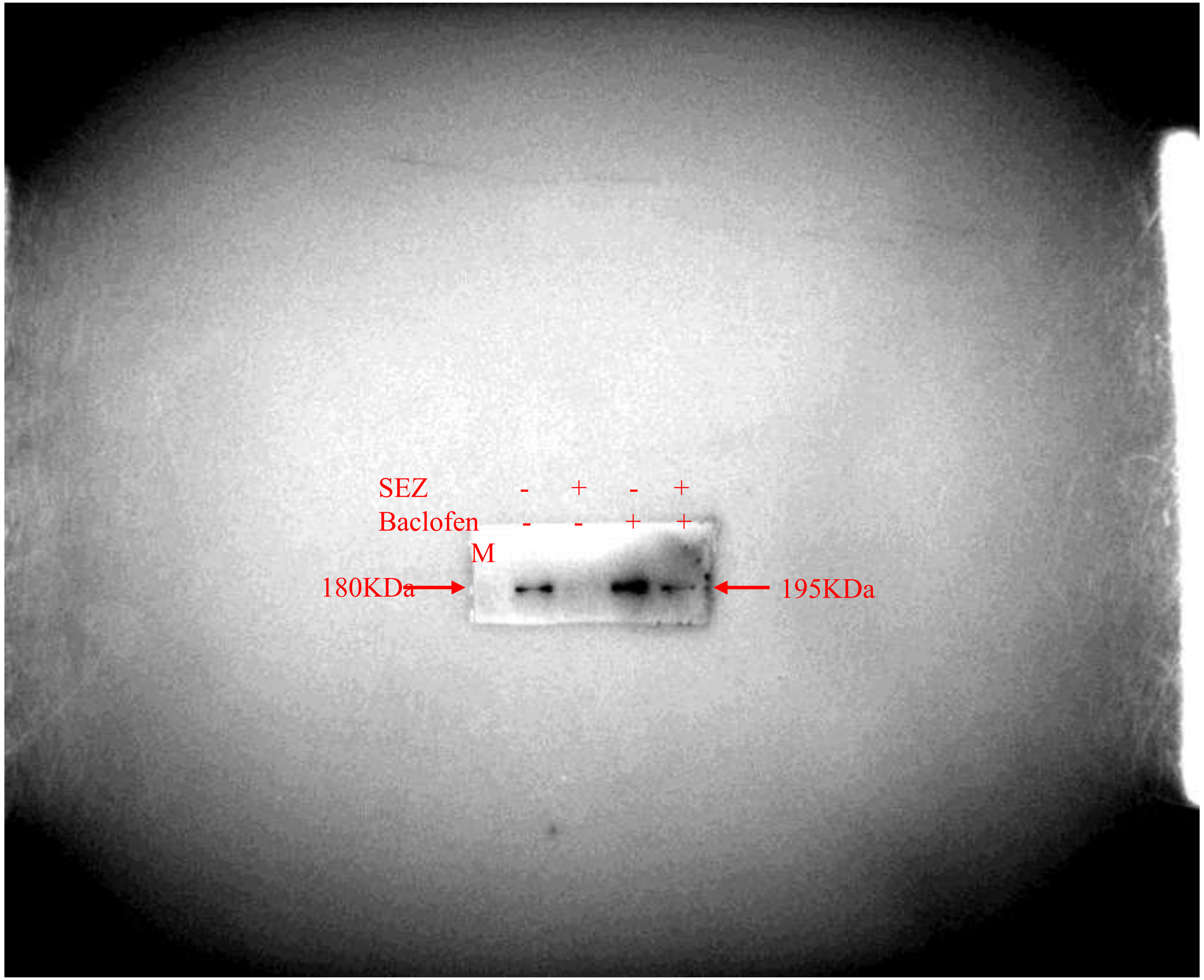

Figure 2 M ZO-1 (repeated 3)

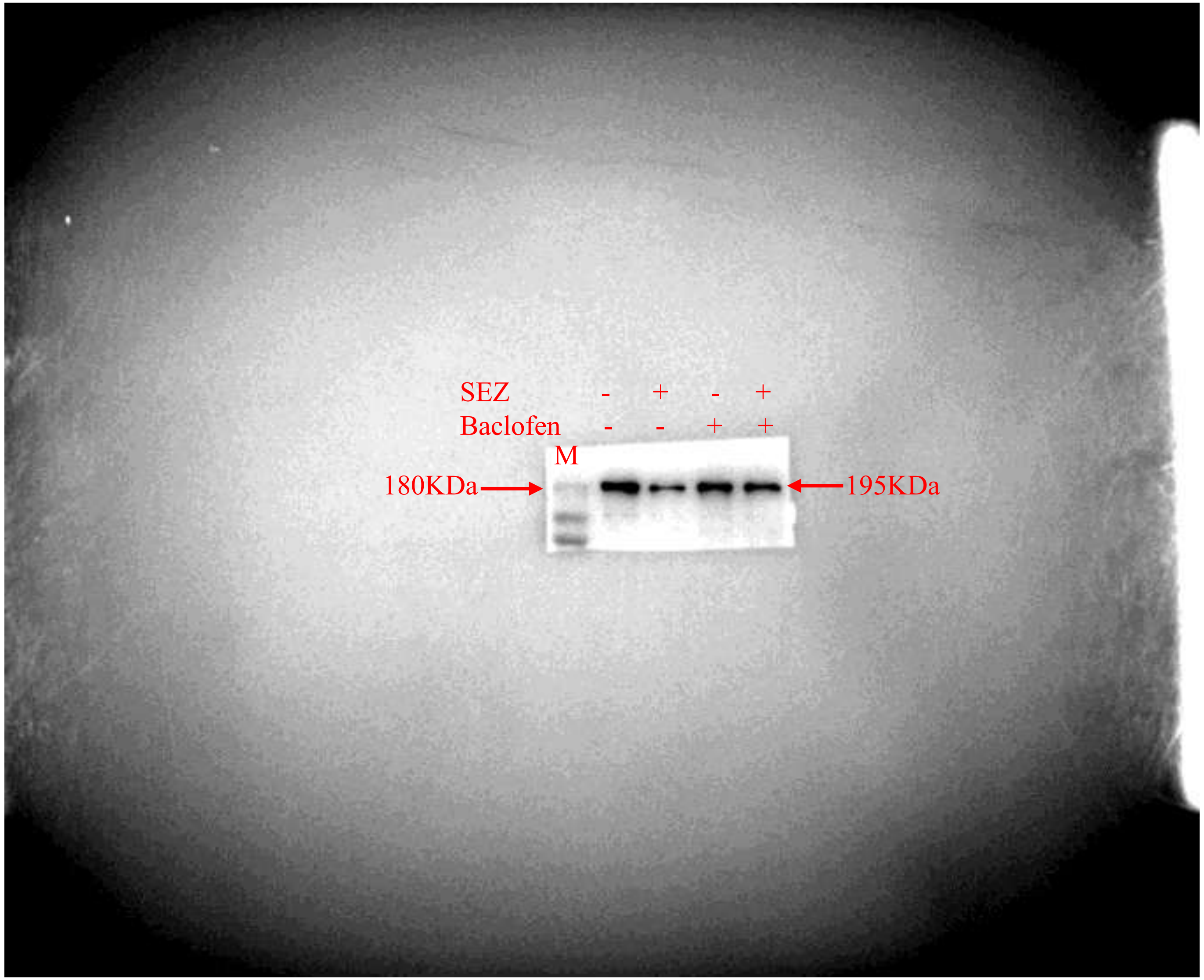

Figure 2 M β-actin (repeated 1)

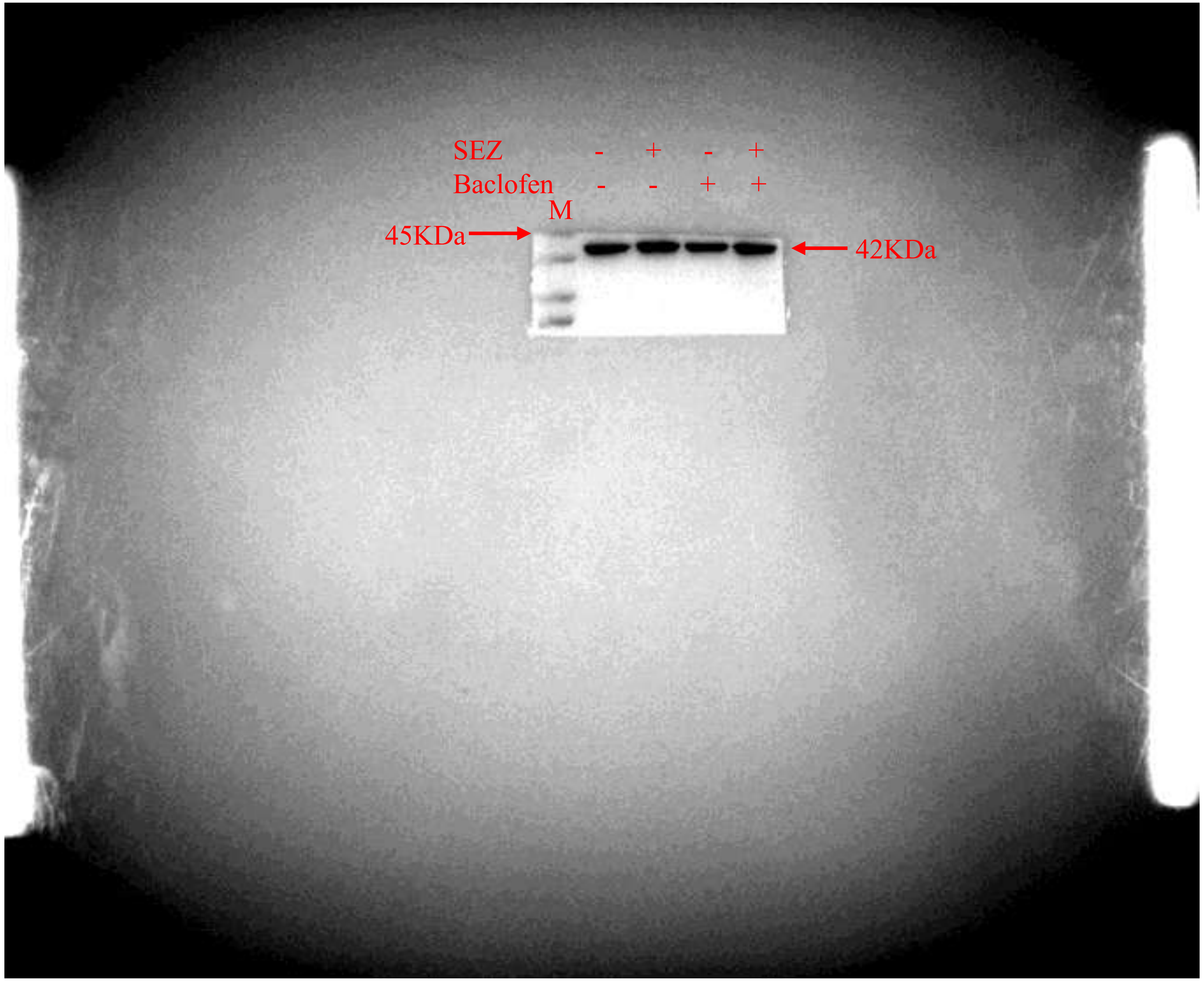

Figure 2 M β-actin (repeated 2)

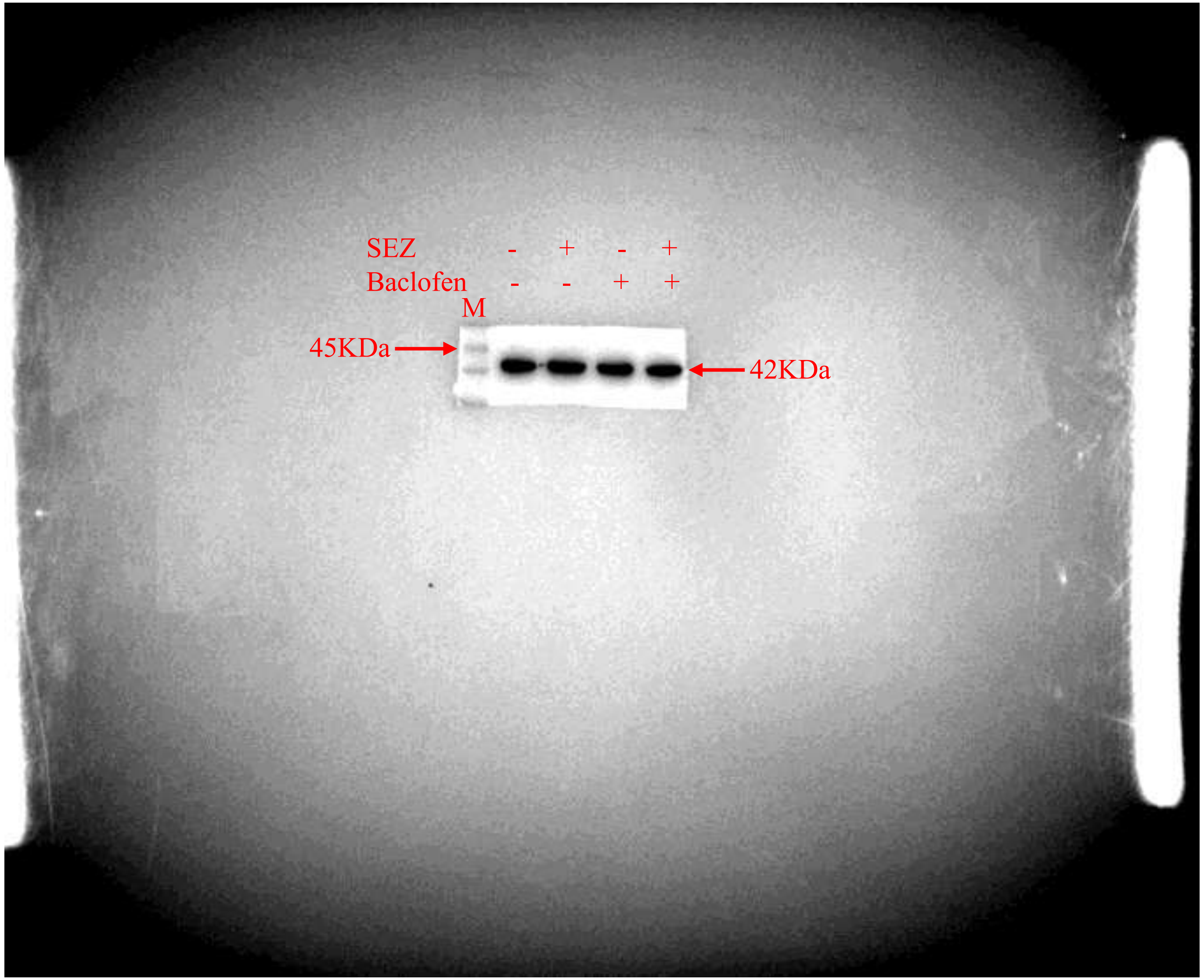

Figure 2 M β-actin (repeated 3)

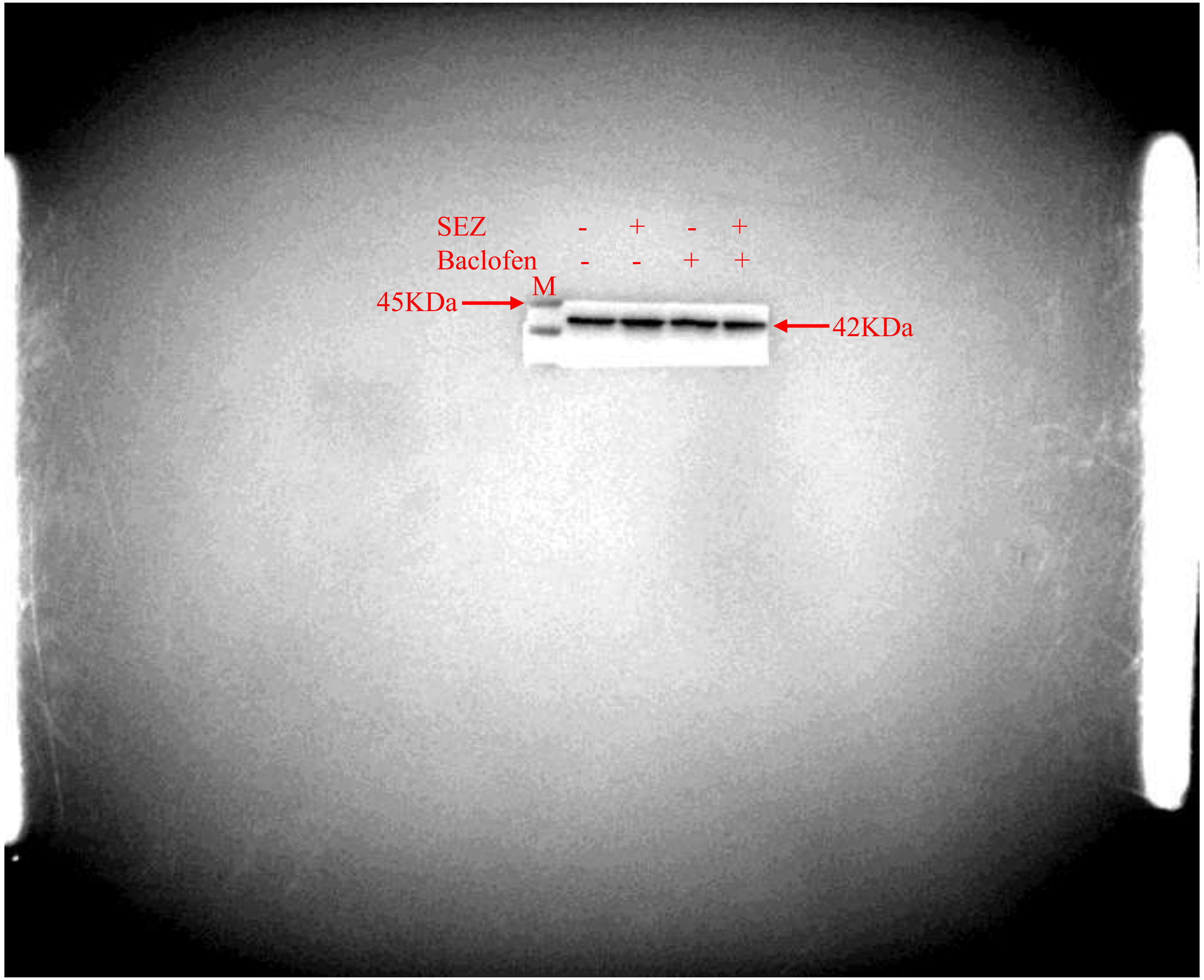

Figure 2 N GPX4 (repeated 1)

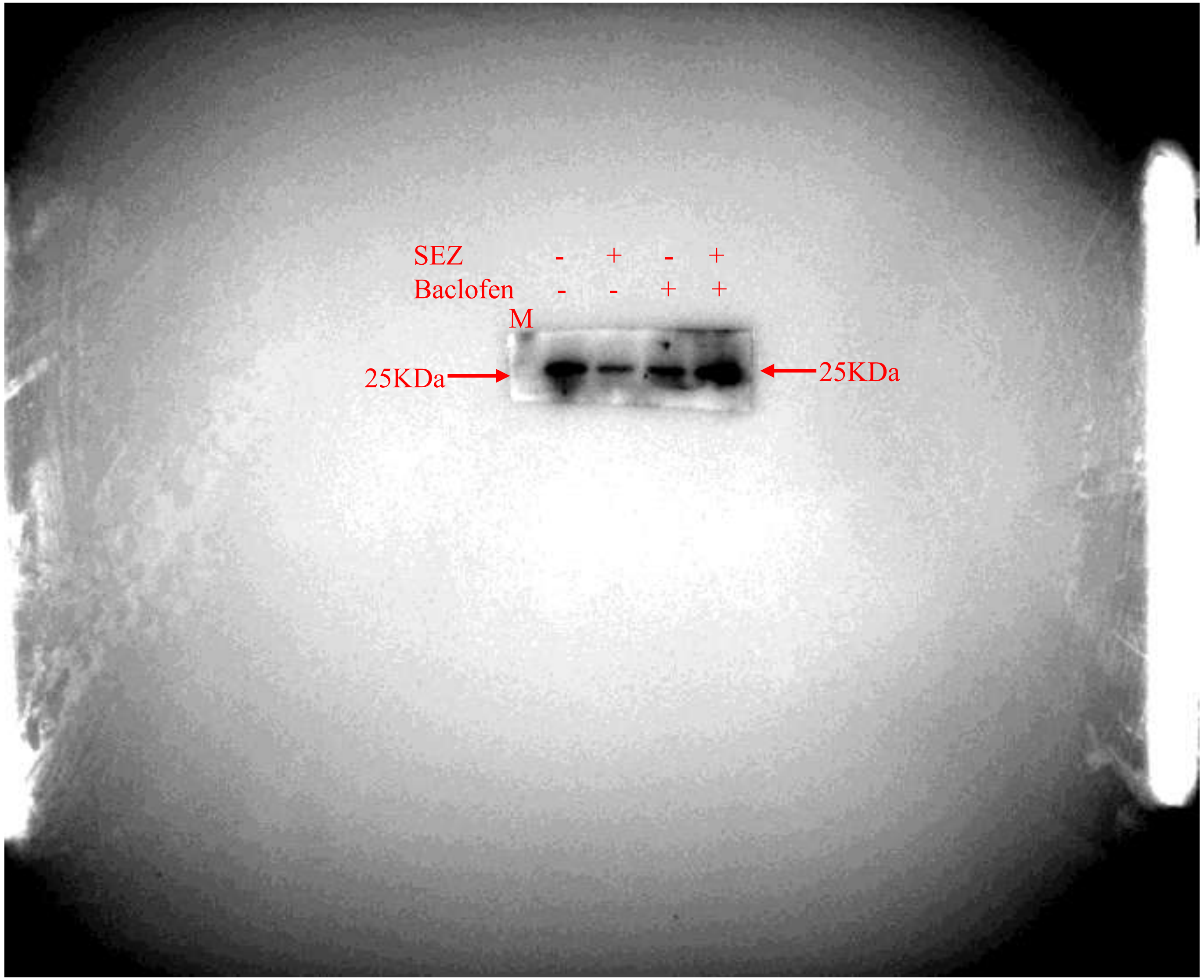

Figure 2 N GPX4 (repeated 2)

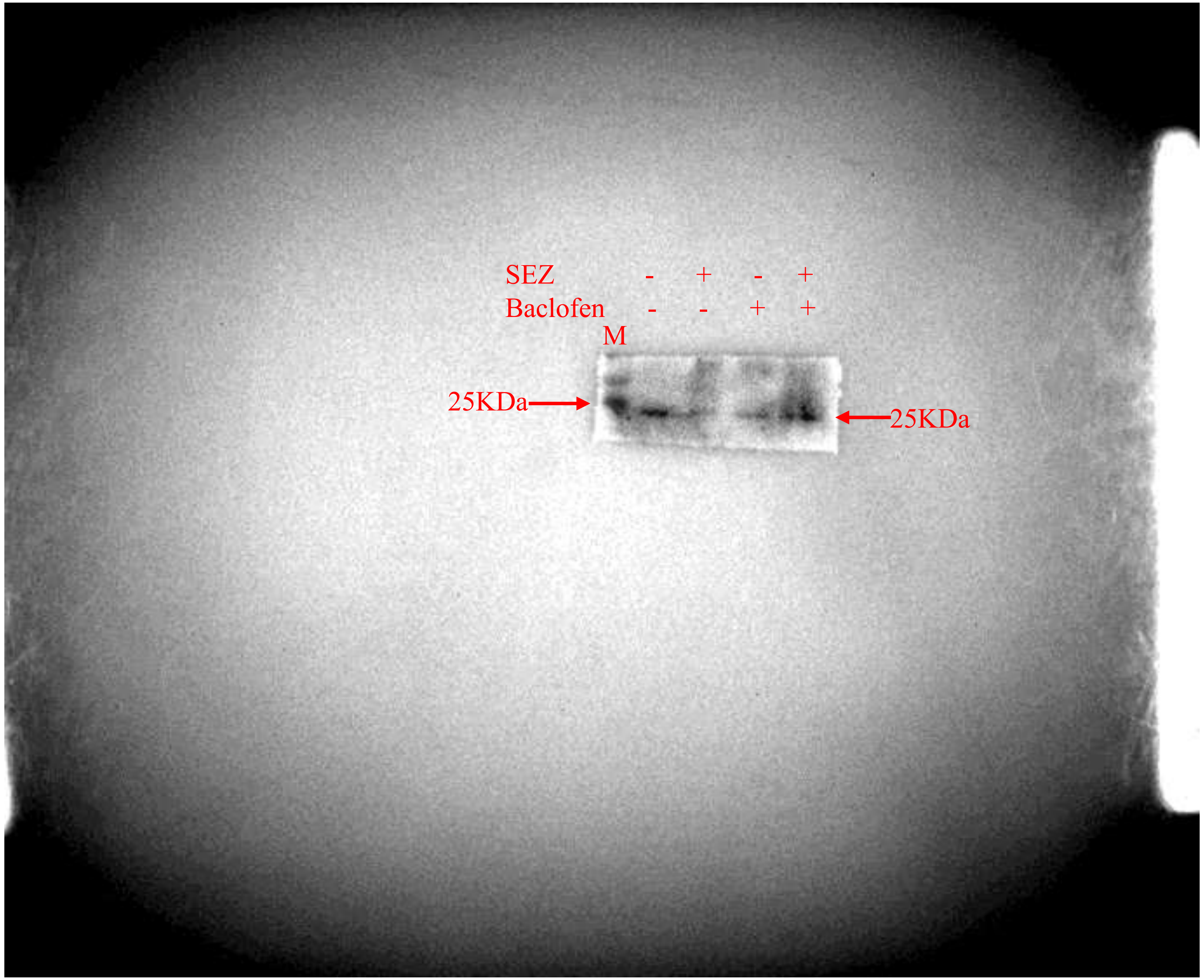

Figure 2 N GPX4 (repeated 3)

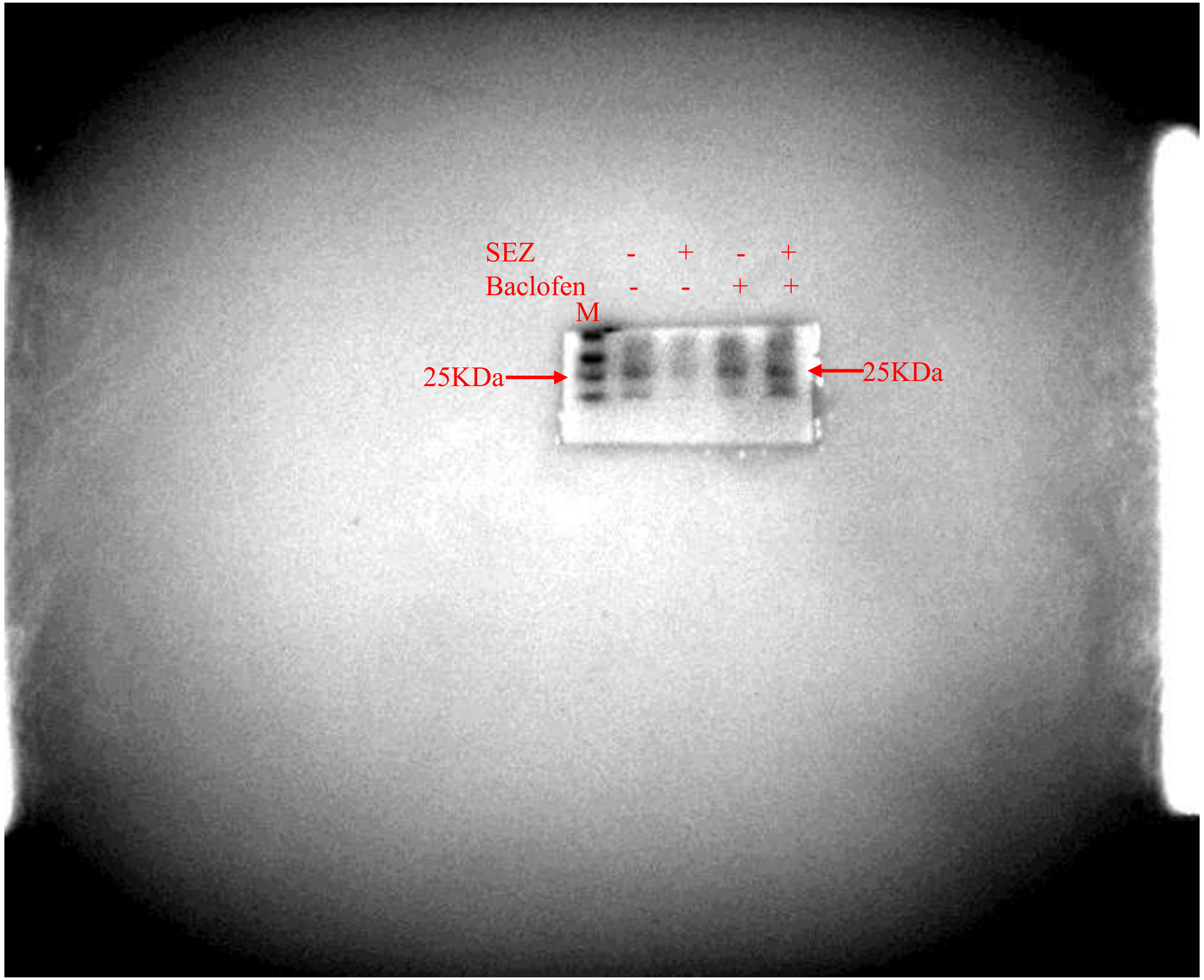

Figure 2 N HO-1 (repeated 1)

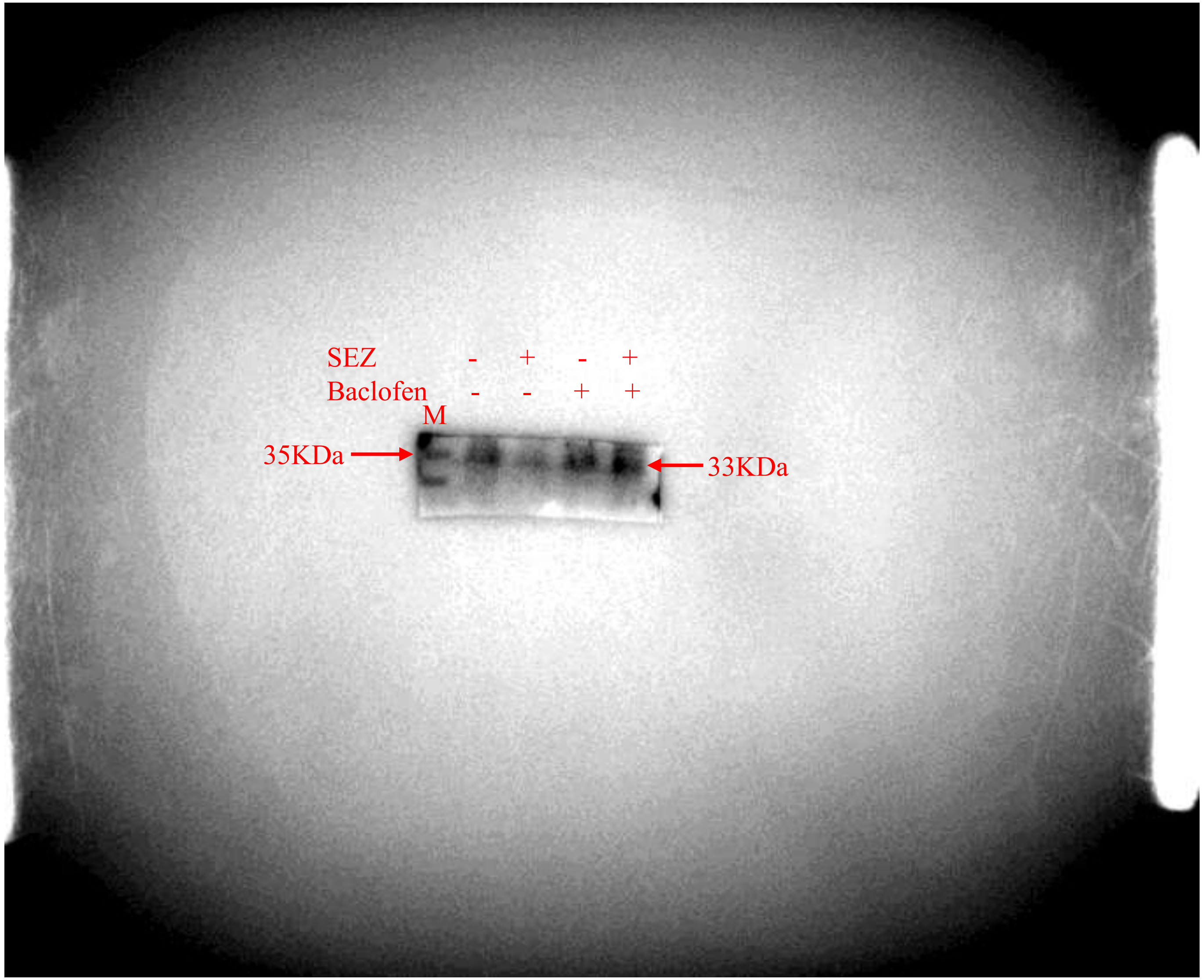

Figure 2 N HO-1 (repeated 2)

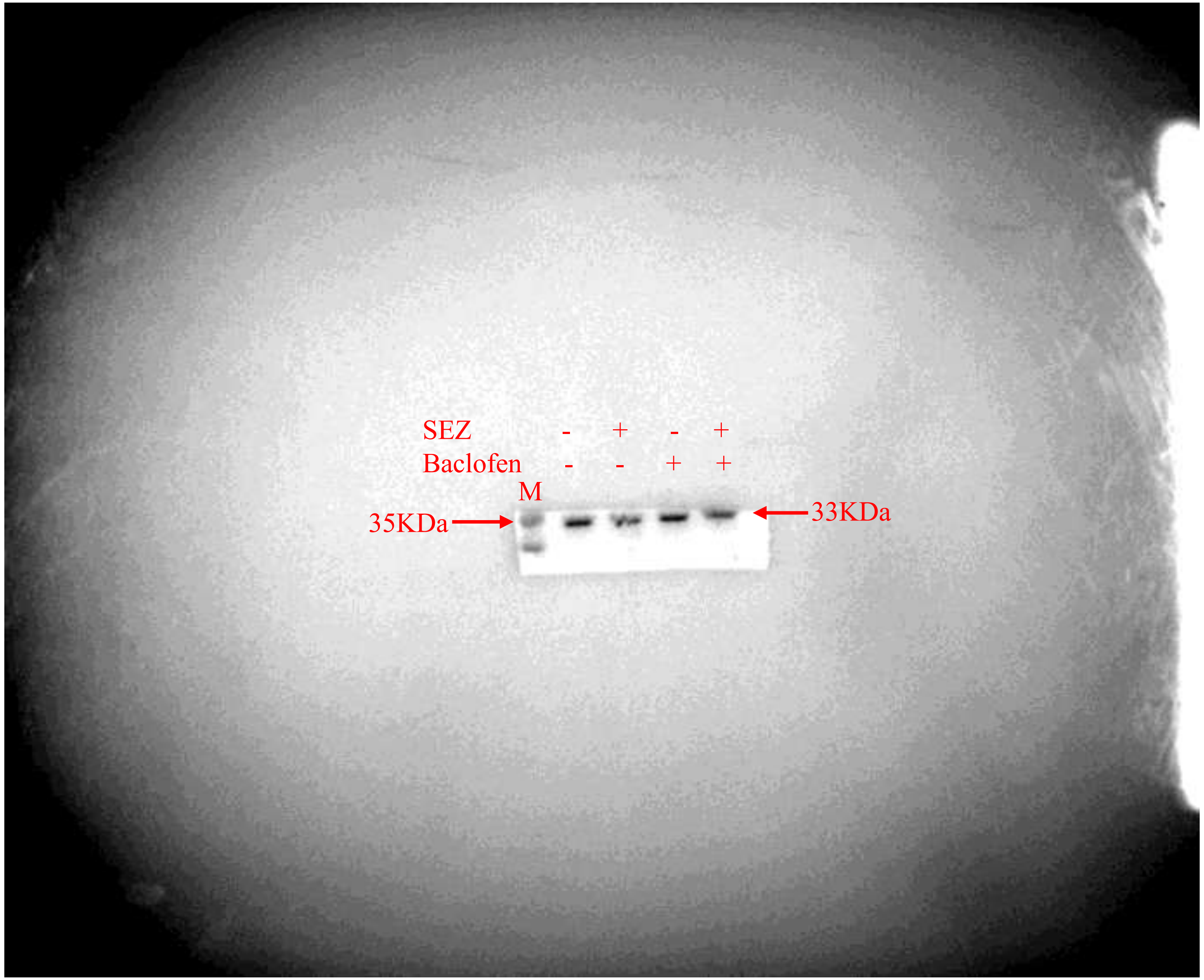

Figure 2 N HO-1 (repeated 3)

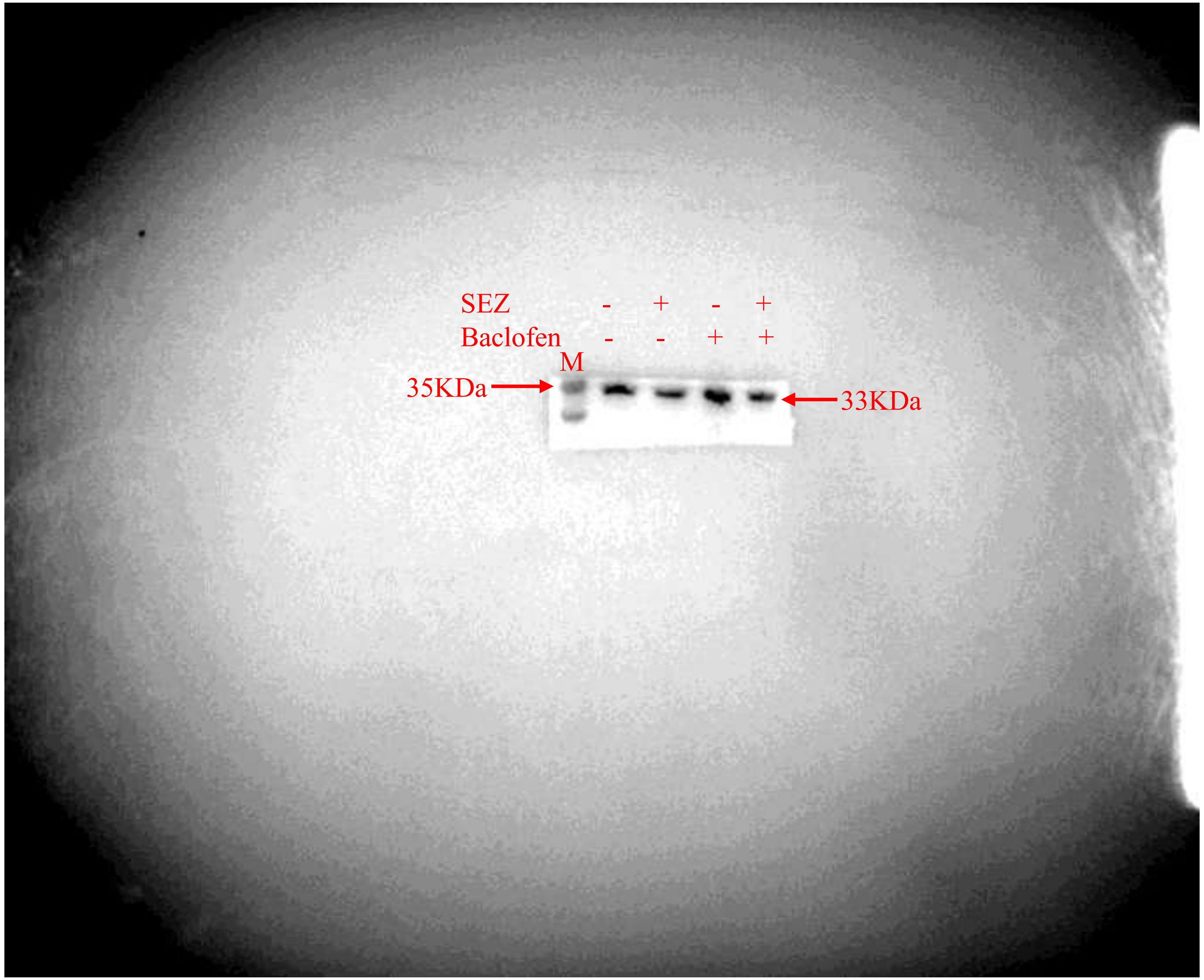

Figure 2 N iNOS (repeated 1)

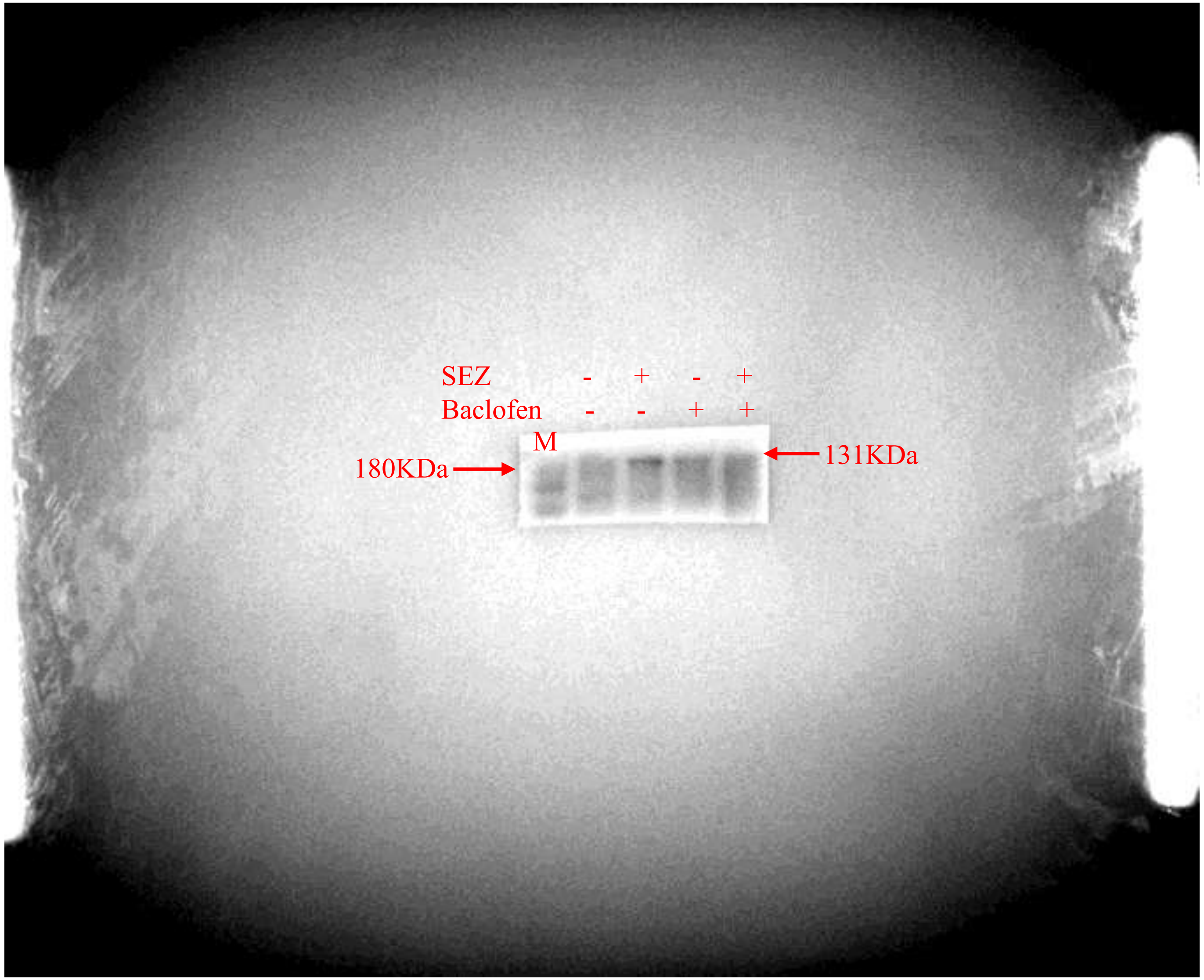

Figure 2 N iNOS (repeated 2)

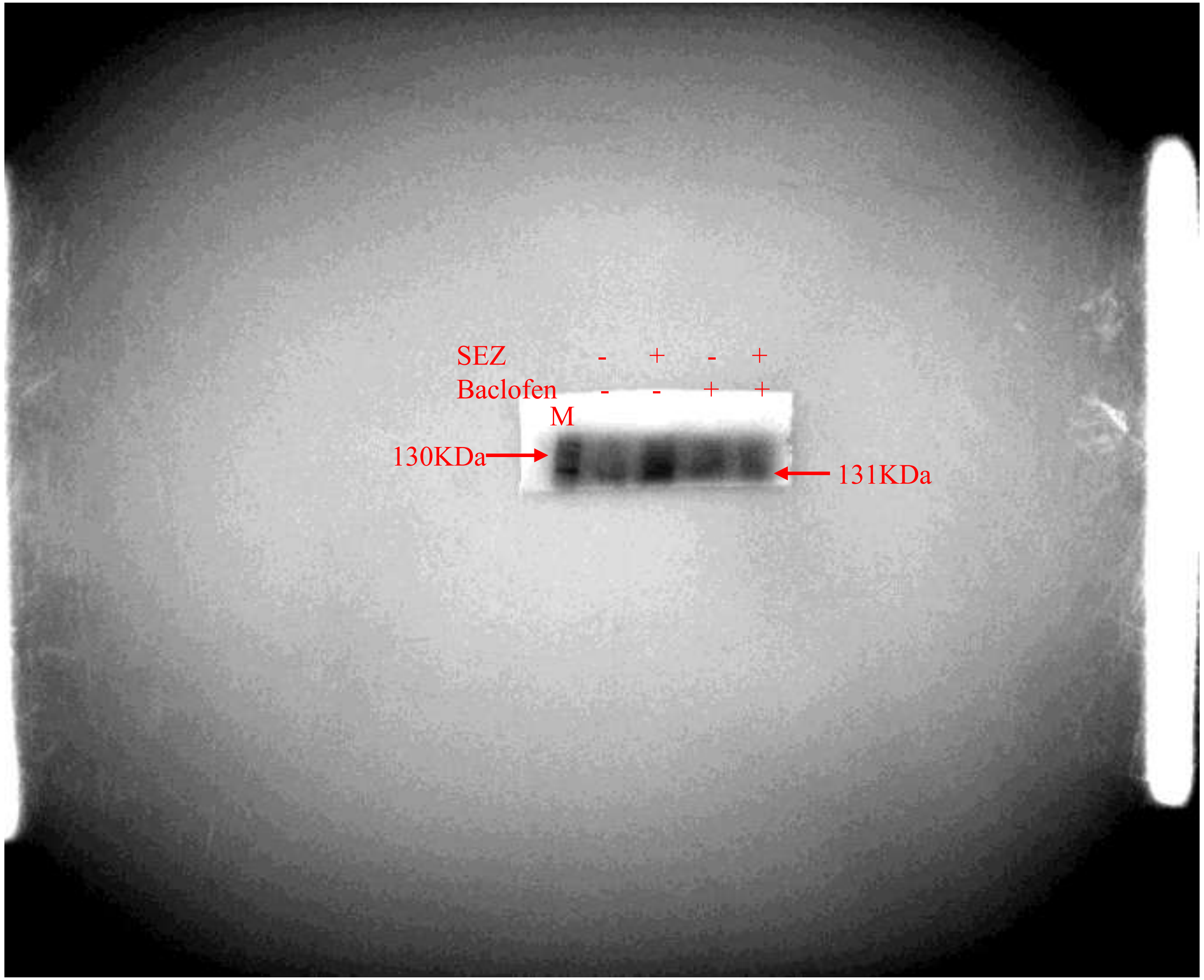

Figure 2 N iNOS (repeated 3)

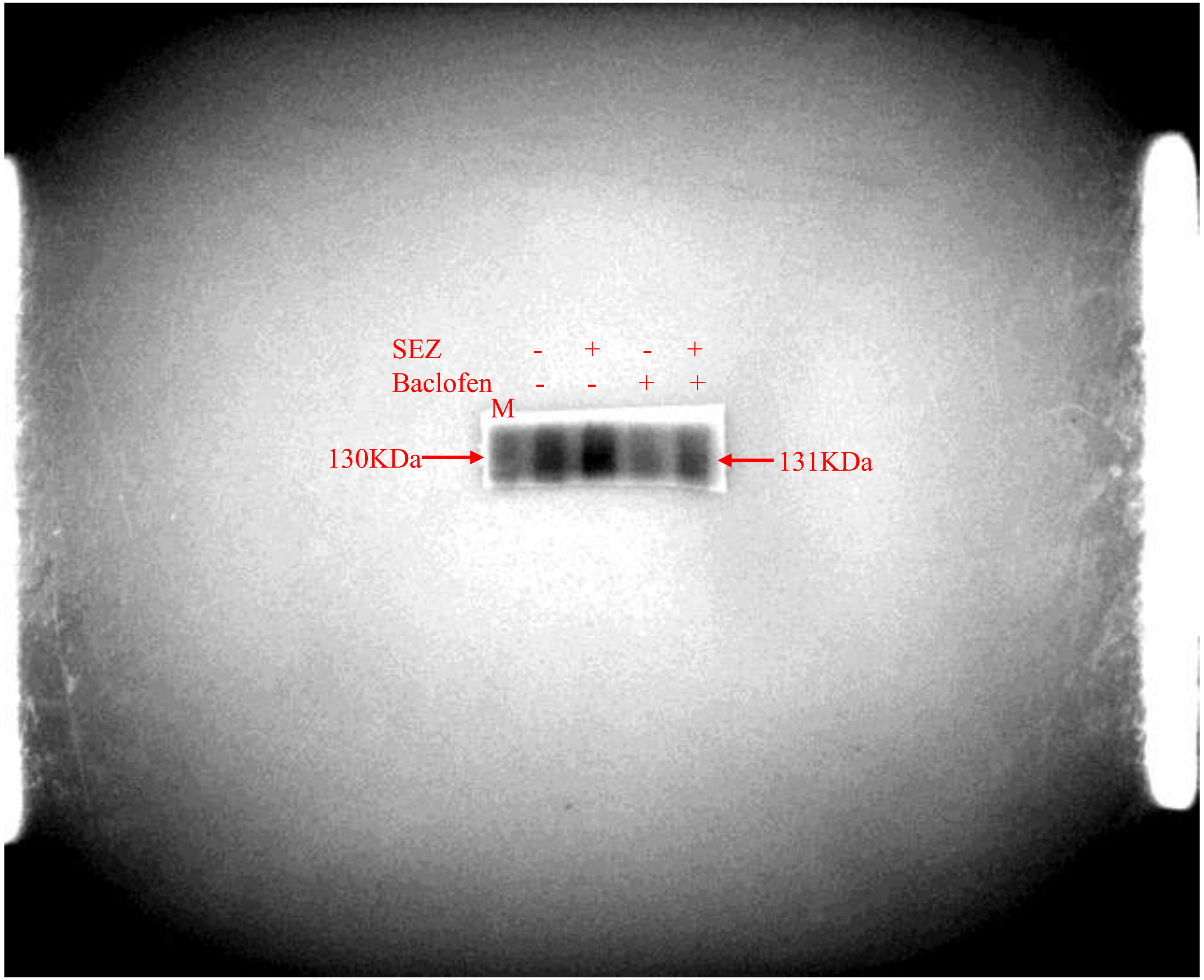

Figure 2 N NRF2 (repeated 1)

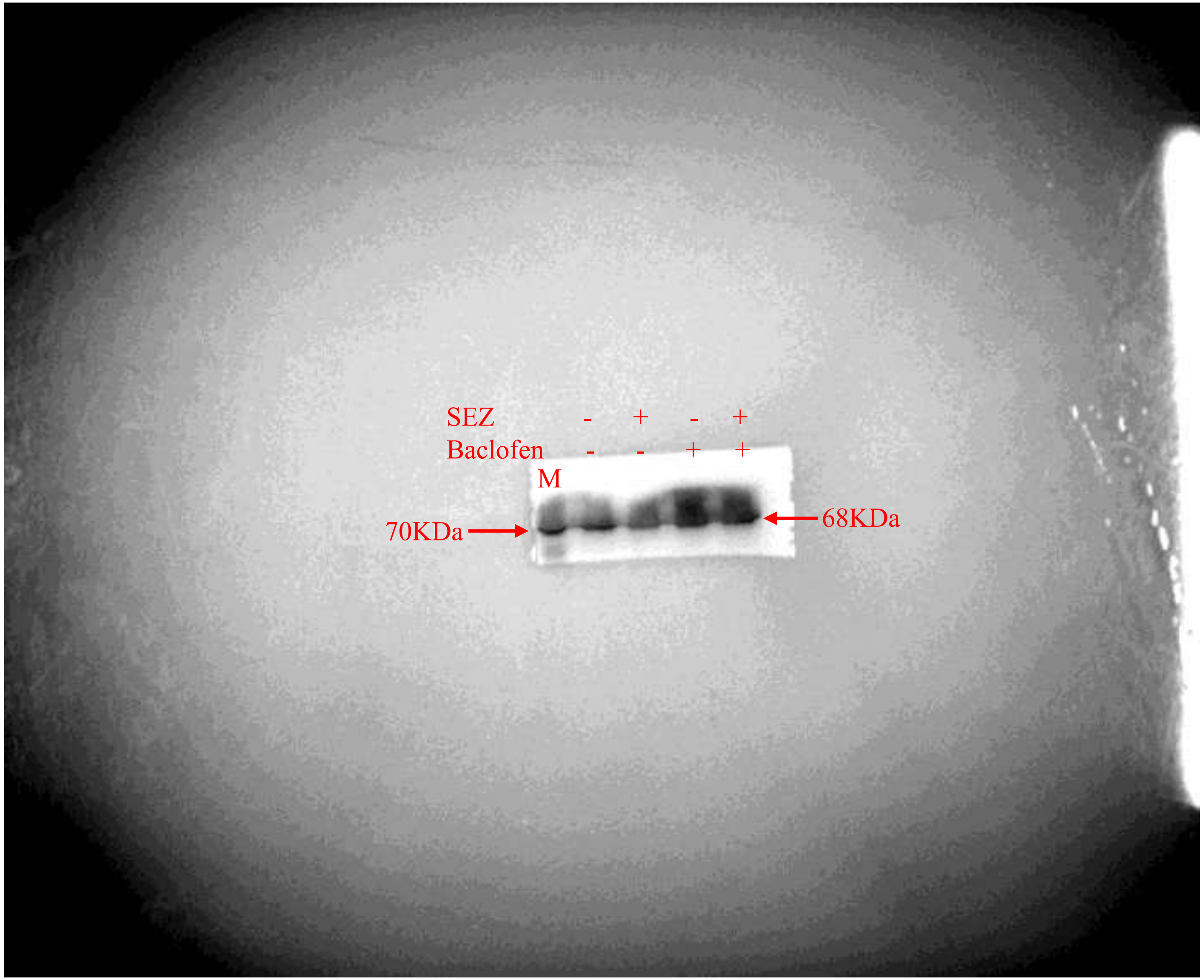

Figure 2 N NRF2 (repeated 2)

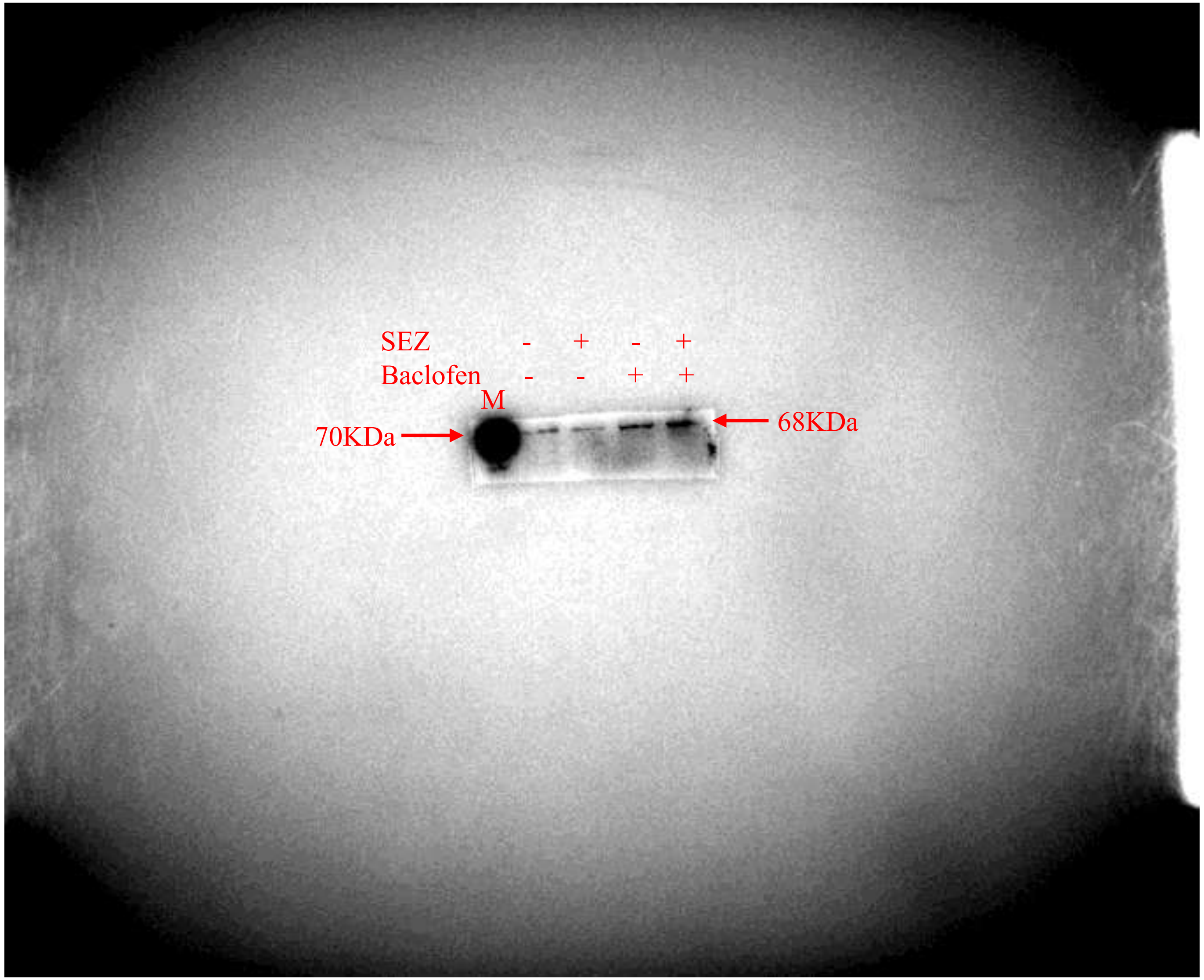

Figure 2 N NRF2 (repeated 3)

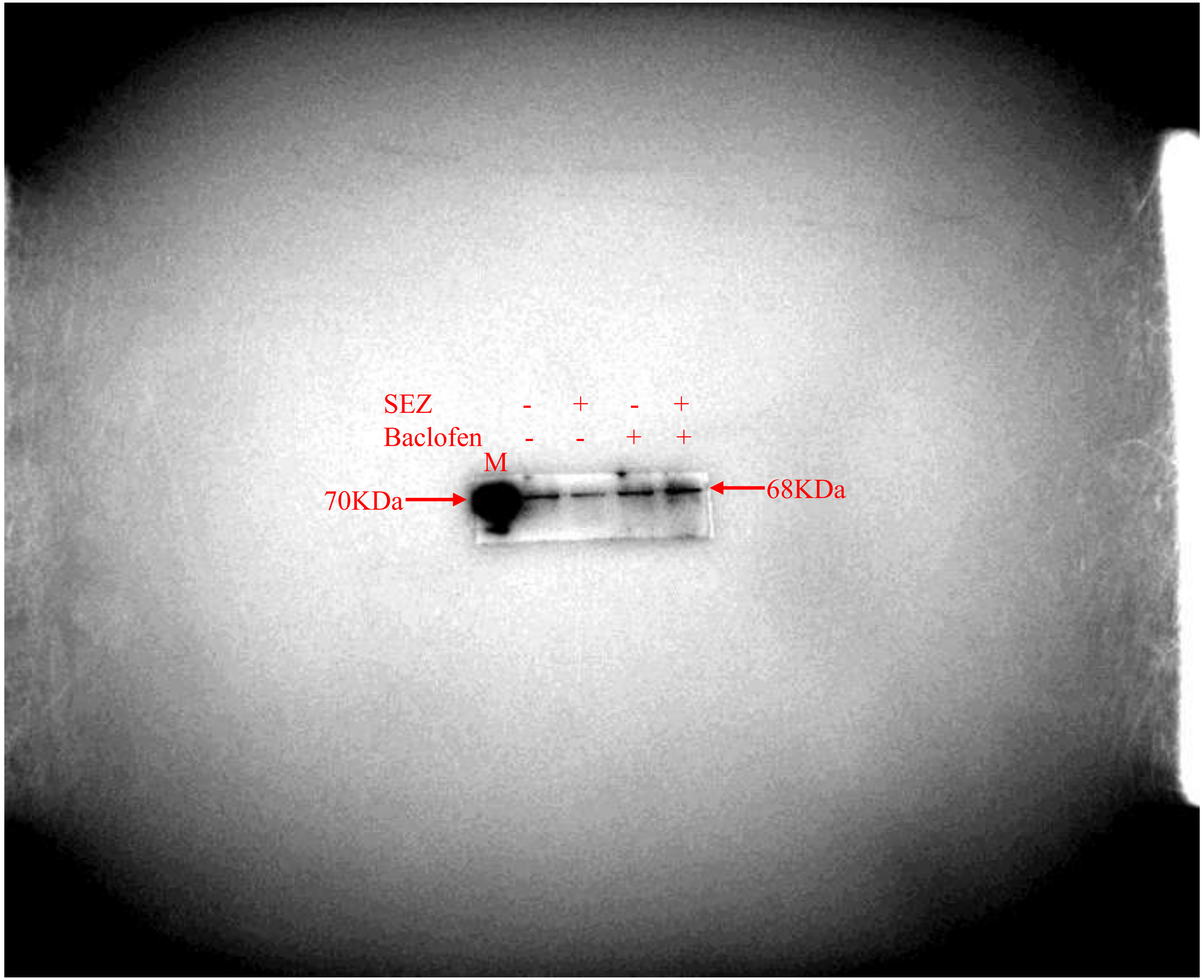

Figure 2 N β-actin (repeated 1)

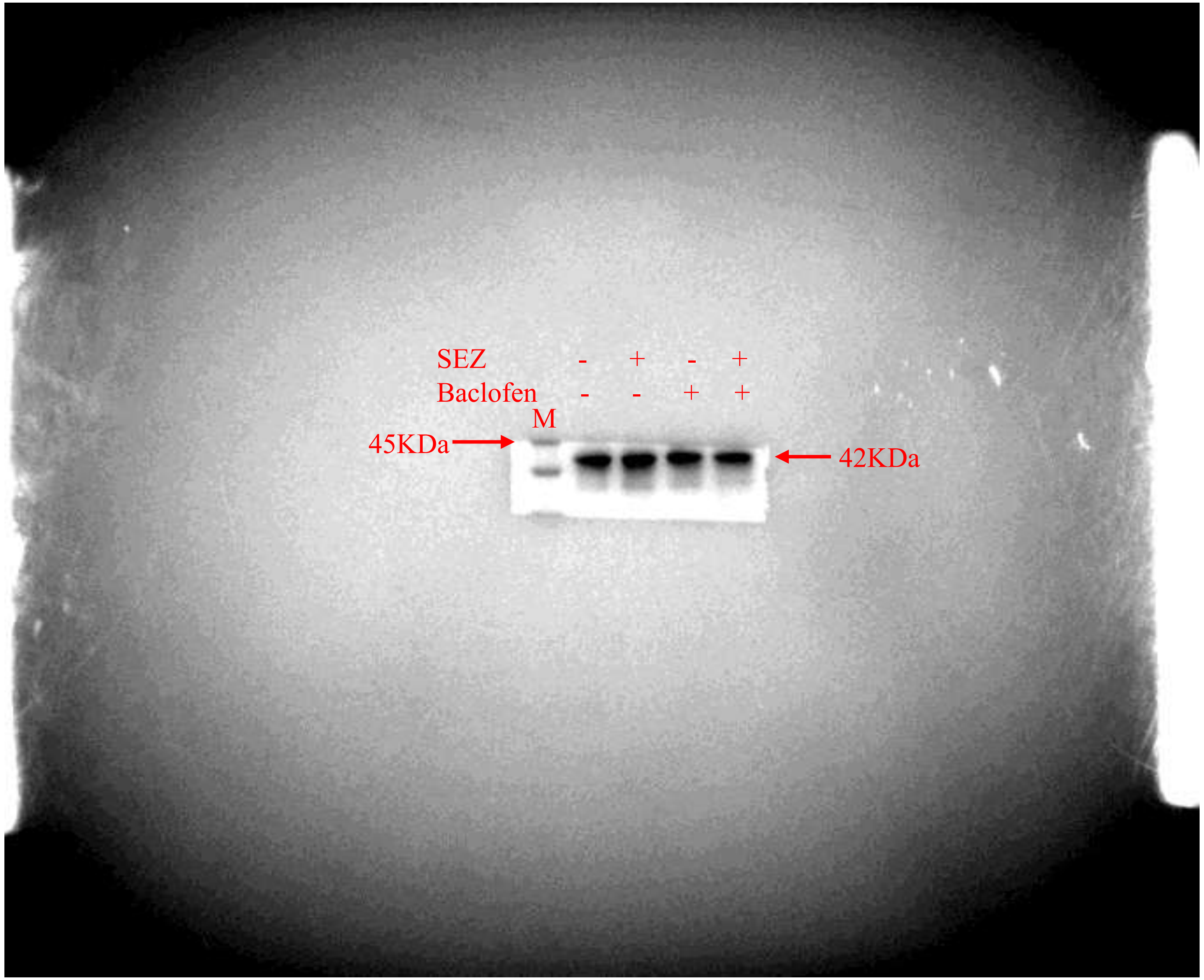

Figure 2 N β-actin (repeated 2)

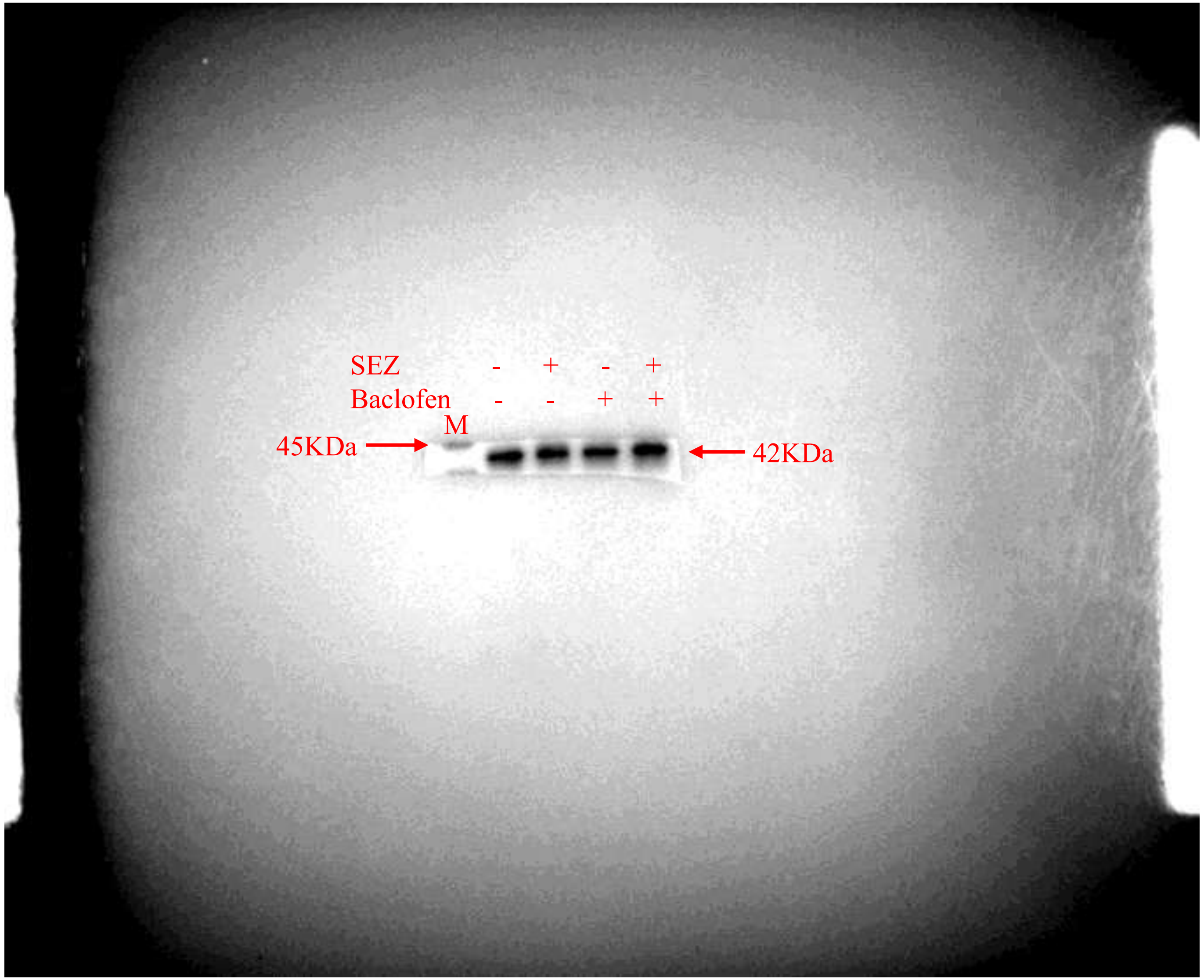

Figure 2 N β-actin (repeated 3)

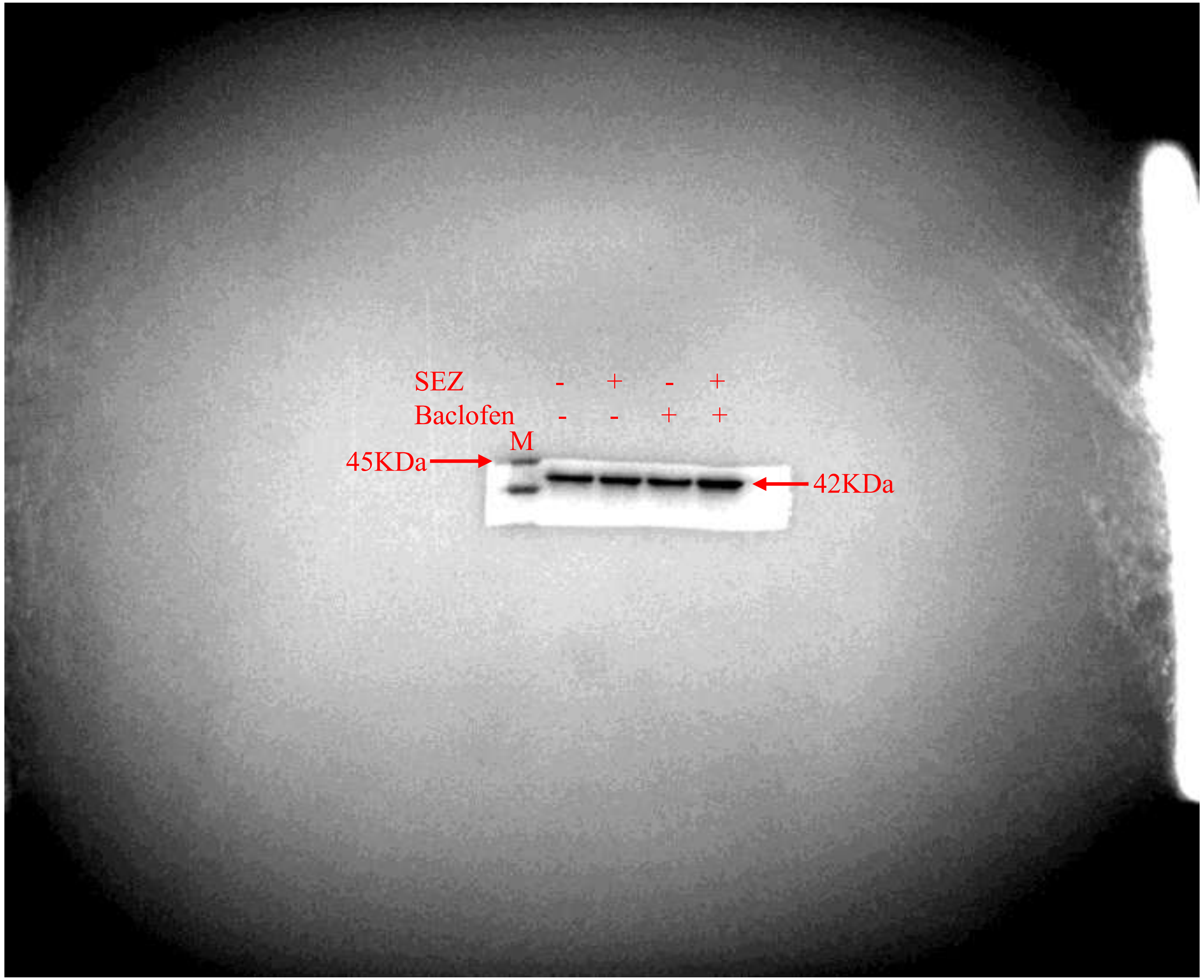

Figure 4 Q GPX4 (repeated 1)

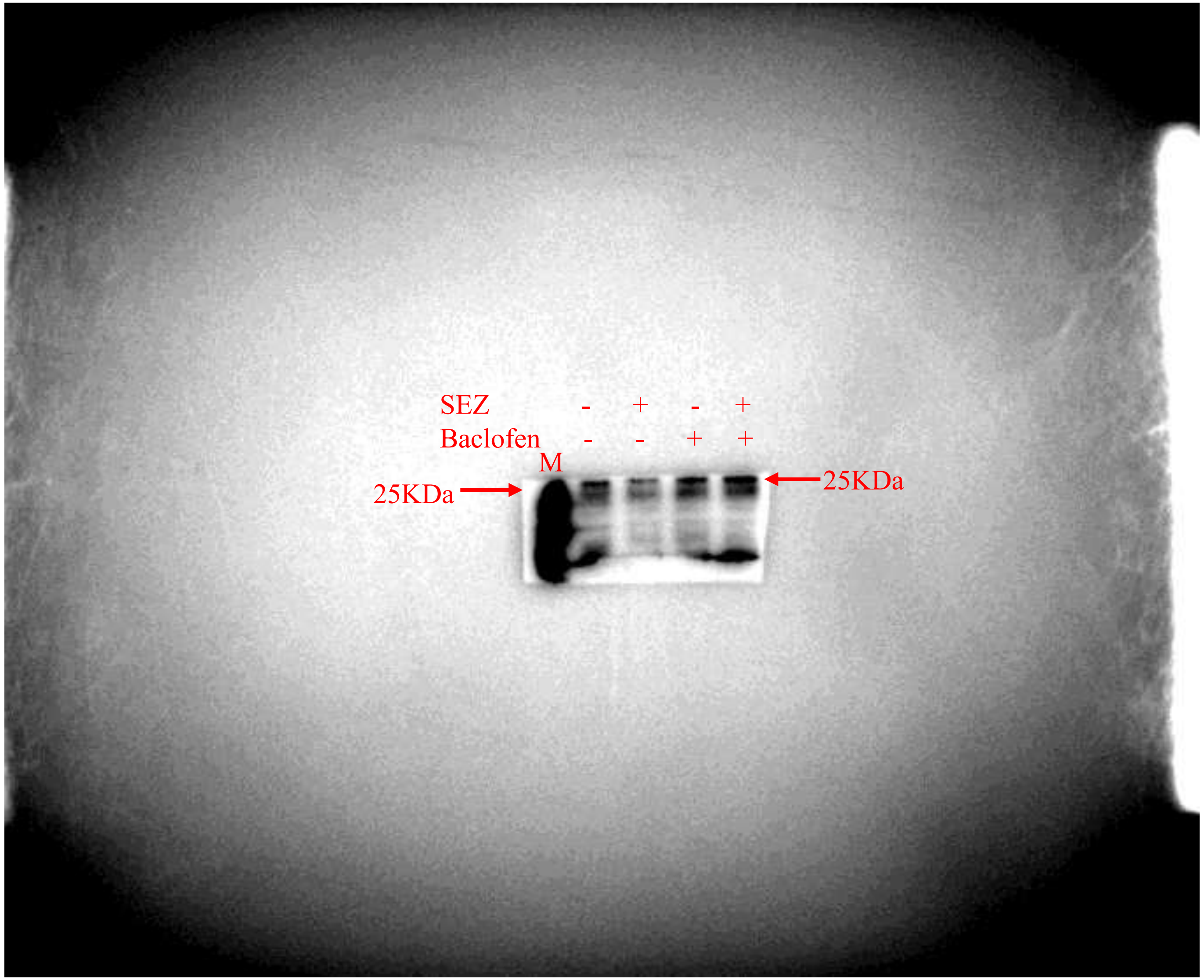

Figure 4 Q GPX4 (repeated 2)

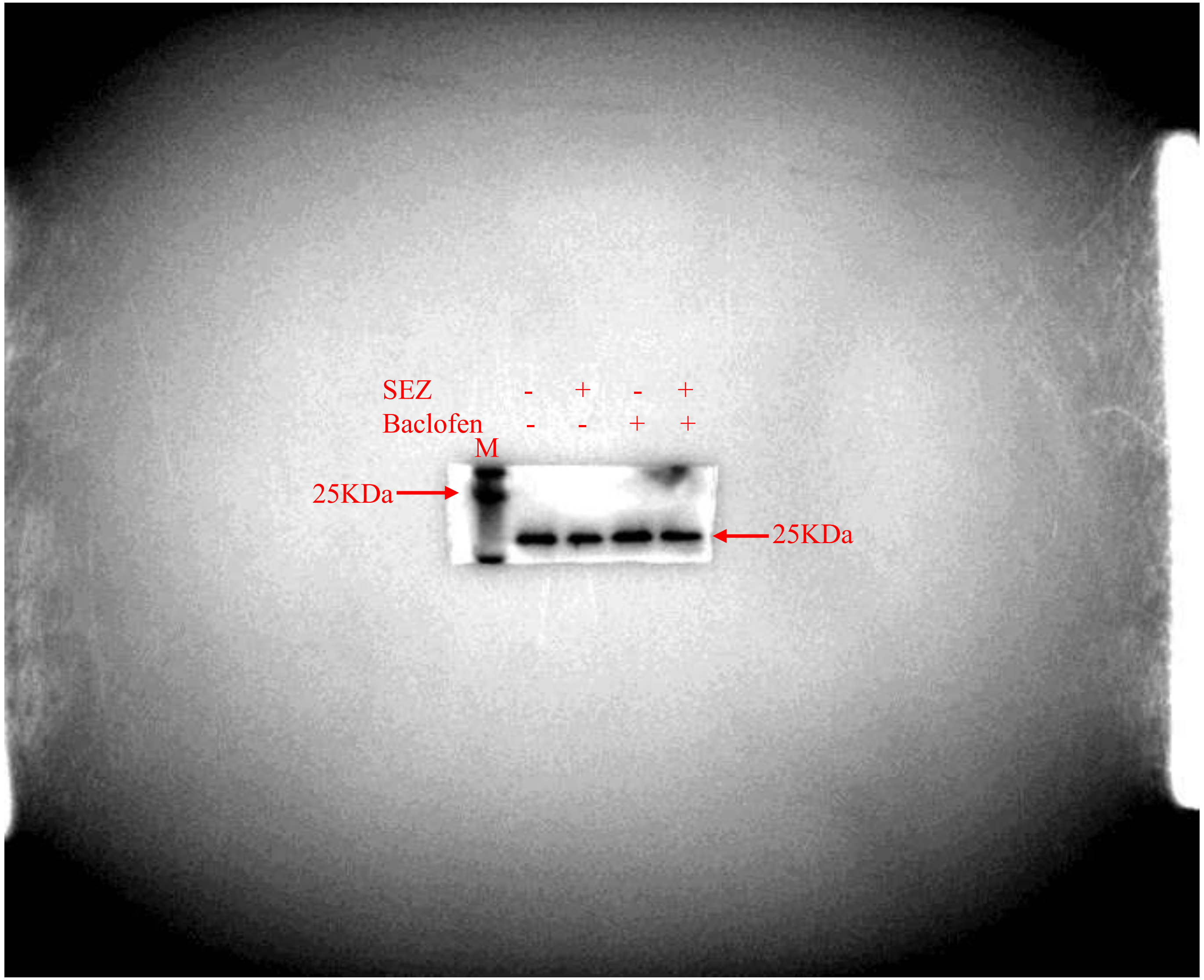

Figure 4 Q GPX4 (repeated 3)

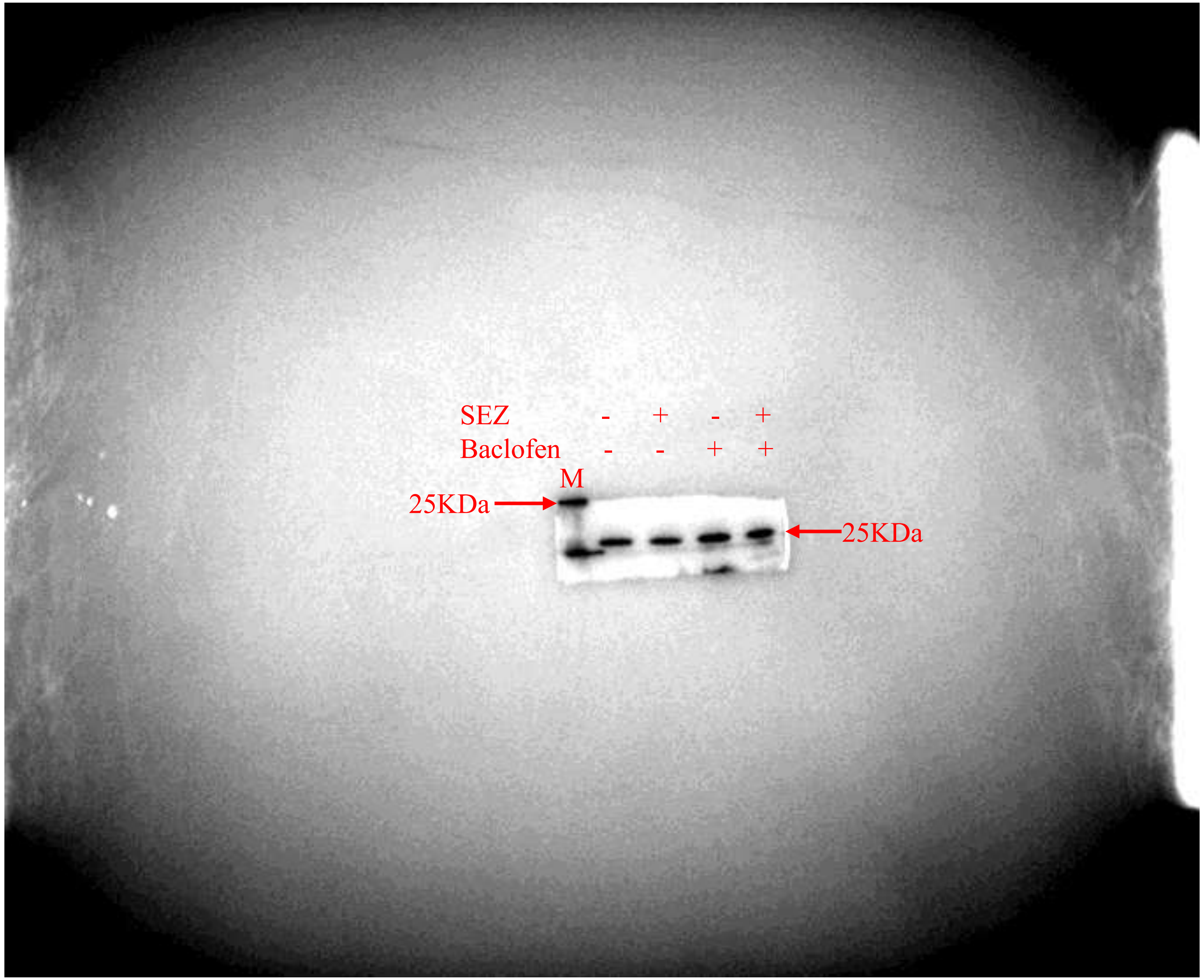

Figure 4 Q HO-1 (repeated 1)

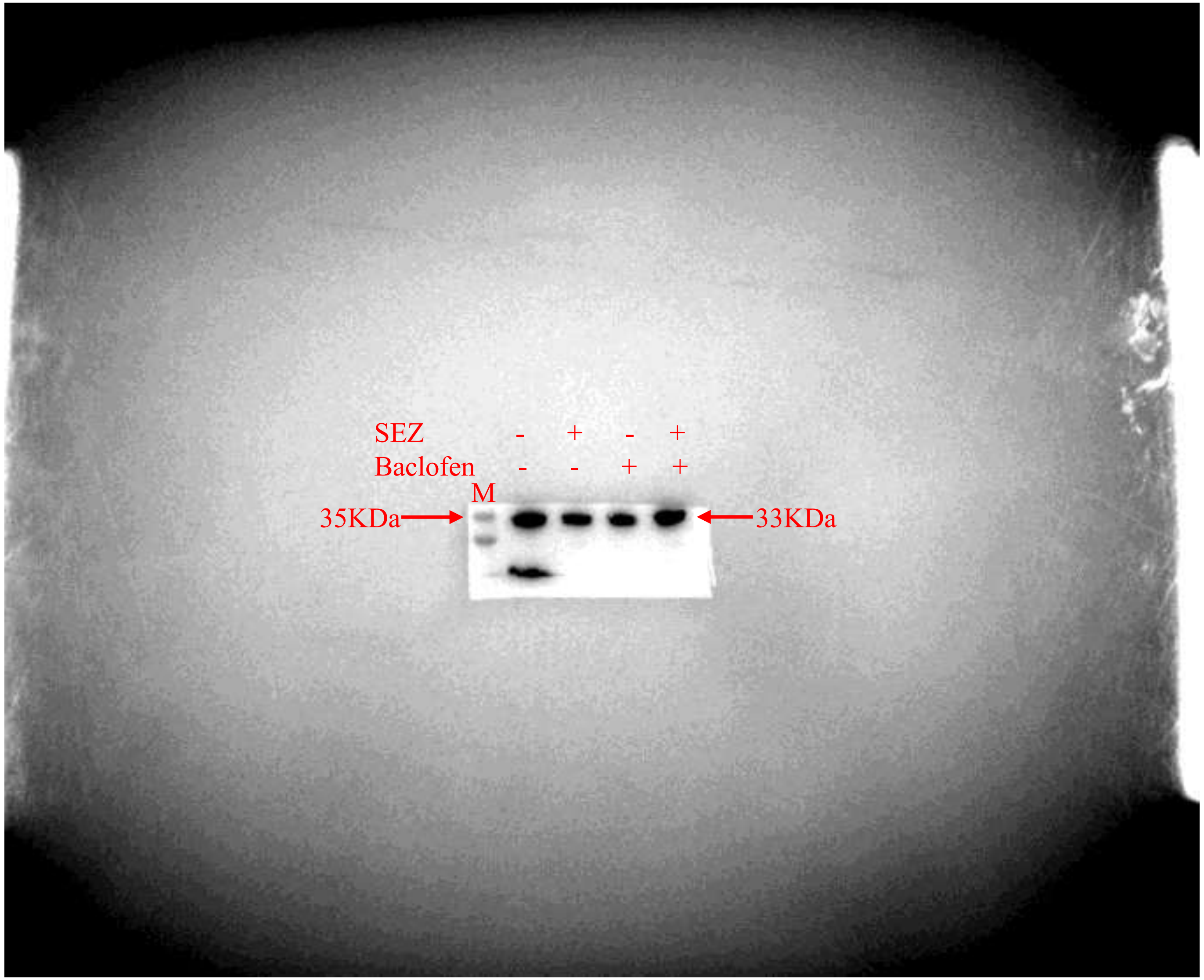

Figure 4 Q HO-1 (repeated 2)

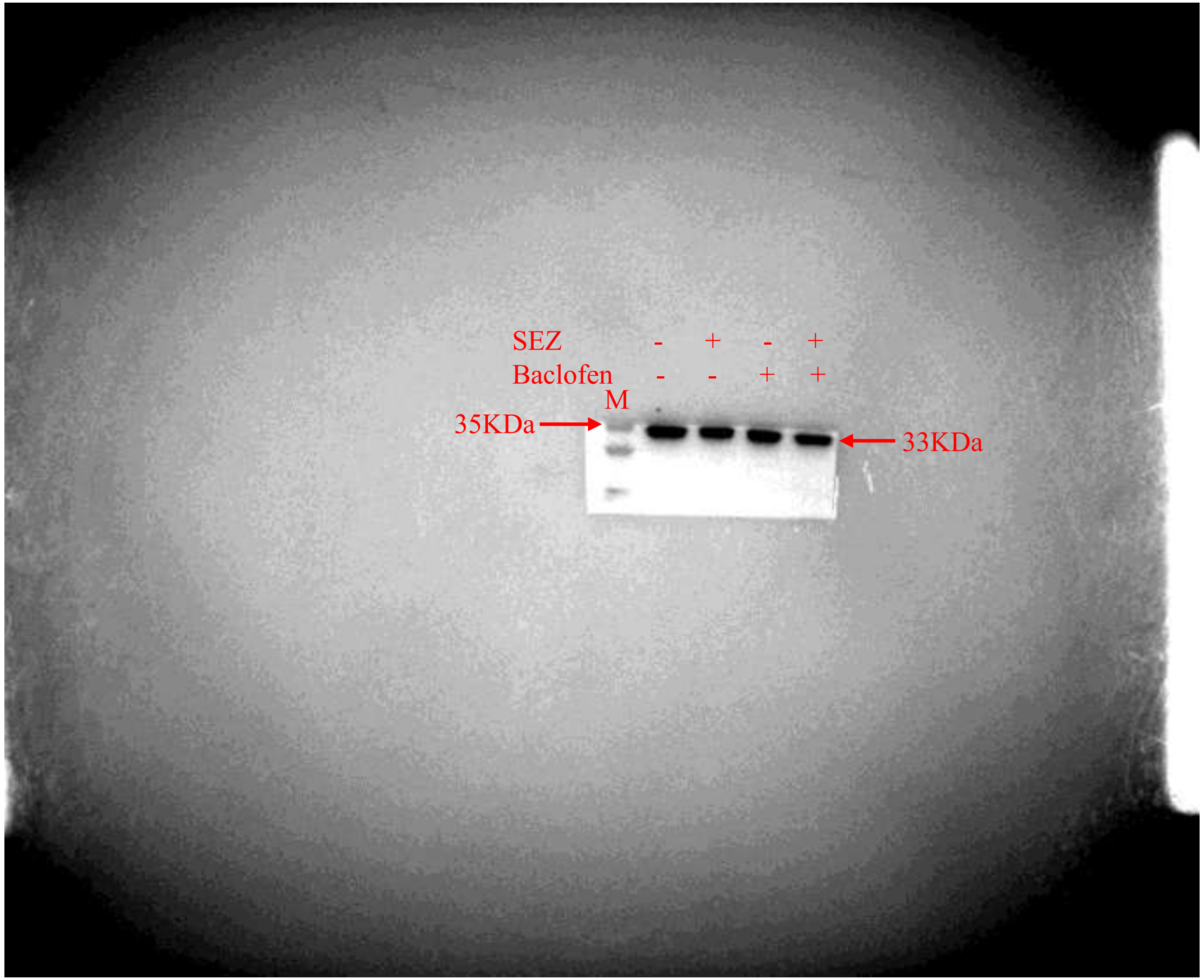

Figure 4 Q HO-1 (repeated 3)

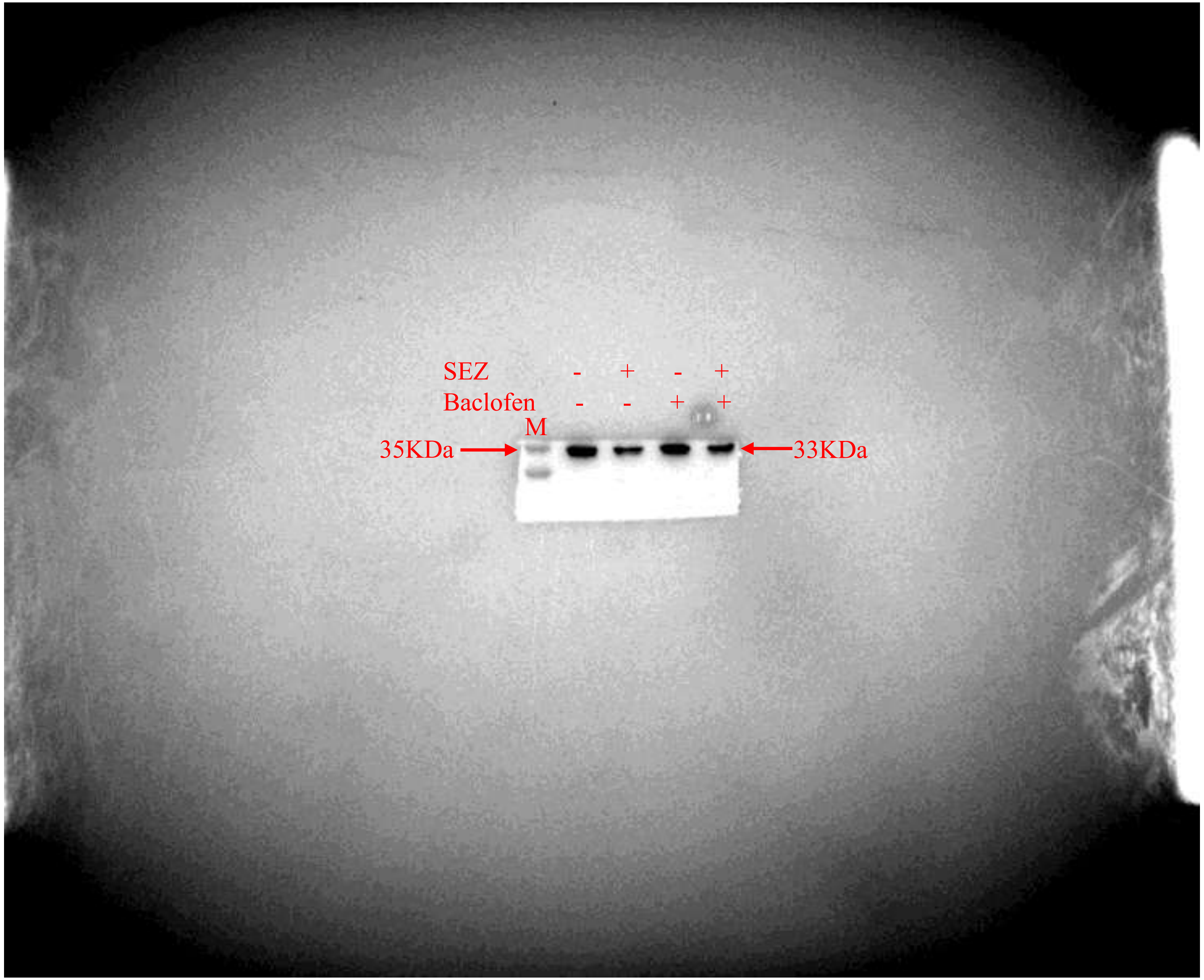

Figure 4 Q iNOS (repeated 1)

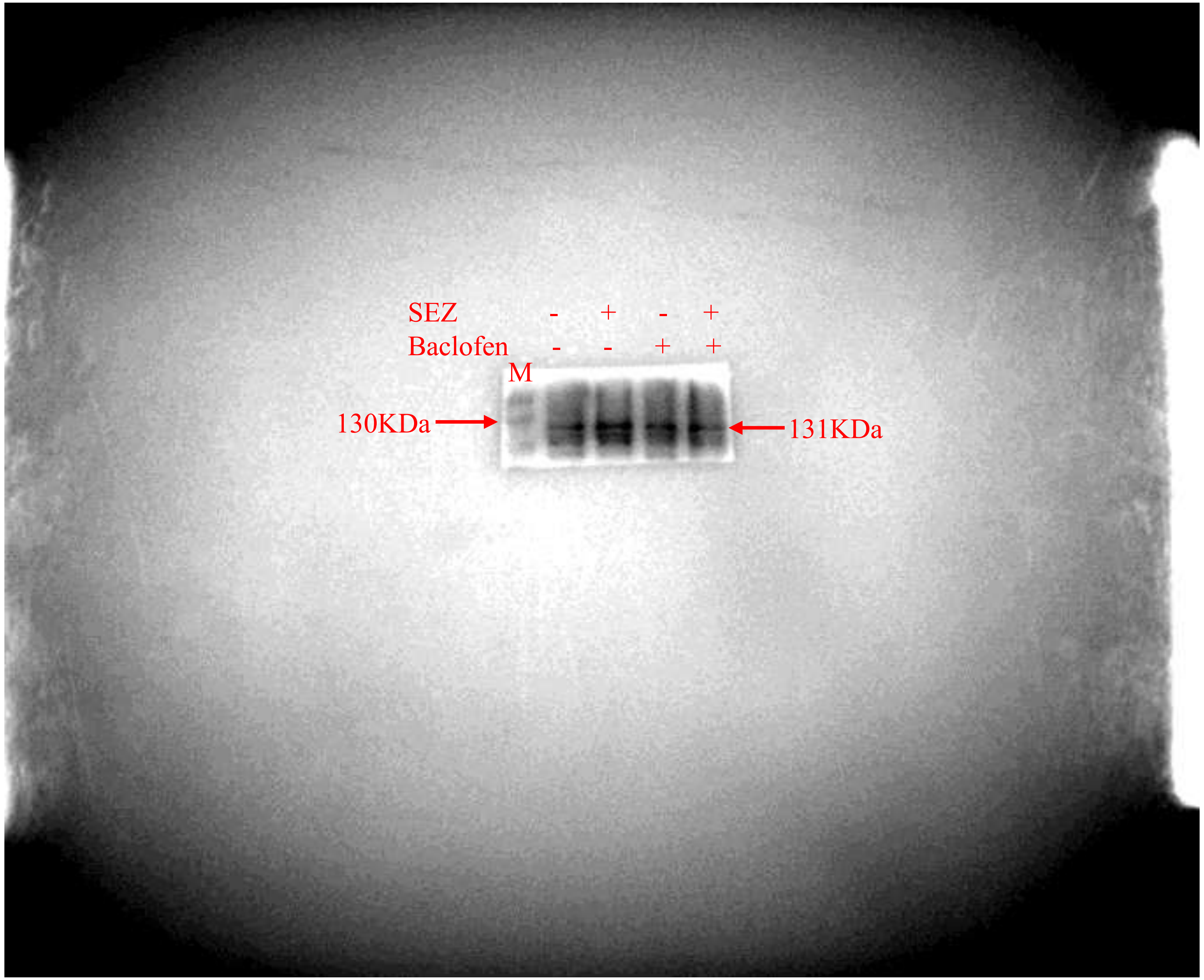

Figure 4 Q iNOS (repeated 2)

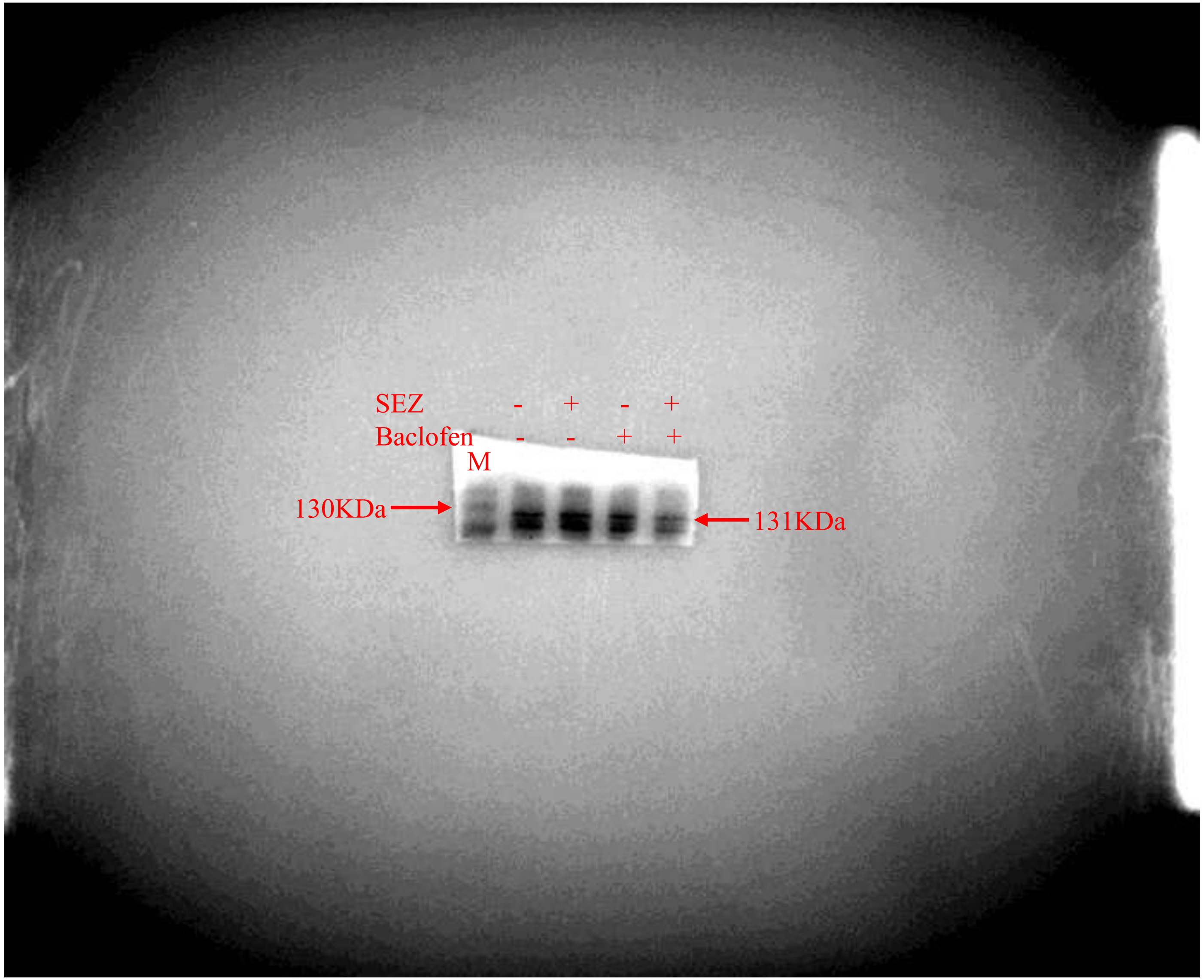

Figure 4 Q iNOS (repeated 3)

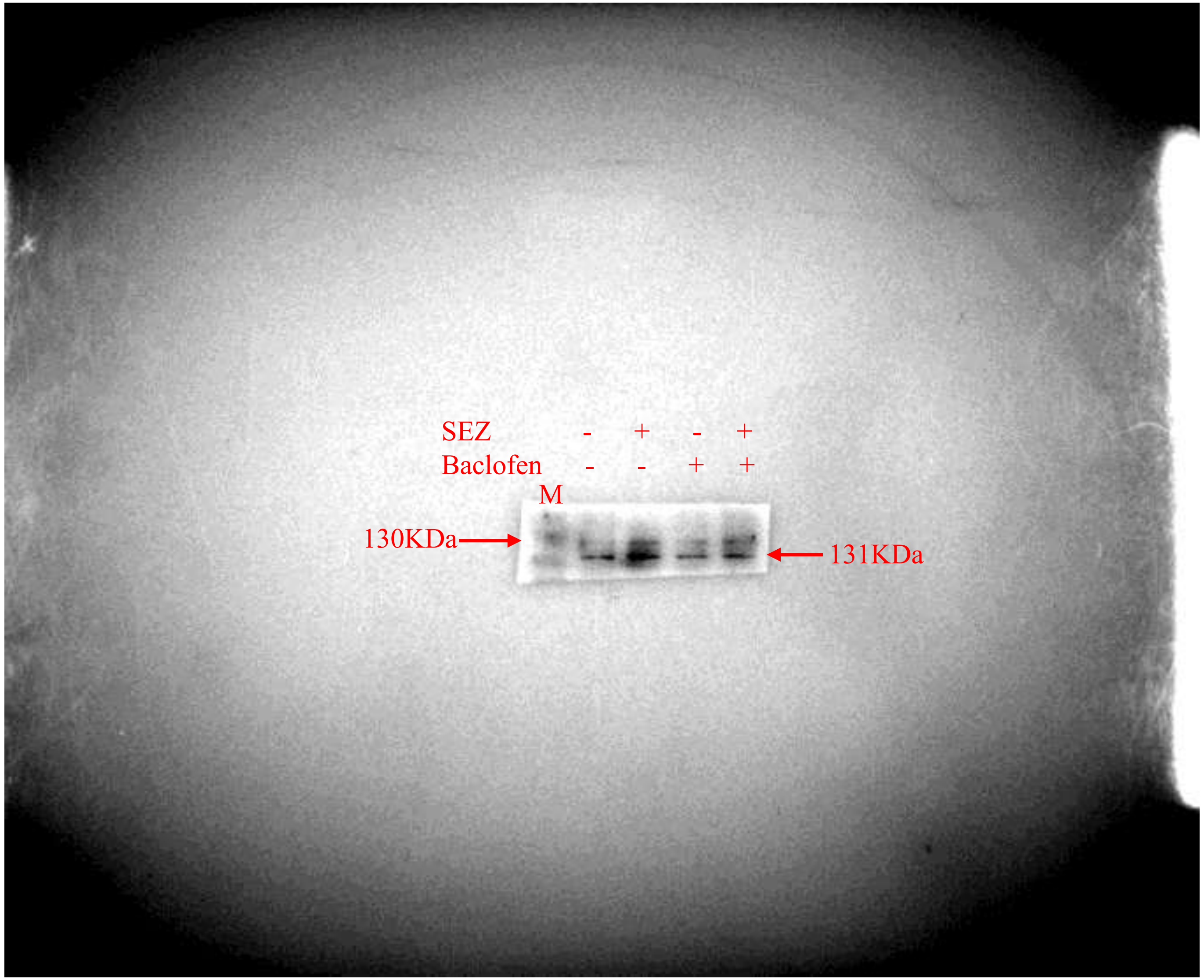

Figure 4 Q NRF2 (repeated 1)

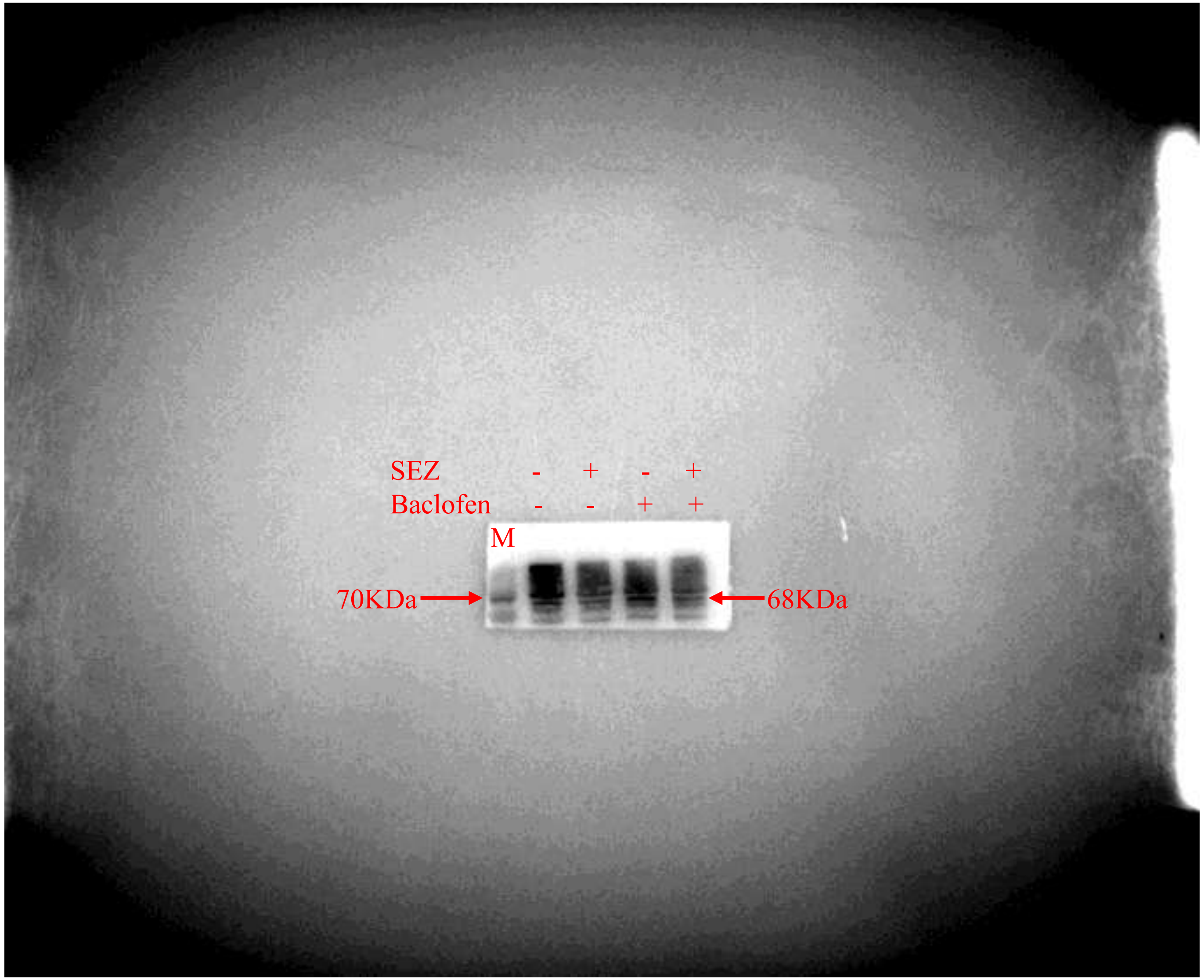

Figure 4 Q NRF2 (repeated 2)

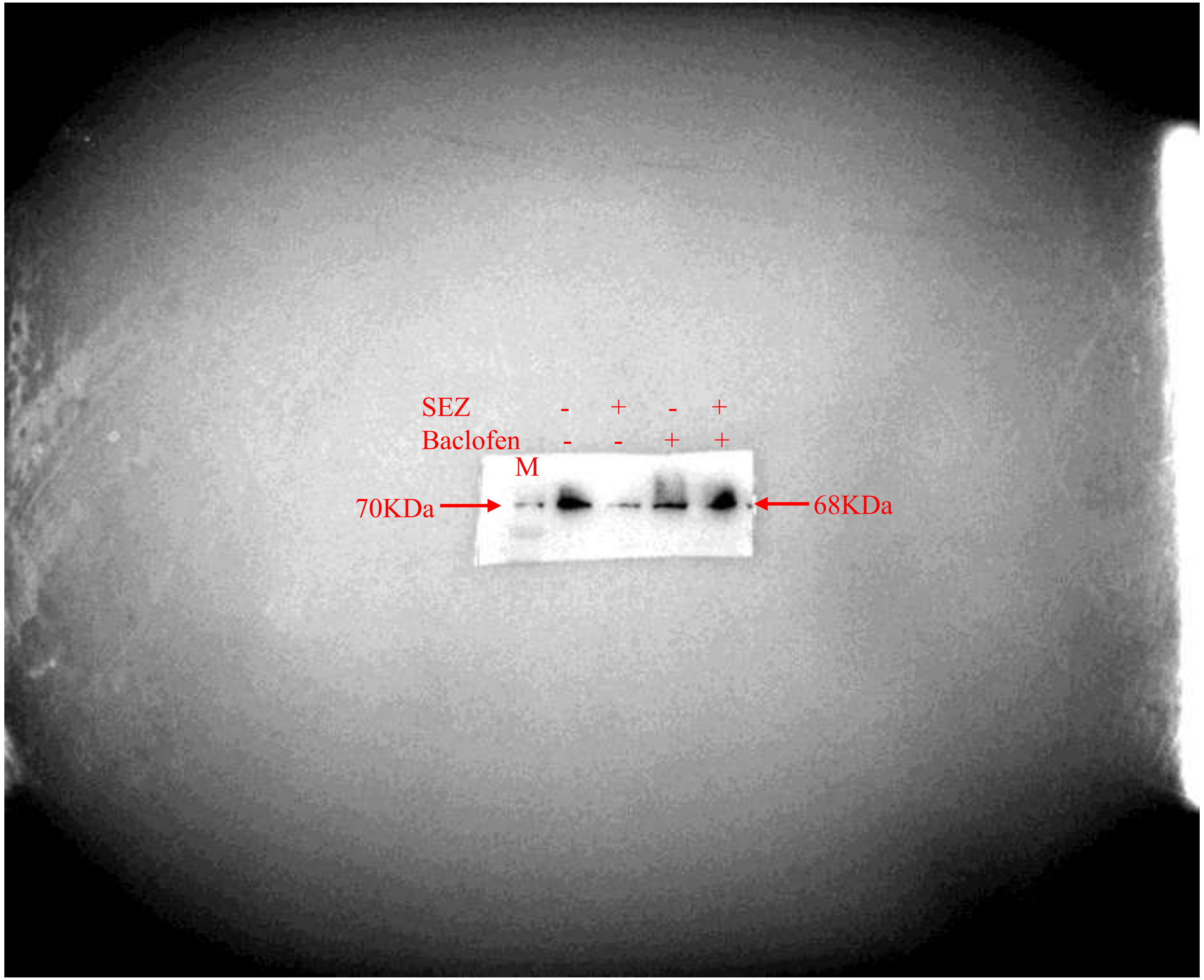

Figure 4 Q NRF2 (repeated 3)

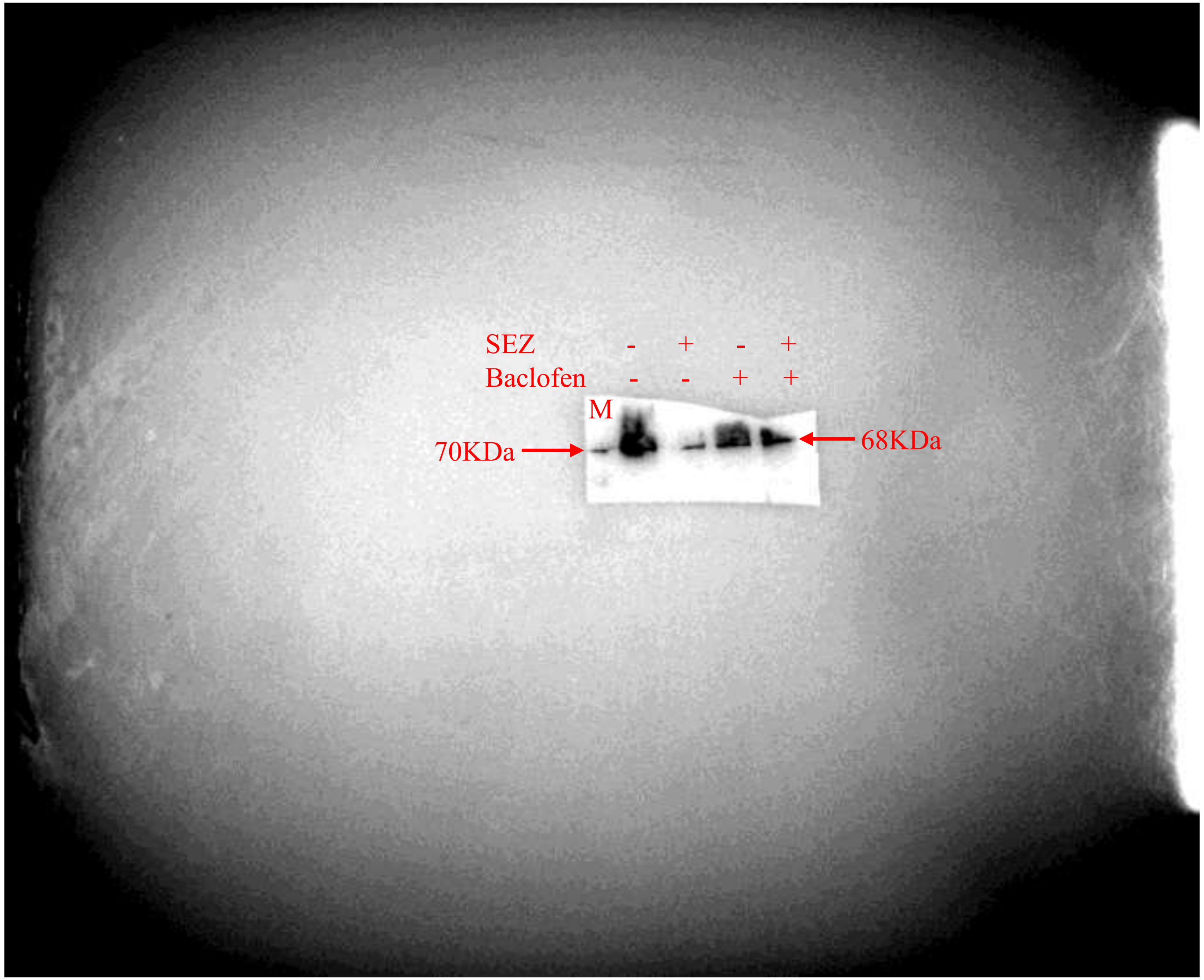

Figure 4 Q β-actin (repeated 1)

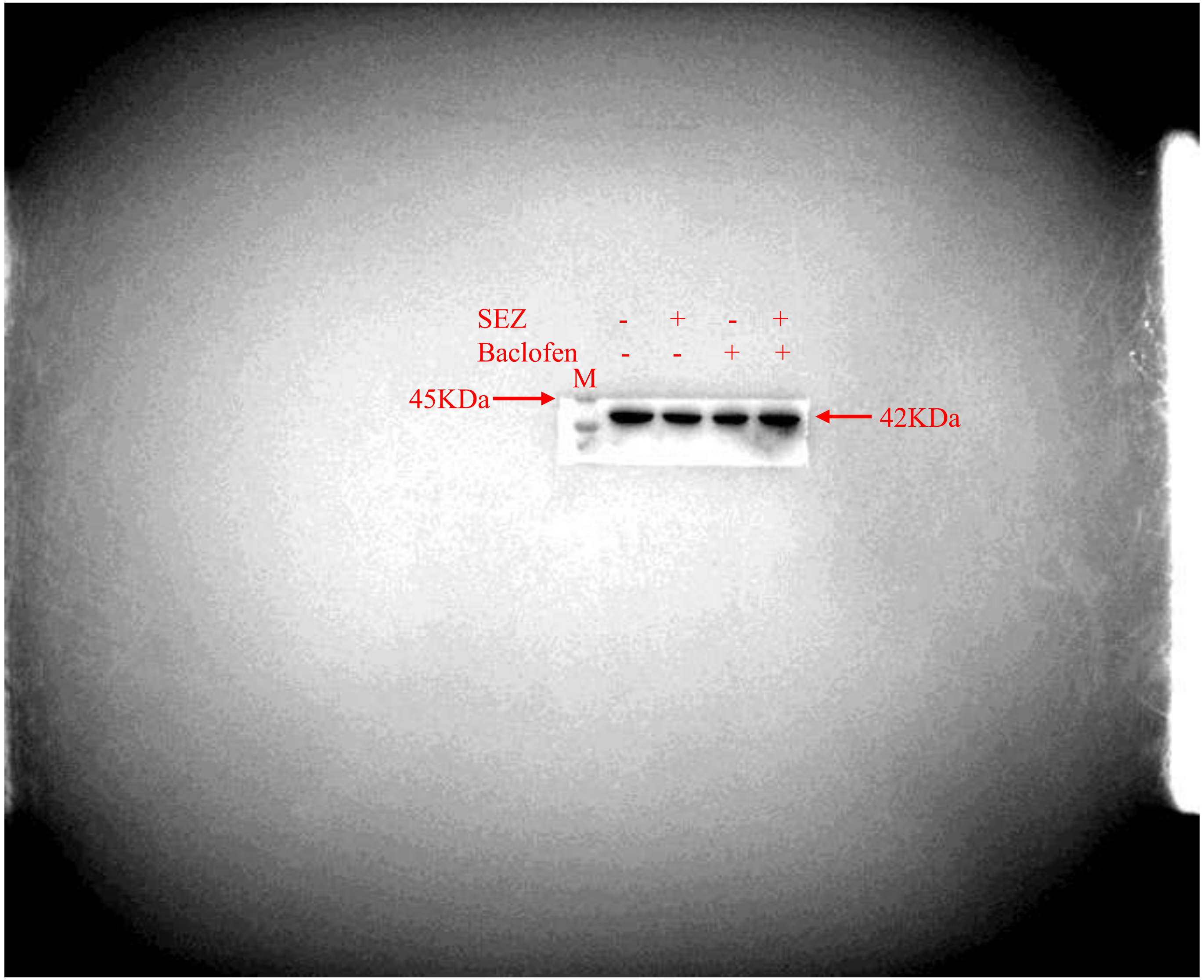

Figure 4 Q β-actin (repeated 2)

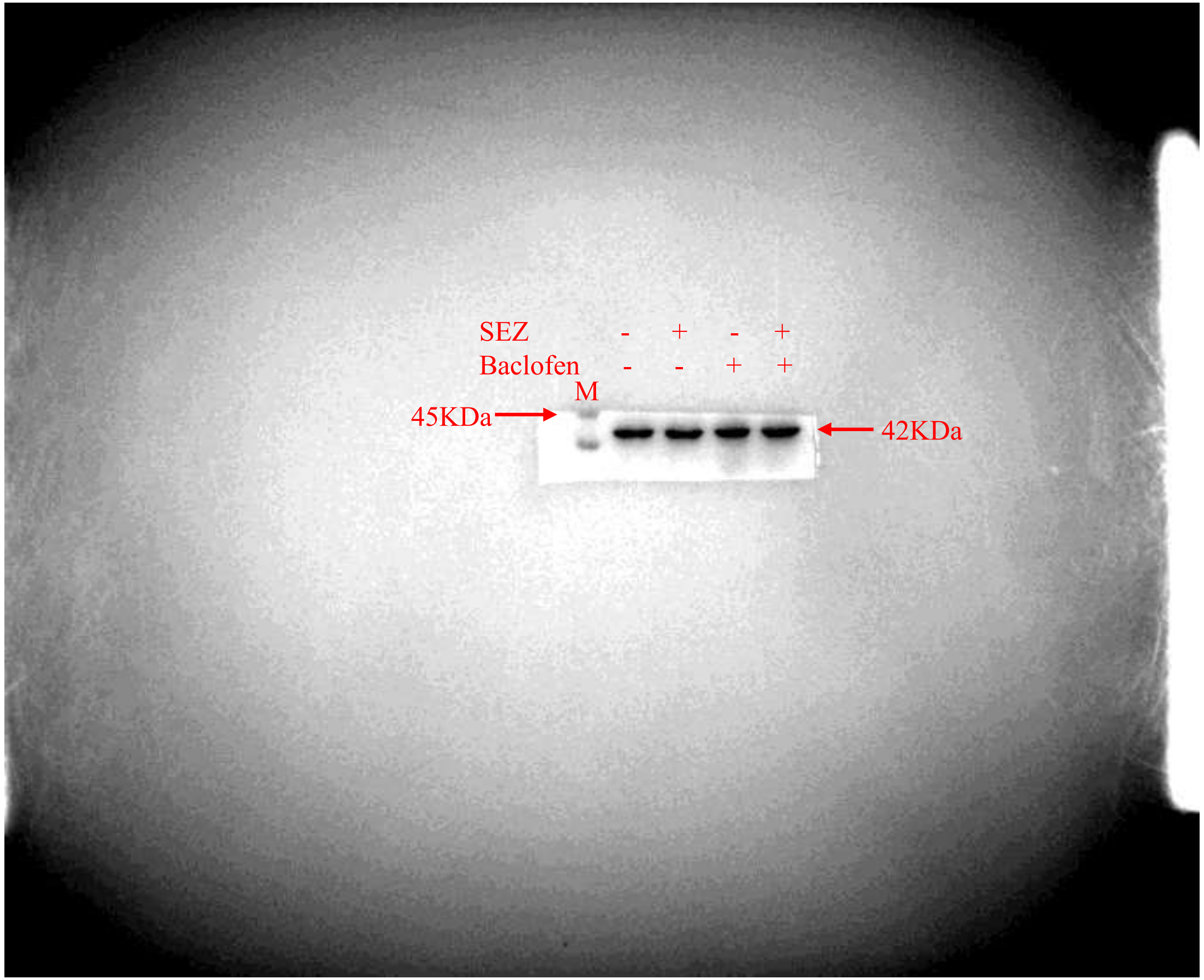

Figure 4 Q β-actin (repeated 3)

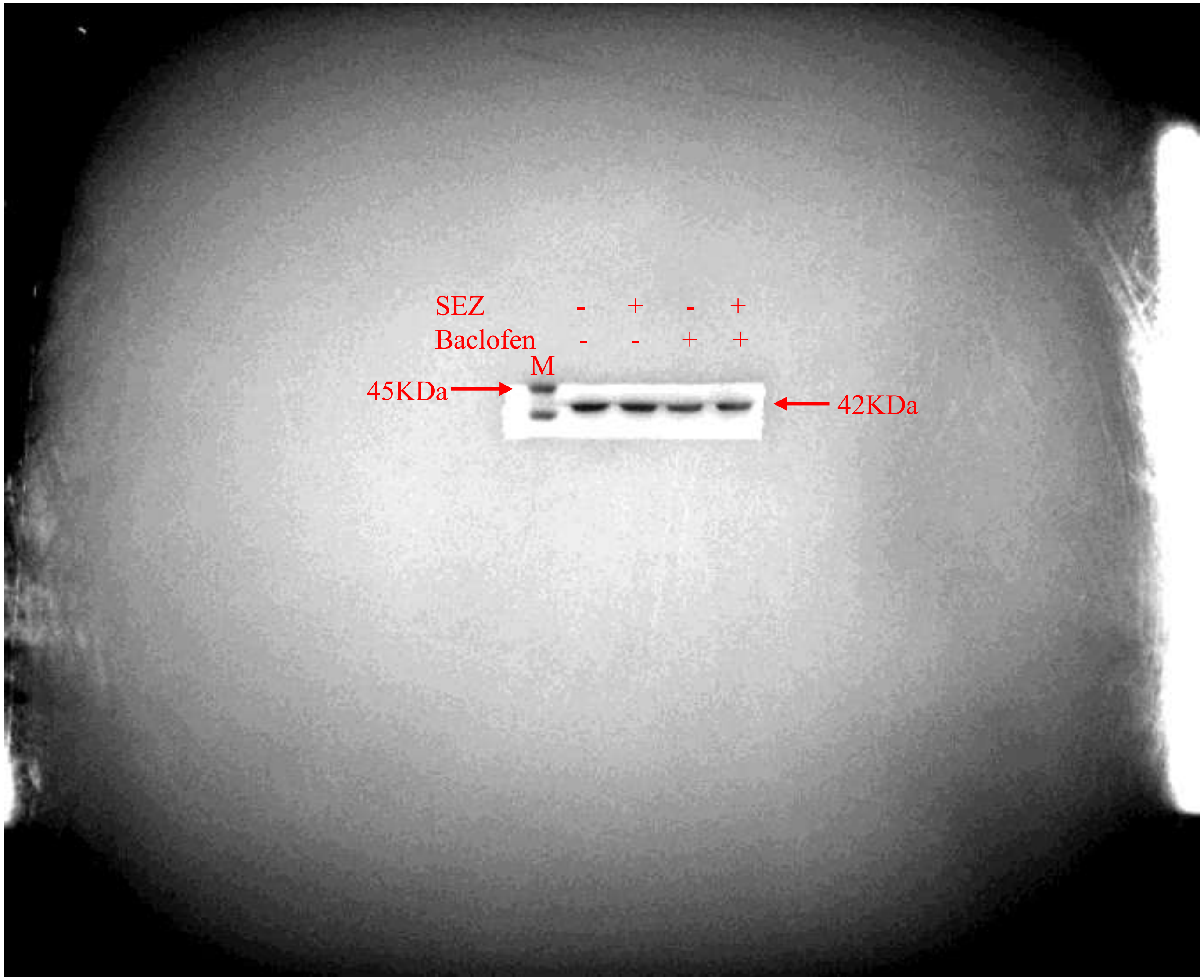

Figure 5 I IRAK-M (repeated 1)

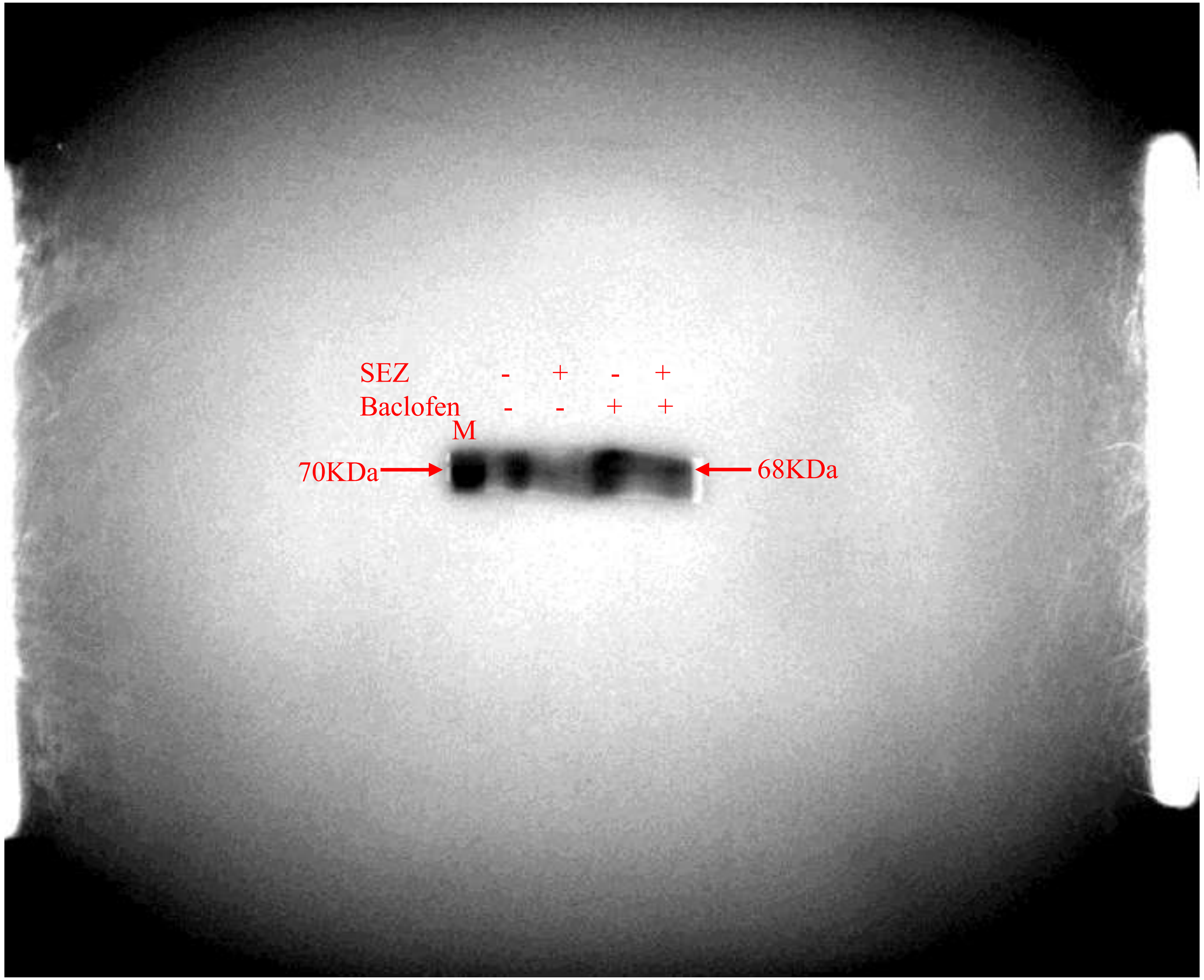

Figure 5 I IRAK-M (repeated 2)

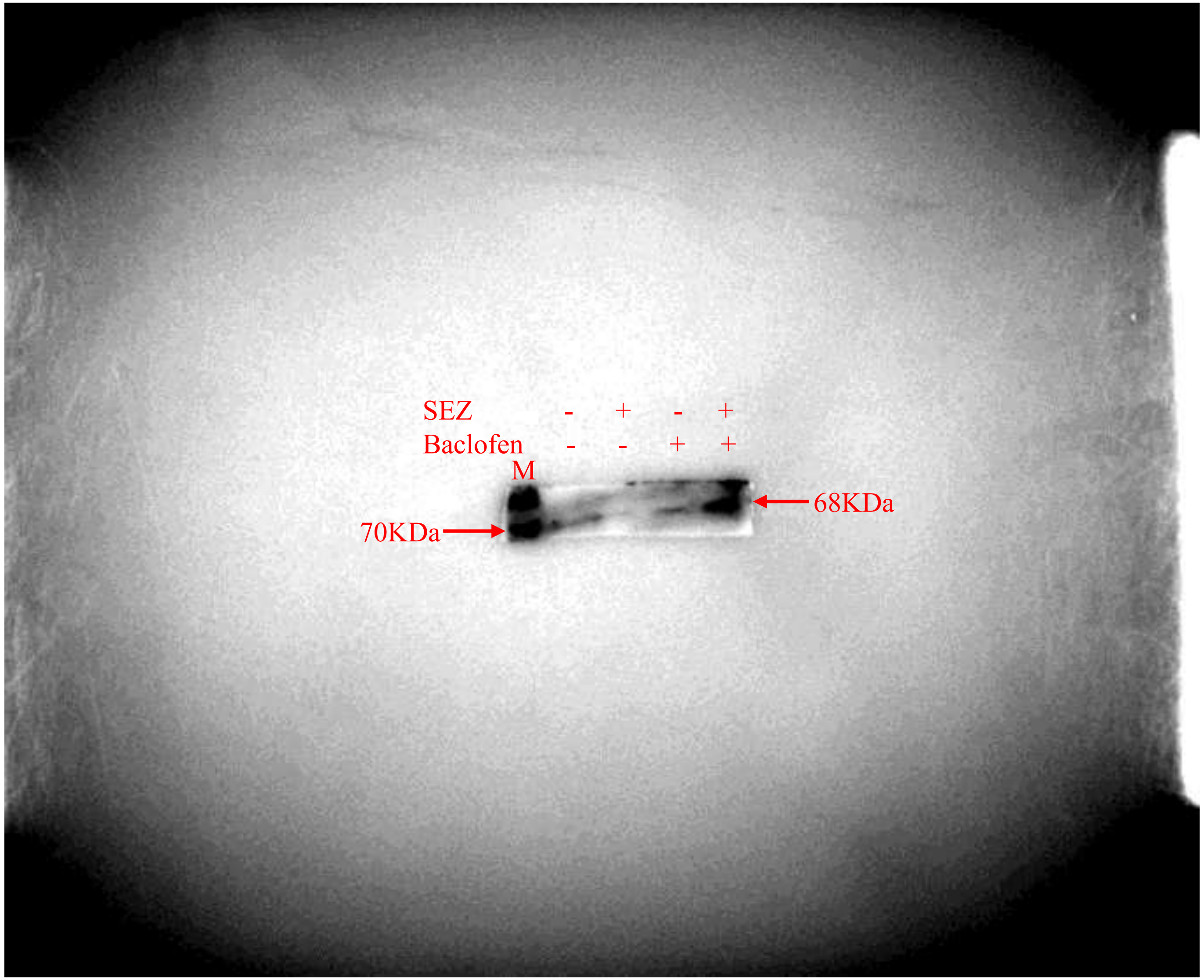

Figure 5 I IRAK-M (repeated 3)

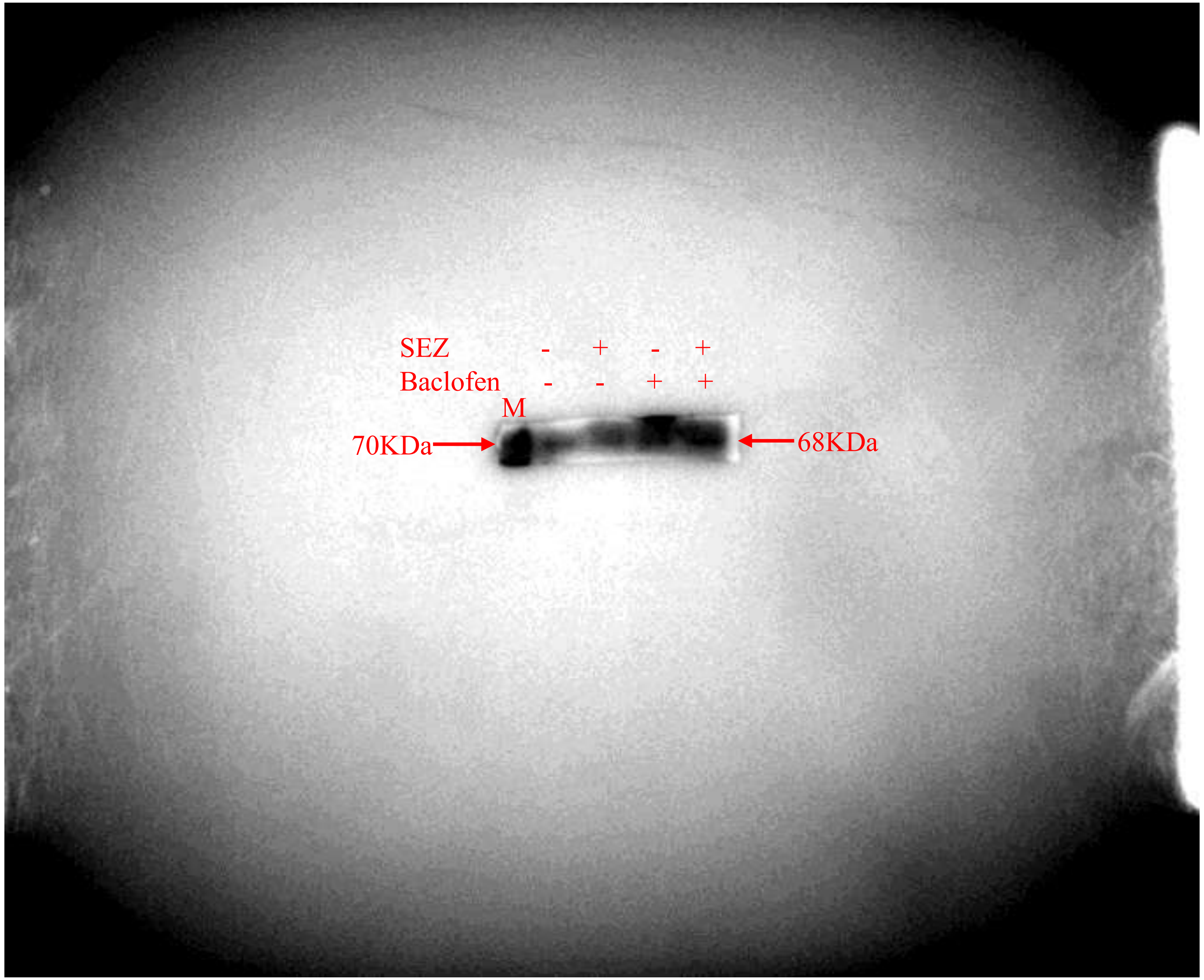

Figure 5 I NLRP3 (repeated 1)

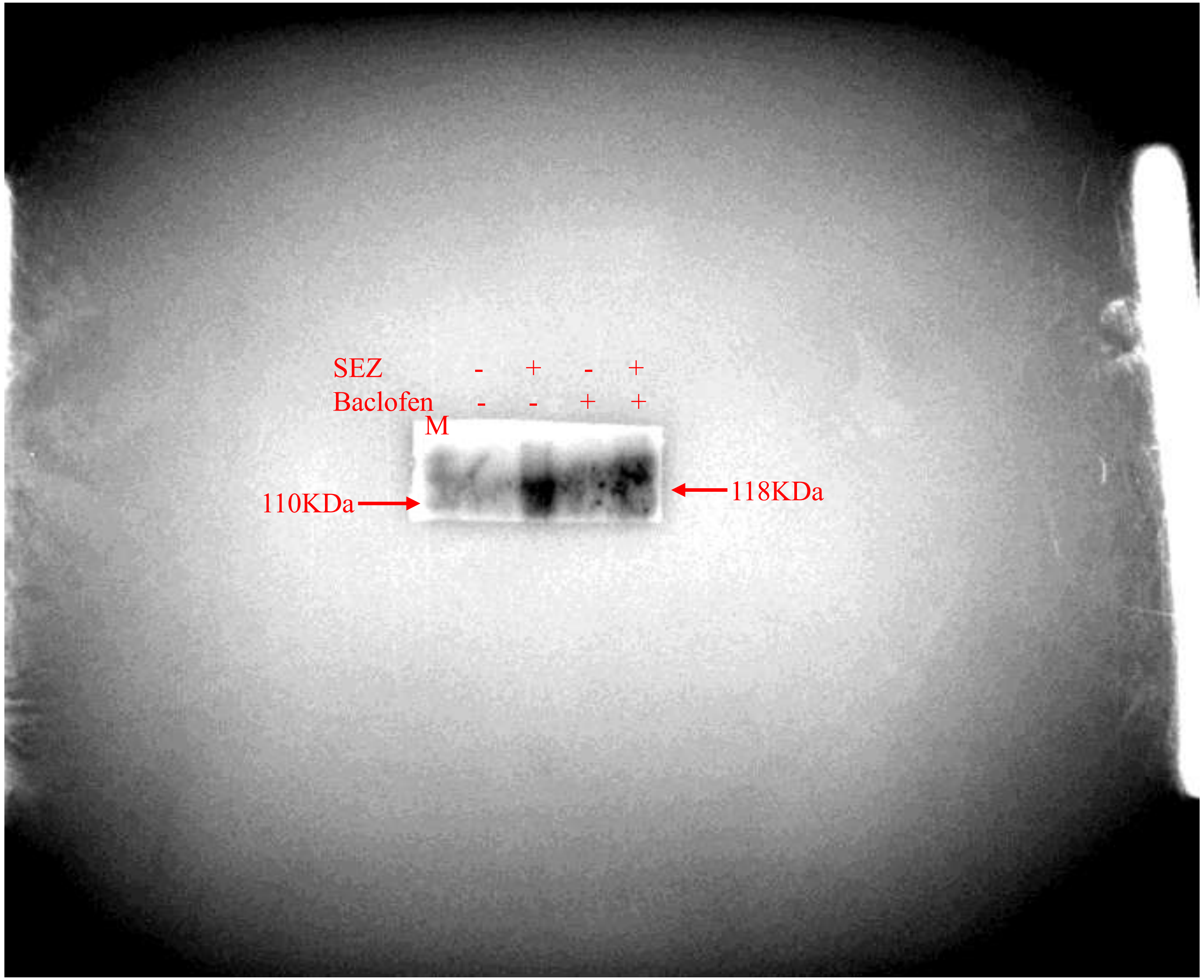

Figure 5 I NLRP3 (repeated 2)

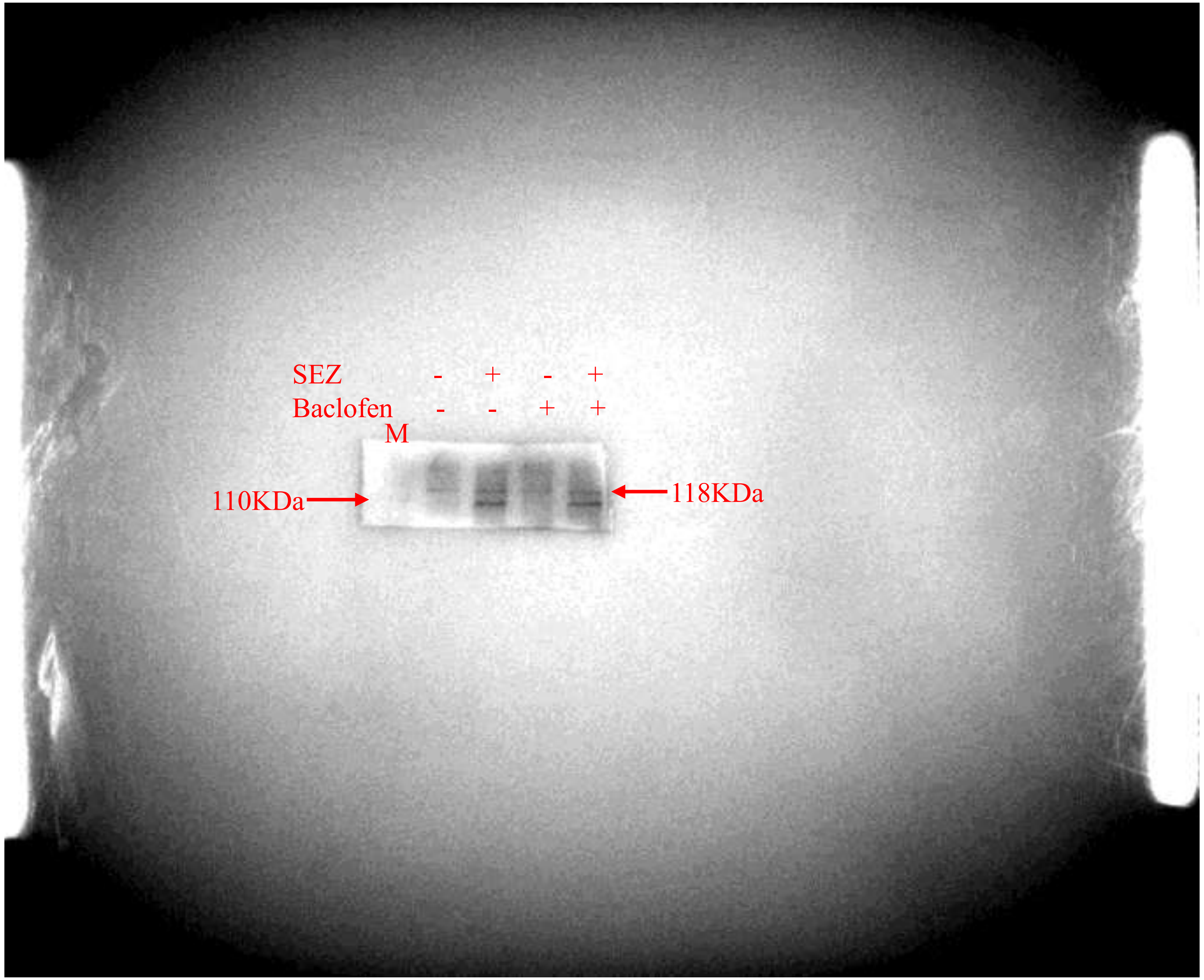

Figure 5 I NLRP3 (repeated 3)

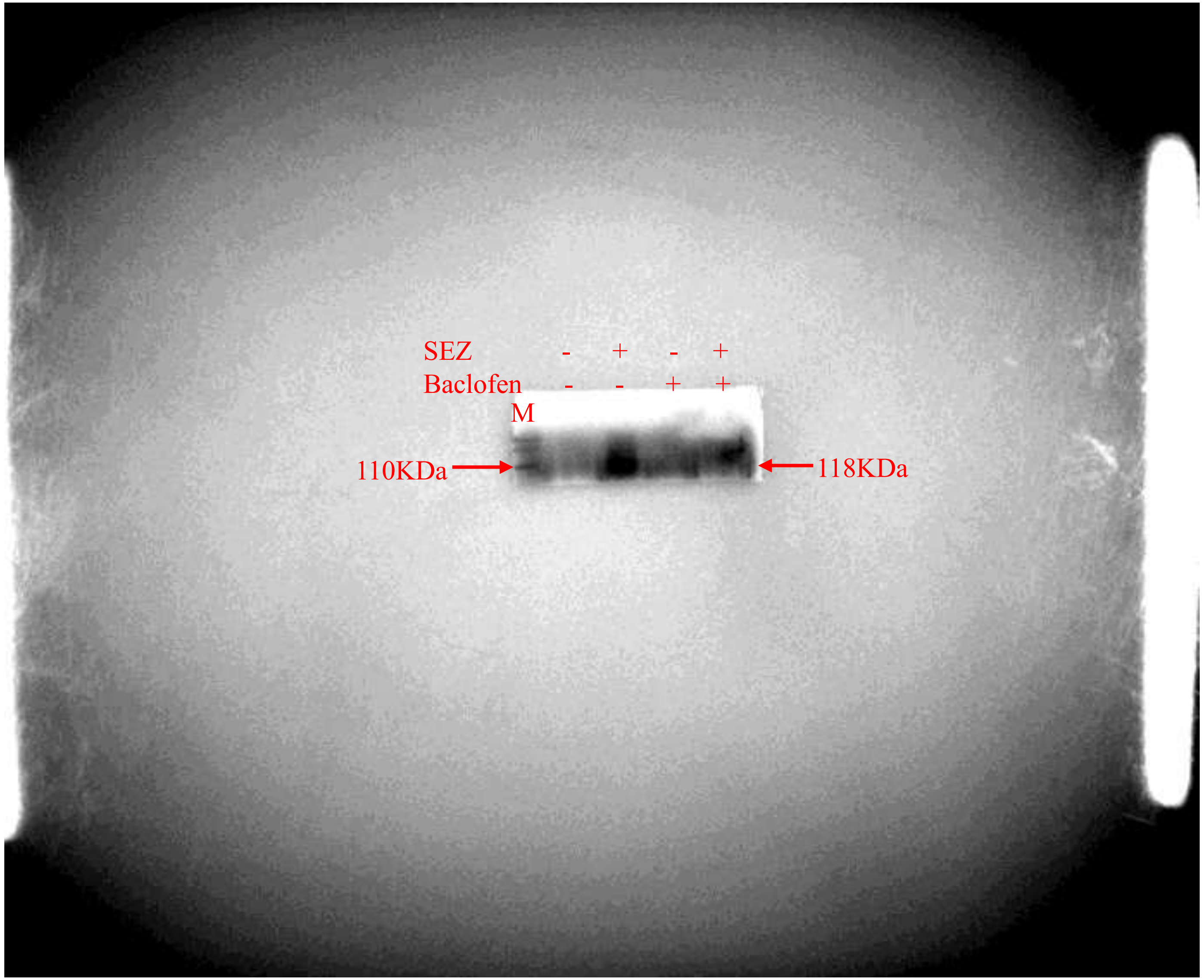

Figure 5 I caspase-1 (repeated 1)

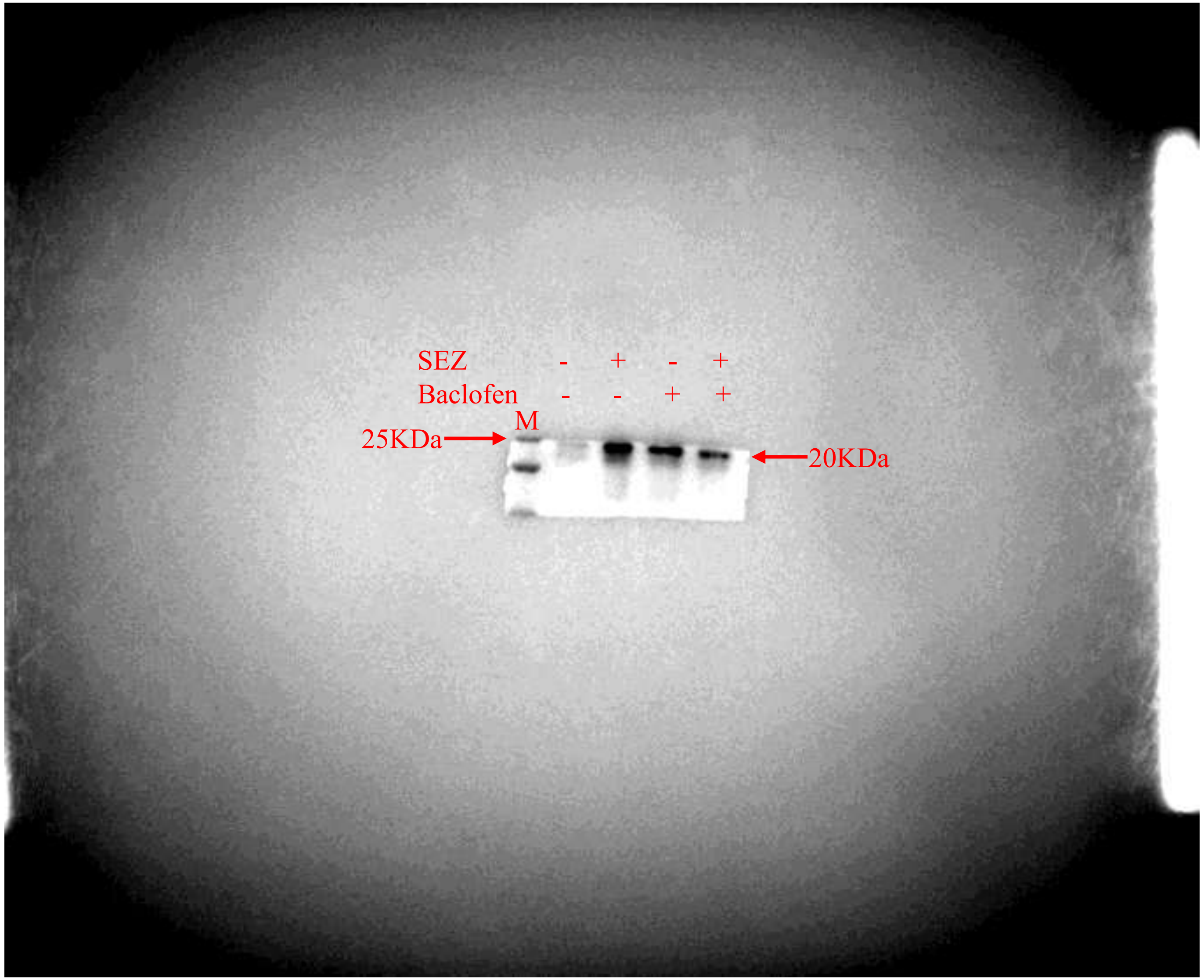

Figure 5 I caspase-1 (repeated 2)

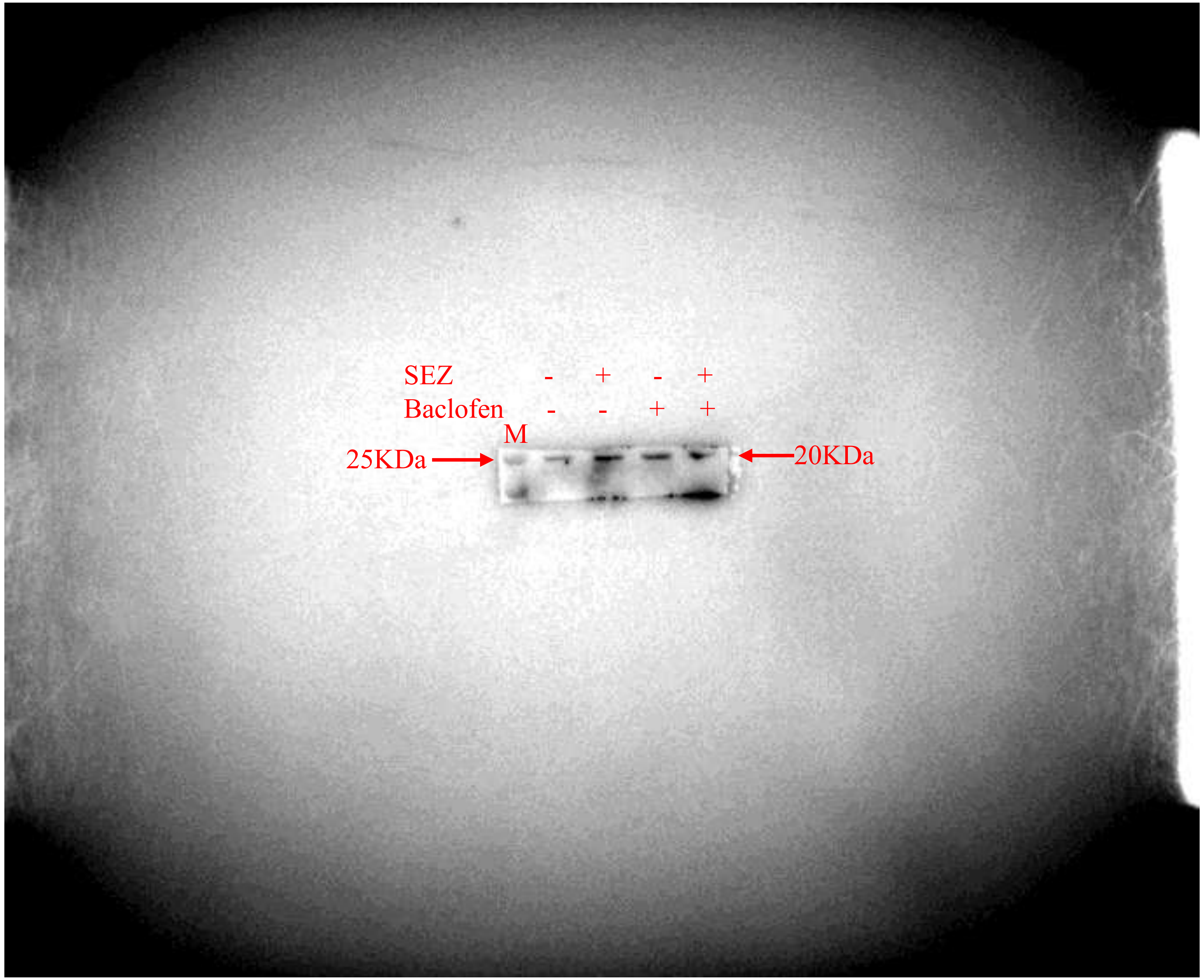

Figure 5 I caspase-1 (repeated 3)

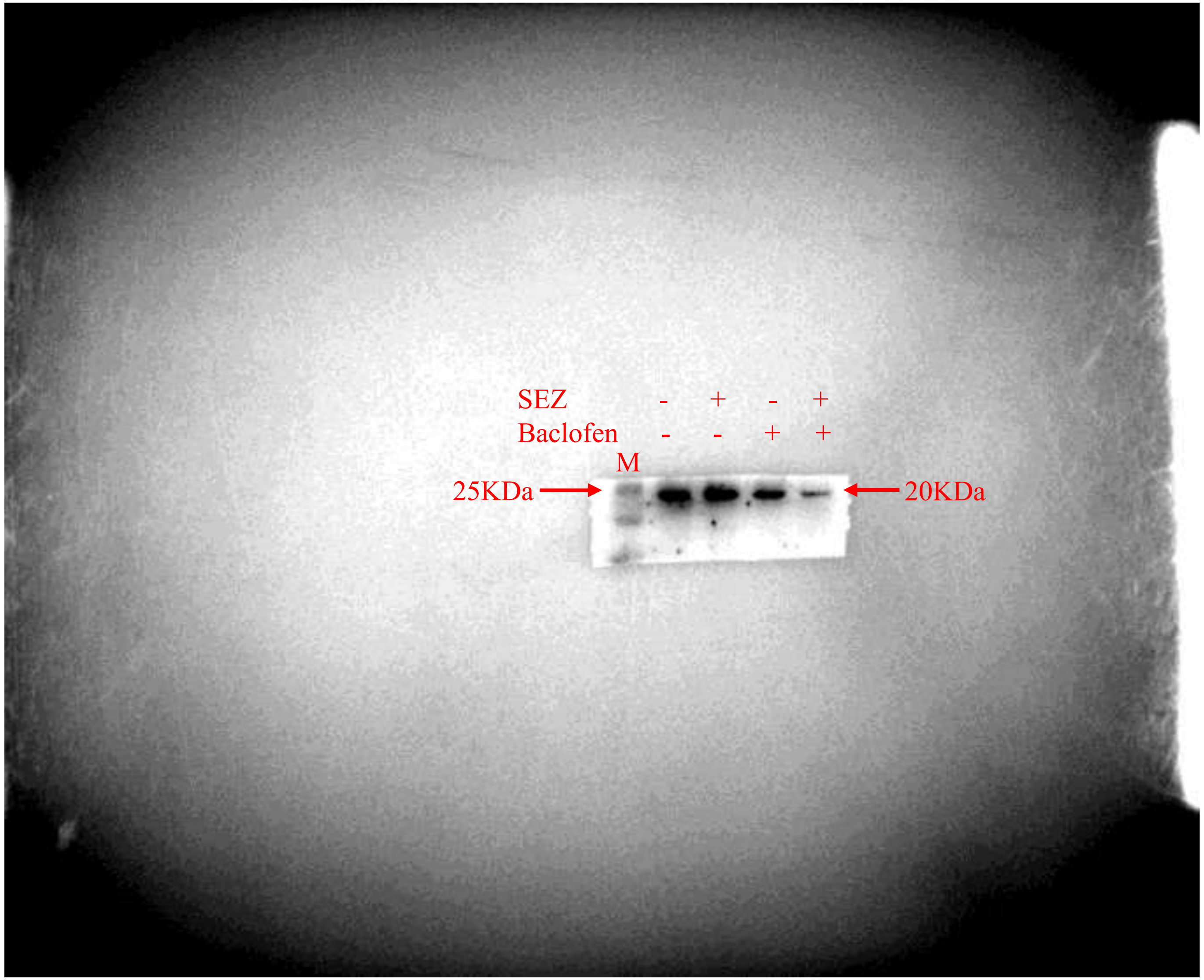

Figure 5 I GSDMD (repeated 1)

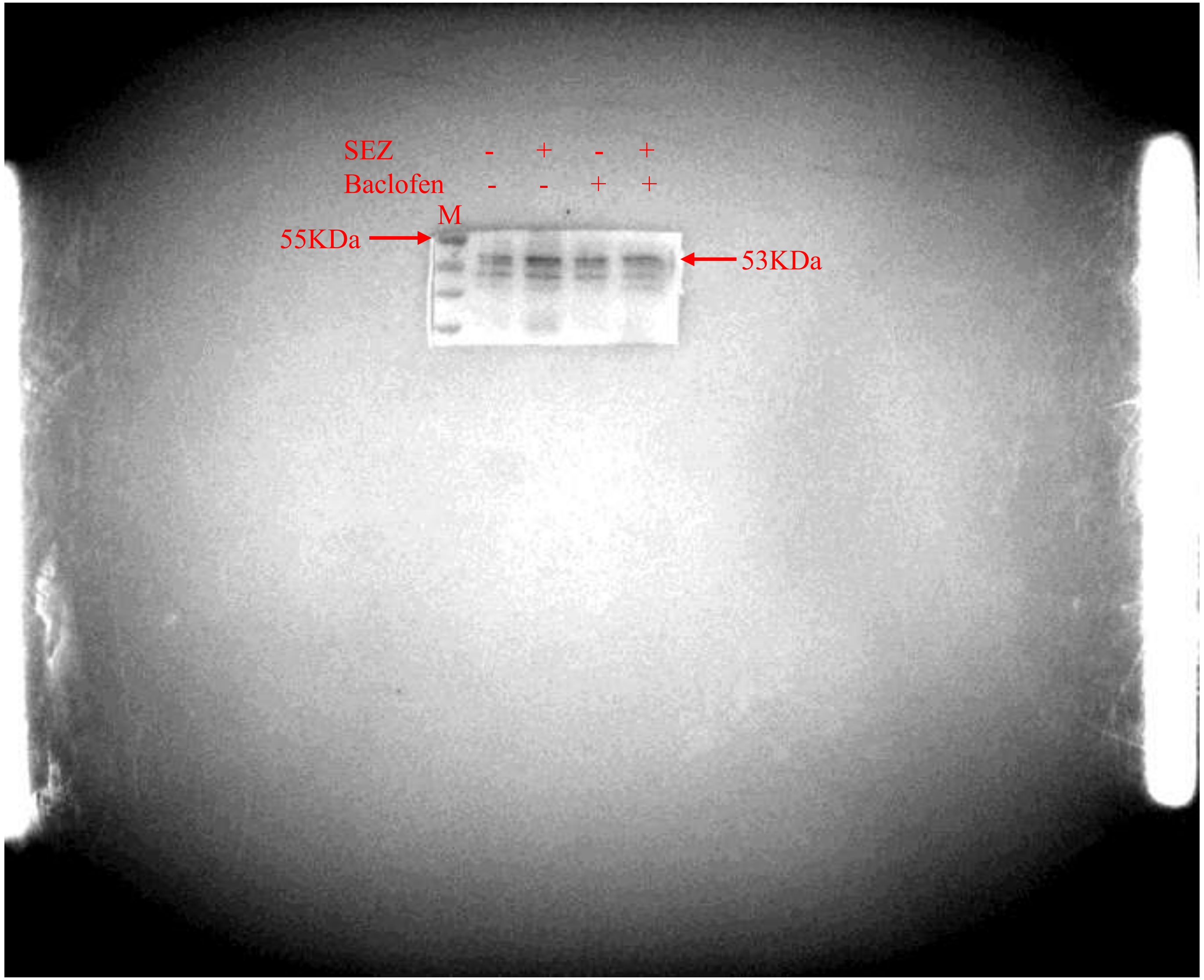

Figure 5 I GSDMD (repeated 2)

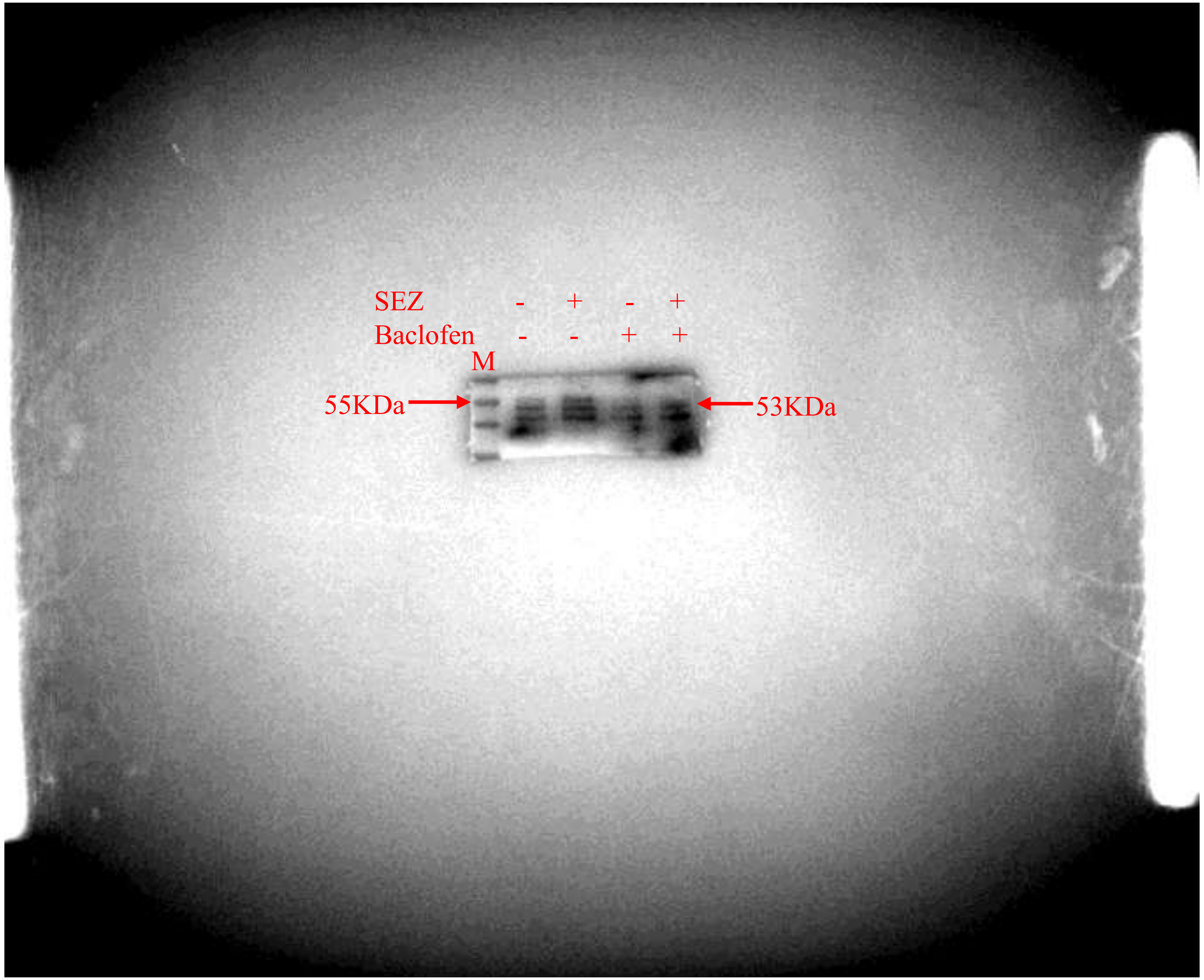

Figure 5 I GSDMD (repeated 3)

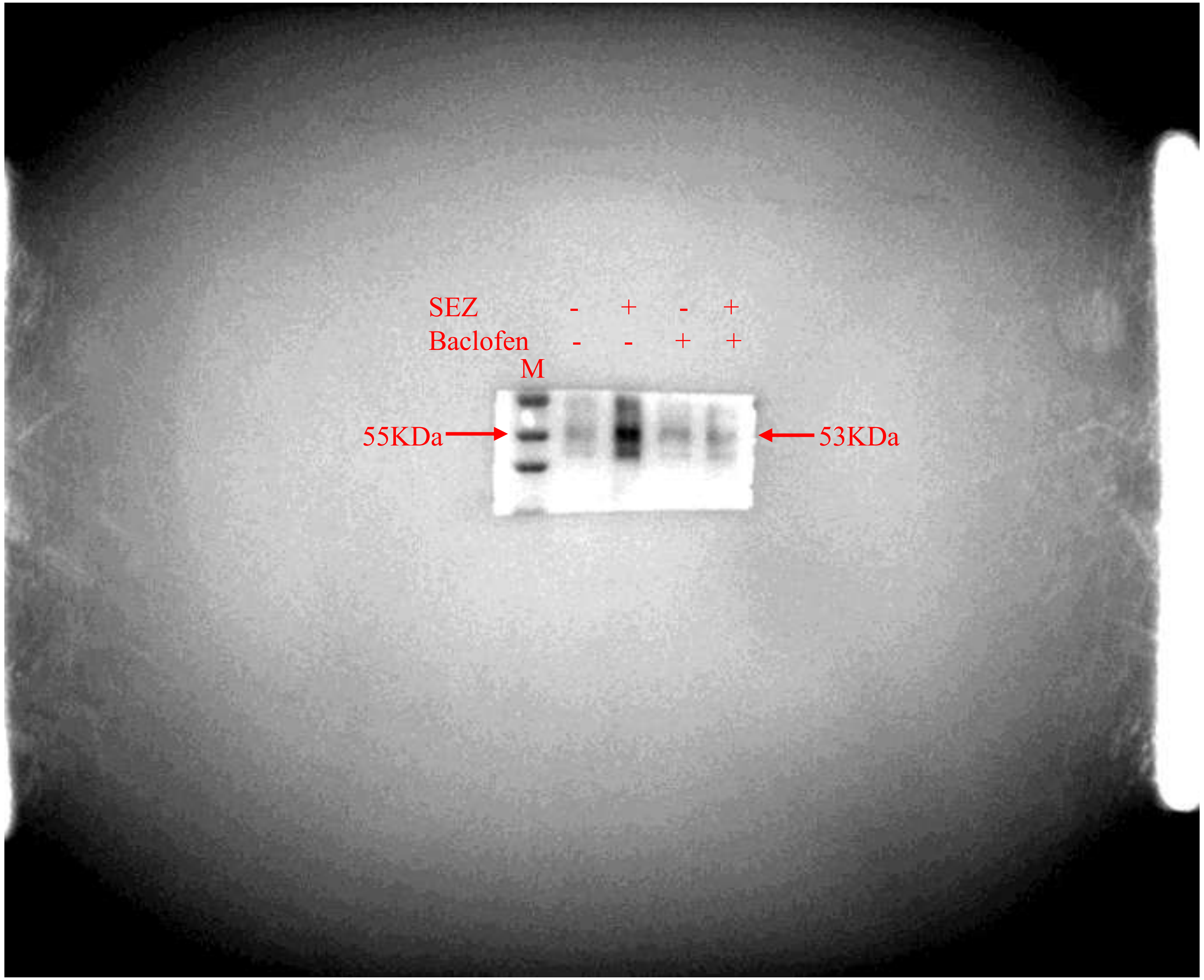

Figure 5 I β-actin (repeated 1)

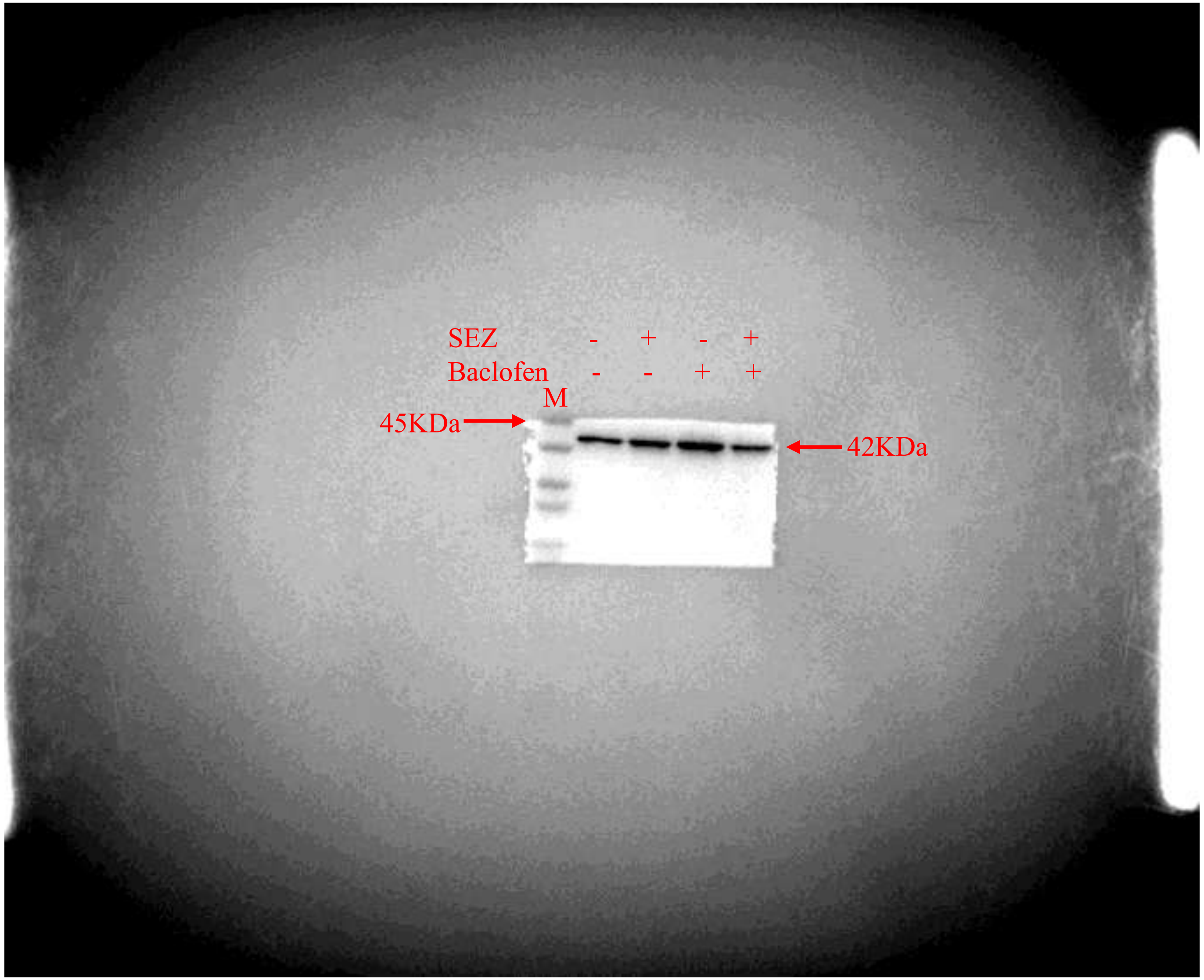

Figure 5 I β-actin (repeated 2)

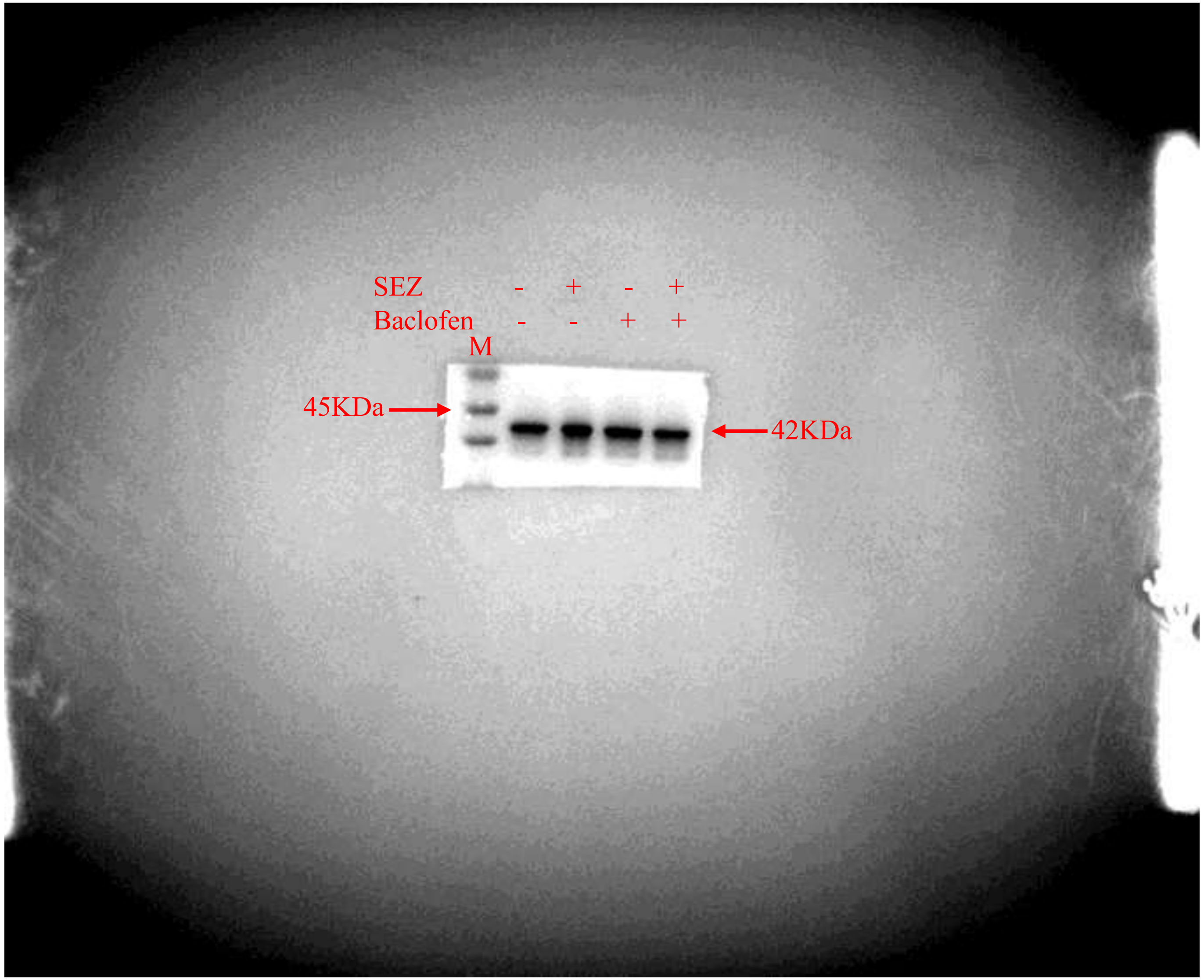

Figure 5 I β-actin (repeated 3)

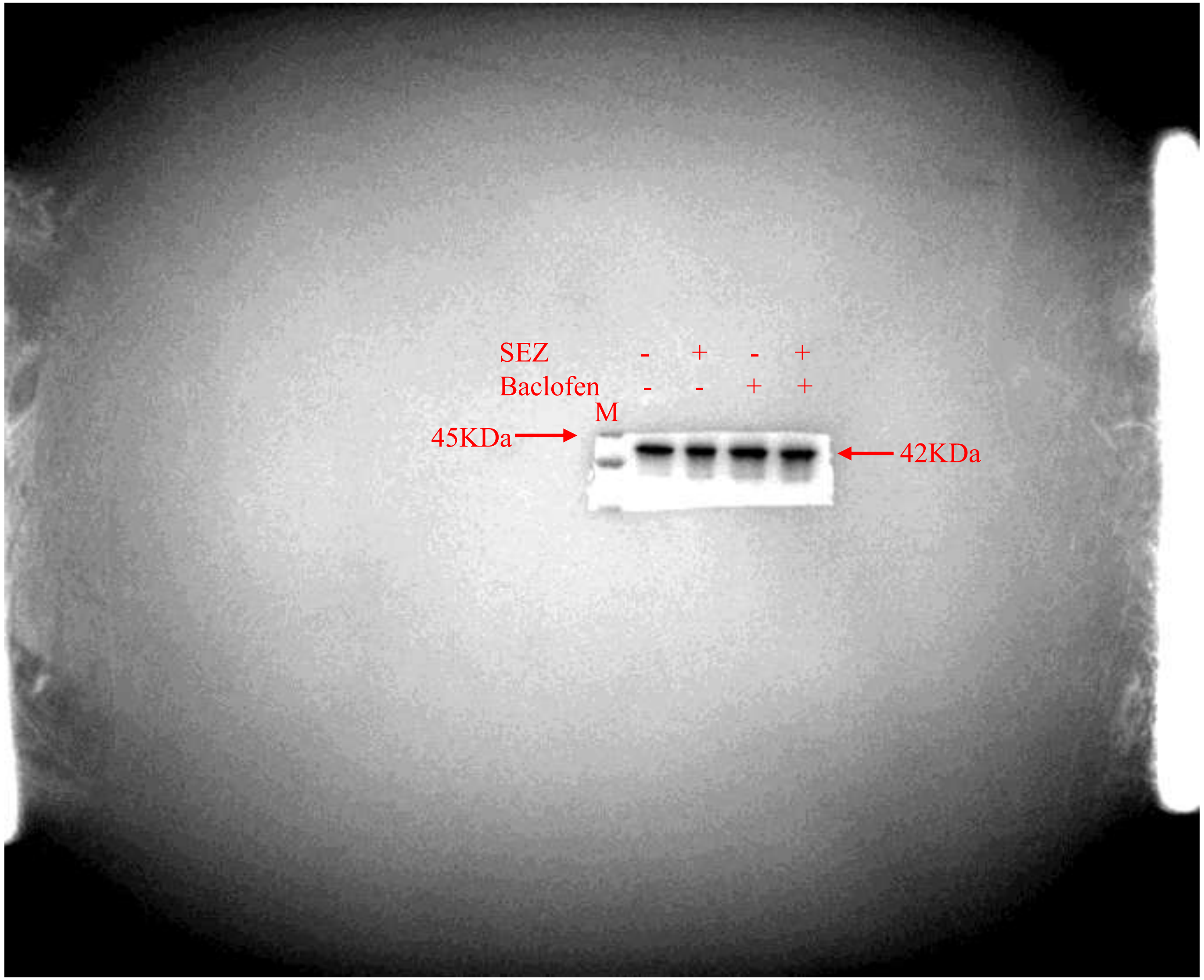

Figure 7 I IRAK-M (repeated 1)

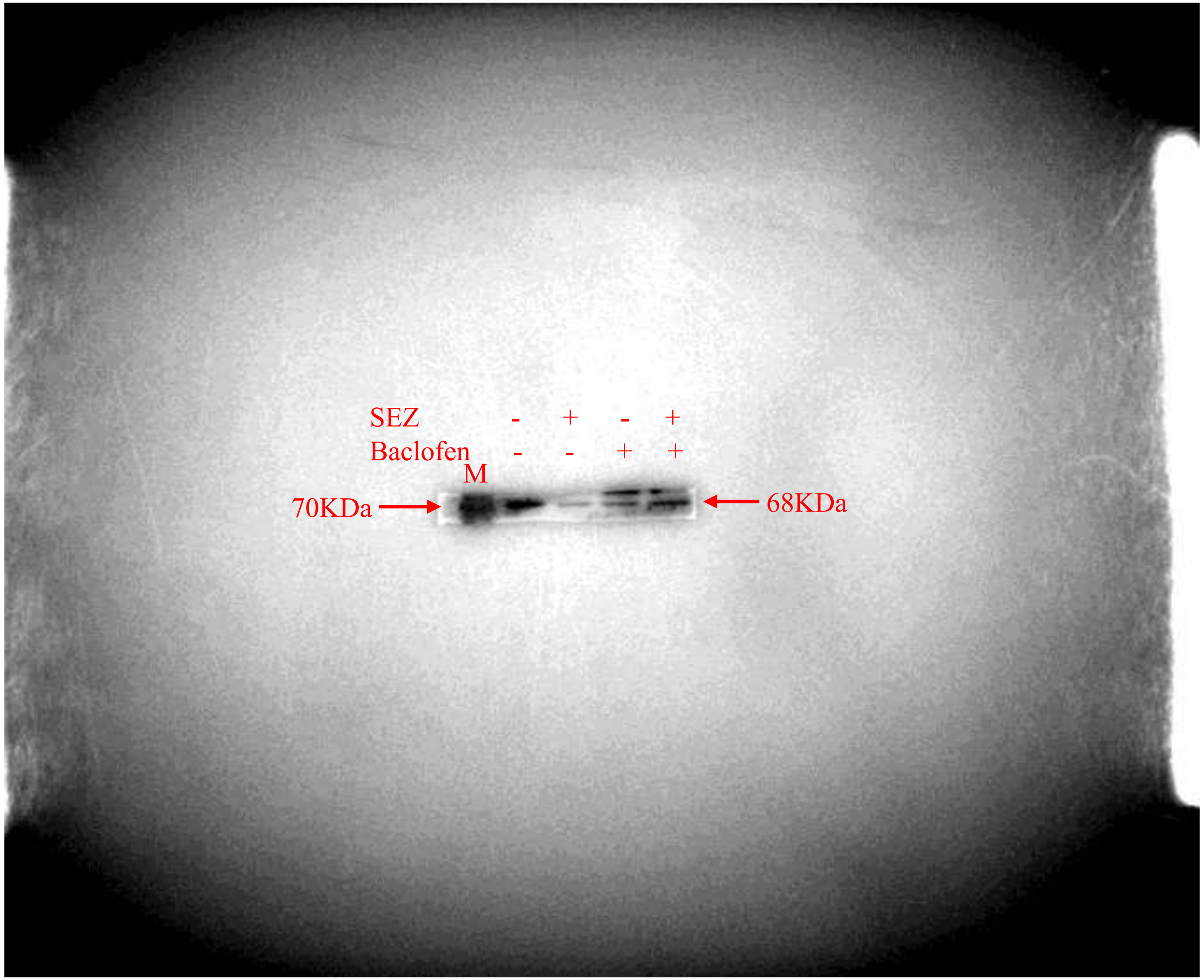

Figure 7 I IRAK-M (repeated 2)

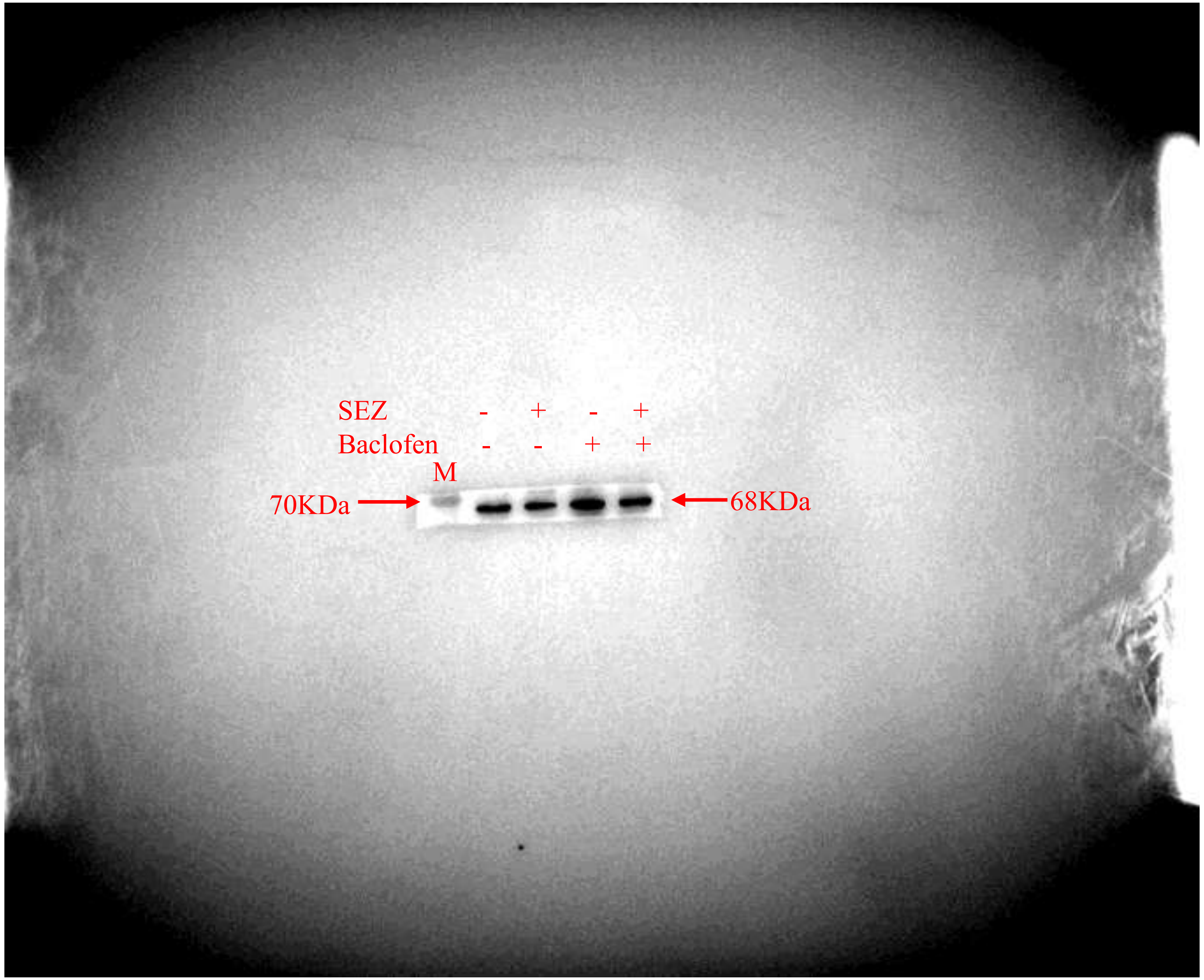

Figure 7 I IRAK-M (repeated 3)

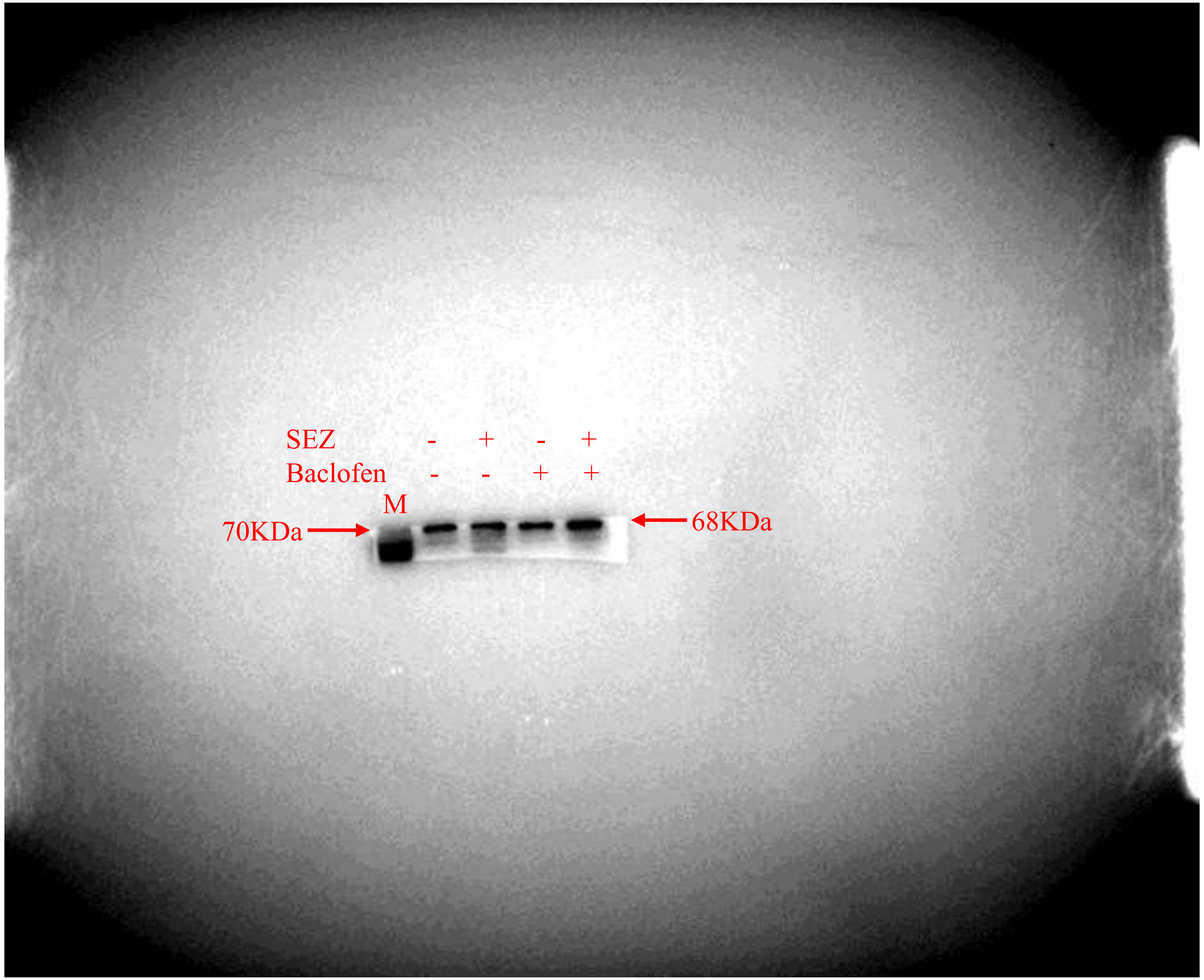

Figure 7 I NLRP3 (repeated 1)

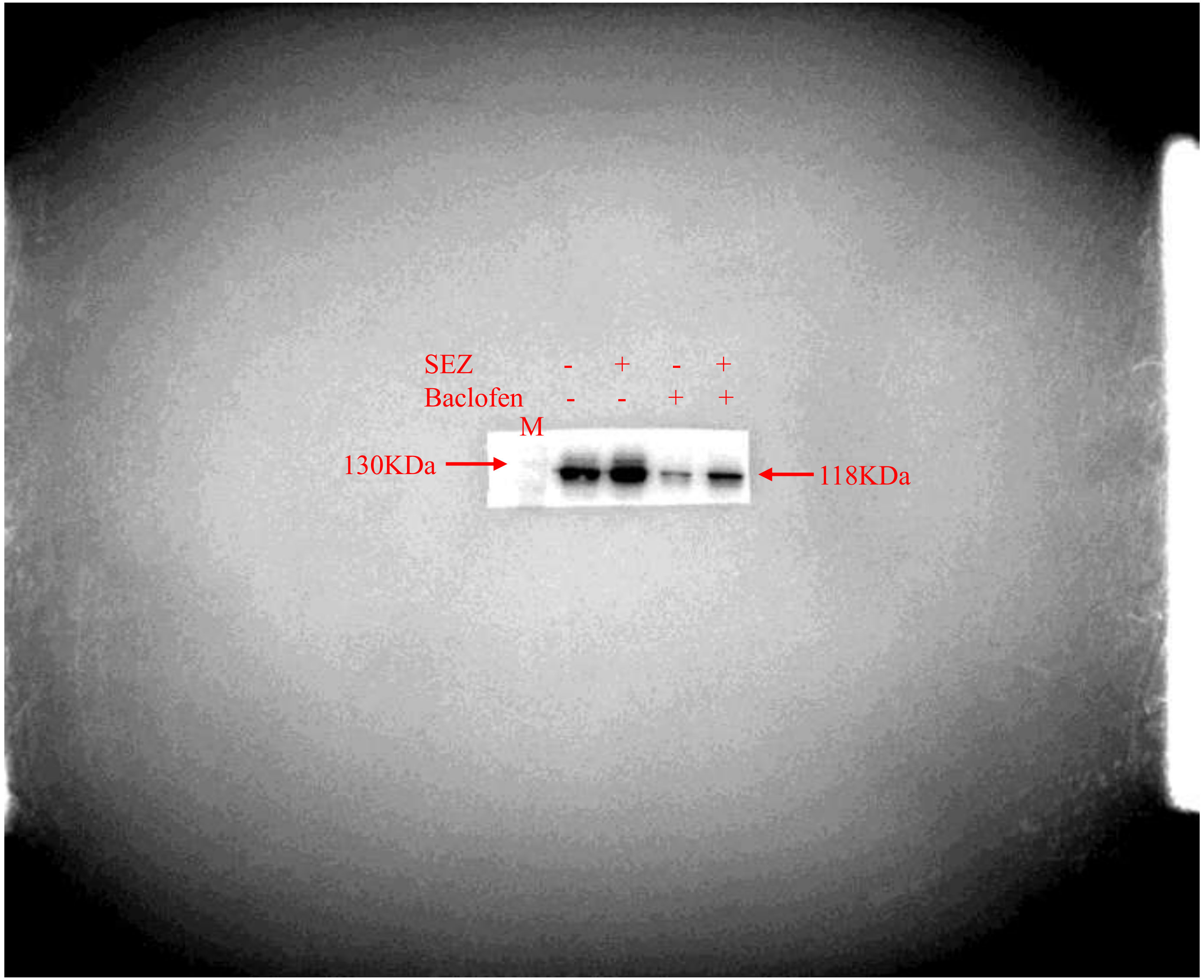

Figure 7 I NLRP3 (repeated 2)

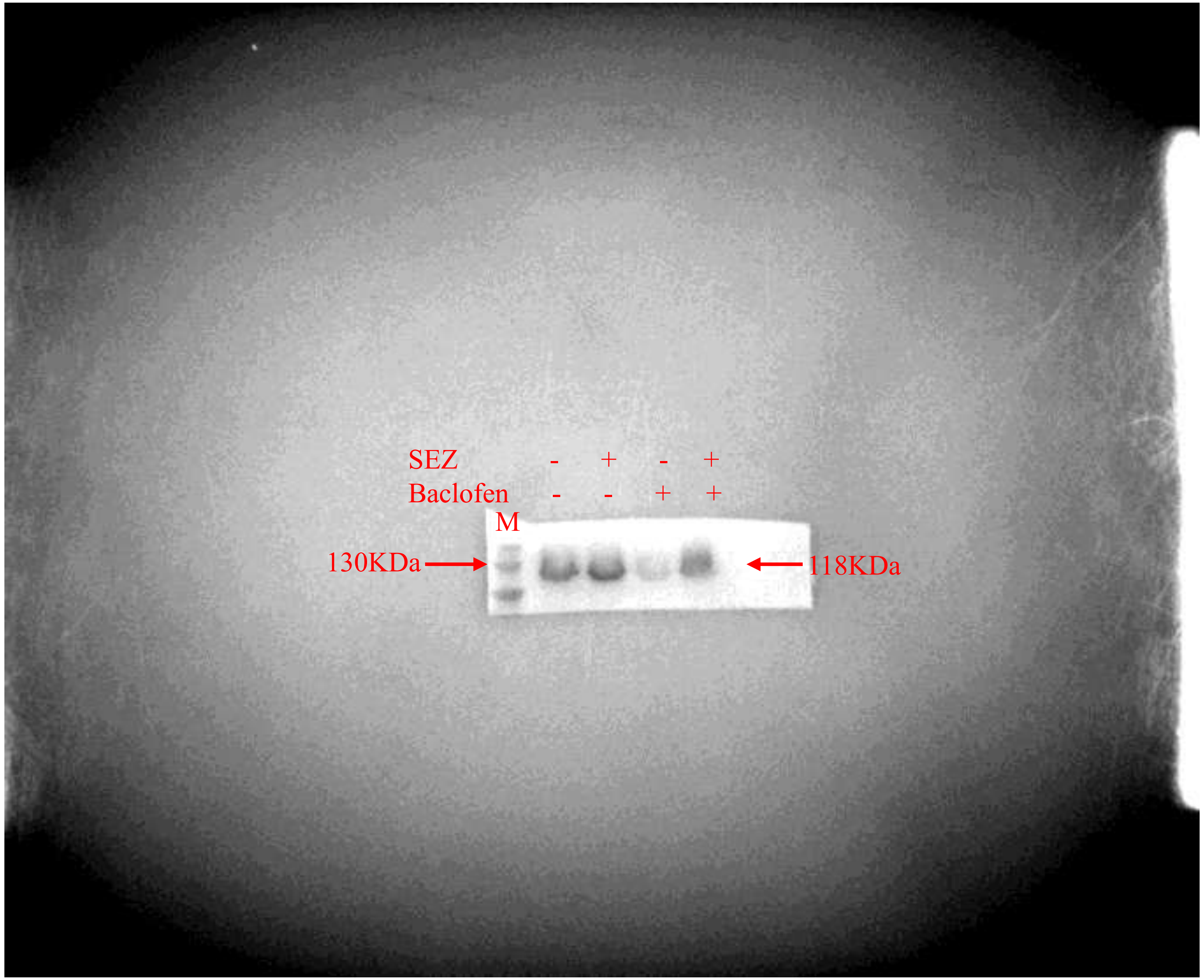

Figure 7 I NLRP3 (repeated 3)

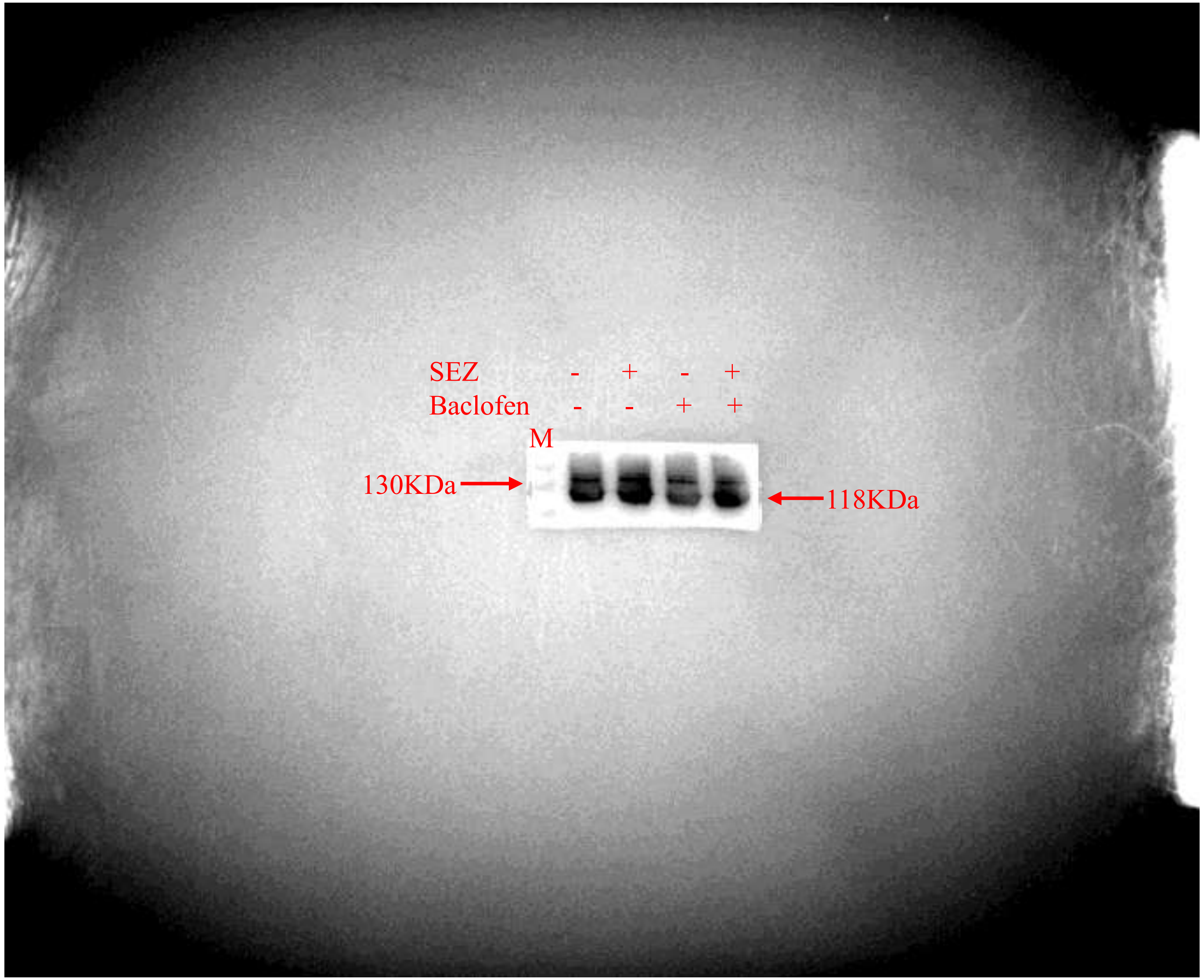

Figure 7 I caspase-1 (repeated 1)

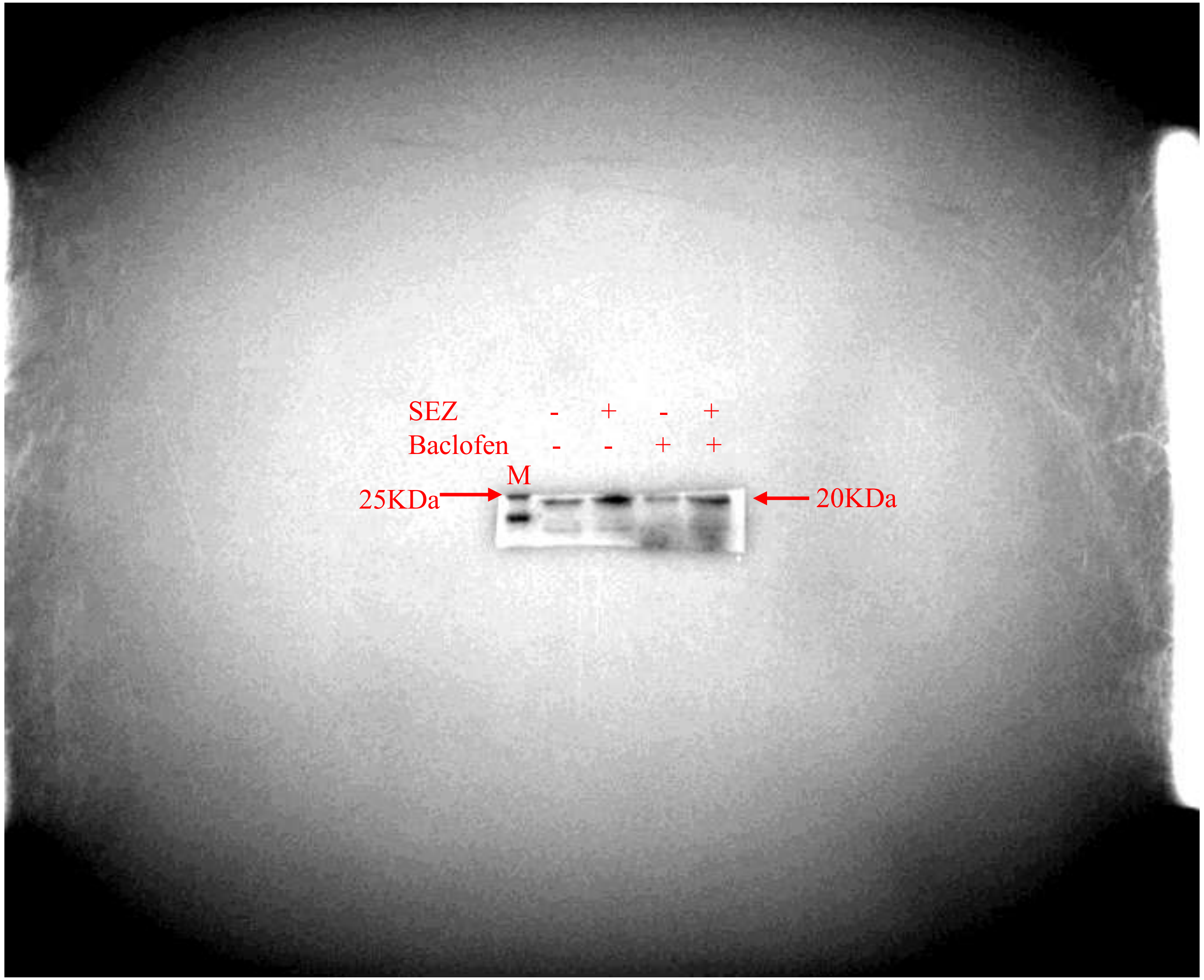

Figure 7 I caspase-1 (repeated 2)

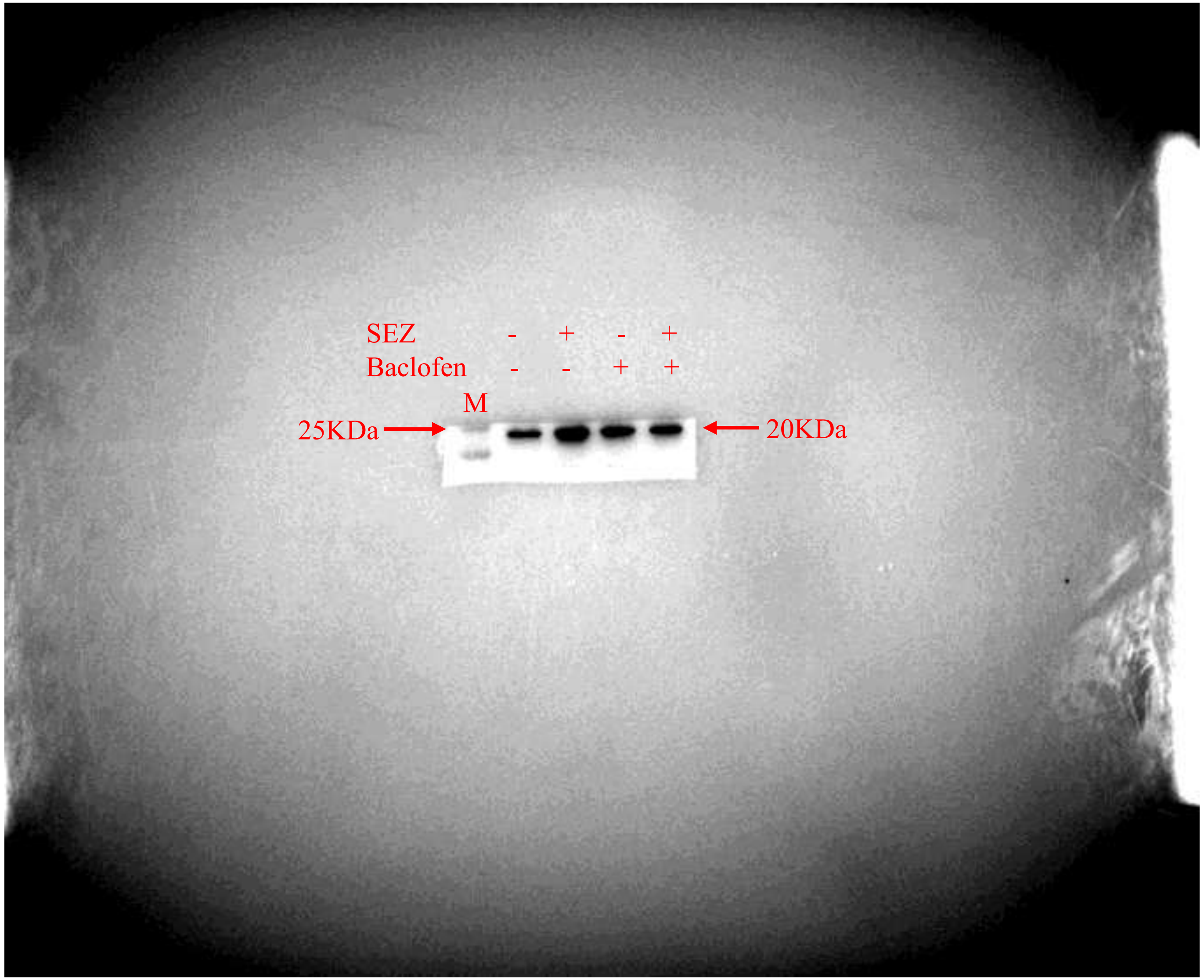

Figure 7 I caspase-1 (repeated 3)

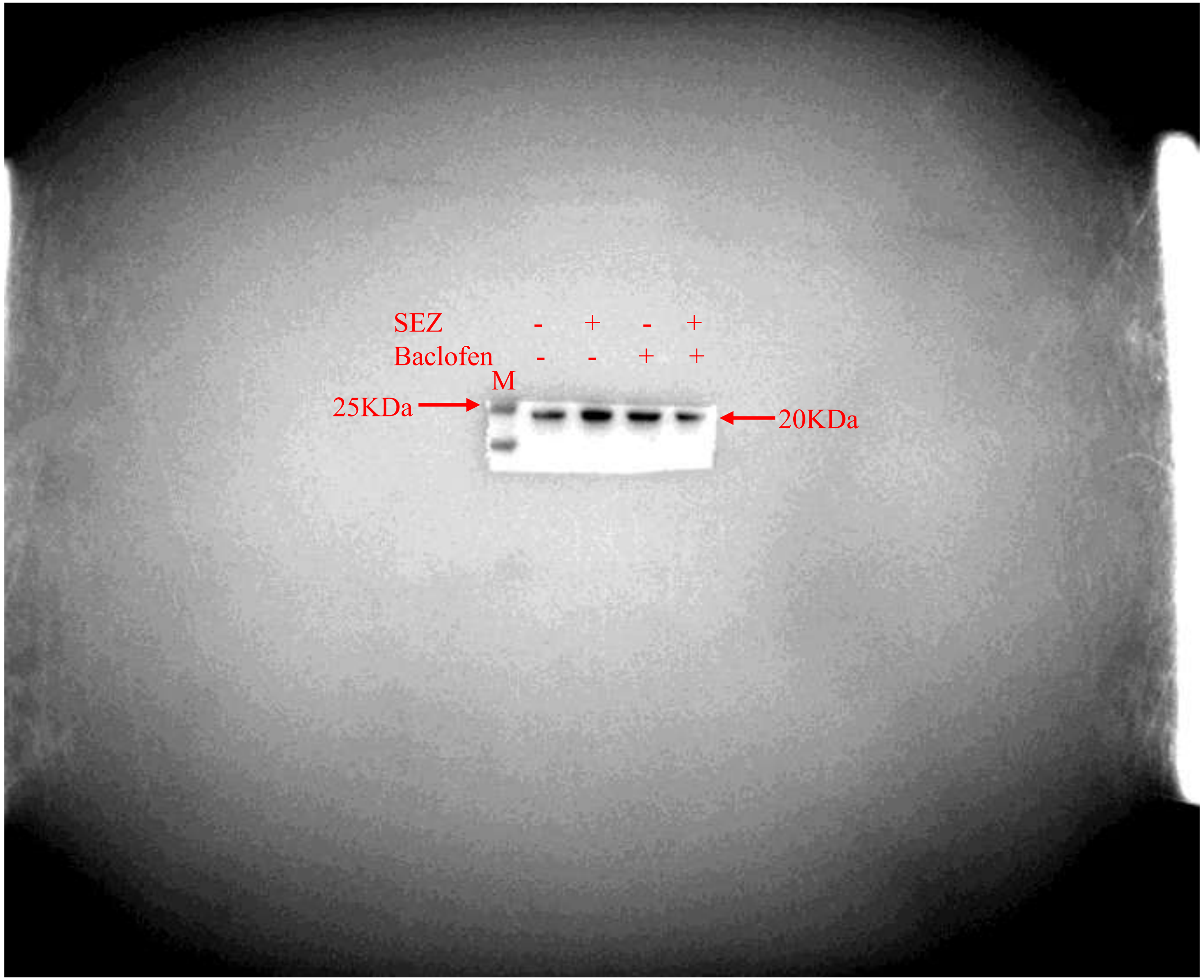

Figure 7 I GSDMD (repeated 1)

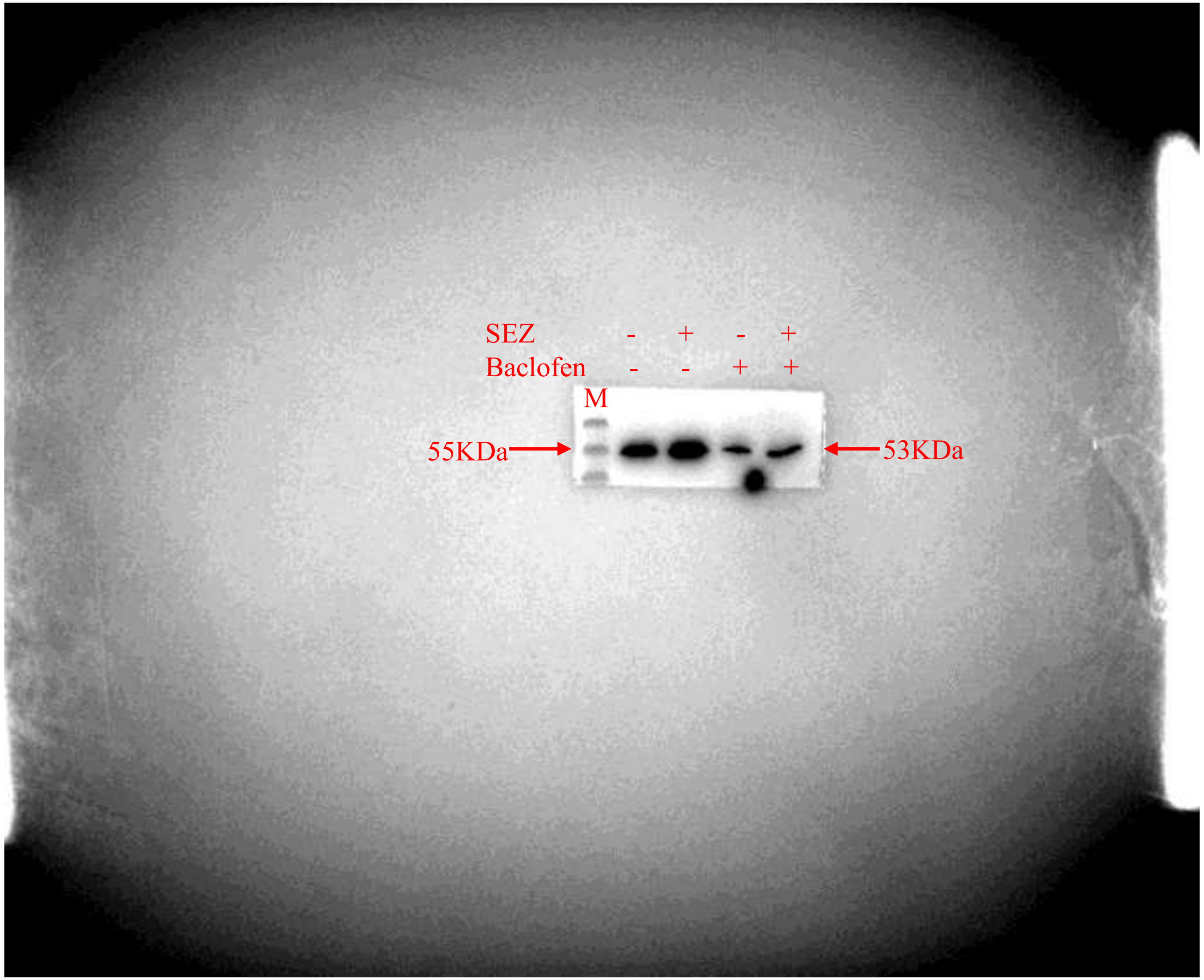

Figure 7 I GSDMD (repeated 2)

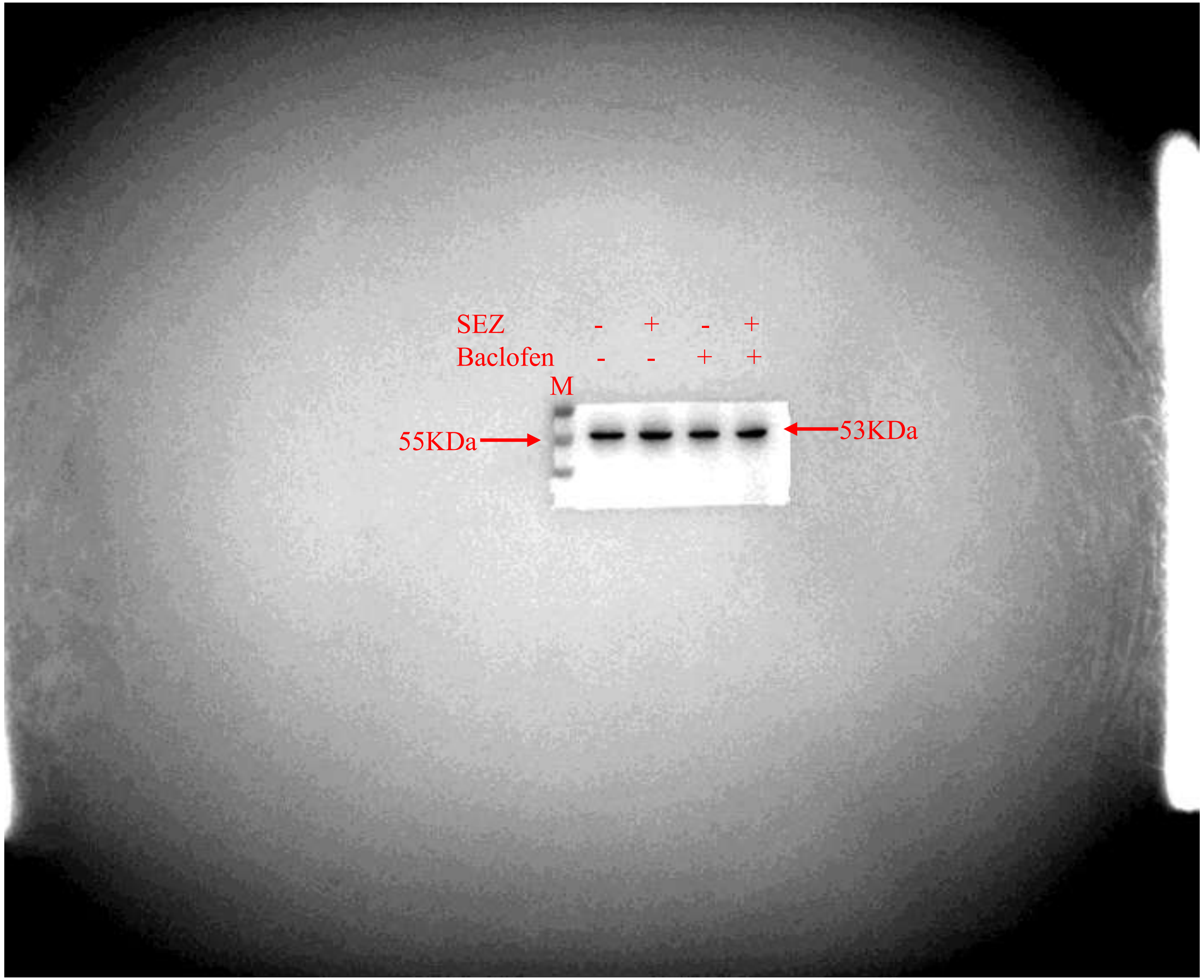

Figure 7 I GSDMD (repeated 3)

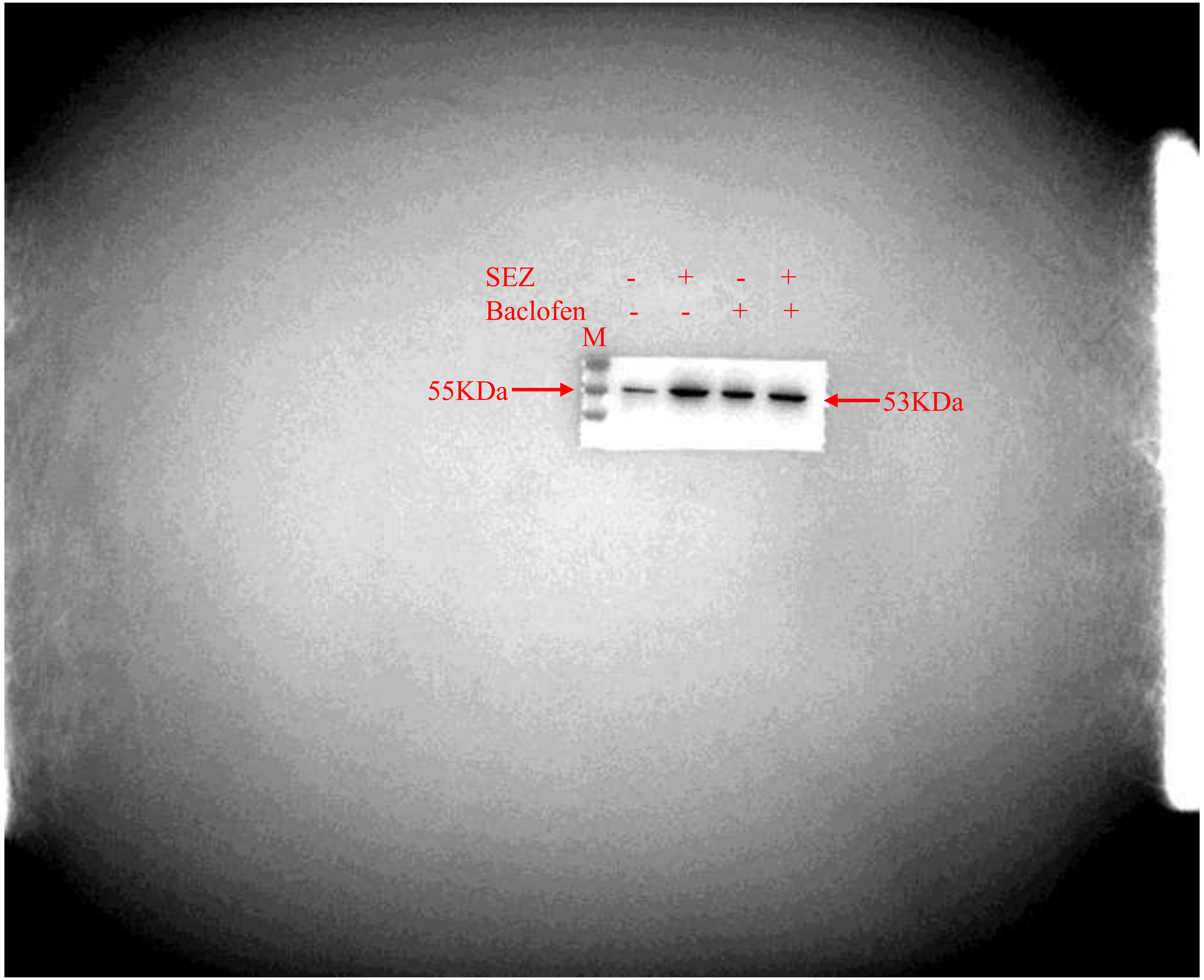

Figure 7 I β-actin (repeated 1)

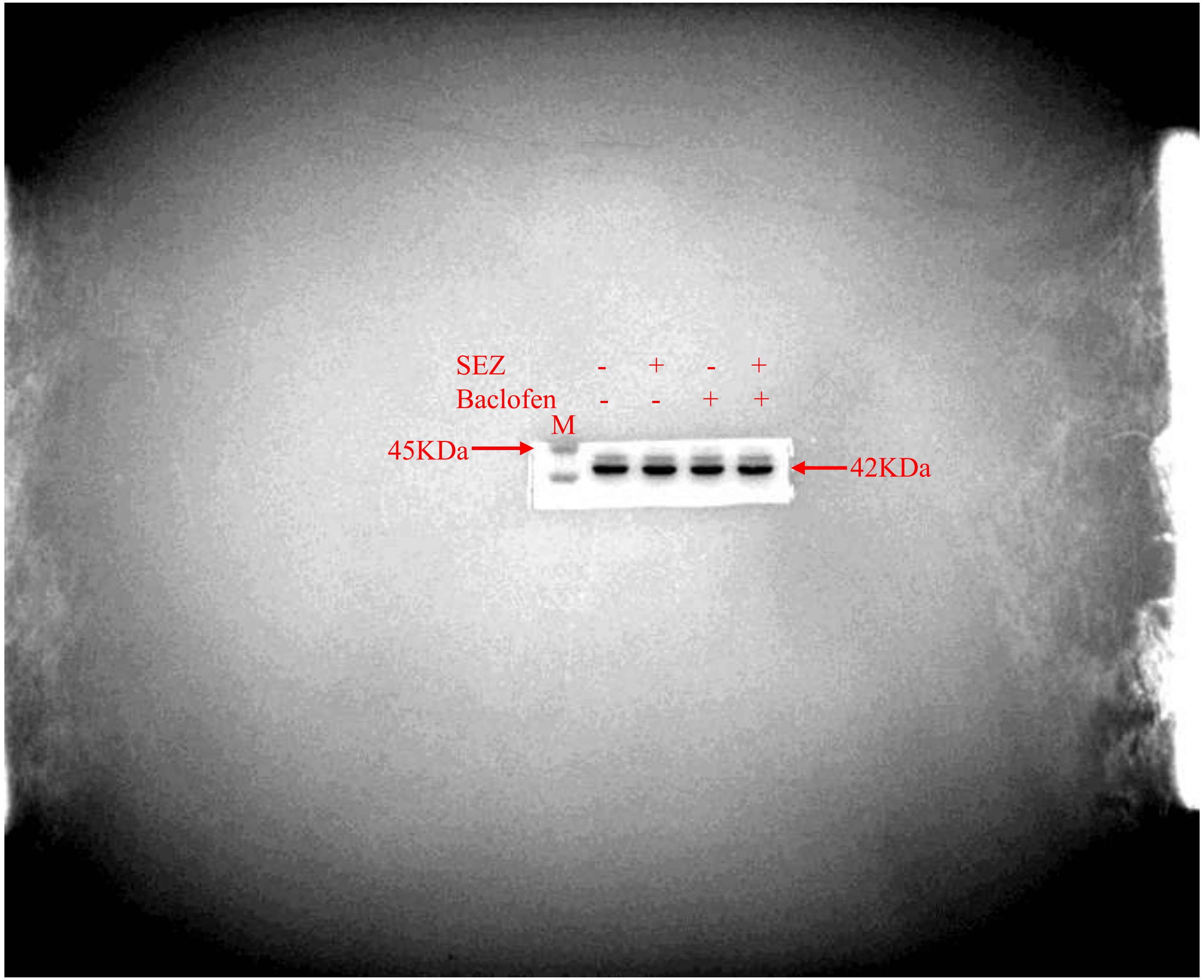

Figure 7 I β-actin (repeated 2)

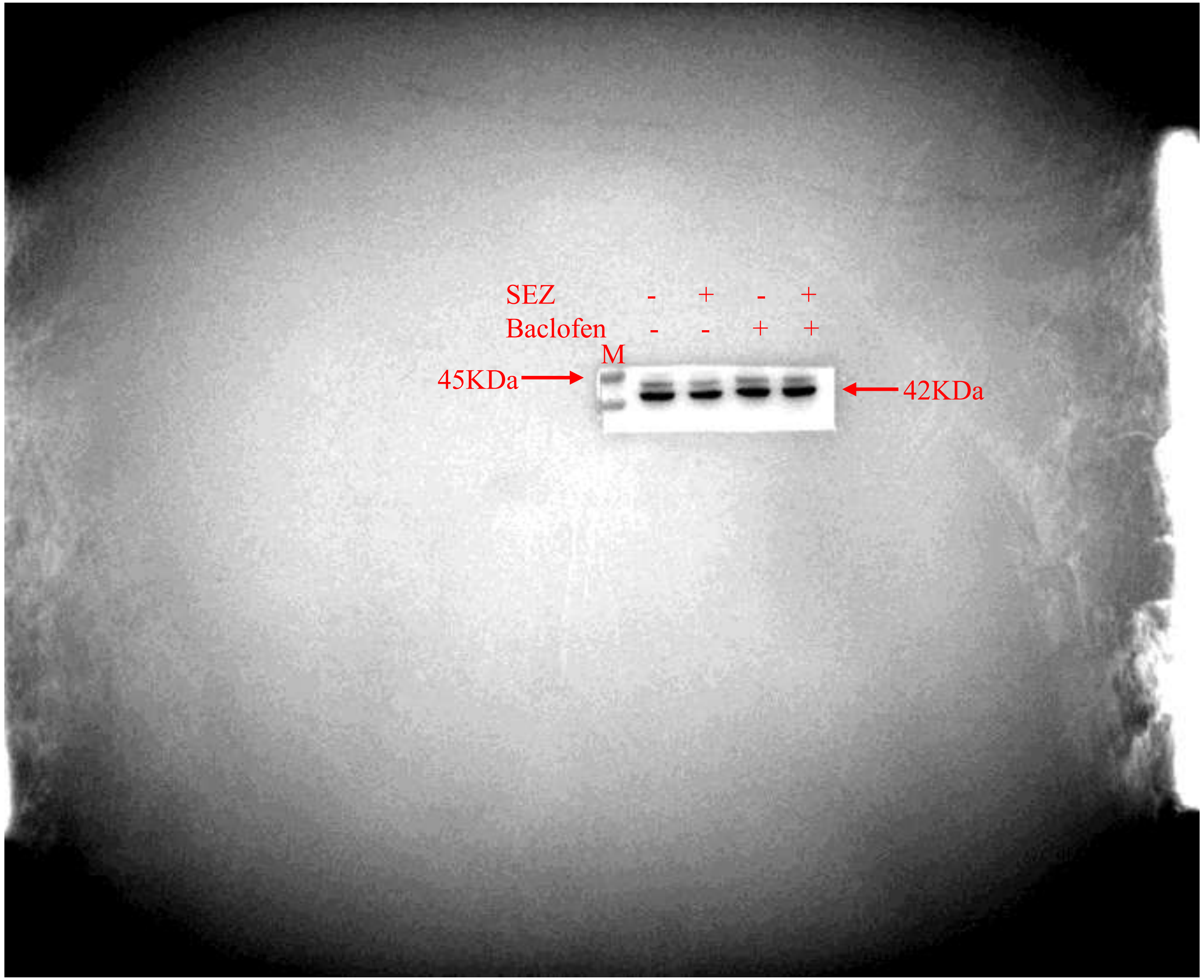

Figure 7 I β-actin (repeated 3)

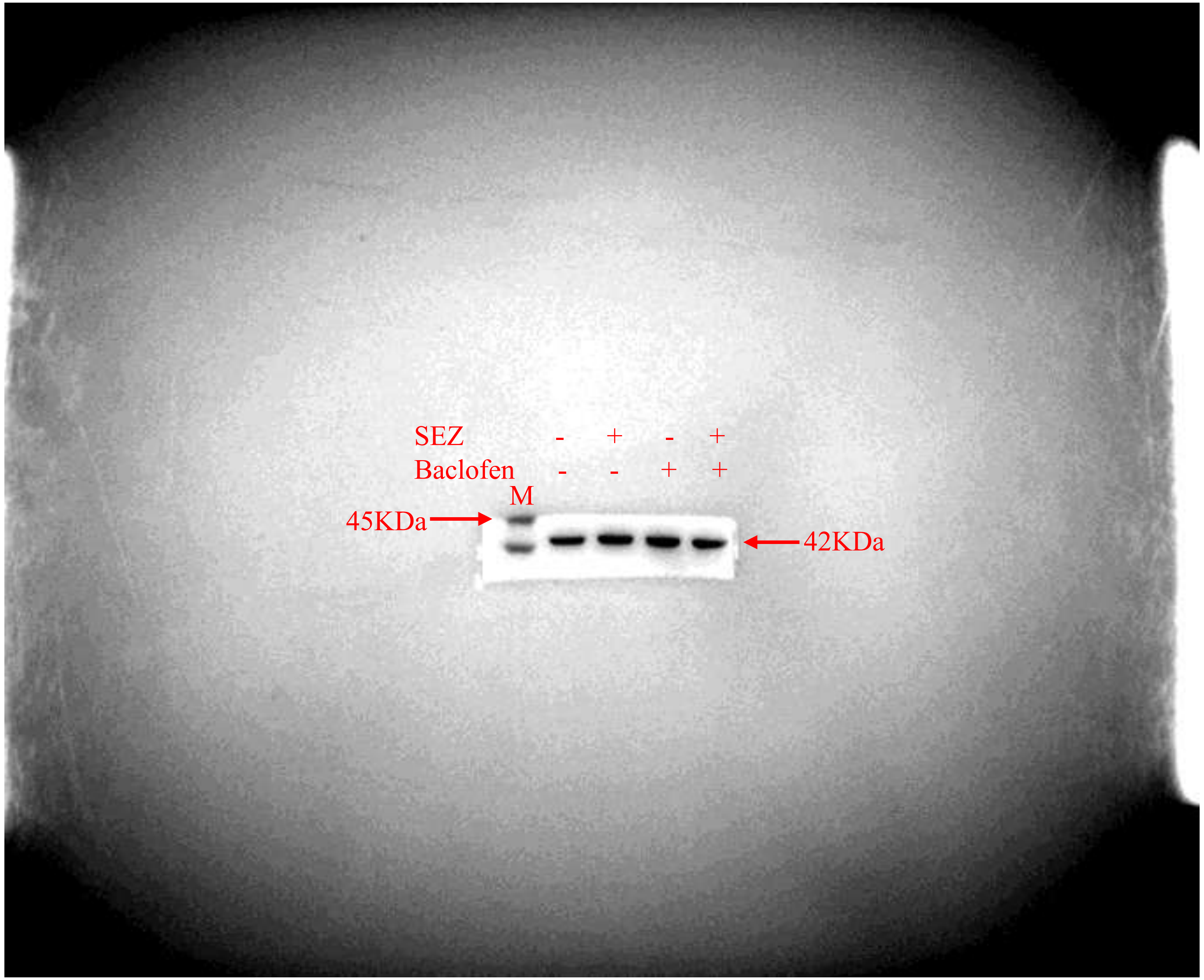

Figure 9 M Claudin-1(repeated 1)

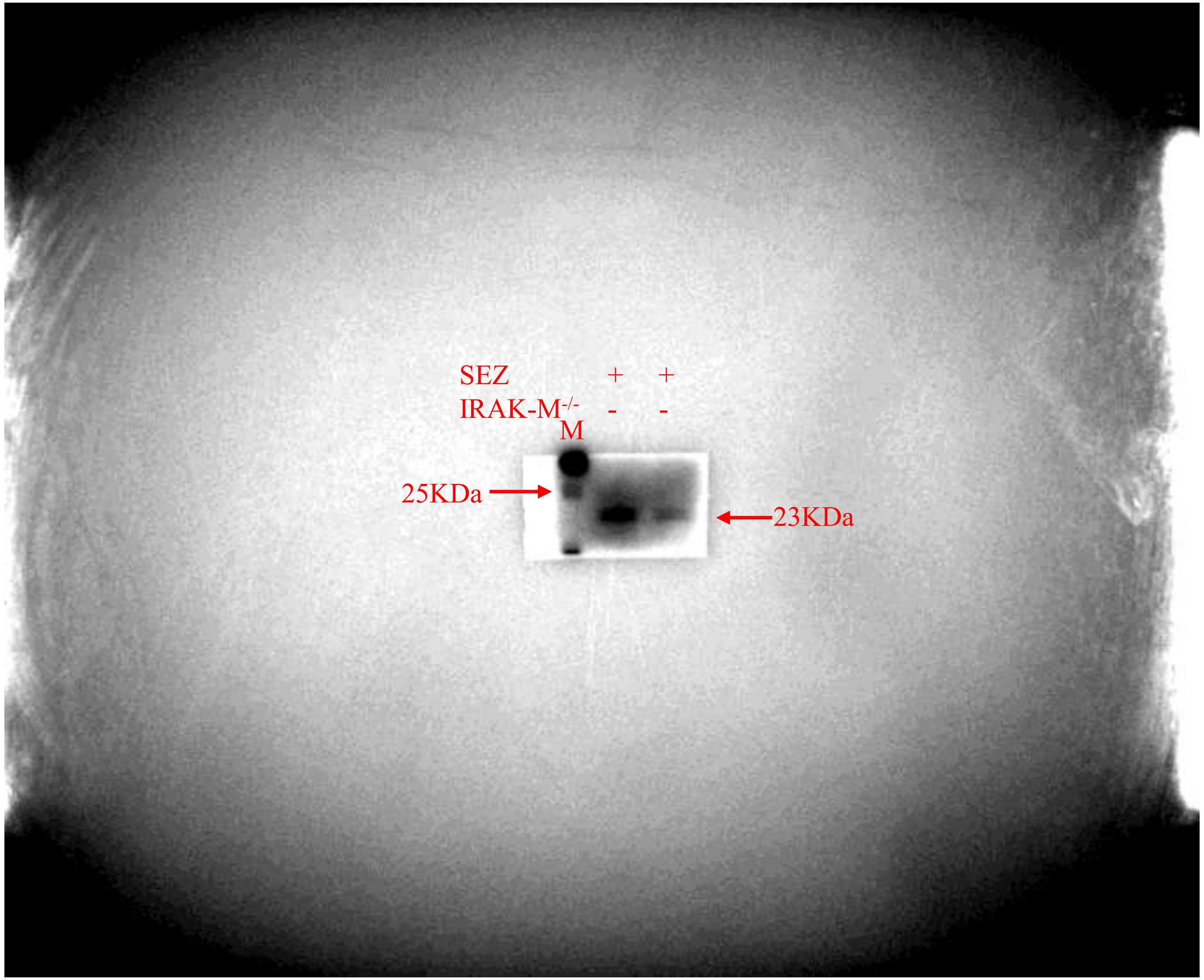

Figure 9 M Claudin-1(repeated 2)

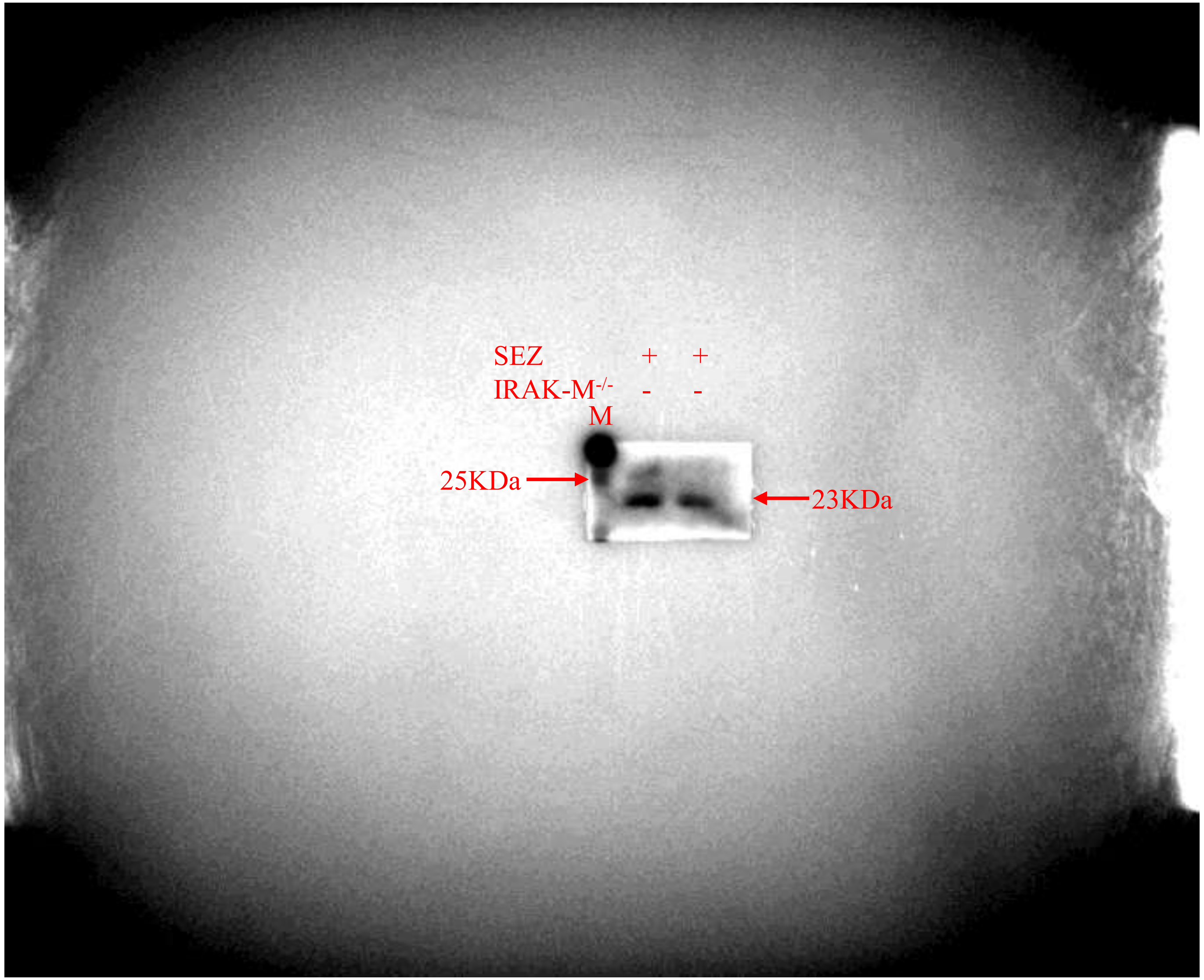

Figure 9 M Claudin-1(repeated 3)

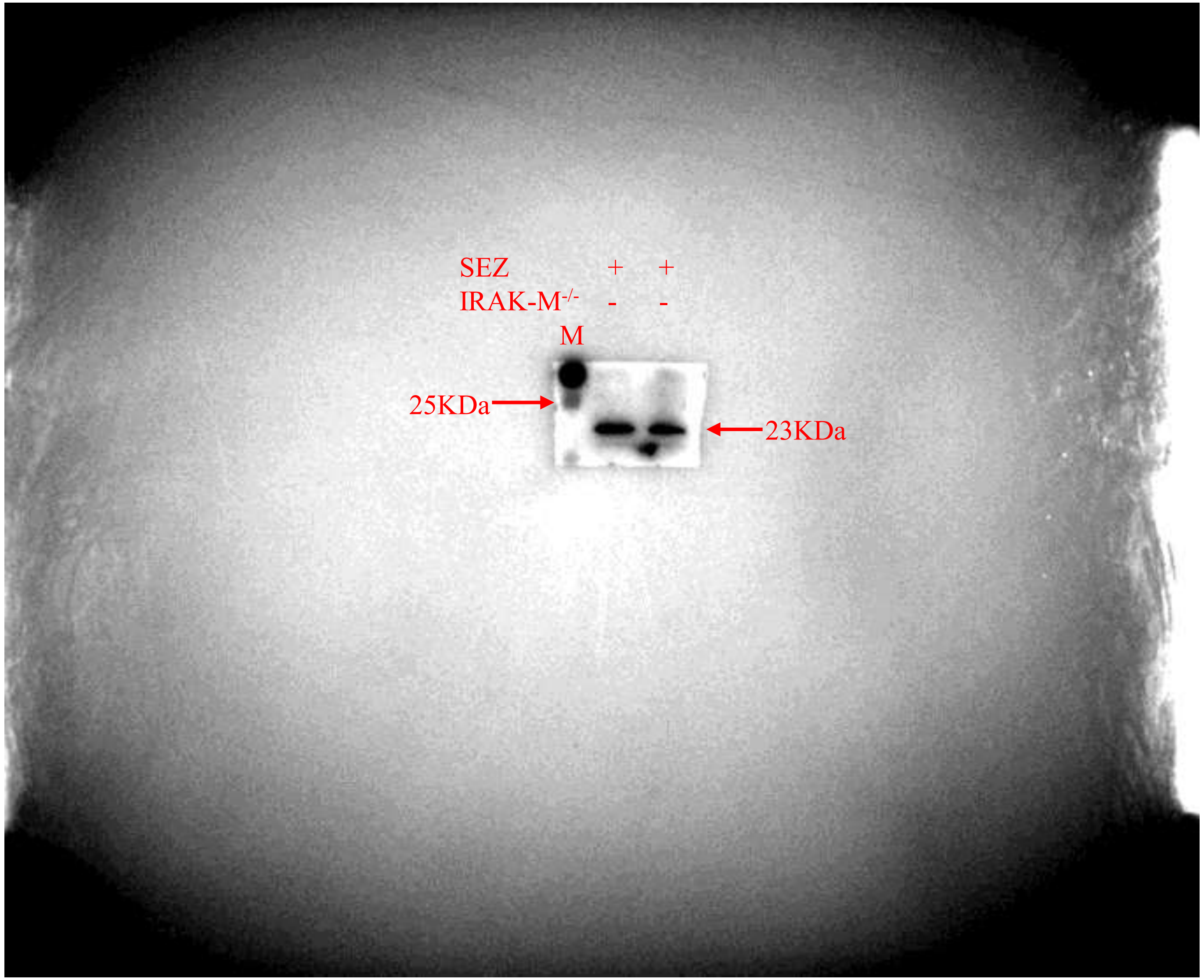

Figure 9 M Occludin (repeated 1)

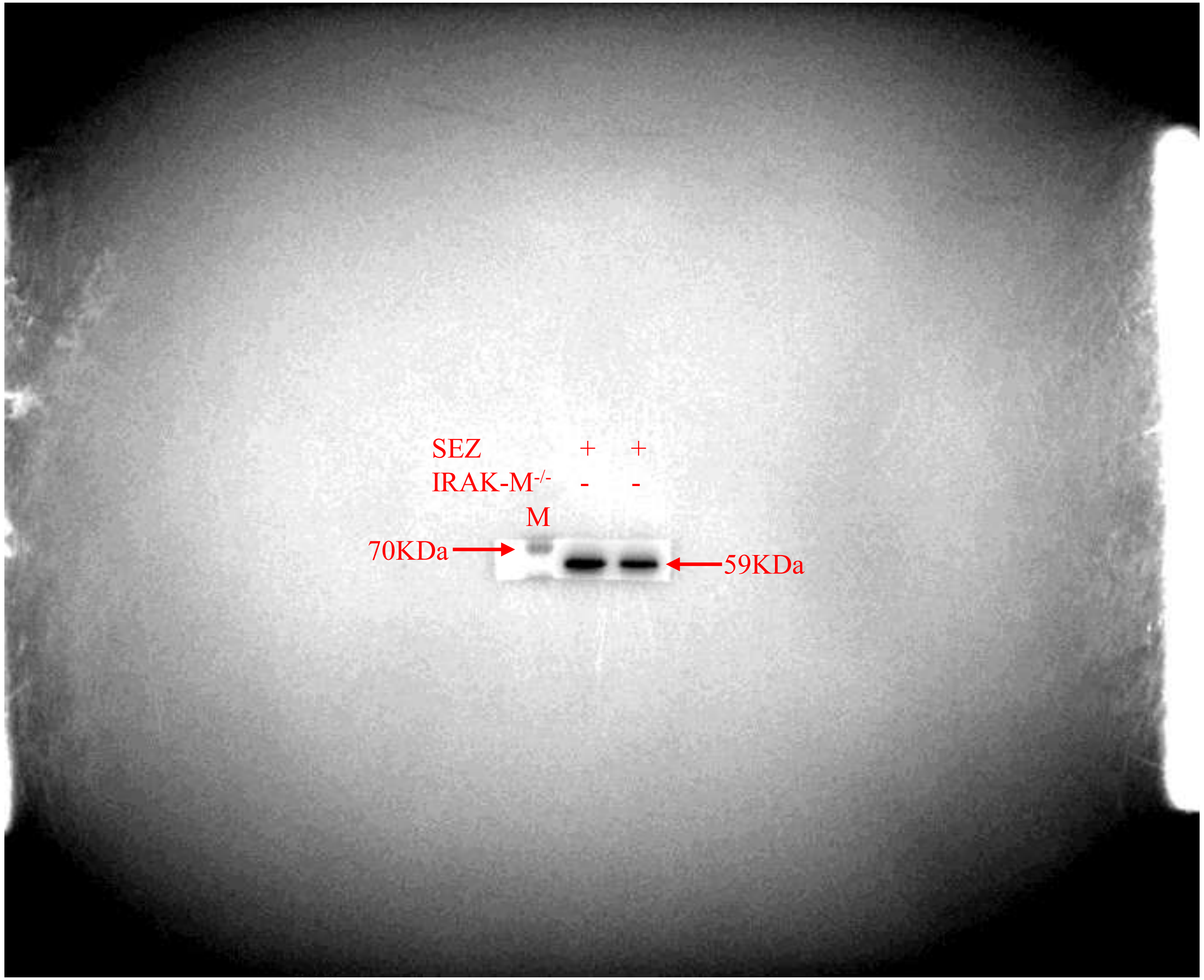

Figure 9 M Occludin (repeated 2)

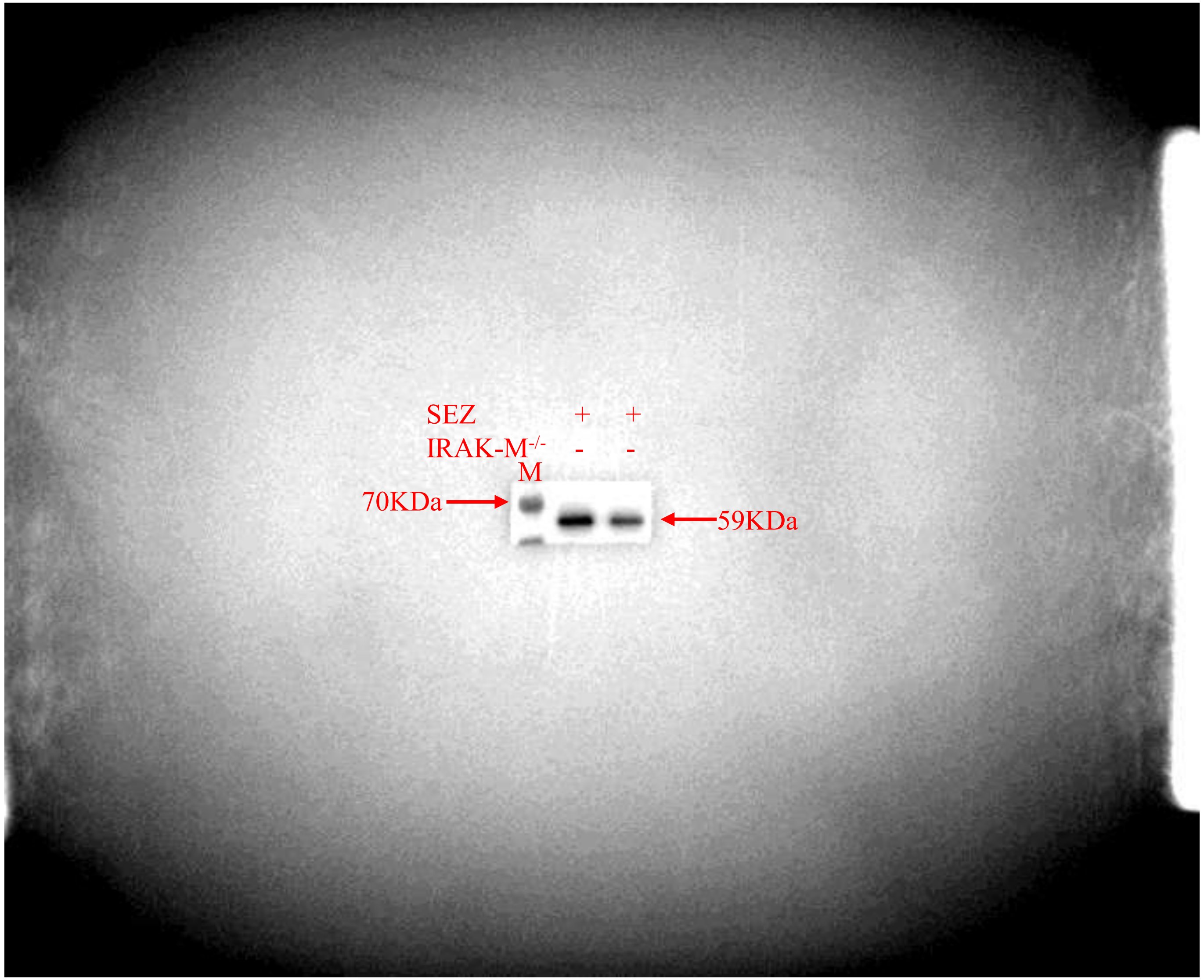

Figure 9 M Occludin (repeated 3)

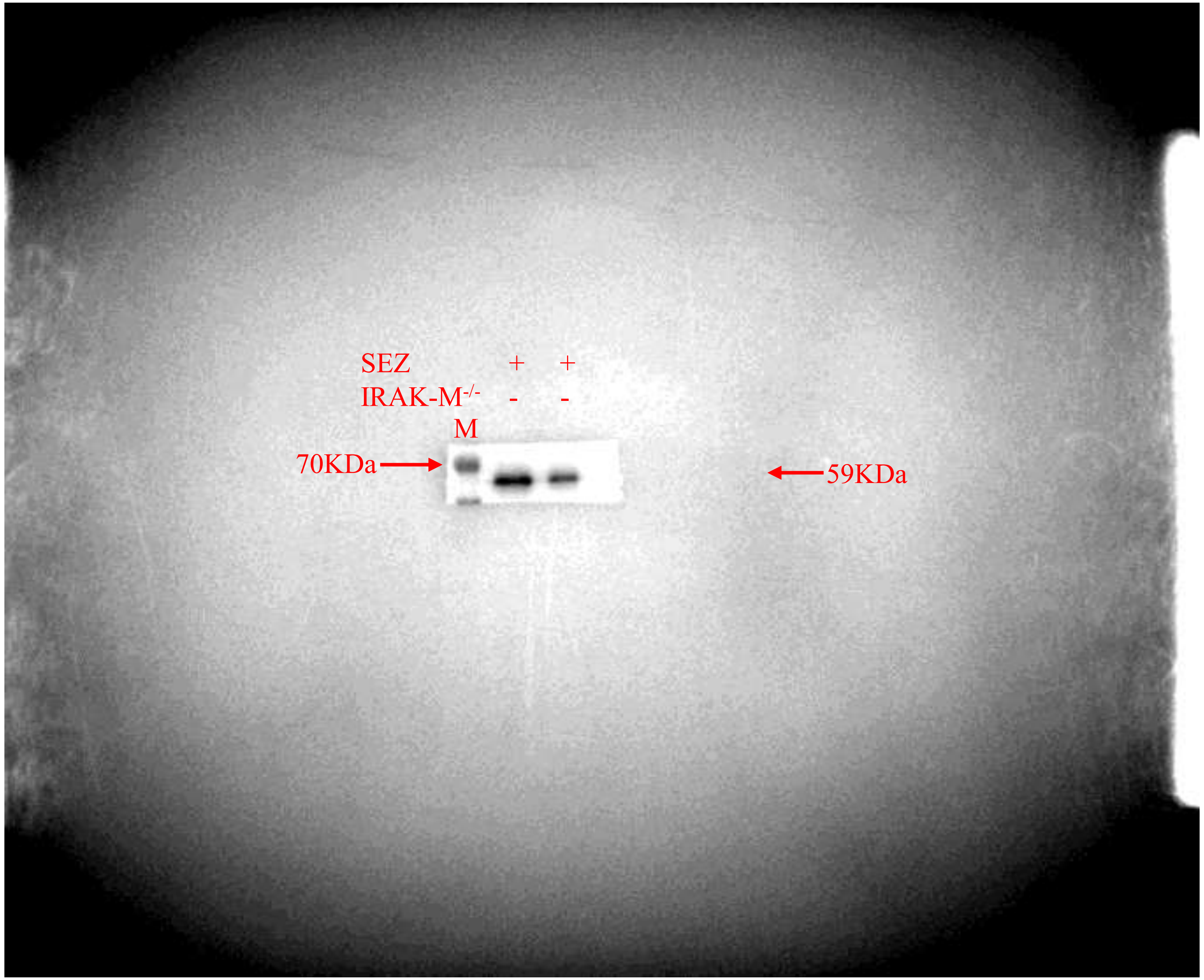

Figure 9 M ZO-1 (repeated 1)

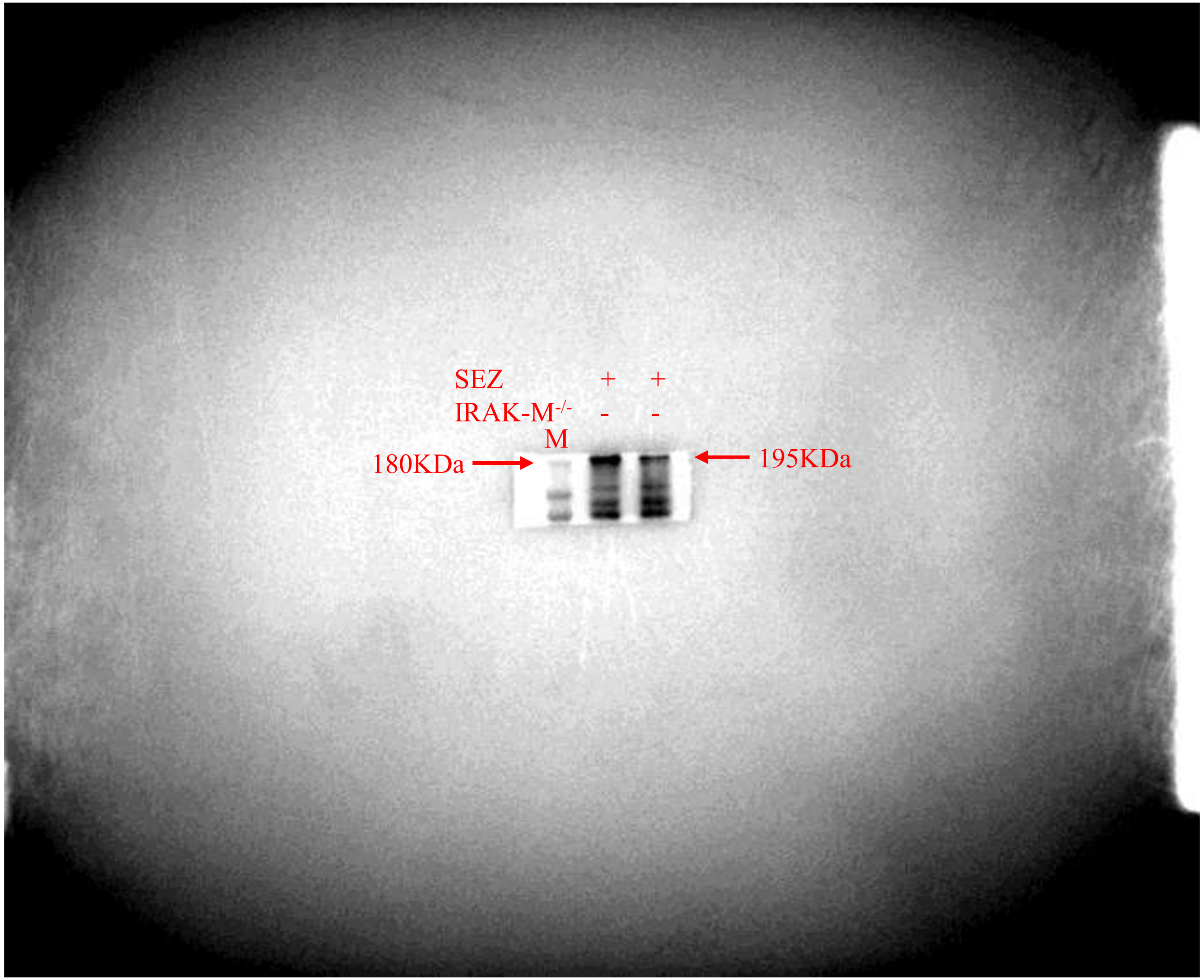

Figure 9 M ZO-1 (repeated 2)

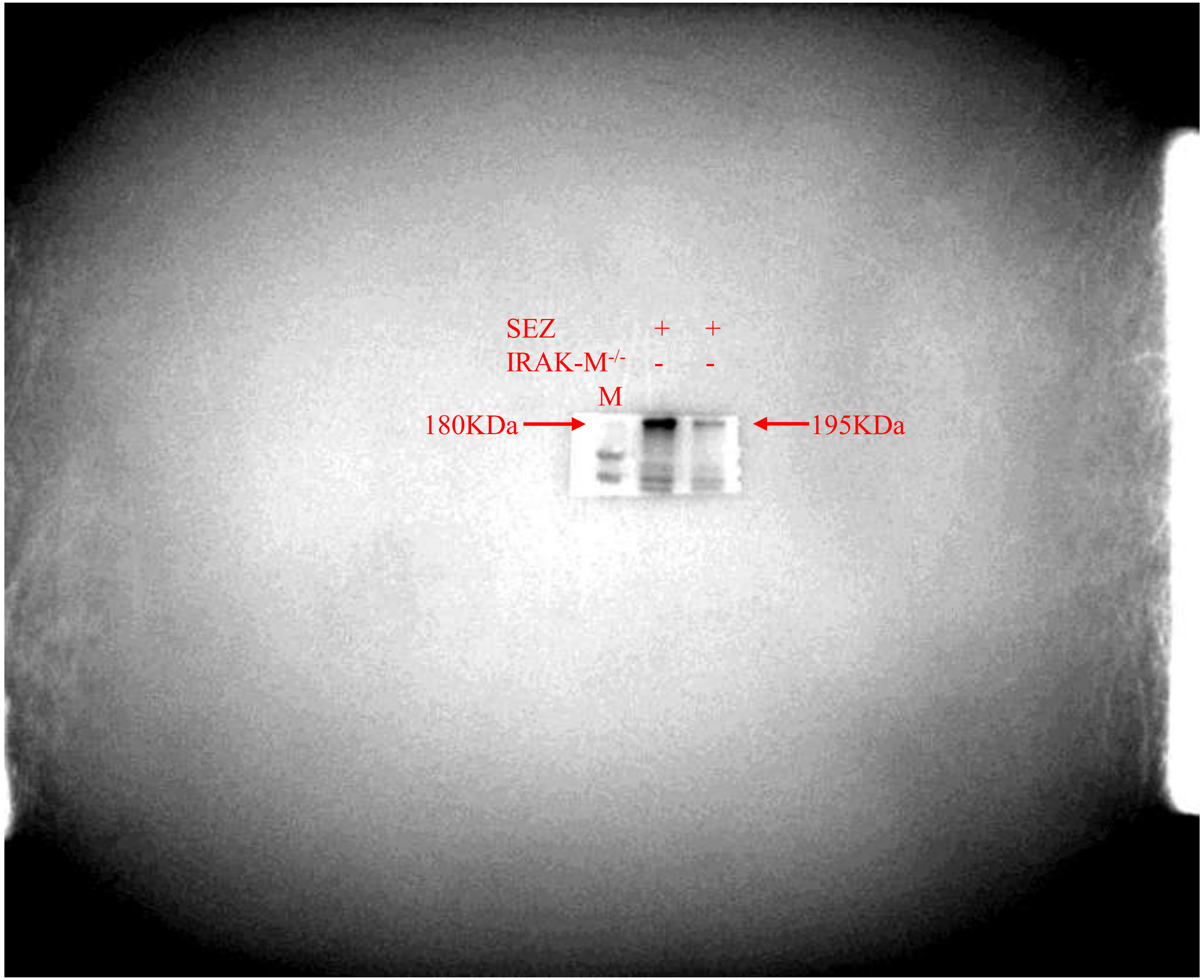

Figure 9 M ZO-1 (repeated 3)

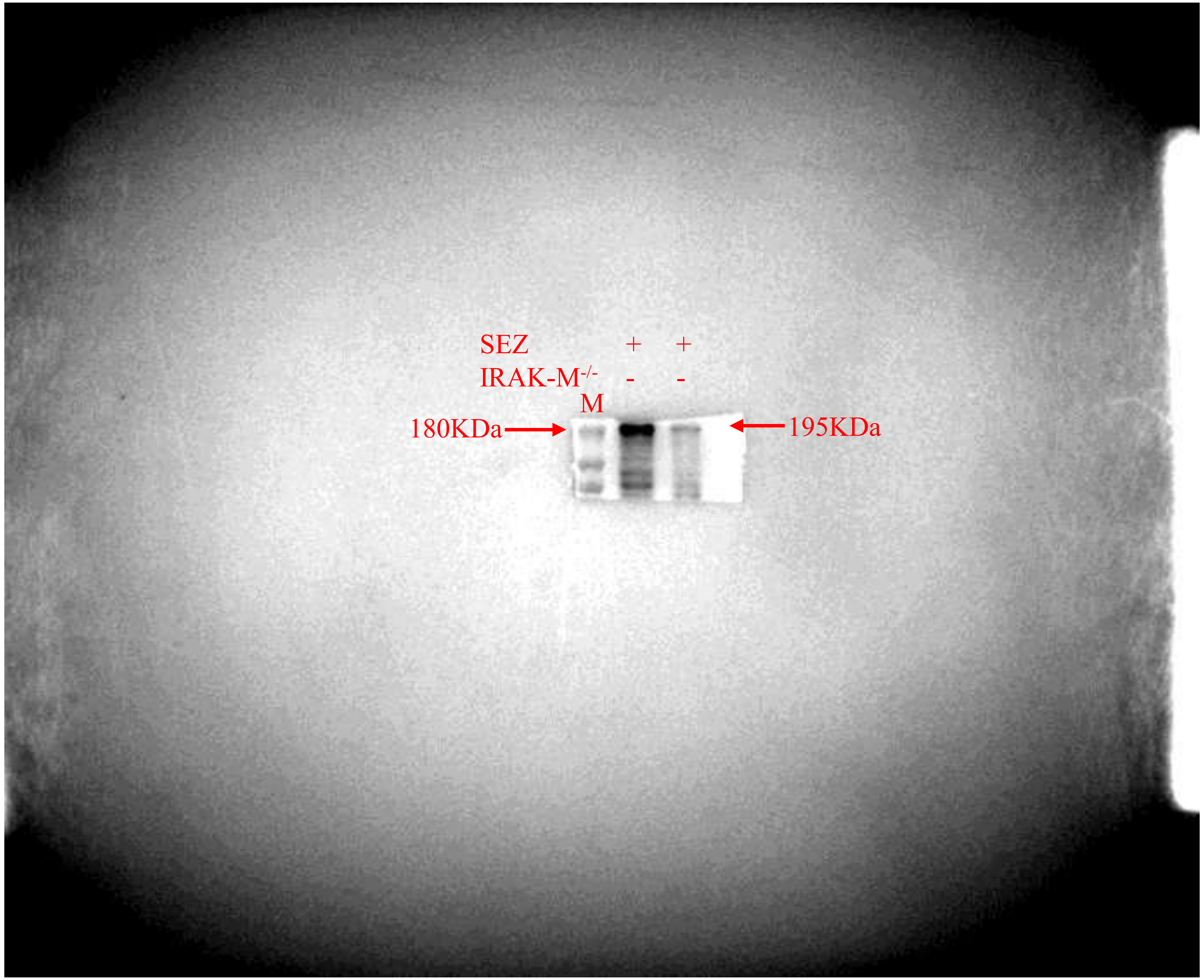

Figure 9 M β-actin (repeated 1)

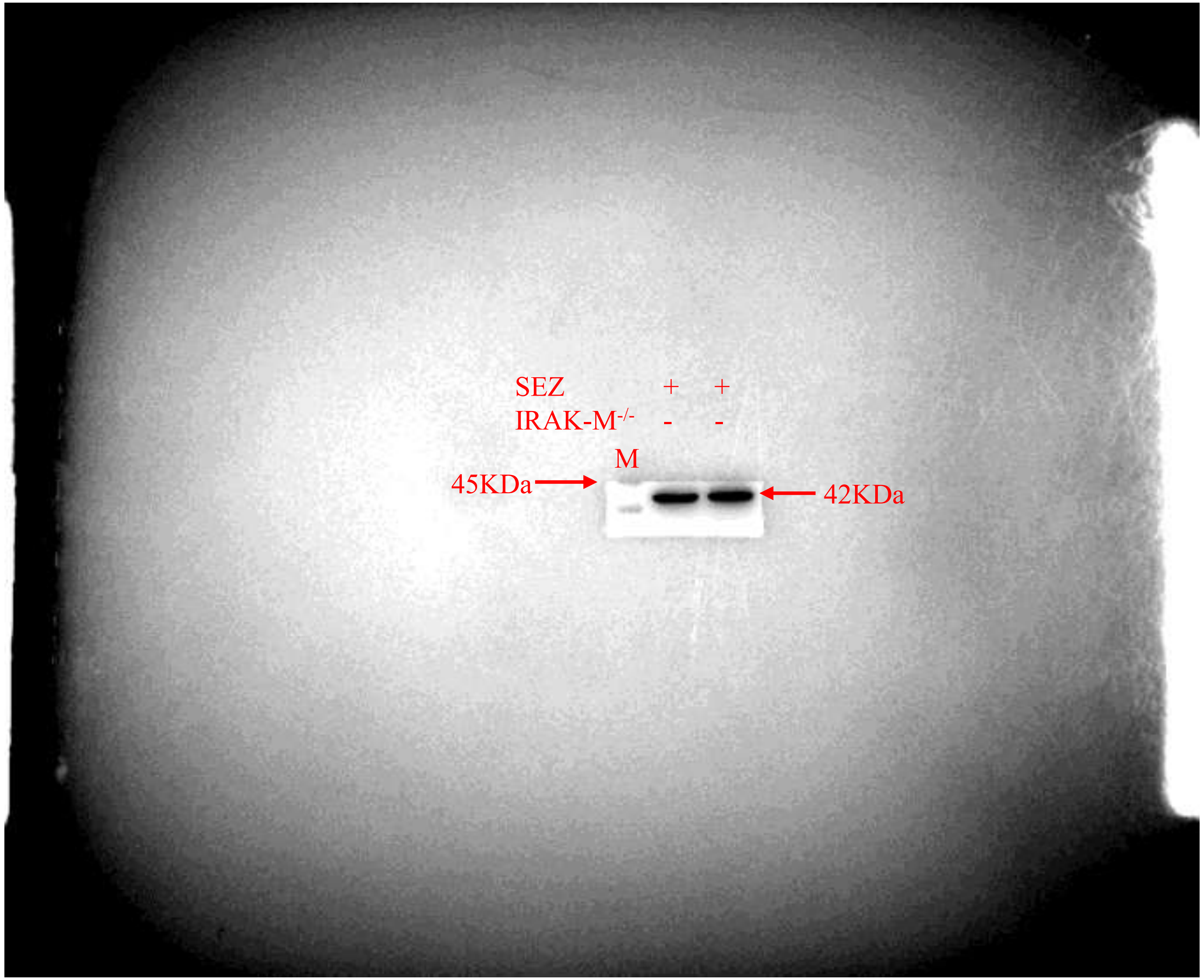

Figure 9 M β-actin (repeated 2)

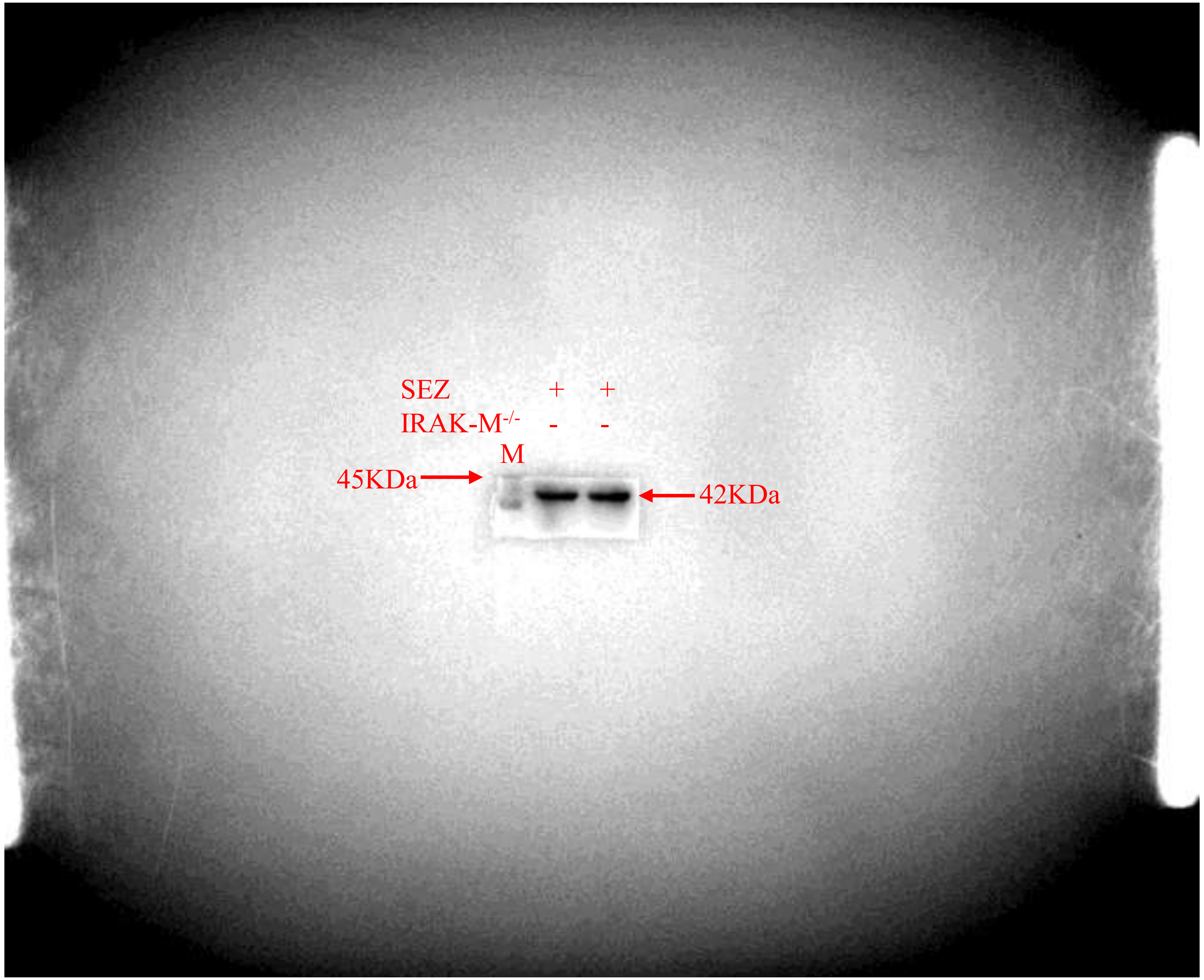

Figure 9 M β-actin (repeated 3)

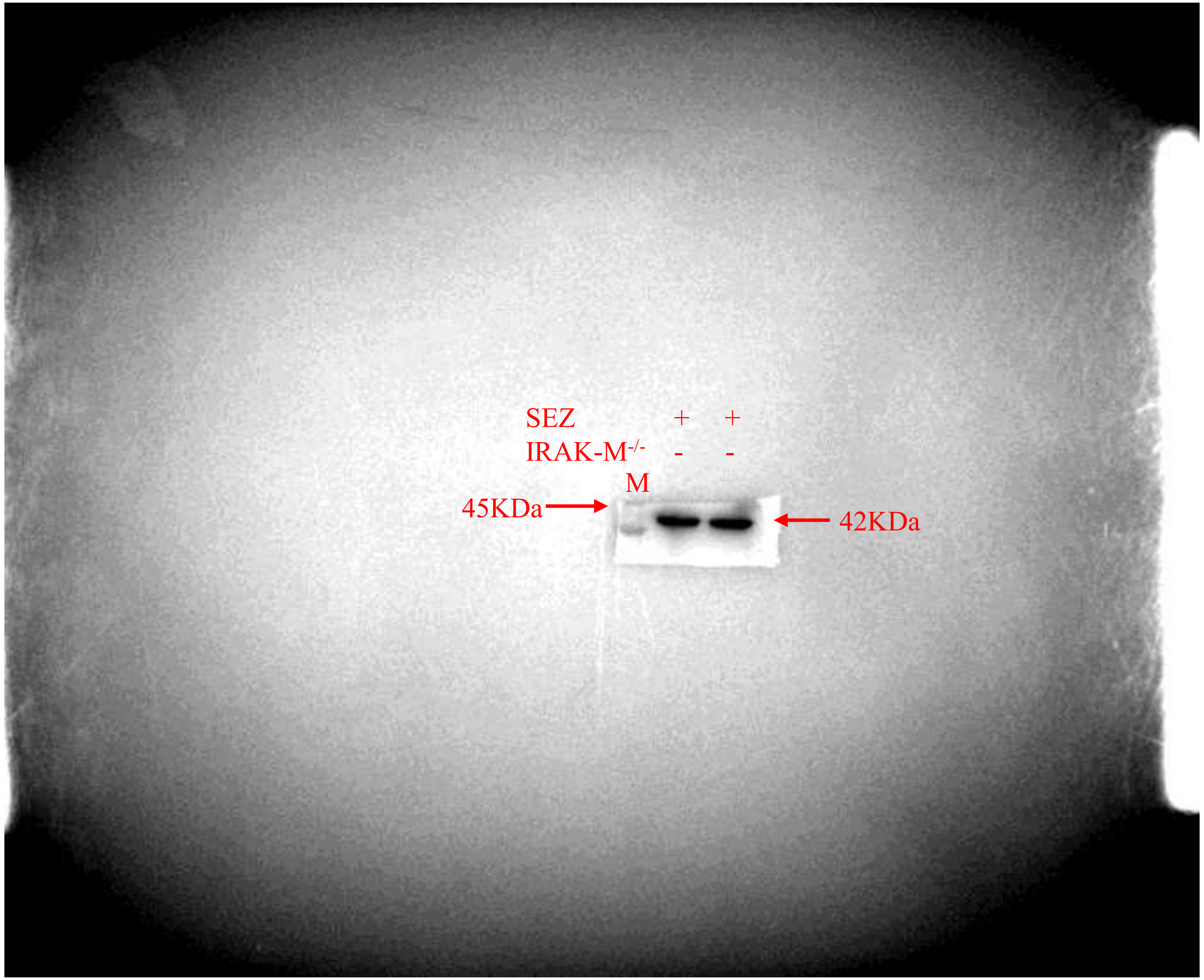

Figure 9 N GPX4 (repeated 1)

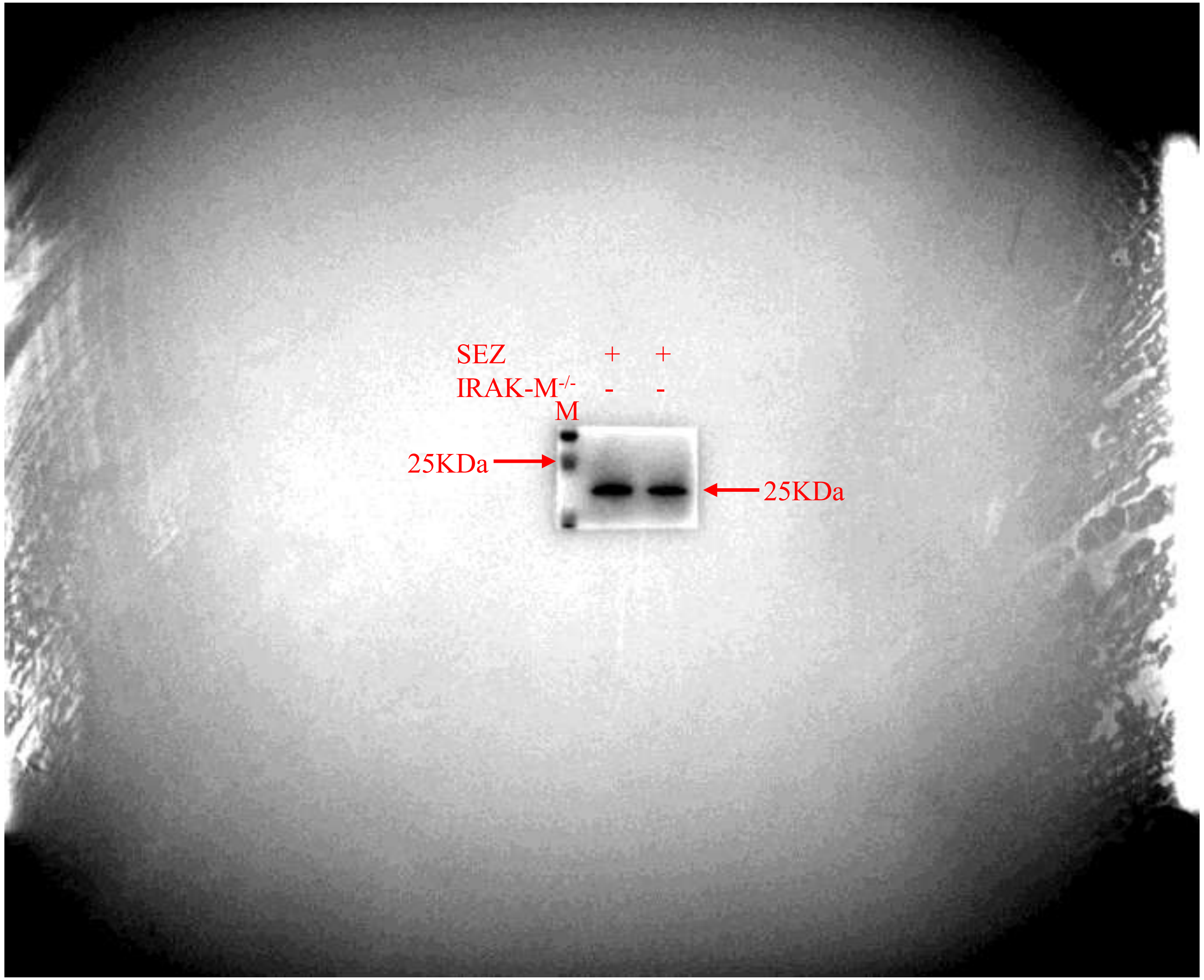

Figure 9 N GPX4 (repeated 2)

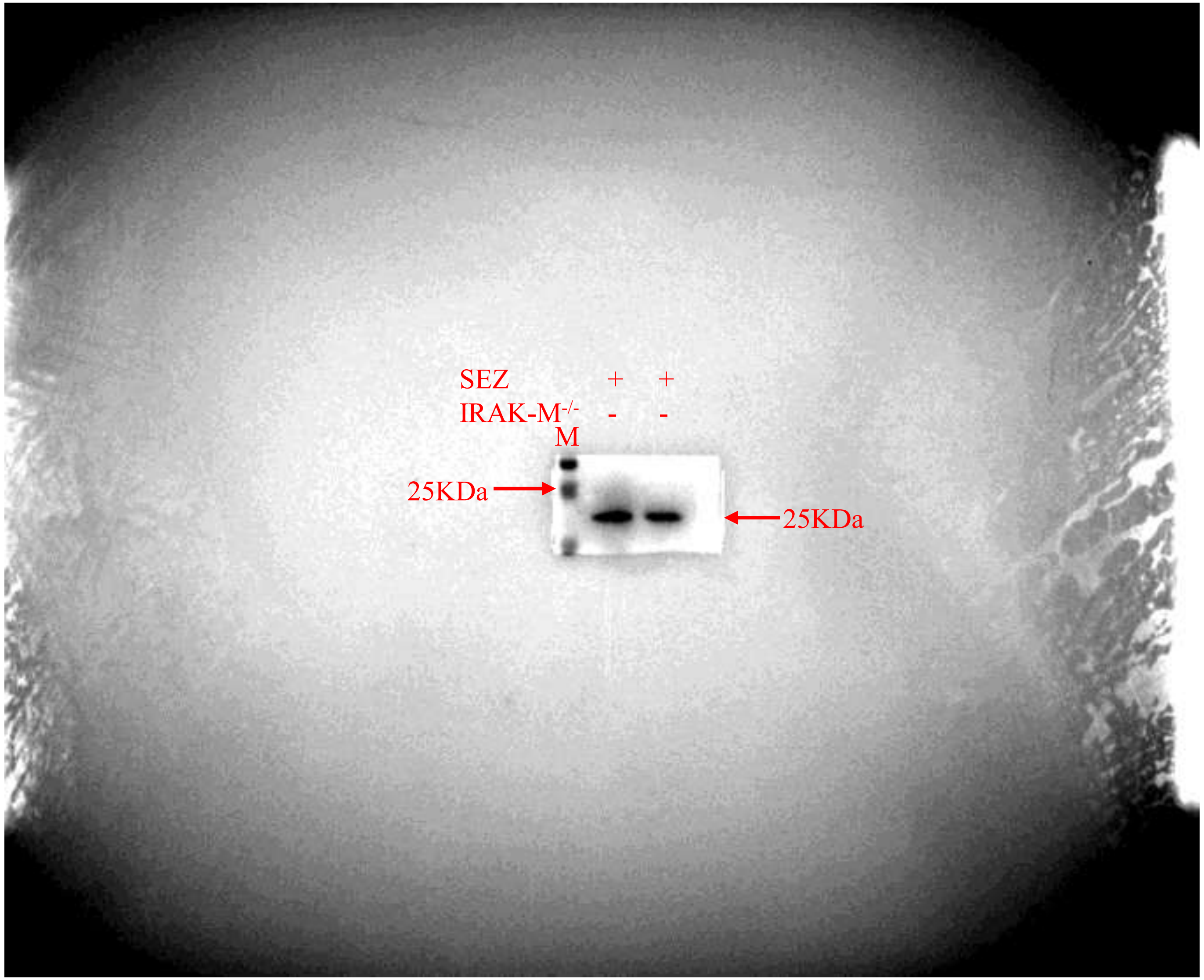

Figure 9 N GPX4 (repeated 3)

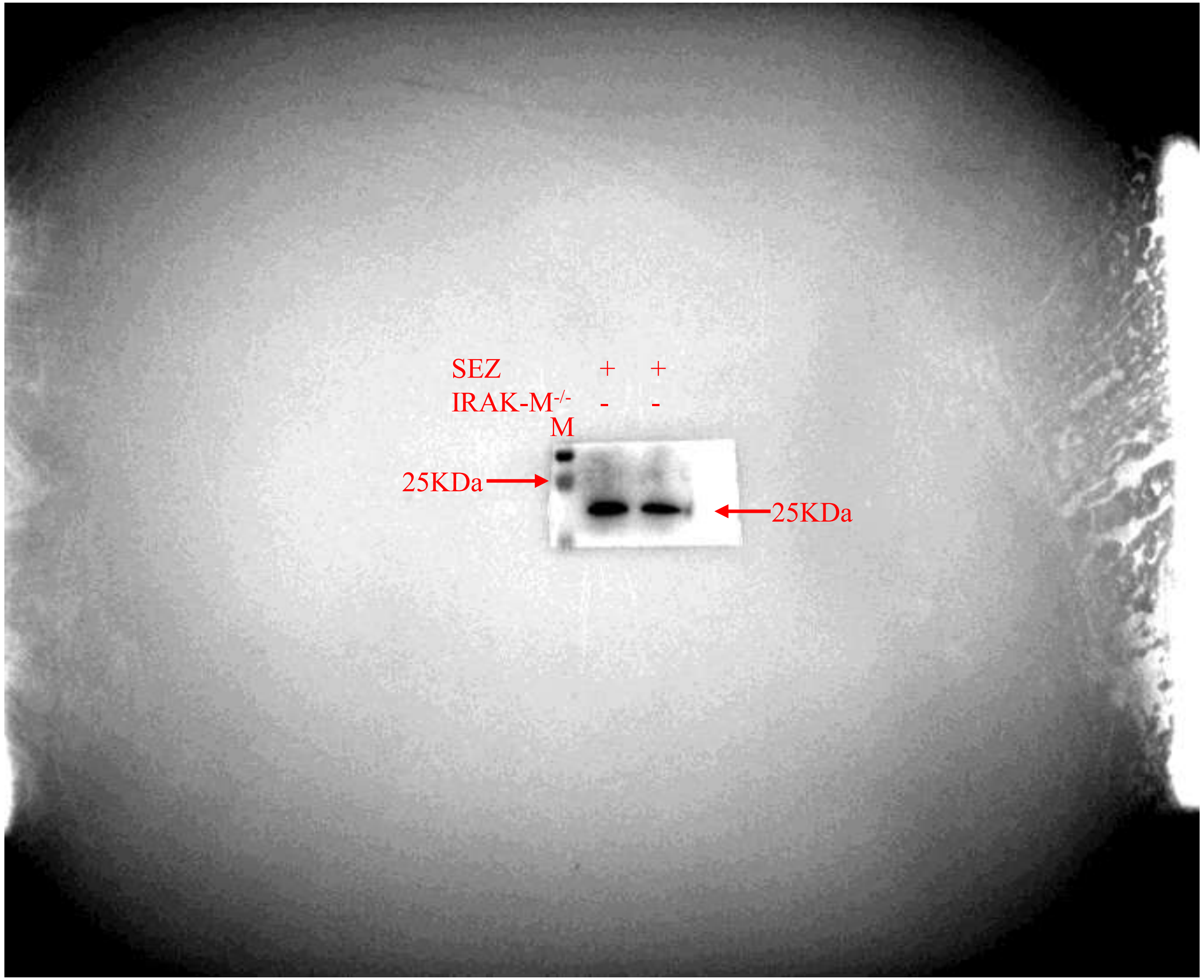

Figure 9 N HO-1 (repeated 1)

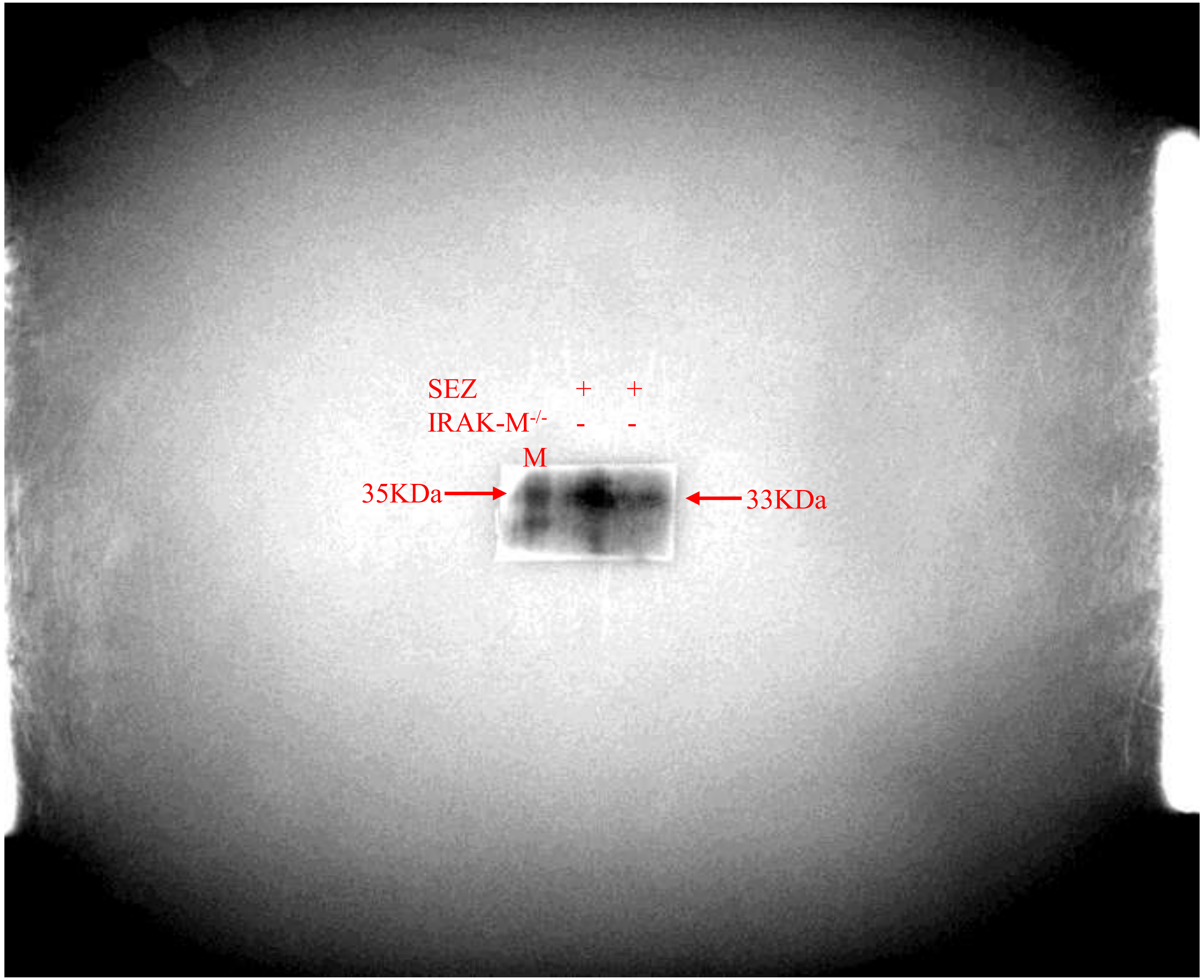

Figure 9 N HO-1 (repeated 2)

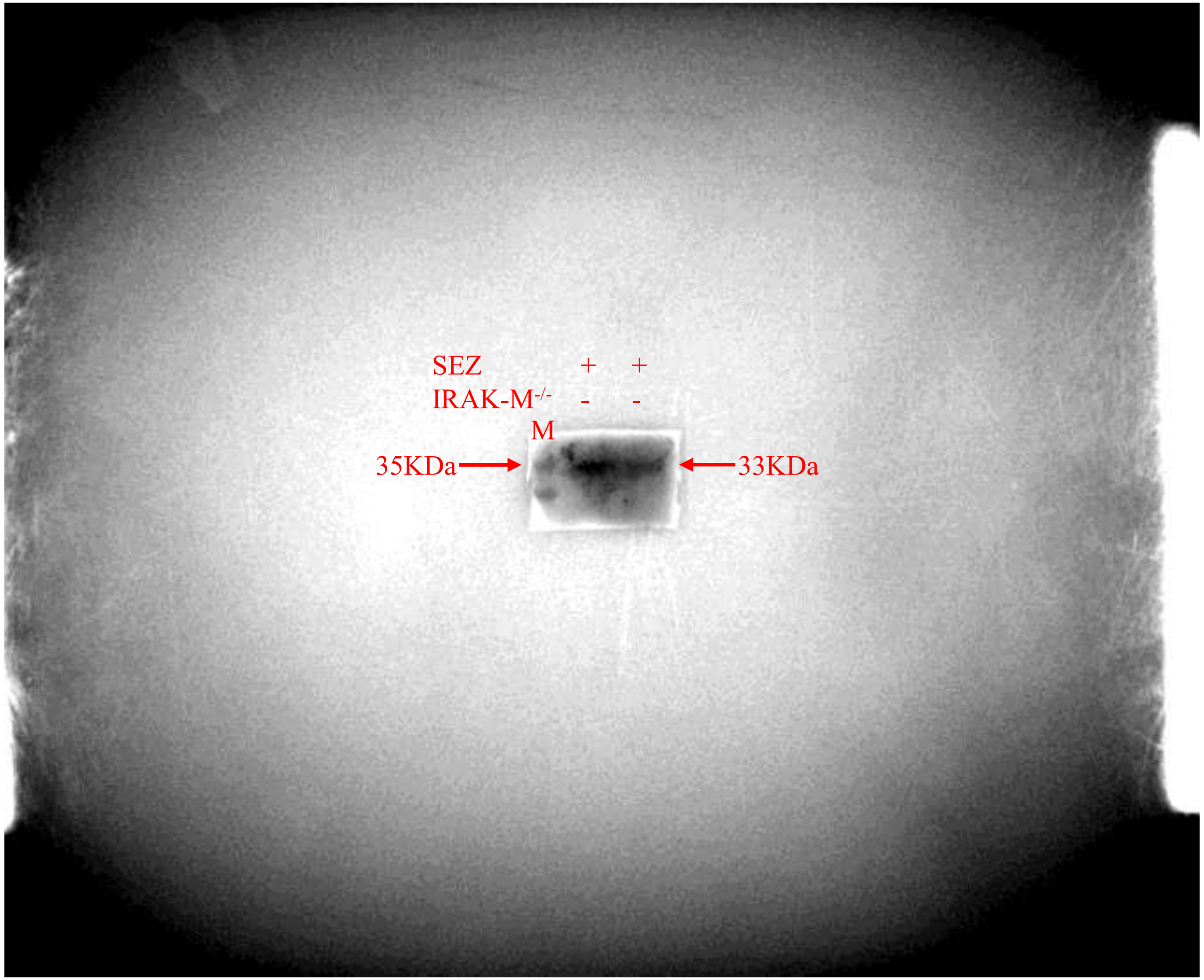

Figure 9 N HO-1 (repeated 3)

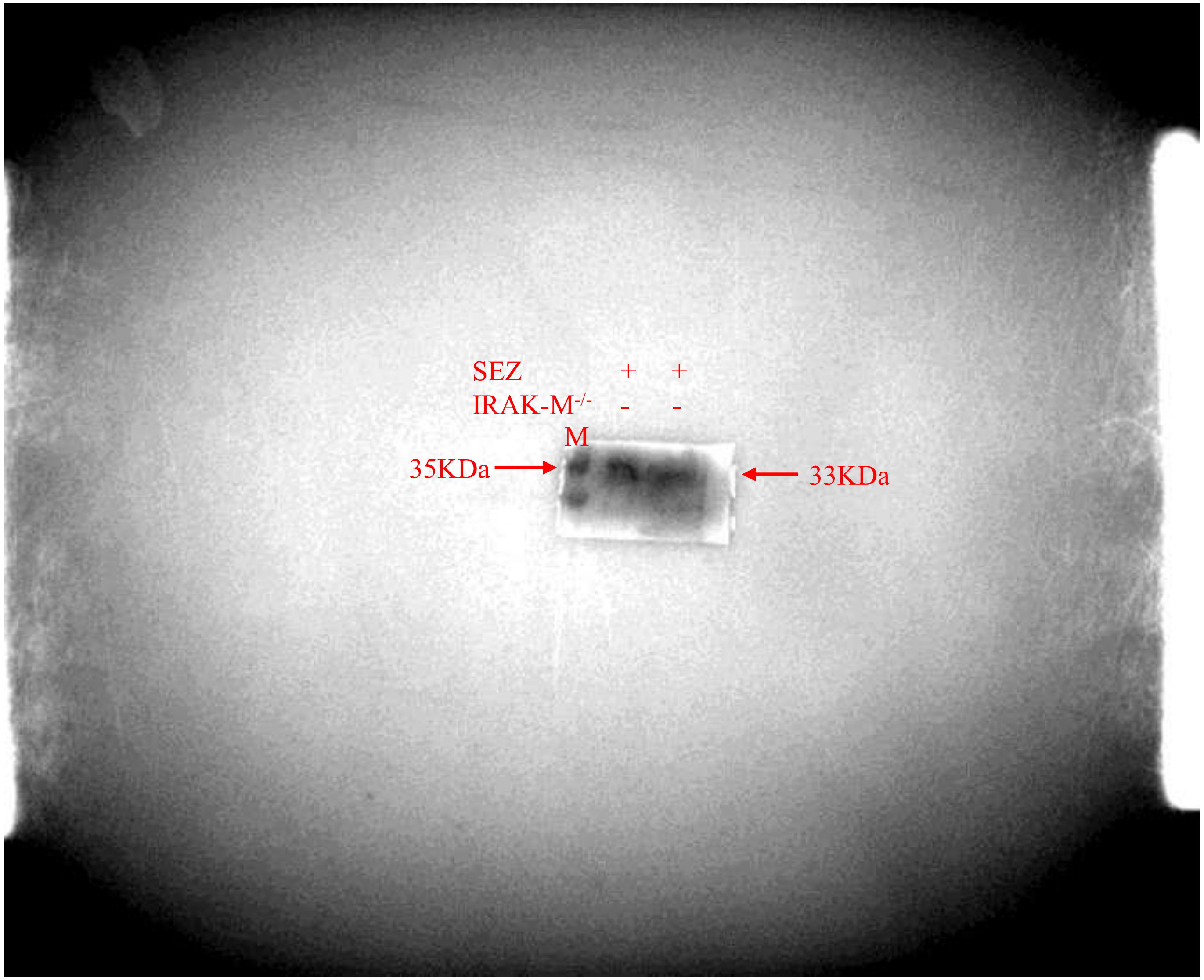

Figure 9 N iNOS (repeated 1)

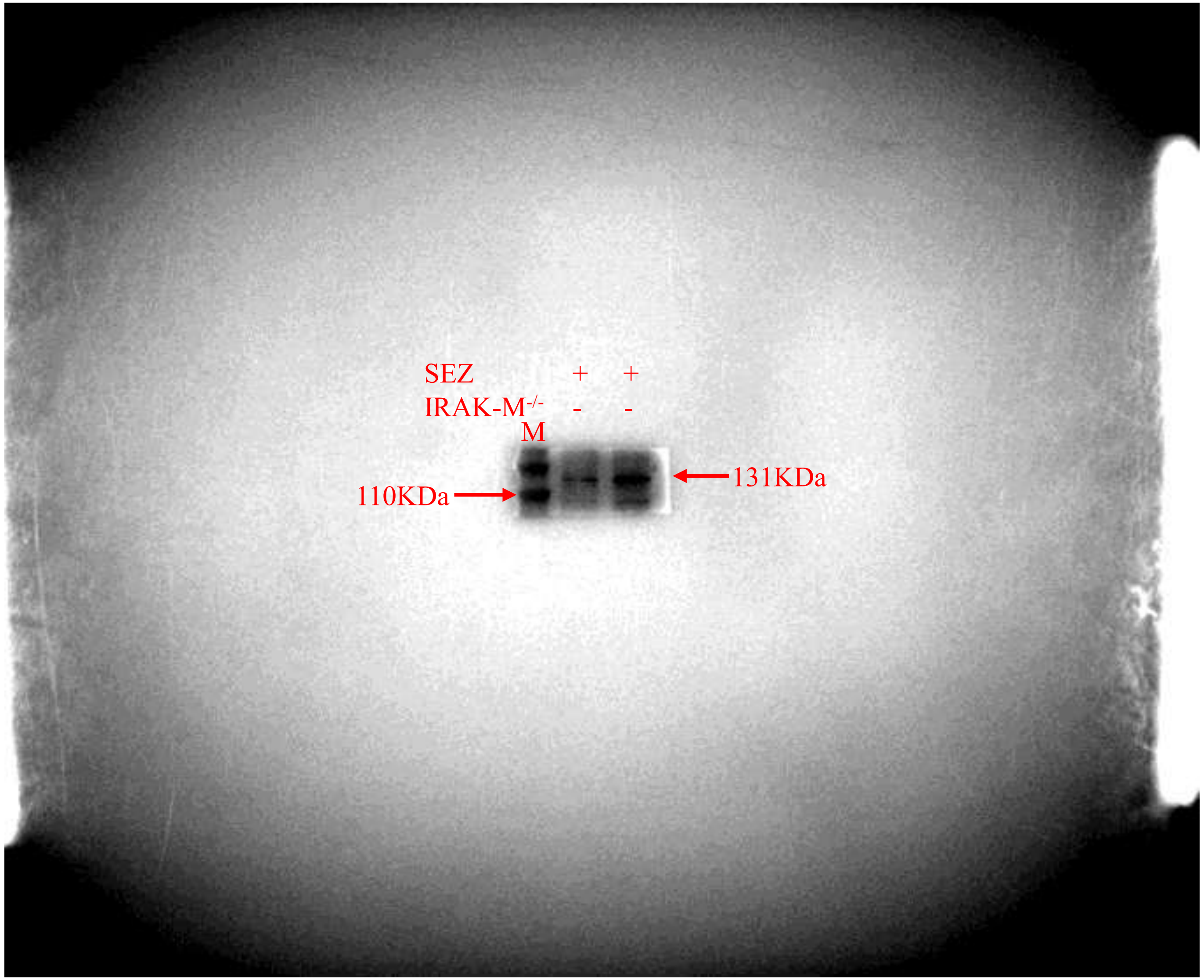

Figure 9 N iNOS (repeated 2)

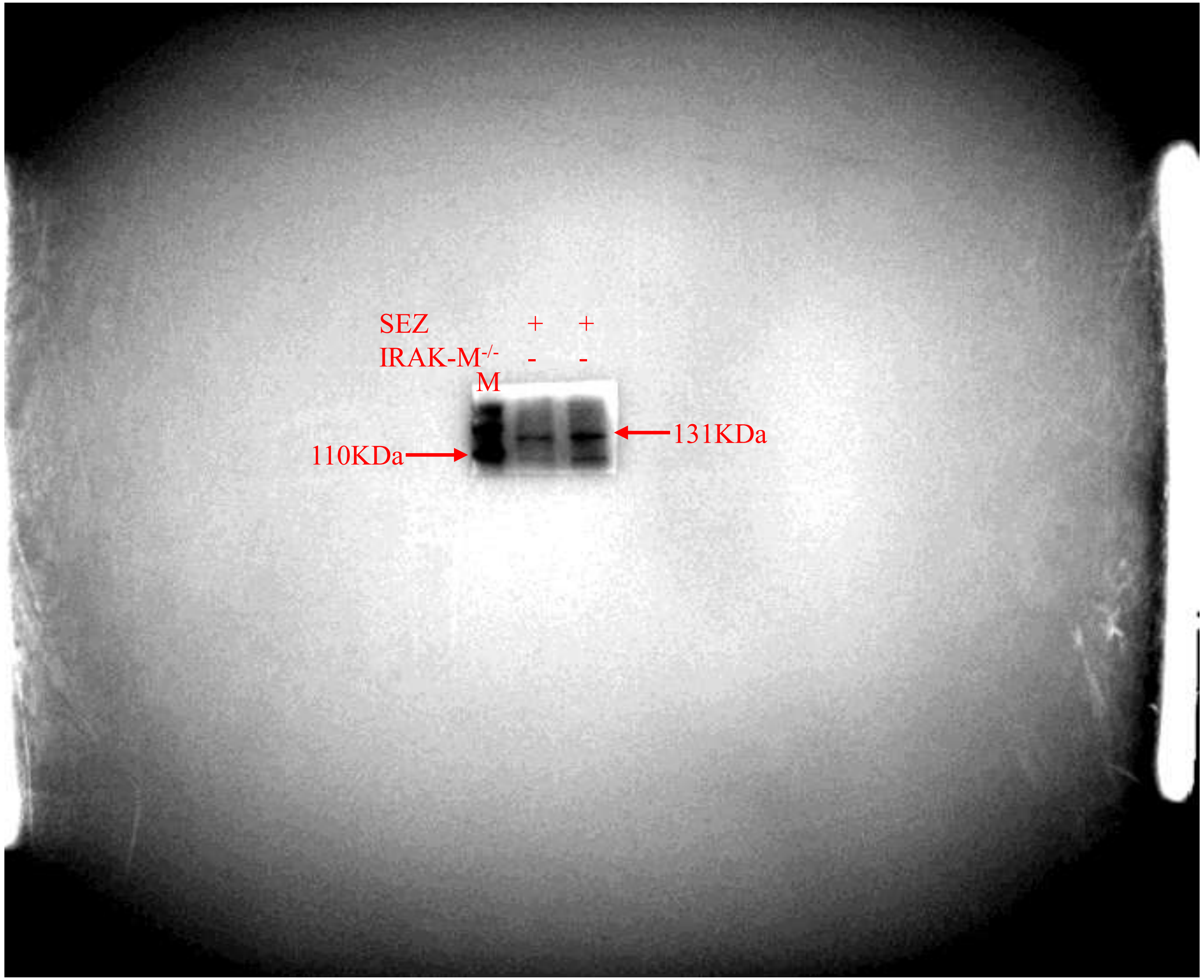

Figure 9 N iNOS (repeated 3)

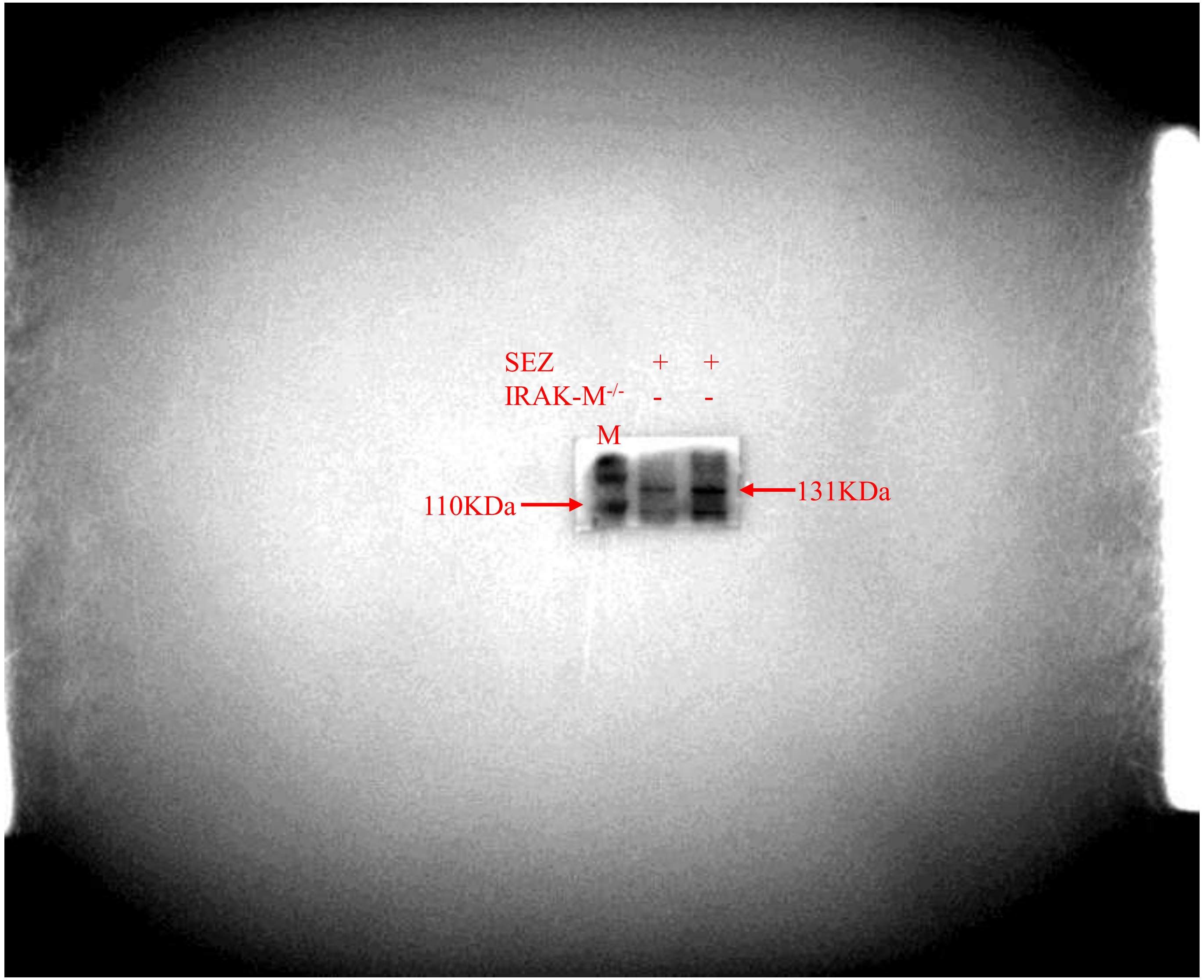

Figure 9 N NRF2 (repeated 1)

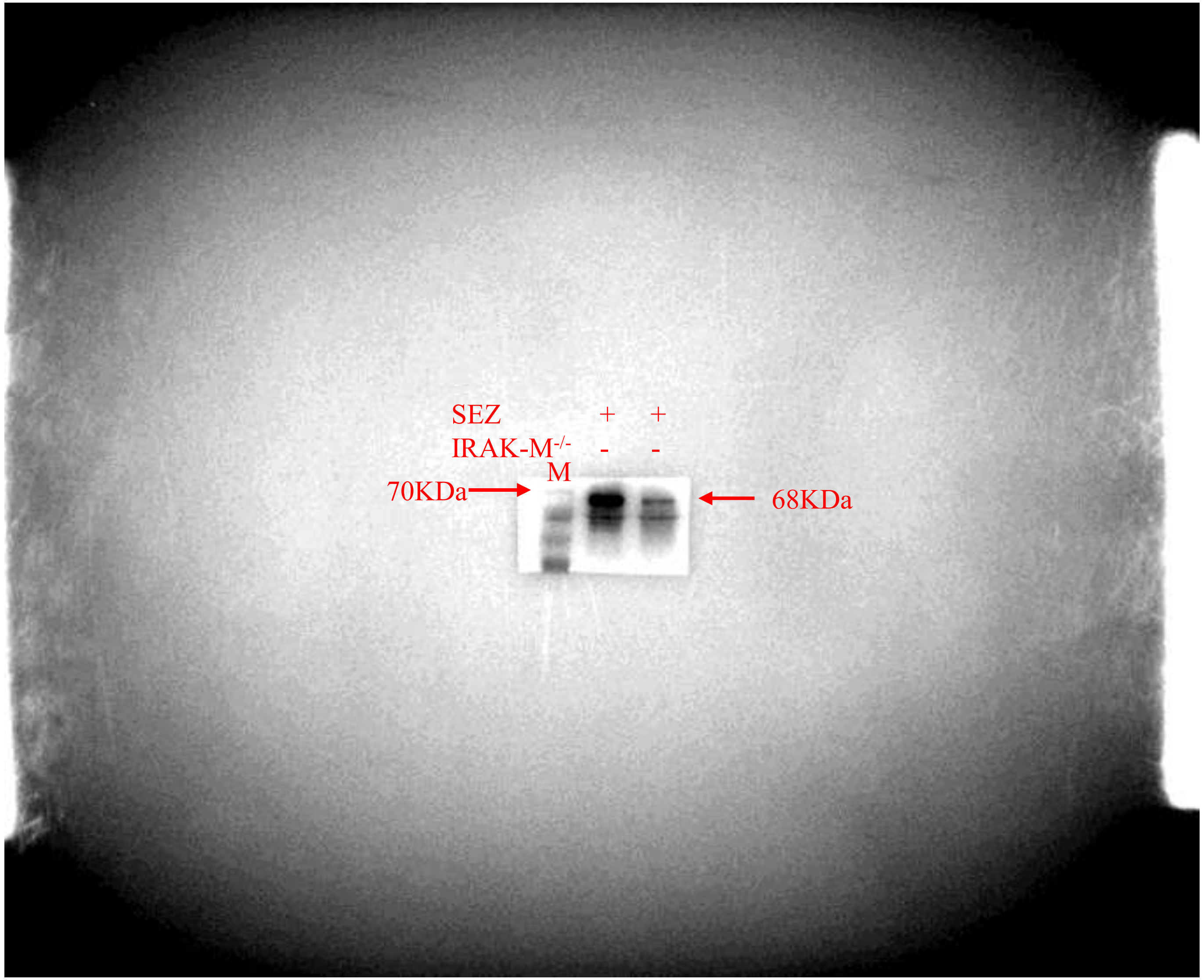

Figure 9 N NRF2 (repeated 2)

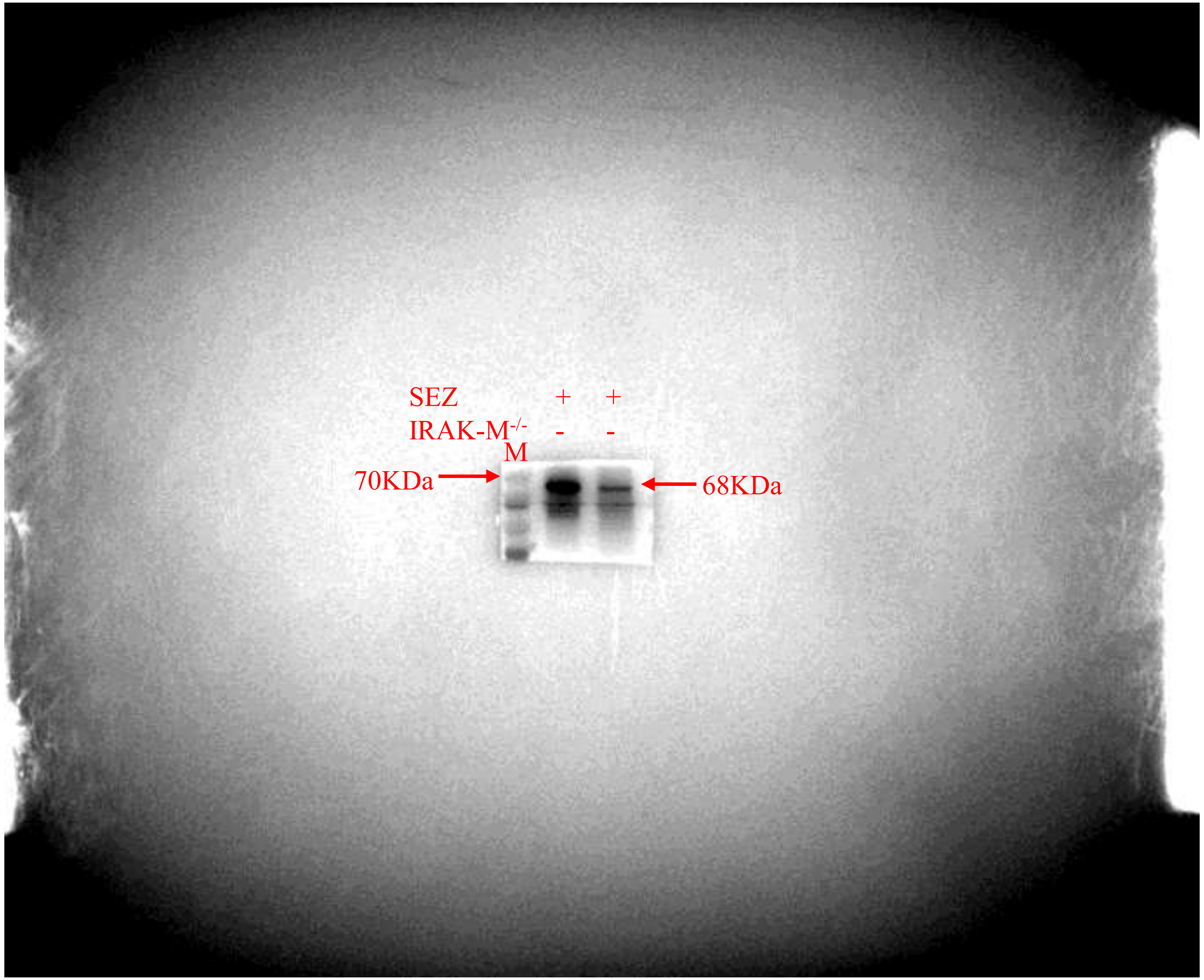

Figure 9 N NRF2 (repeated 3)

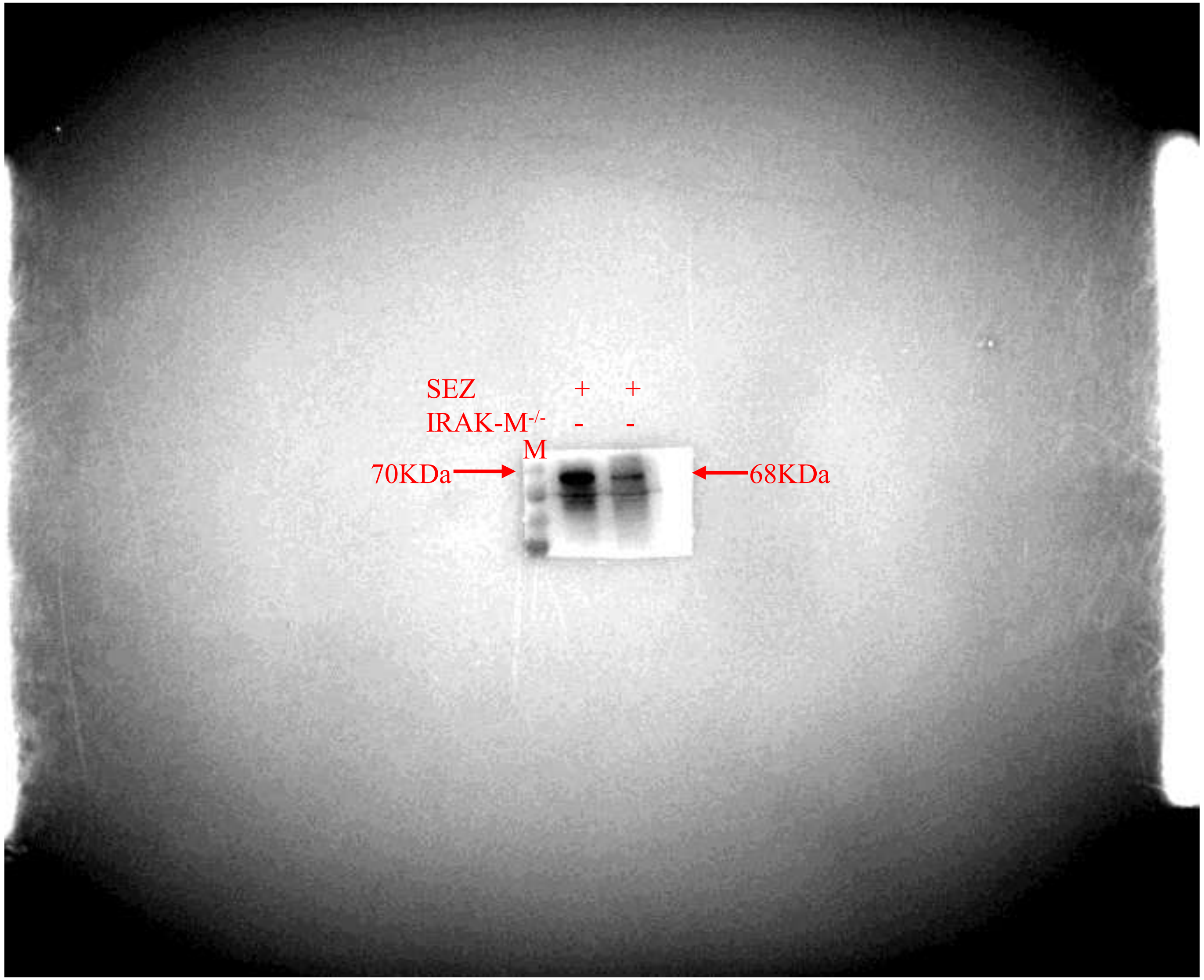

Figure 9 N β-actin (repeated 1)

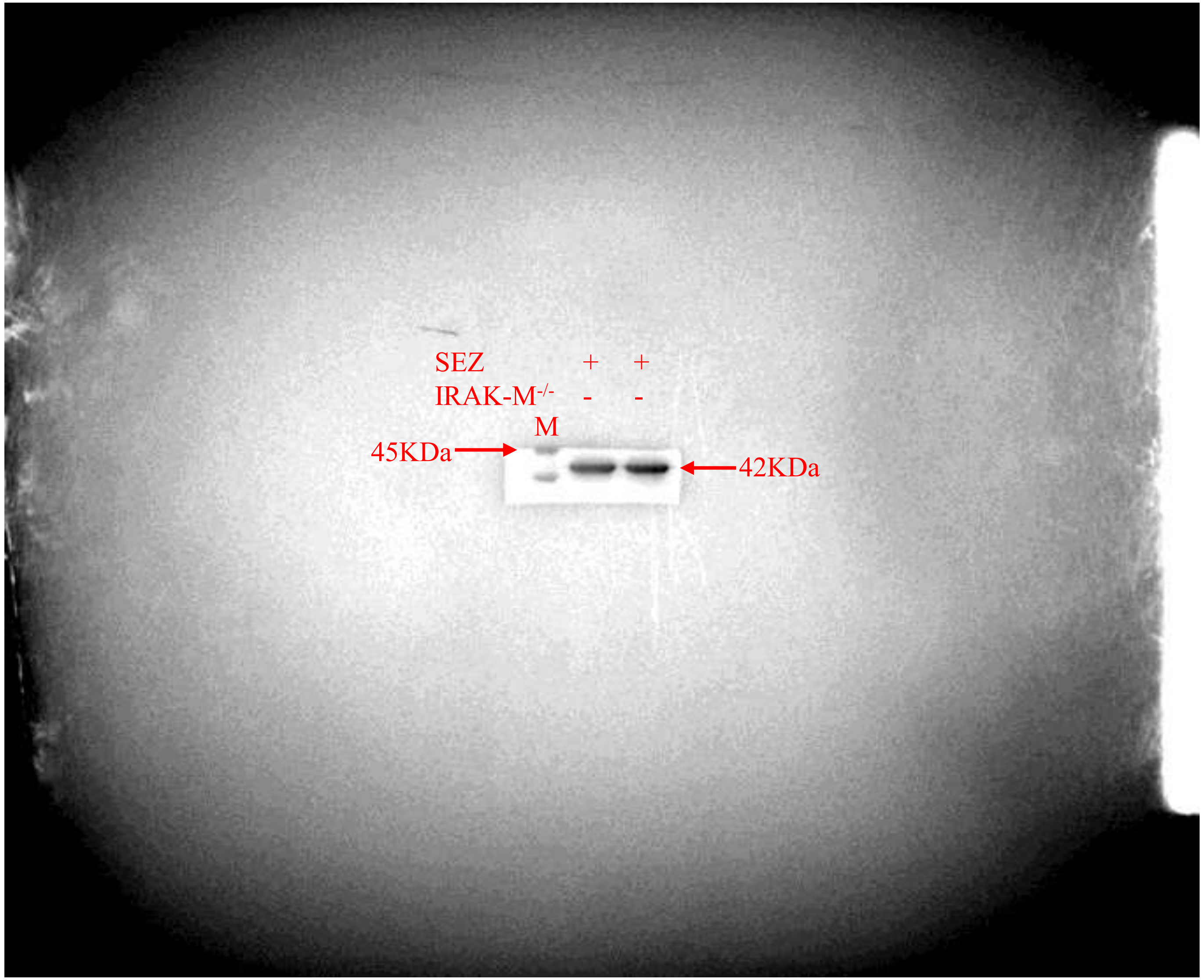

Figure 9 N β-actin (repeated 2)

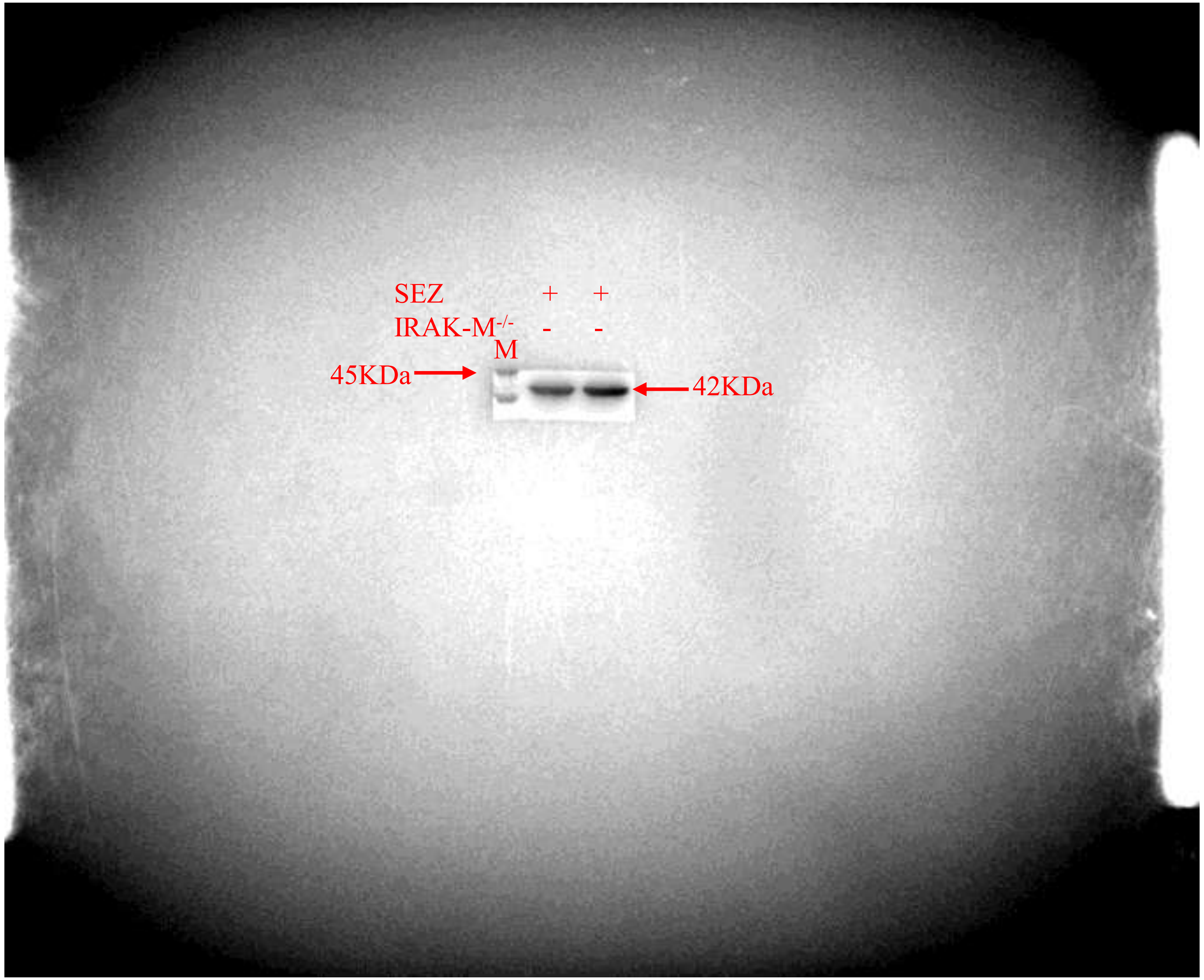

Figure 9 N β-actin (repeated 3)

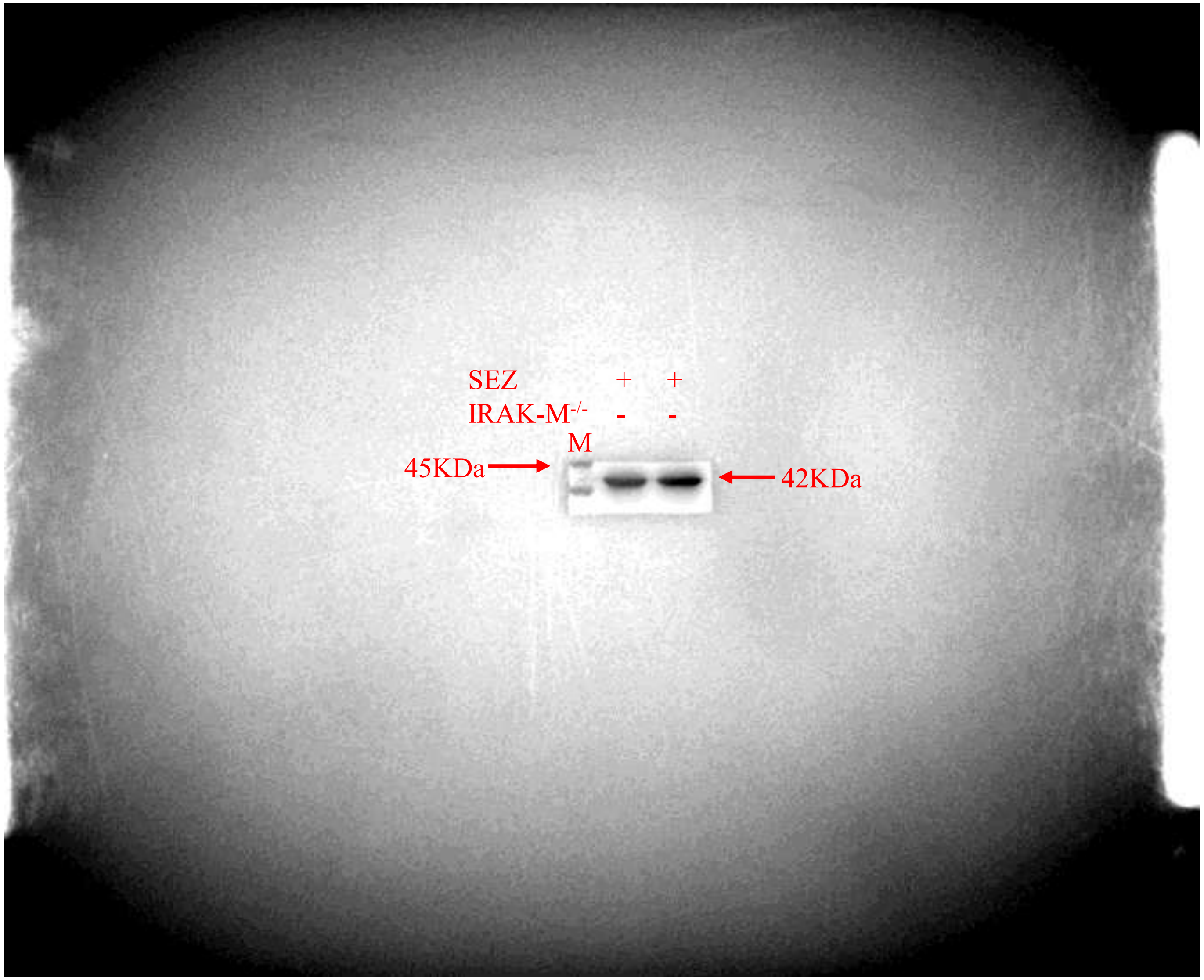

Figure 10 G NLRP3 (repeated 1)

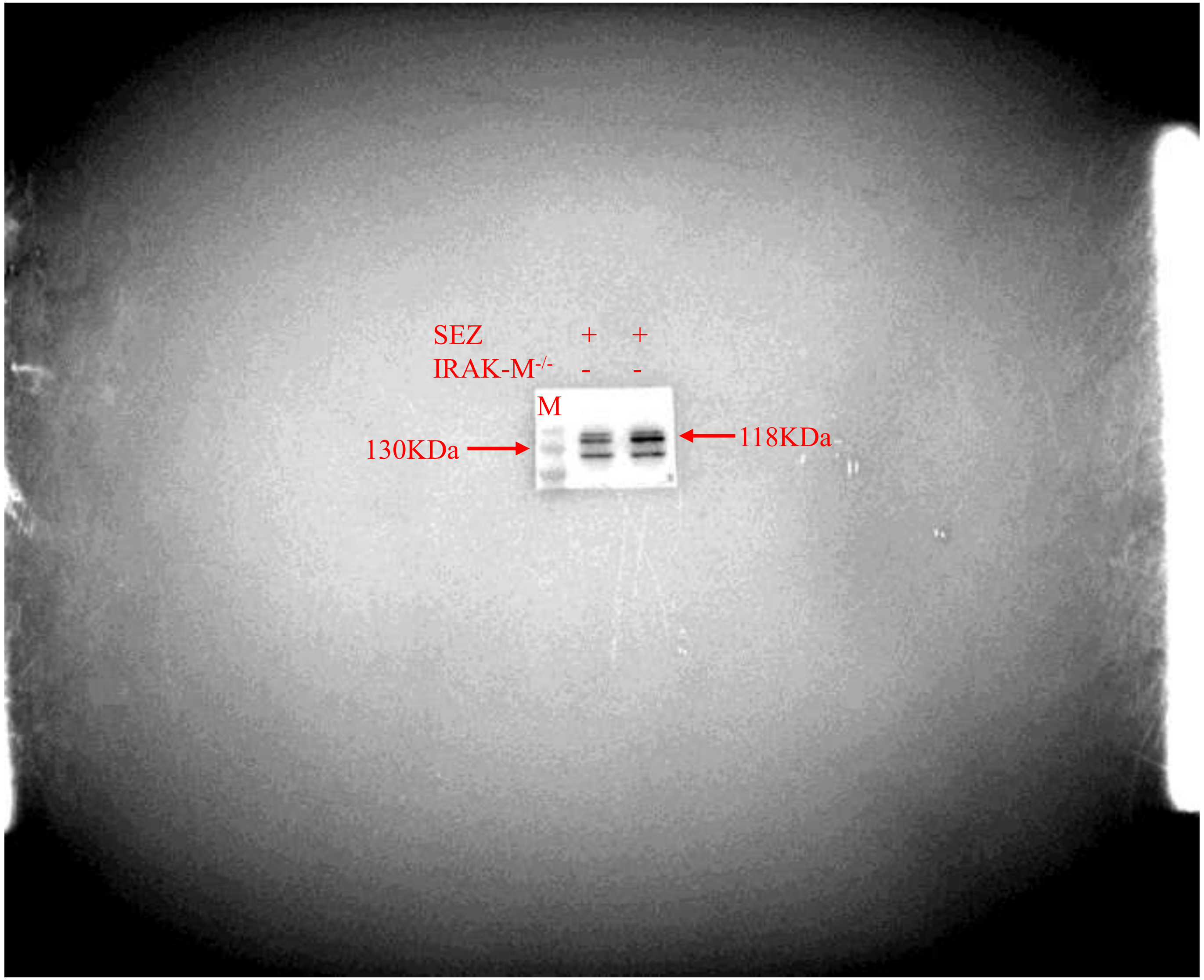

Figure 10 G NLRP3 (repeated 2)

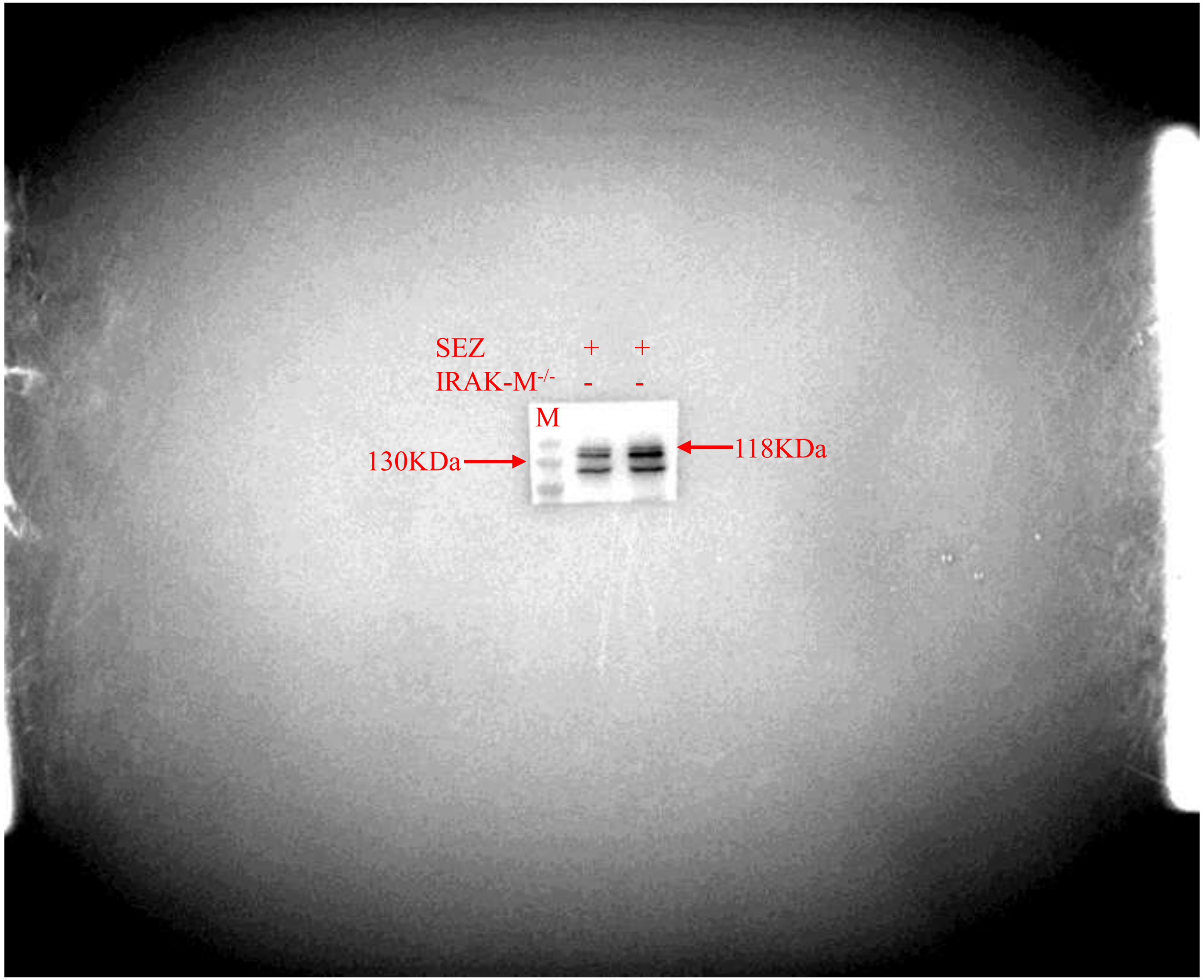

Figure 10 G NLRP3 (repeated 3)

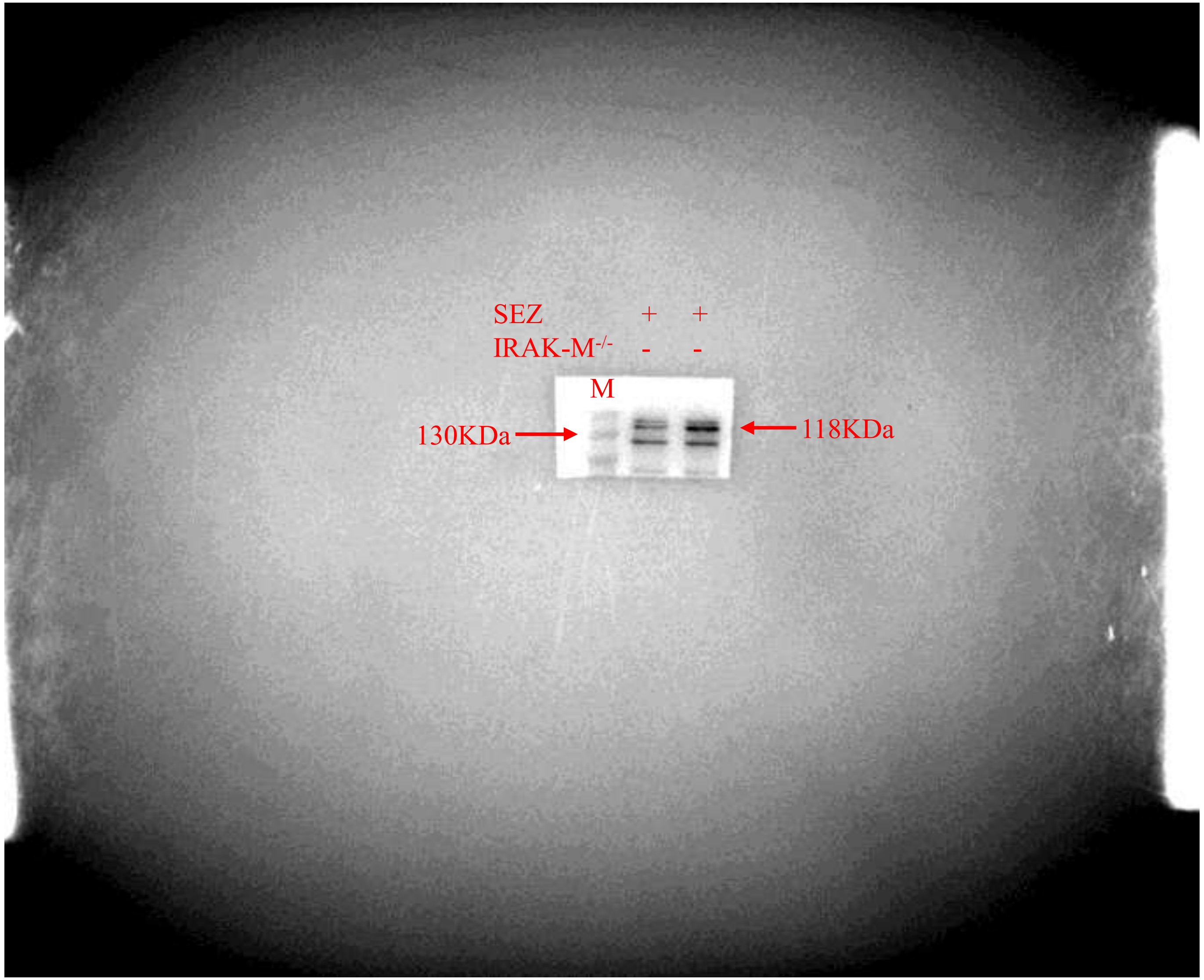

Figure 10 G caspase-1 (repeated 1)

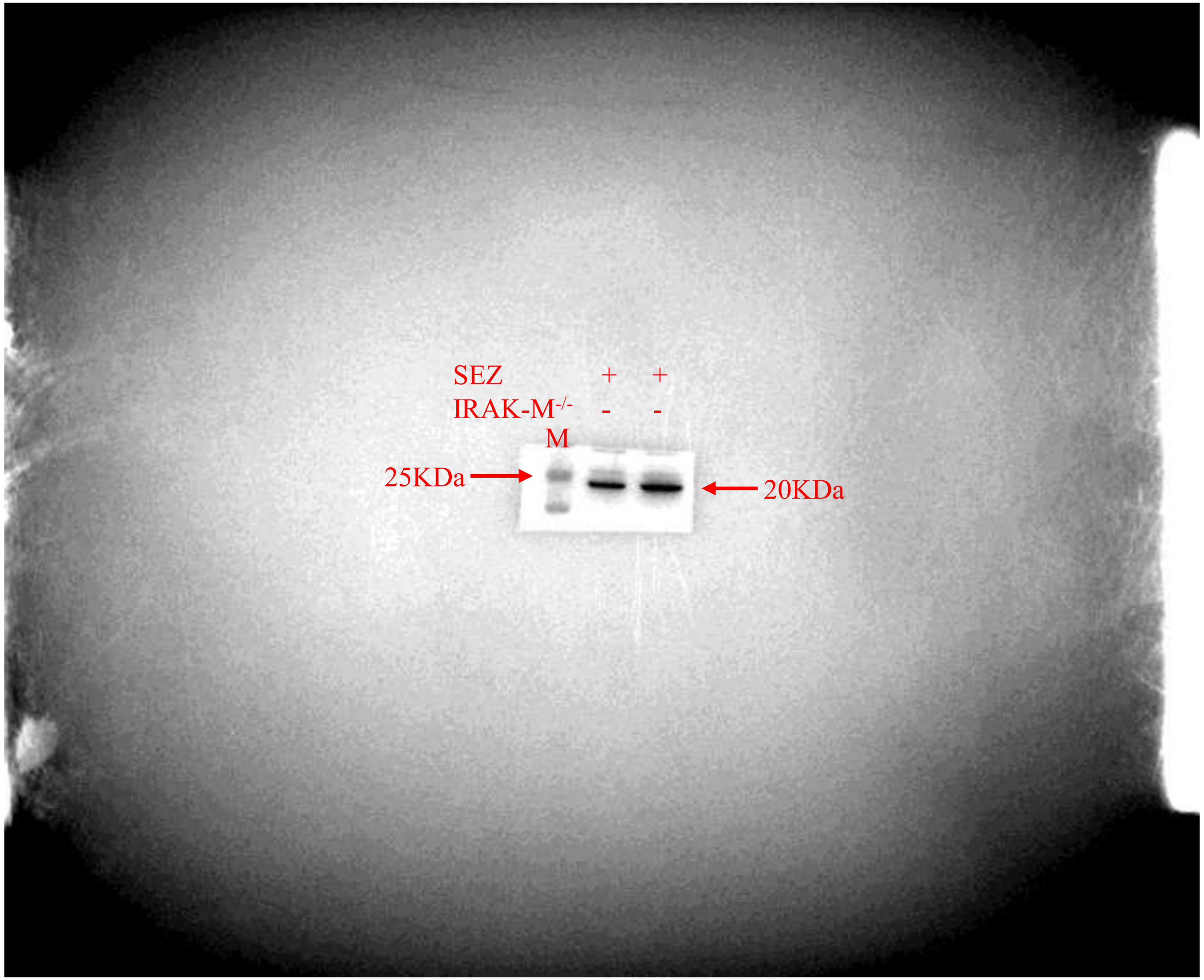

Figure 10 G caspase-1 (repeated 2)

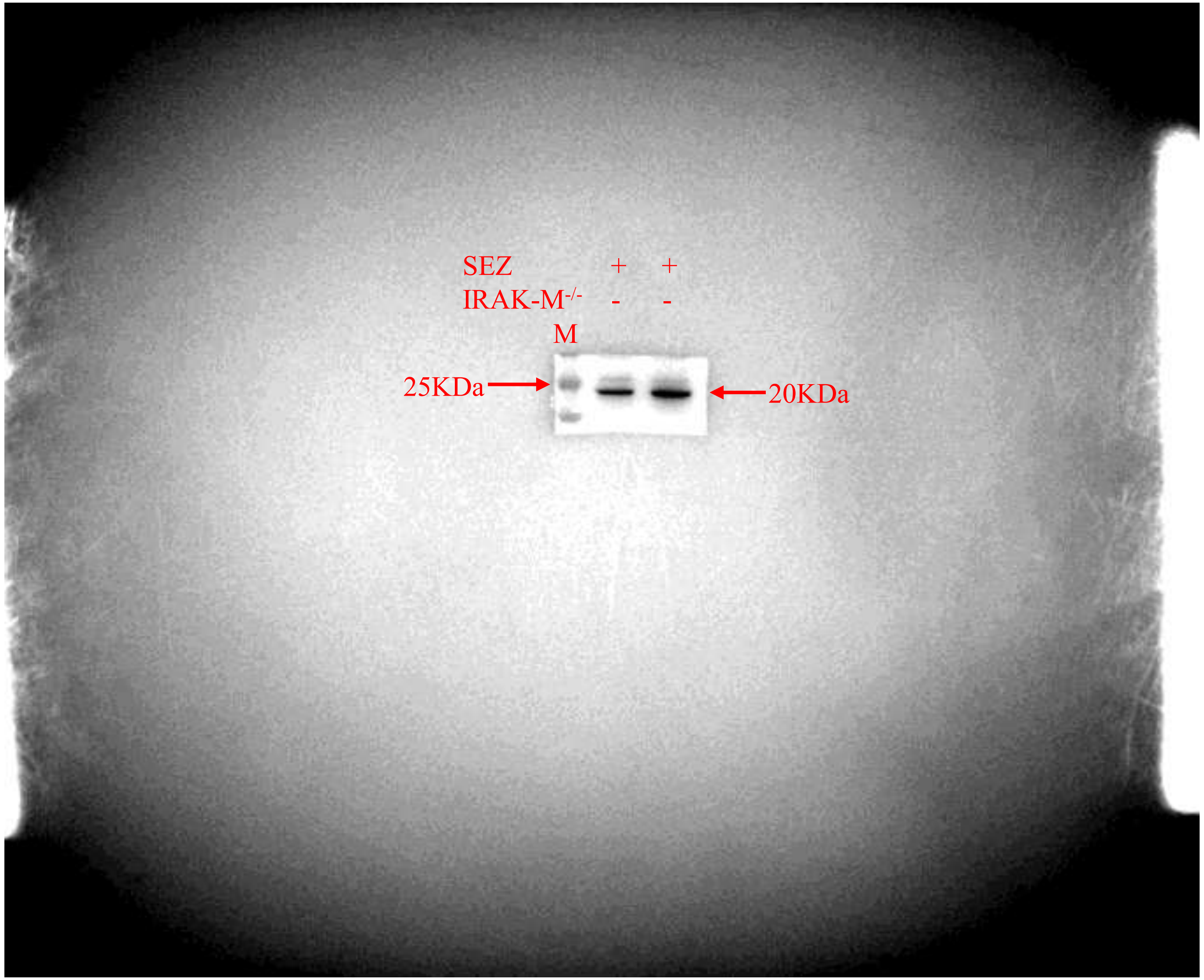

Figure 10 G caspase-1 (repeated 3)

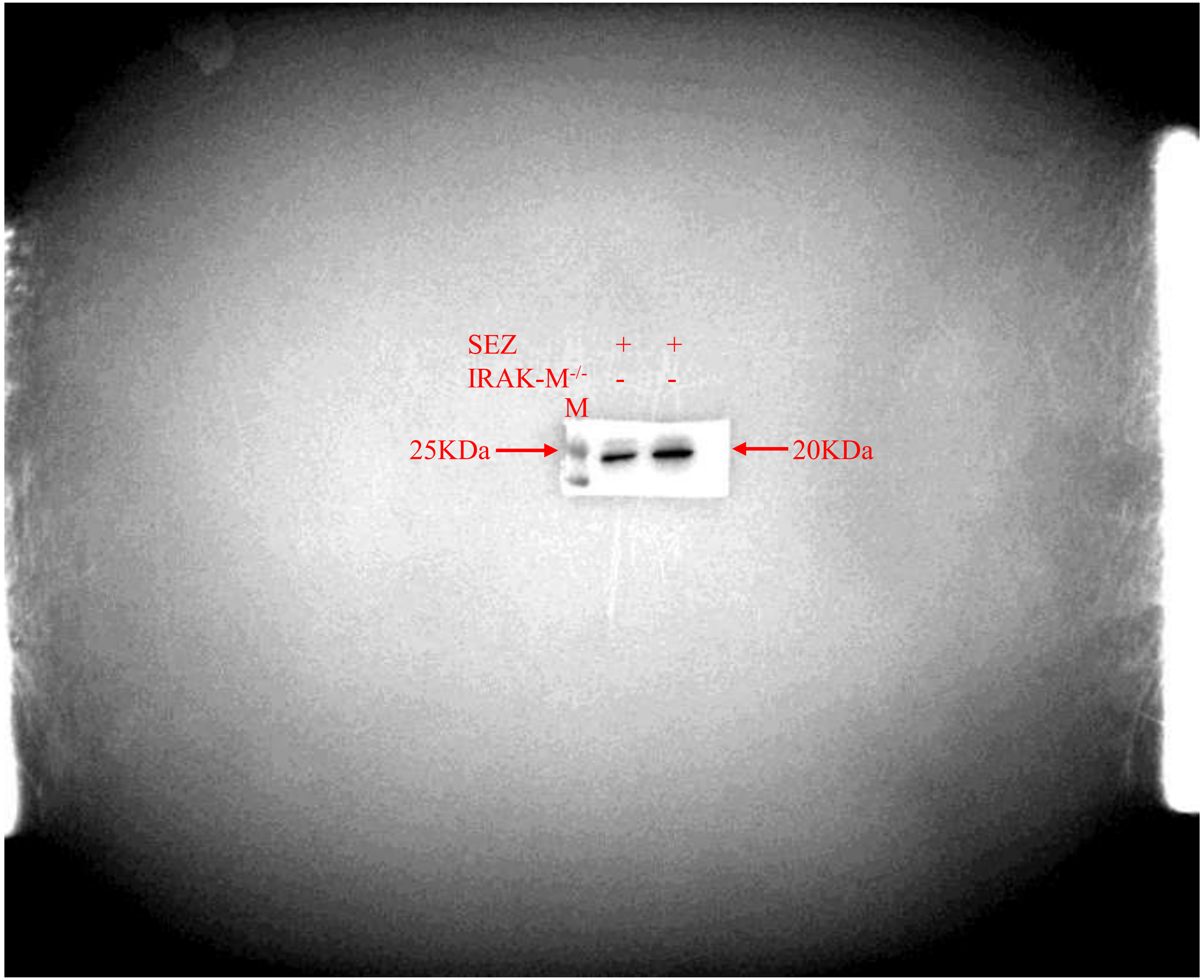

Figure 10 G GSDMD (repeated 1)

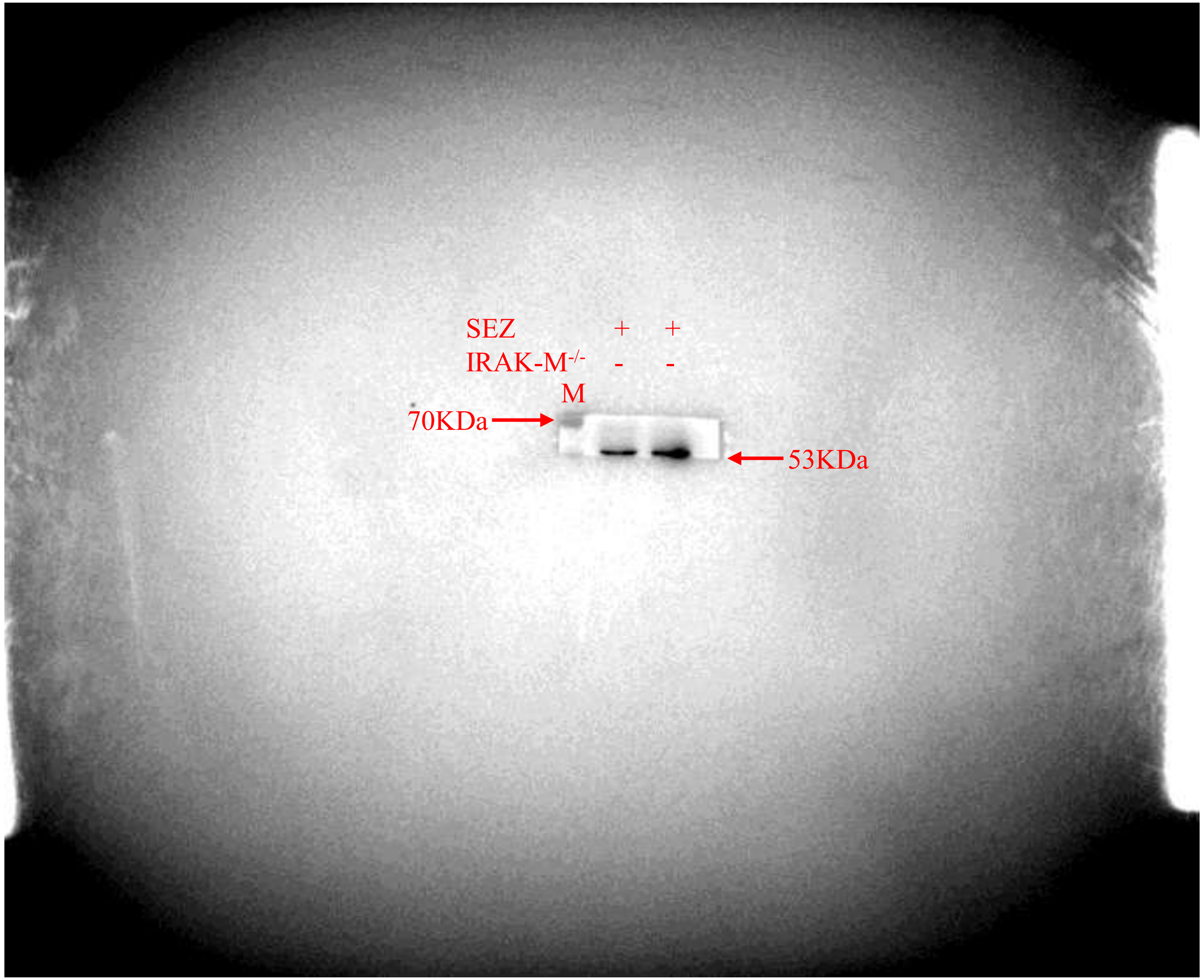

Figure 10 G GSDMD (repeated 2)

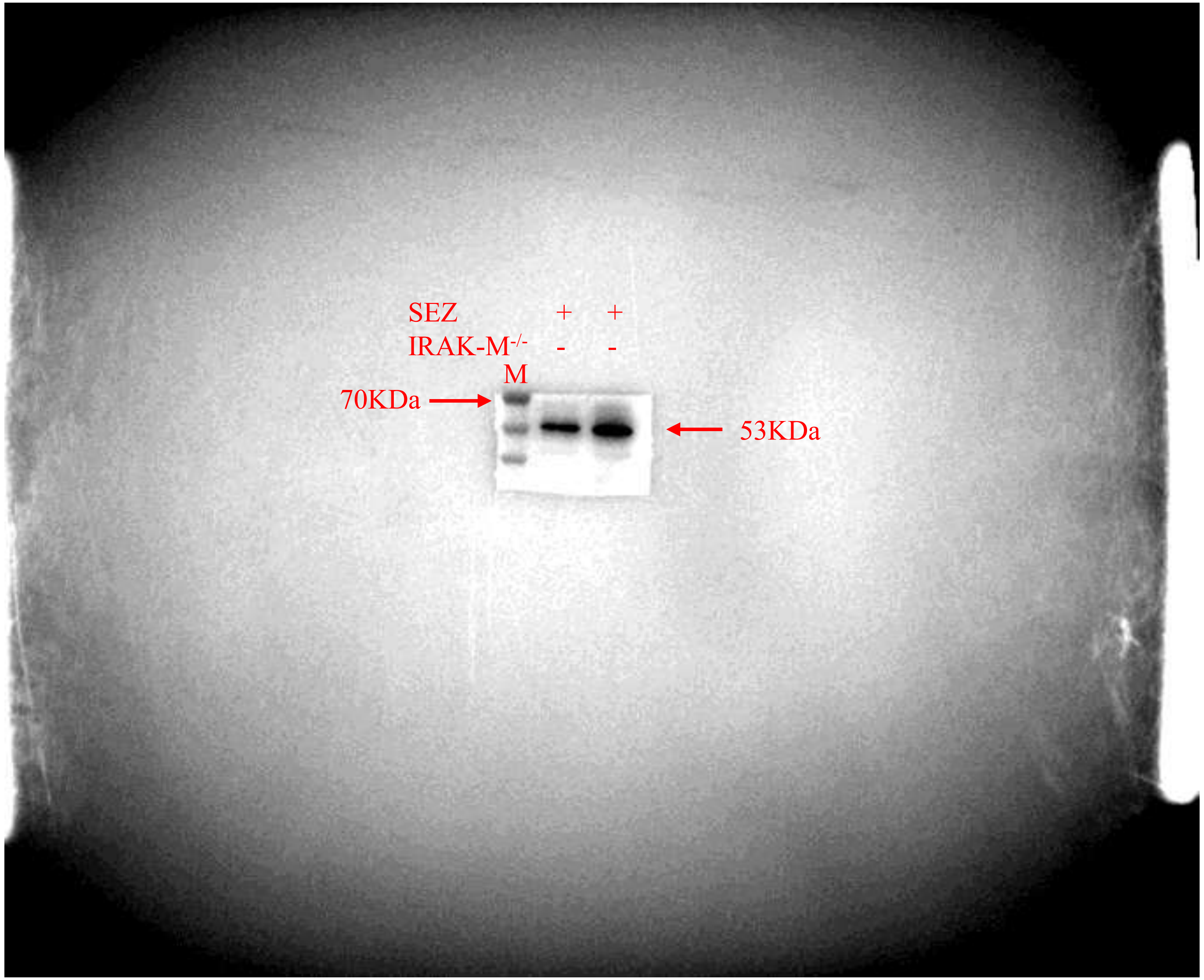

Figure 10 G GSDMD (repeated 3)

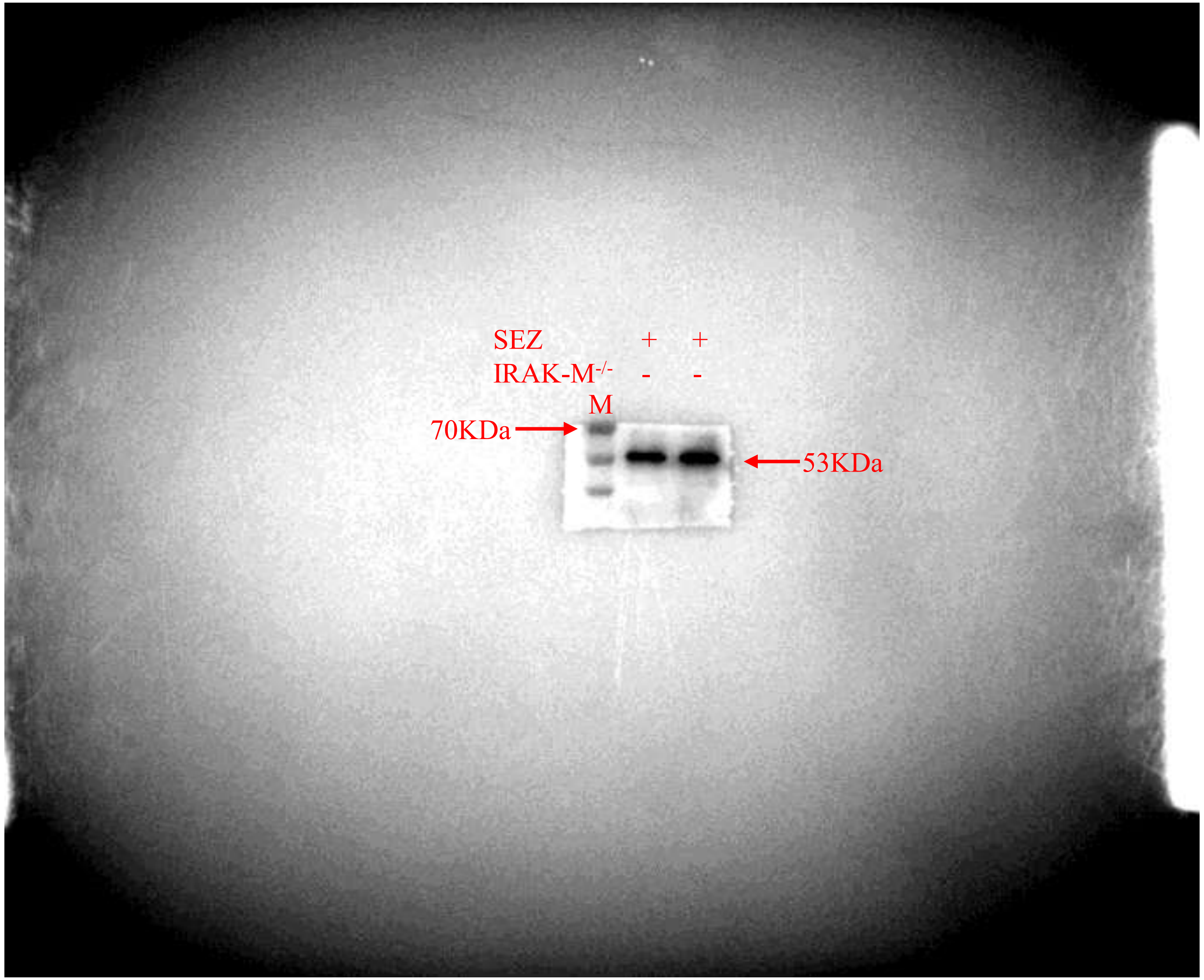

Figure 10 G β-actin (repeated 1)

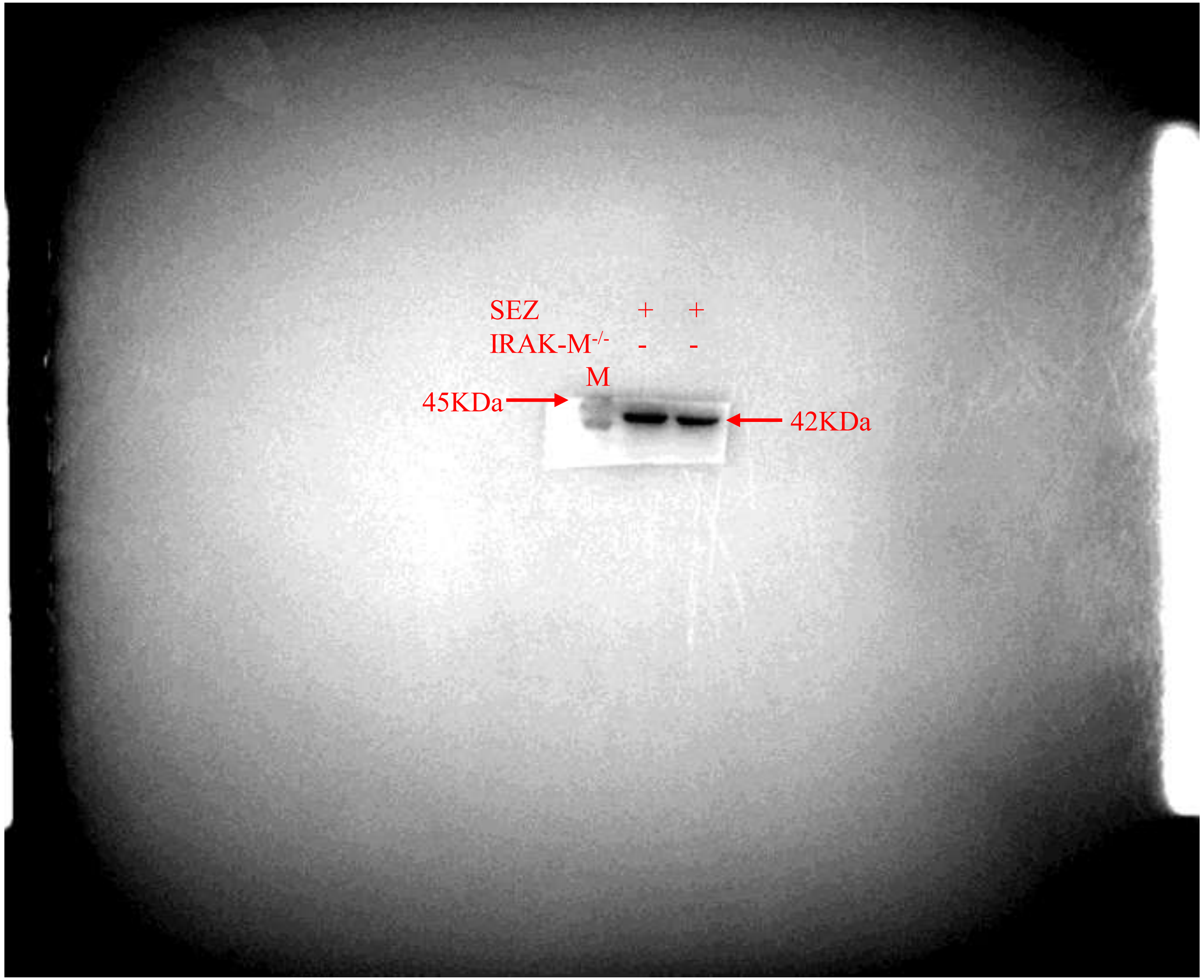

Figure 10 G β-actin (repeated 2)

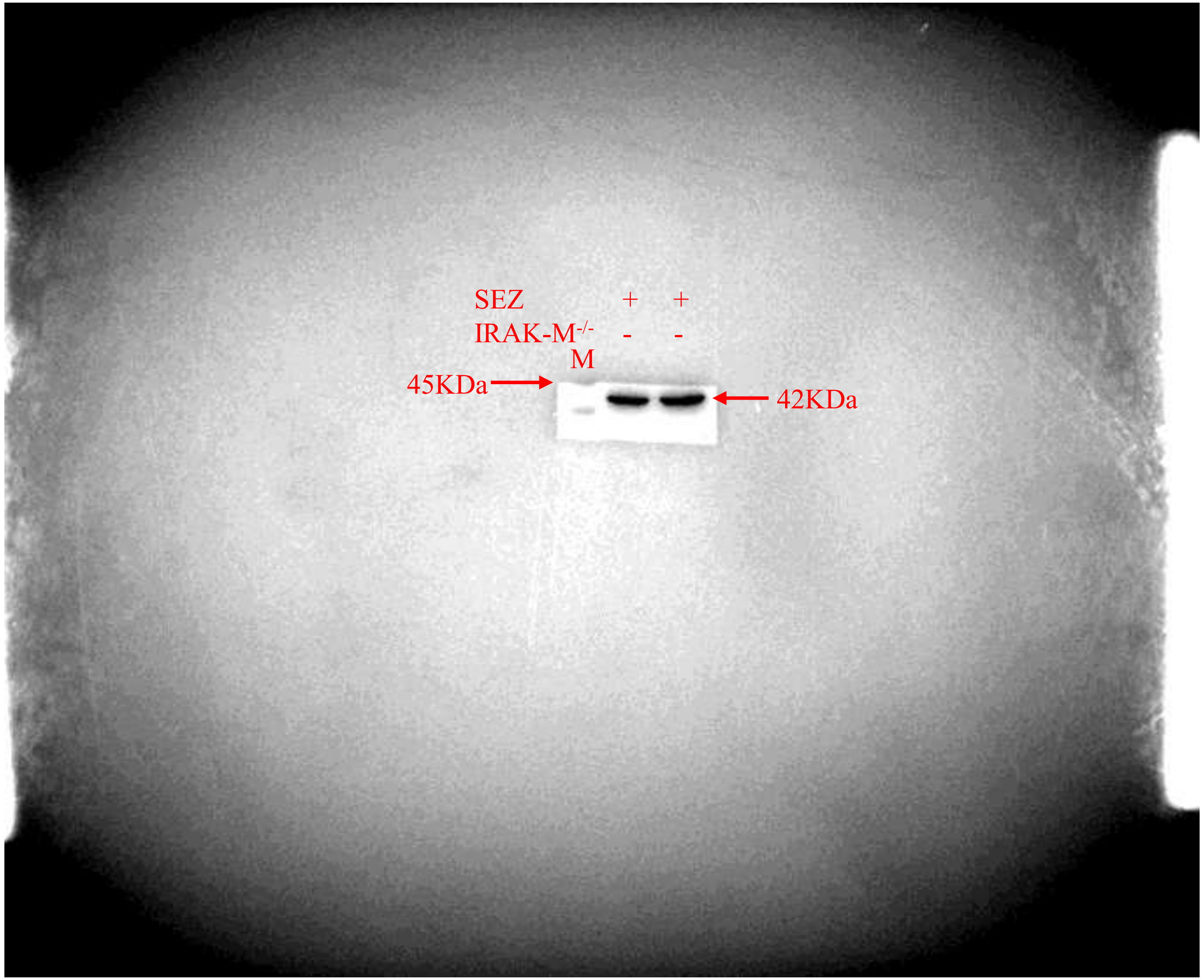

Figure 10 G β-actin (repeated 3)

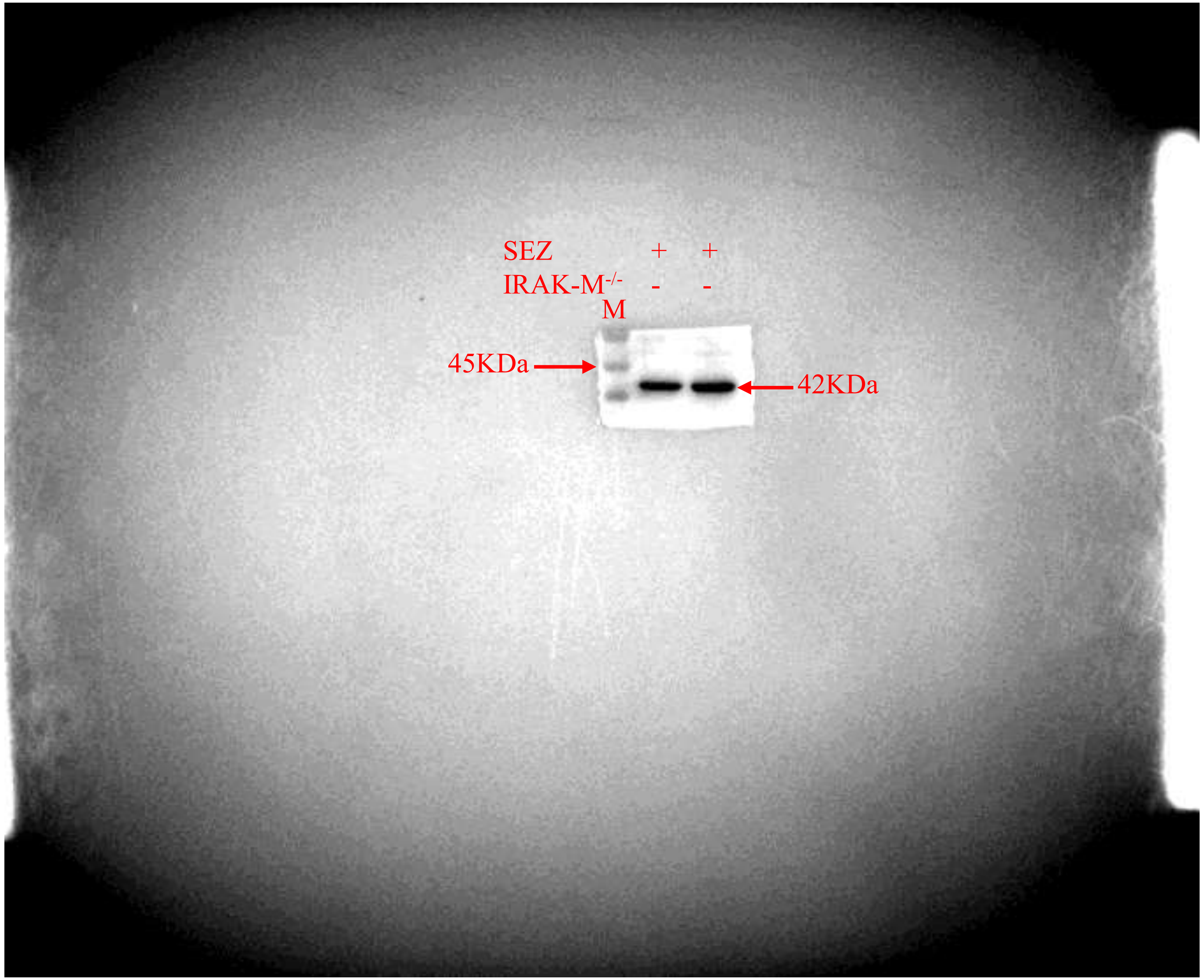

Supplement: Supplementary file 1 [file spectrum.01040-26-s0002.pdf]
