# Supplementary material for: GABABR activation alleviates Streptococcus equi subsp. zooepidemicus-induced acute lung injury by suppressing inflammation and oxidative stress in pulmonary macrophages via IRAK3
Source: Microbiol Spectr. 2026 Oct 6;14(11):e01040-26. doi: 10.1128/spectrum.01040-26 (PMC13729176; doi:10.1128/spectrum.01040-26)
Supplement: Supplementary file 2 [file spectrum.01040-26-s0001.docx]

**Supplementary materials**

**Supplementary Table 1** Specific primers used in qRT-PCR analysis.

| **Gene** | **Direction** | **Primer Sequence (5’→3’)** |
| --- | --- | --- |
| CXCL9 | Forward  Reverse | TCTGCCATGAAGTCCGCTG  CCGGATCTAGGCAGGTTTGA |
| CXCL10 | Forward  Reverse | AAGTGCTGCCGTCATTTTCT  GTGGCAATGATCTCAACACG |
| GM-CSF | Forward  Reverse | GAAGGGCATGTTTAACGACA  ACTGAGCAAACTATTACGGTGG |
| IL-1β | Forward  Reverse | GCTGCTTCCAAACCTTTGAC  AGCTTCTCCACAGCCACAAT |
| IL-6 | Forward  Reverse | TAGTCCTTCCTACCCCAATTTCC  TTGGTCCTTAGCCACTCCTTC |
| Il-17A | Forward  Reverse | TTTAACTCCCTTGGCGCAAAA  CTTTCCCTCCGCATTGACAC |
| MCP-1 | Forward  Reverse | GGTCCCTGTCATGCTTCTGG  CCTGCTGCTGGTGATCCTCT |
| TNF-α | Forward  Reverse | CCGATGGGTTGTACCTTGTC  AGATAGCAAATCGGCTGACG |
| Claudin-1 | Forward  Reverse | GCTGGGTTTCATCCTGGCTTCTC  CCTGAGCGGTCACGATGTTGTC |
| Claudin-2 | Forward  Reverse | GTCATCGCCCATCAGAAGAT  ACTGTTGGACAGGGAACCAG |
| Occludin | Forward  Reverse | ATGTCCGGCCGATGCTCTC  TTTGGCTGCTCTTGGGTCTGTAT |
| ZO-1 | Forward  Reverse | CCACCTCTGTCCAGCTCTTC  CACCGGAGTGATGGTTTTCT |
| CAT | Forward  Reverse | TTCCTGAGCAAGCCTTC  CACATGAATGGCTATGGATCACA |
| GPX4 | Forward  Reverse | TTCTCAGCCAAGGACATCG  CACTCAGCATATCGGGCAT |
| GSH | Forward  Reverse | CCGTGGAAGATTTGAAGATGTC  CTGCTGCACCTTCTTAGTCCC |
| GSH-Px | Forward  Reverse | GTCCACCGTGTATGCCTTCT  TCTGCAGATCGTTCATCTCG |
| HO-1 | Forward  Reverse | CGCCTCCAGAGTTTCCGCAT  GACGCTCCATCACCGGACTG |
| iNOS | Forward  Reverse | AGGTTGTCTGCATGGACCAG  GCTGGGACAGTCTCCATTCC |
| NRF2 | Forward  Reverse | CAGTGCTCCTATGCGTGAA  GCGGCTTGAATGTTTGTC |
| SOD | Forward  Reverse | GAGACCTGGGCAATGTGACTG  CGCAATCCCAATCACTCCACA |
| IRAK-M | Forward  Reverse | CAGGTGTCCTTCTCCACTGTTCTTG  GTGACCTCAGACTGGCTGCA |
| NLRP3 | Forward  Reverse | CCAGGAGTTCTTCGCTGCTATGTAC  CCTTGGCTTTCACTTCAATCCAC |
| caspase-1 | Forward  Reverse | ACAAGGCACGGGACCTATG  TCCCAGTCAGTCCTGGAAATG |
| GSDMD | Forward  Reverse | CTGGGTCTTGCTAGAAGAATGTGG  CTGGCCTAGACTTGACAATAGGAAC |
| GAPDH | Forward  Reverse | GGGTGTGAACCACGAGAAAT  CCTTCCACAATGCCAAAGTT |

**Supplementary Table 2** Primary antibodies used in WB.

| **Antibody** | **Source** | **Company** | **Code** | **Dilution** |
| --- | --- | --- | --- | --- |
| Anti-Claudin-1 | Rabbit | Proteintech | 28674-1-AP | 1:3000 |
| Anti-Occludin | Rabbit | Proteintech | 27260-1-AP | 1:10000 |
| Anti-ZO-1 | Rabbit | Proteintech | 21773-1-AP | 1:10000 |
| Anti-GPX4 | Rabbit | FineTest | FNab03622 | 1:1000 |
| Anti-HO-1 | Rabbit | HUABIO | ER1802-73 | 1:2000 |
| Anti-iNOS | Rabbit | Proteintech | 18985-1-AP | 1:2000 |
| Anti-NRF2 | Rabbit | FineTest | FNab05855 | 1:5000 |
| Anti-IRAK-M | Rabbit | ABclonal | A5467 | 1:1000 |
| Anti-NLRP3 | Rabbit | Abmart | P60622R3 | 1:2000 |
| Anti-caspase-1 | Rabbit | Affinity | AF5418 | 1:2000 |
| Anti-GSDMD | Rabbit | Proteintech | 20770-1-AP | 1:5000 |
| Anti-β-actin | Rabbit | Proteintech | 66009-1-Ig | 1:50000 |

**Supplementary Table 3** Primary antibodies used in immunofluorescence assays.

| **Antibody** | **Source** | **Company** | **Code** | **Dilution** |
| --- | --- | --- | --- | --- |
| Anti-GPX4 | Rabbit | FineTest | FNab03622 | 1:200 |
| Anti-HO-1 | Rabbit | HUABIO | ER1802-73 | 1:500 |
| Anti-iNOS | Rabbit | Proteintech | 18985-1-AP | 1:200 |
| Anti-NRF2 | Rabbit | FineTest | FNab05855 | 1:200 |
| Anti-IRAK-M | Rabbit | ABclonal | A5467 | 1:200 |
| Anti-NLRP3 | Rabbit | Abmart | P60622R3 | 1:200 |
| Anti-caspase-1 | Rabbit | Affinity | AF5418 | 1:200 |
| Anti-GSDMD | Rabbit | Proteintech | 20770-1-AP | 1:200 |

**Supplementary Figure 1** GABA_B_R activation enhances the antioxidant capacity of peritoneal macrophages in mice 24 hours post SEZ infection. **(A)** Immunofluorescence detection of oxidative stress proteins GPX4, HO-1, iNOS, and NRF2 (green) in peritoneal macrophages. Nuclei are counterstained with DAPI (blue). Scale bar, 200 μm.

**Supplementary Figure****2** GABA_B_R activation blocks inflammatory signaling pathway in peritoneal macrophages upon mice 24 hours post SEZ infection. (A) Immunofluorescence staining of peritoneal macrophages for inflammatory proteins IRAK3, NLRP3, caspase-1, and GSDMD (green) and for nuclei (DAPI, blue). Scale bar, 200 μm.

**Supplementary Figure****3** IRAK3 knockout impairs antioxidant capacity of peritoneal macrophages in mice 24 hours post infection. **(A)** Immunofluorescence detection of oxidative stress proteins GPX4, HO-1, iNOS, and NRF2 (green) in peritoneal macrophages. Nuclei were counterstained with DAPI (blue). Scale bar, 200 μm.

**Supplementary Figure****4** IRAK3 knockout potentiates inflammatory signaling of peritoneal macrophages in mice 24 hours post infection. **(A)** Immunofluorescence staining shows inflammatory proteins NLRP3, caspase-1, and GSDMD (green) in peritoneal macrophages, with nuclei counterstained by DAPI (blue). Scale bar, 200 μm.
